# The evolution of fungal substrate specificity in a widespread group of crustose lichens

**DOI:** 10.1098/rspb.2018.0640

**Published:** 2018-10-17

**Authors:** Philipp Resl, Fernando Fernández-Mendoza, Helmut Mayrhofer, Toby Spribille

**Affiliations:** 1 Faculty of Biology, Department I, Systematic Botany and Mycology, University of Munich (LMU), Menzinger Straße 67, 80638 München, Germany; 2 Institute of Biology, Division of Plant Sciences, NAWI Graz, University of Graz, Holteigasse 6, 8010 Graz, Austria; 3 Department of Biological Sciences CW405, University of Alberta, Edmonton, Alberta, Canada T6G 2R3

**Keywords:** diversification, fungal niche, niche, phylogenetic comparative methods, phylogenetic uncertainty, symbiosis

## Abstract

Lichens exhibit varying degrees of specialization with regard to the surfaces they colonize, ranging from substrate generalists to strict substrate specialists. Though long recognized, the causes and consequences of substrate specialization are poorly known. Using a phylogeny of a 150–200 Mya clade of lichen fungi, we asked whether substrate niche is phylogenetically conserved, which substrates are ancestral, whether specialists arise from generalists or vice versa and how specialization affects speciation/extinction processes. We found strong phylogenetic signal for niche conservatism. Specialists evolved into generalists and back again, but transitions from generalism to specialism were more common than the reverse. Our models suggest that for this group of fungi, ‘escape’ from specialization for soil, rock and bark occurred, but specialization for wood foreclosed evolution away from that substrate type. In parallel, speciation models showed positive diversification rates for soil and rock dwellers but not other specialists. Patterns in the studied group suggest that fungal substrate specificity is a key determinant of evolutionary trajectory for the entire lichen symbiosis.

## 1. Background

Lichens are frequently held up as exemplary environmental indicators owing to their sensitivity to abiotic conditions [1]. The narrow ecological amplitudes of lichens have been attributed to the need to satisfy the physiological requirements of their symbiotic components [2,3]. Central to a lichen's overall realized niche, in addition to climate and precipitation chemistry, is its substrate preference. While a small number of lichen symbioses occur over a wide range of substrates, flourishing on rock, organic soil, tree bark and wood, the large majority of lichens have narrower ranges of substrate use, so much so that substrate has for decades been used as a surrogate for subtle morphological characters to recognize lichens, literally as a key character [4,5]. In the narrowest cases of substrate affinity, a specific lichen symbiosis may occur abundantly on one substrate type but not colonize adjacent others despite massive diaspore rain. These are considered to be substrate obligates [6].

Evolutionary biologists have been keen to identify universal patterns associated with niche width [7–9]. Prevailing models of niche width evolution assume single organisms in a competition for resources [10,11]. Under this assumption, the use of a wide range of resources must come at a cost, or else all species would be generalists [7]; selective pressure would result in narrowing niches [12]; and narrowing niches would in turn lead to greater species turnover in evolution [13], through increased speciation being balanced by the greater extinction risk of narrow niches [12]. It is however becoming evident that symbiosis, both mutualistic and antagonistic, may tip the scales of niche evolution [14,15]. Takeover of a functional role by a second symbiont can lead either to narrower niches if genes are lost in the first symbiont owing to relaxed selection [16,17], or to ecological range expansion if a symbiont switch brings new functionalities [18]. That closely related fungi can consort with different species of alga [19] suggests that in lichens, at least, switches are common over evolution. Any given lichen symbiont pedigree may have been associated with different symbionts, or different numbers of symbionts, over evolutionary timescales, an attribute that makes them attractive systems in which to study the effects of symbiosis on niche.

The last decades have seen at least three major changes to our understanding of lichens that frame how we assess niche breadth and the symbiotic relationships that potentially affect it. The first change concerns the circumscription of the species itself. Historically, lichens were classified using a mixture of traits assumed only in symbiosis and other, purely fungal characteristics such as spore size; the totality was called a lichen species. Assumed evolutionary groupings, such as the genus *Lecidea*, included dozens of interdigitated species that were specialized for rock, bark or wood [20–22]. In 1950, in order to rectify the nomenclatural instability arising from the recognition of lichens as multi-domain symbioses, the name of a lichen was anchored to that of its presumed single fungus by a change to the code of nomenclature [23]. Molecular phylogenetic studies of the fungus have since resulted in drastically changed species circumscriptions, with some species split into many narrower ‘cryptic species’ and others more broadly delimited, with downstream consequences for (re-)assessing niche breadth [24,25]. The second change is another by-product of fungal molecular phylogenetics: species once thought closely related have often turned out not to be [21]. We now know that the rock-dwelling species of *Lecidea,* for instance, are only distantly related to those found on bark and wood, placed in other genera and families altogether [22,26]. The third change concerns the nature of the symbioses themselves. Lichens were long thought of as a neat twosome of a fungus and alga, but metagenomic data are unearthing evidence of additional constituent fungi, of yet unknown function [27] as well as suites of algal lineages, rather than a single alga, present in common lichens [28,29]. Evidence is likewise building that bacterial assemblages influence lichen symbioses [30].

The extraordinary substrate specificity of many lichens raises intriguing questions about how the range of substrate use evolved and under what circumstances it switches. In the present study, we ask four specific questions about niche breadth evolution in the constituent fungus in an ancient and taxonomically well-studied group of crustose lichens: (i) is substrate affinity phylogenetically conserved in the constituent fungus? (ii) what are ancestral substrate types in this group? (iii) is there evidence for specialists evolving from generalists or vice versa? and (iv) how does specialization correlate with patterns of speciation and extinction? Our study group represents a cross-section of different kinds of substrate specificity and niche breadth and may serve as a good test case for evolutionary niche studies in a lichen symbiont.

## 2. Material and methods

### (a) Study system

We focused on lichens formed by members of the ascomycete families Trapeliaceae and Xylographaceae (hereafter: trapelioid lichens). The constituent fungi of trapelioid lichens form a monophyletic group, which has been well studied from taxonomic and phylogenetic perspectives [6,31–34]. Trapelioid fungi began diversifying about 150–200 Ma BP [35]. The lichens in which they occur are exclusively crust-forming and establish physical bonds with a wide range of mineral and organic substrates. They can occur on multiple (generalist) or only one (specialist) substrate type, which can be carbohydrate-rich (e.g. wood and bark) or carbohydrate-poor (rock).

Our taxon set consists mostly of specimens and sequences published by Resl *et al*. [32] and Schneider *et al*. [33], augmented with some new data (electronic supplementary material, table S1). In *Placopsis*, we have considered as separate species the operational taxonomic units estimated by Schneider *et al*. [33], although they have yet to be formally described as species. Sequences were generated following methods and primers described in [32].

### (b) Estimating taxon sampling completeness

To account for bias in species capture, we estimated the number of known species in each group using one of the largest databases for fungal taxonomy, Index Fungorum (www.indexfungorum.org; accessed January 2018). We checked every trapelioid genus except the recently described *Ducatina* and recorded the total number of described species. Additionally, taxonomic and evolutionary knowledge on trapelioid lichens accumulated over the years [6,31–34] allows us to estimate the expected total species number of the group with confidence. We then calculated per cent ratios of total known versus included species per genus in our dataset (electronic supplementary material, figure S1). Whenever possible, we performed analyses under multiple sampling regimes.

### (c) Chronogram estimation

We assembled a dataset of eight fungal loci including mitochondrial ribosomal (mtSSU), nuclear ribosomal (ITS, SSU, LSU) and nuclear protein-coding genes (RPB1, RPB2, MCM7 and EF1α; abbreviations following [32]). DNA isolation, polymerase chain reaction and Sanger sequencing were performed as in [32] and [33]. Alignments were generated for each locus using MAFFT [36] following our *phylo-scripts* pipeline [32,37]. Using BEAST 2.2.4 [38], we estimated time-calibrated phylogenies for the concatenated dataset using locus-independent site and clock models and a birth-death tree prior. We chose the best substitution models according to the Akaike information criterion (AIC) for each locus with JModelTest 2 [39].

### (d) Tree selection and phylogenetic uncertainty

For downstream analyses, we consistently used either (i) a random subset of 100 trees selected from the BEAST posterior distribution for analyses using multiple trees to account for phylogenetic uncertainty, or (ii) a maximum clade credibility (MCC) tree when only single topologies could be used (Bayesian analysis of macroevolutionary mixtures (BAMM)). The MCC topology was estimated in TreeAnnotator 2.2.1 after discarding the first 15% of trees as burn-in.

### (e) Coding ecological and substrate preference characters

We coded ecological strategies as two sets of categorical variables. Specialization (GS) was treated as binary (generalist: growing on multiple substrates; specialist: growing on single substrate), while the preferred substrate (PS) was coded as multi-state (rock, soil, bark, wood, other lichens). We derived substrate use data from our own collections as well as from herbarium collections (BG, GZU, UPS), species catalogues [40], identification keys [4,5] and recent monographs (*Placopsis*: [41], *Xylographa*: [31]). A fungal-species was considered a specialist when greater than 95% of its global occurrences were from one substrate.

### (f) Testing for phylogenetic signal

We estimated phylogenetic signal of the PS variable using two simulation-based multi-tree approaches: (i) recursive use of Pagel's *λ* [42], and (ii) comparison of the distribution of cophenetic distances based on the assumption that closely related species are ecologically similar [43,44]. For further details, see the electronic supplementary material.

### (g) Ancestral state reconstruction

**Figure 1.**
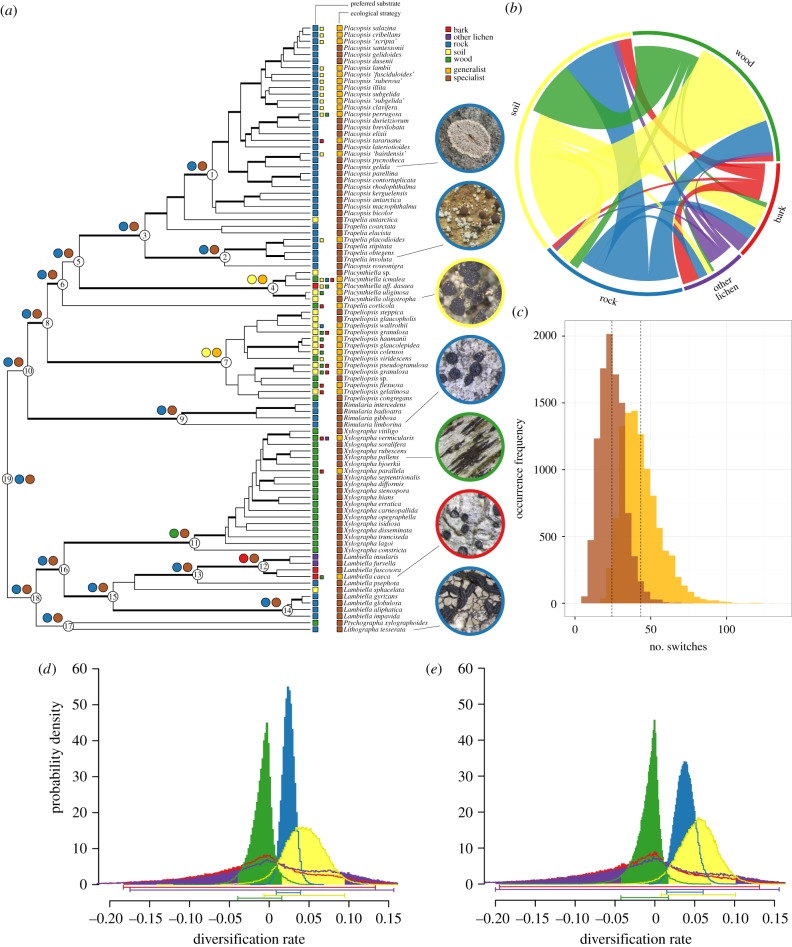
Substrate evolution of trapelioid fungi. (*a*) MCC tree from BEAST2 analyses. Thick branches indicate posterior probability support greater than 95%. Coloured squares indicate preferred substrate (left column) and ecological strategy (right column) characters. Small squares refer to substrates on which a species has also been found infrequently. Quotes indicate working names of undescribed taxa. Most likely ancestral states of individual nodes are indicated with coloured circles. Reconstructions are based on 30 and 20 different evolutionary models and 100 trees for the PS and GS characters, respectively. Individual results are provided as the electronic supplementary material, figures S4–S117. (*b*,*c*) Transitions between different character states according to 10 000 stochastic maps created for 100 trees. (*b*) Switches between different preferred substrates. Indented sides of connectors indicate transition origins. Numbers underlying this figure are given in the electronic supplementary material, table S2. (*c*) Distribution of the number of switches between generalist and specialist states. Dotted lines indicate means. Orange, specialist to generalist switches; brown, generalist to specialist switches. (*d*,*e*) Probability density distribution of diversification rate (*λ*–*μ*) estimates calculated with MuSSE for 100 trees. Scenarios imposing (*d*) 100% species sampling completeness and (*e*) our own estimate of species sampling completeness.

We employed two maximum-likelihood (ML) approaches based on implementations in ape [45] and corHMM [46] and stochastic character mapping implemented in phytools [47] to reconstruct ancestral states of the GS and PS characters at the main 19 internal nodes of the Trapeliales phylogeny (figure 1). To provide a summary of ancestral character reconstruction while accounting for bias introduced by methods, models and tree topologies, we developed a recursive strategy. First, we fitted models with all possible parameter combinations available for each method to each tree. Then we only considered as the most probable ancestral state the one recovered most often across all 24 analyses and tree topologies which are shown with the MCC tree. For each node, we created a plot indicating the number of trees for which a particular ancestral character state was estimated under all possible models for one method.

### (h) Reconstructing transitions between substrates

Transitions between the different character states were counted from unconstrained stochastic character mappings as created for ancestral state reconstructions (see above). The cumulative results of the 10 000 alternative transition histories were summarized numerically and are presented as histograms for binary ecological strategy characters (GS) and as circle plots for multi-state substrate characters (PS).

### (i) Testing substrate ‘no-switch’ scenarios

To test whether models prohibiting certain substrate transitions are more likely given our set of trees, we created 30 transition rate matrices describing different scenarios of character change. We compared these constrained models on each of the 100 trees from the BEAST posterior distribution of trees. The tested models include all possible combinations of no-switch scenarios for our multi-state substrate character. Each model and each tree was subjected to a ML ancestral state estimation using ape [45]. We then calculated and ranked models from best to worst according to AIC score comparisons for each tree. To see which models scored best over all trees, we calculated for how many trees a specific model would be the best, second best, third best and so on. We then searched for the models for which the majority of trees were recovered in the first five ranks.

### (j) Modelling of diversification rates

We characterized the diversification dynamics of the trapelioid clade with character-independent BAMM 2.6.0 [48] on the MCC tree topology as well as with character-dependent multi-state speciation and extinction (MuSSE) [49,50] models on a set of 100 tree topologies (see above). We analysed the output of the BAMM analyses with BAMMtools [51]. We combined the posterior samples from all MuSSE runs and created density plots for diversification rate (speciation–extinction). To identify significantly different speciation rates, we compared the obtained probability distributions with the Mann–Whitney tests for all possible combinations of characters.

Owing to the lack of consensus on the performance and suitability of the SSE approach to model evolutionary trends [52], we tested the extent to which the modelled diversification rates respond to the phylogenetic tree alone without a further connection to the distribution of characters on the tree according to the method described in [52].

## 3. Results

### (a) Phylogenetic reconstruction confirms previous results

Our phylogenetic results (figure 1*a*) recover the same relationships found in previous studies ([32]; fig. 4) and confirm the recently recognized two-family split between Trapeliaceae and Xylographaceae [32]. We could also confirm the paraphyly of *Trapelia* and *Placopsis* [32,33] and the monophyly of all other genera. A table with all used sequences is provided in the electronic supplementary material, table S1.

### (b) Substrate association displays strong phylogenetic signal

The distribution of substrate characters displays strong phylogenetic signal according to both simulation approaches. Model fit was significantly better for the real character data compared to all randomizations under multiple scenarios of Pagel's *λ* > 0 (electronic supplementary material, figure S2; *p* < 0.05) except when *λ* = 0 and the tree is one single polytomy (electronic supplementary material, figure S2). The mean tip-to-tip distance method yielded similar results (electronic supplementary material, figure S3). In greater than 95% of simulations, the mean tip-to-tip distance between tips with the same character coding was significantly shorter for the real character distribution (electronic supplementary material, figure S3; *p* < 0.05) compared to randomizations. For bark-growing species, the mean distance of the real distribution was significantly shorter in 75 of the simulations (*p* < 0.05; electronic supplementary material, figure S3), which probably referred to the low number of tips with that character state.

### (c) Ancestral substrate use and amplitude

We recovered evidence for ancestral ecological strategy and preferred substrate use of 19 nodes representing all currently recognized trapelioid genera, as well as important nodes of the tree backbone (figure 1*a*). Our approach is based on three methods imposing 30 and 20 different models for the preferred substrate and ecological strategy characters, respectively (electronic supplementary material, figures S4–S117). How many methods recovered which ancestral states in figure 1*a* are given in the electronic supplementary material, table S6. For all extant species groups, we recovered the currently preferred substrate as its ancestral substrate. We estimated rock as the ancestral substrate for *Placopsis* (node 1; 29 out of 30 methods), *Trapelia* (node 2; 29 out of 30 methods), *Rimularia* (node 9; 29 out of 30 methods), and the *Lambiella impavida* group (node 14; 29 out of 30 methods), wood for *Xylographa* (node 11; 28 out of 30 methods) and soil for *Placynthiella* (node 4; 28 out of 30 methods). The ancestor of *Trapeliopsis* was found to be soil-growing (node 7; 22 out of 30 methods). The most recent common ancestor (MRCA) of all trapelioid fungi most likely grew on rock (node 19; 16 out of 30 methods) and was a specialist (node 19; 13 out of 20 methods).

### (d) Greater frequency of generalist to specialist, soil/rock to bark/wood switches

Stochastic mapping returned mixed results for transitions between preferred substrate types. We found a higher number of transitions to bark-, lichen- and wood-growing species than to rock and soil, and conversely, a higher number of transitions away from rock- and soil-growing species to bark, lichens (as substrate) and wood (figure 1*b*, electronic supplementary material, table S2). For the GS character set, a higher number of transitions to the specialist state indicates that, despite the generalist state being in theory derived, generalists are more likely to spawn specialists than vice versa (figure 1*c*).

### (e) Some types of substrate switches are rarer than others

**Figure 2.**
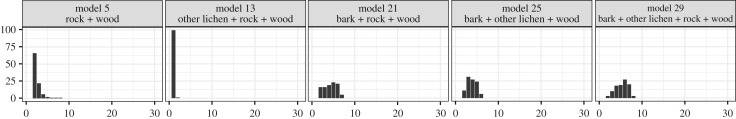
Results of evolutionary dead-end analyses based on comparison of 30 models of character change and 100 trees. The five models imposing different evolutionary dead-end scenarios that continuously (for the majority of trees) ranked among the five best models according to their AIC scores. Numbers behind this figure as well as plots for all additional models are given in the electronic supplementary material, table S3 and figure S123. *x*-axis: rank of model among the 30 tested models. *y*-axis: number of trees for which this model scored a particular rank.

Comparison of constrained ancestral state reconstruction models reveals the rarity with which wood- and rock-growing groups (and to a lesser extent bark- and lichen-colonizing lineages) switch substrates. Of 30 tested models (electronic supplementary material, figure S123 and table S3), the five best-performing models (figure 2) are those in which switches away from wood, rock, other lichens (as a substrate) and bark are specifically constrained in combination (figure 2). All five of the best-performing models included wood, but models that constrained switches away from any one of these substrates in isolation (including wood) without simultaneously constraining others performed significantly worse (electronic supplementary material, table S3 and figure S123). The only substrate absent from the best-performing models was soil, indicating that switches away from soil in any combination result in poor model performance, i.e. such switches were likely and soil inhabiting species are less constrained to their substrate. For seven of the 3000 tested model/tree combinations, ape's ace function failed to return any ML solution (summarized in the electronic supplementary material, table S4). We thus excluded those seven data points from figure 2 and the electronic supplementary material, figure S123 and table S3.

### (f) Elevated diversification rate of soil and rock growers in MuSSE

**Figure 3.**
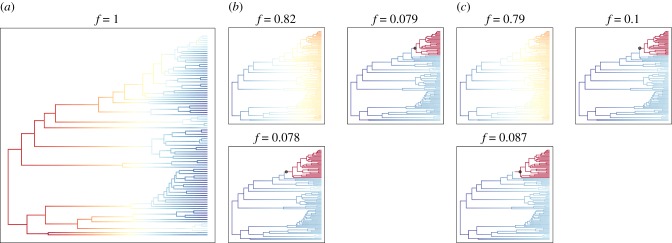
A 95% credibility set of speciation rate shift configurations obtained from analysing the MCC tree with BAMM. Warm colours indicate high speciation rates and cold colours indicate low speciation rates in different parts of the tree relative to the overall rate of the tree. Small circles indicate abrupt rate changes. *f*, occurrence frequency of individual rate shift scenarios in the 95% credibility set of rate shifts. (*a*) Scenario imposing 100% species sampling completeness. (*b*) Scenario imposing sampling completeness according to indexfungorum.org. (*c*) Scenario imposing species sampling completeness according to own estimates.

To assess the effect of character on diversification dynamics, we analysed scenarios assuming 100% sampling completeness (A; figure 3*a*) and our own estimates of the true number of species (B; figure 3*b*). A scenario based on species number estimates from indexfungorum.org was not applicable because we also included data from undescribed species (e.g. in *Placopsis*). In both tested scenarios, we found significantly elevated diversification rates for rock and soil growers (figure 1*d,e*; all comparisons Mann–Whitney test *p* < 0.01; see the electronic supplementary material, table S5). Wood-, bark- and lichen-inhabiting species had diversification rates close to zero (figure 1*d*,*e*). Individual plots for estimates of *λ* and *μ* are provided as electronic supplementary material, figure S118. Simulations to test if the tree topology obscures character-dependent speciation rate show that the MCC tree is potentially affected by slightly elevated type 1 error rates (electronic supplementary material, figure S119). For low transition rates (*q* = 0.01, *q* = 1), approximately 25 of the tests detected false correlations of character distribution and speciation rate dynamics. For very high transition rates (*q* = 10), an incorrect connection was reported in approximately 90% of simulations (electronic supplementary material, figure S119).

### (g) Character-independent speciation rate analysis (Bayesian analysis of macroevolutionary mixtures)

Analysing speciation rate in a character-independent framework allowed us to include three scenarios of sampling completeness: (A) imposing all species present (figure 3*a*), (B) relying on information from indexfungorum.org (figure 3*b*), and (C) relying on our own estimates (figure 3*c*). Assuming complete species sampling the only rate shift configuration recovered contains no significant speciation rate shift (cumulative frequency = 1; figure 3*a*). In scenarios B and C, the single most often observed shift configuration also lacks abrupt rate shifts (cumulative frequency = 0.82 and 0.79; figure 3*b*,*c*). In a subset of model runs, we detected speciation rate shifts along the evolution of the *Placopsis* clade in scenarios B and C (figure 3*b*; cumulative frequency = 0.157 and figure 3*c*; cumulative frequency = 0.187).

## 4. Discussion

Whatever the proximate factors specifically tying trapelioid lichens to their substrates are, they have clearly left an indelible mark during millions of years of evolution in the constituent fungus. Because five types of obligate specificity are present in the trapelioid evolutionary tree, in addition to generalists, and because the MRCA existed so long ago [35], it is possible to discern a pattern at least three layers deep, i.e. consistent with evolution from specialists to generalists and back again. All of our ancestral state reconstructions support the overall trapelioid MRCA being a rock-dwelling specialist. At the same time, our analyses show that transitions from generalist to specialist are more frequent than vice versa. This is only possible to derive from the same data because switches to generalist happened multiple times, most deeply in the tree at the MRCA to modern *Trapeliopsis* species, and again separately for *Placynthiella* (a third deep transition may have happened again for *Trapelia corticola*, but ancestral state recovery from this node may suffer because only a single-species data point is extant).

### (a) Mechanisms that enforce substrate specificity

The underlying causes of substrate specificity in lichens have yet to be explored in depth. Contemporary approaches to identifying the source of lichen–substrate interactions reflect two theoretical models. Under the prevalent (though usually unstated) model, lichens are seen as nutritionally autonomous on account of photobionts supplying their carbohydrate needs [3]. Consistent with this, many lichen ecologists analyse lichen diversity composition, like that of plants, as a response to abiotic gradients and competition (e.g. [53,54]), assuming lichens are free to colonize all surfaces. An alternative model allows that carbohydrate sourcing may differ among lichens, or over time. Echoing an early mechanistic explanation by Schwendener [55], several authors have either proposed hidden saproby [56] or suggested that the inability of some lichens to establish on adjacent, diaspore-drenched but unsuitable substrates may reflect an underlying trophic relationship with the obligate substrate [31,57,58]. It is also now well known that the constituent fungi in lichens are polyphyletic. Some are closely related to saprobic or even pathogenic fungi, such as in Dothideomycetes [59], Arthoniomycetes [60] and Eurotiomycetes [61], and it may not be safe to assume that the corresponding repertoire of carbohydrate-active enzymes is lost upon lichenization.

A more commonly invoked reason for substrate specificity is preference for specific chemical environments, often expressed in terms of cation ratios and pH [53,54,62], potentially also extending to carbohydrate chemistry [63]. In some cases, species responses to these factors suggest, if anything, a broadening of the niche by facilitating species' occurrence on multiple substrate types, e.g. through calcareous dust exposure enabling otherwise rock-dwelling species to occur on wood and bark [64]. In theory, such requirements could also enforce the occurrence of a species on a particular substrate, but cation content may be too easily compensable for it to be solely responsible for substrate obligacy. Ultimately, poorly understood physiological attributes such as relationship to porosity and water uptake and biophysical attributes such as fungus-specific surface adhesion [65] should also be explored for enforcing mechanisms.

### (b) Specialization ‘beyond all exit ramps’?

Our data indicate that the evolution of specificity is in some cases less the acquisition of a specialization than the loss of ability to colonize other substrates. By selectively constraining transition matrices to prevent substrate switches, we assessed which scenarios are more likely, given the data. The most likely were those that prevented transitions out of some combination of wood and either rock, bark or other lichens. Wood alone did not score among the four best models because model-fitting included all possible permutations; however, when the five individual substrate types were constrained alone, wood-only again performed best. Switches from soil to other substrates, by contrast, appear likely given the data. If this tentative pattern holds up, it suggests that not all specializations are equal, in other words, some species may reach a point ‘beyond all exit ramps’ (or ‘dead end’ [12]) where acquisition of adaptive traits that lead out of their specialization type becomes increasingly unlikely. This scenario is strikingly similar to the phenomenon that has been called compensated trait loss [17]. Our data only allow us to see the imprint of loss in ecological and evolutionary data, but we do not know specifically which functions were lost or gained, nor can we assume that functional plasticity follows the same patterns in all lichens. However, in trapelioids, a clue can be gleaned from the fact that in *Xylographa*, at least two species have secondarily lost lichenization, i.e. are no longer associated with algae (*Xylographa constricta* and another yet undescribed species not included here). Several other cases of delichenization in Ostropomycetidae (e.g. most Ostropales: [58,66]; *Agyrium rufum:* [67]) suggest that this is not a rare phenomenon in lichens with intimate substrate associations. In such cases, it is tempting to conclude, as Schwendener [55] did, that some lichen-forming fungi never actually lost the ability to obtain and use exogenous carbohydrates.

The ‘beyond all exit ramp’ explanation might be a good fit for the fungus if it were not for the fact that we are studying it in isolation, disembodied from the symbiosis in which we assume it completes its life cycle. The genus *Placopsis* may present an example of a lineage that has found an ‘exit ramp’ through symbiotic innovation, despite a history of specialization. We have previously highlighted the increase in thallus size and complexity relative to *Trapelia* that happened in the whole lichen after acquisition of a cyanobacterial symbiont [33], which as a nitrogen fixer acts as a natural fertilizer for the thallus [68]. Our substrate data now suggest that *Placopsis* species not only evolved larger size but also escaped substrate constraints, particularly in the *Placopsis lambii* clade.

MuSSE invokes a much different model in that it allows for modelling effects of character states on both speciation and extinction processes [49]. The results of MuSSE, which show greater net speciation for soil and rock dwellers, are partially driven by the surge in speciation in *Placopsis*. As *Placopsis*, a predominantly rock-dwelling genus, is the only one in the dataset to have also acquired an additional cyanobacterial symbiont, it is likely that the MuSSE models do not solely reflect substrate effect.

### (c) Niche width, effective population size and speciation rate

The assignment of species to one or more of five different substrate types is the best approximation of niche width we could find for every species in our dataset. To some extent, however, this approach obscures the true extent to which some niches can be much narrower, or others broader. Some *Xylographa* species inhabit only driftwood (e.g. *Xylographa opegraphella*), while others grow only on hard conifer wood (*Xylographa stenospora*), soft conifer logs (*Xylographa septentrionalis*), oak logs (*Xylographa lagoi*), snags (*Xylographa difformis*) or charred wood (*Xylographa isidiosa*; [31]) and may be correspondingly rare. On the other end of the spectrum, other species in our dataset colonize not only soil but various types of soil of different pH, logs, plant detritus and tree bark (e.g. *Trapeliopsis granulosa*, *Trapeliopsis flexuosa*) and are correspondingly common. It follows that changes in niche amplitude correlate with differences in local and regional population sizes, which in turn affect gene flow (niche breadth with speciation: [69]): smaller, more fragmented populations will be more likely to speciate over geographical time, and are also more prone to extinction. This may explain why we have fewer species growing on soil than on other substrate types in our dataset, and why their character-independent diversification rates are flat (figure 3*a–c*; electronic supplementary material, figures S120, S121 and S122), notwithstanding increased diversification rates in character-dependent analyses (figure 1*d*,*e*). Parsing the effect of niche width on effective population size would require data more fine-grained than anything available in this study.

### (d) Areas of uncertainty in using phylogenetic comparative methods

#### (i) Sampling completeness estimates

It has not escaped our notice that the group which exhibits character-independent speciation rate increases has recently been subjected to a systematic revision (*Placopsis*). However, we were aware of this disparity and made an effort to offset potential estimation bias. Genera such as *Lambiella, Trapelia* and *Xylographa* have seen considerable recent systematic investment, including by us, and we are also revising *Trapeliopsis*, fully aware of undescribed species in this group. We are confident that we have provided realistic relative estimates of species numbers, given the geographical regions and systematic work available to us and we have incorporated this information whenever possible in our analyses.

#### (ii) Rate variation

Both trait-dependent and independent speciation and extinction models have recently been criticized [52,70,71]. In the absence of a scientific consensus on how to evaluate model inadequacies, we applied both approaches with caution. We tested for intrinsic rate variation [52] to identify speciation rate shifts in trait-dependent models. We found that the overall speciation rate of different trapelioid groups is relatively constant (electronic supplementary material, figures S120, S121 and S122). Our MCC tree is affected by moderately increased type-1 error rates in MuSSE analyses, especially for high transition rates (electronic supplementary material, figure S118). Increased error rates are, however, much lower than in the example cited by Rabosky & Goldberg [52] and we found the rates of character change to be very low. Although speciation rate analyses largely concur (figures 1*d*,*e* and 3), we treat these results with caution. It remains unclear how to evaluate shortcomings of character-independent analyses [70,71], e.g. with sensitivity analyses similar to the character-dependent models. That said, we have sought to take into account and test potential model bias wherever possible and we present alternative interpretations to our results.

### (e) Conclusion

The study of niche evolution in lichen fungi is in its early phases, but already patterns are evident from substrate use that are broadly relevant to the study of niche evolution. First, lichen–substrate relationships in trapelioids are phylogenetically conserved and stable over millions of years. If better measures of niche breadth could be developed, it may be possible to detect a simultaneous narrowing of niche and increase in species turnover in some clades, e.g. *Xylographa*. Second, a directionality from generalists to specialists is broadly consistent with other results from the niche breadth evolution literature. At the same time, signs of niche broadening in *Placopsis* suggest that symbiont switching could influence fungal evolution. The role of green algal symbionts in switching throughout the trapelioid tree is difficult to assess, in part because trapelioids, like other lichens, can contain multiple algal species *in vivo* [28,29], and assigning a single algal symbiont to a fungal species may be biologically unrealistic. Disentangling algal entourages, e.g. through metagenomics, would be key to tracking the effect of symbiosis on niche evolution in the future.

## Supplementary Material

Electronic supplements

## Supplementary Material

Table S1

## Data accessibility

Data, R code and software control files (e.g. for BAMM) underlying analyses are available on the Dryad Digital Repository: http://dx.doi.org/10.5061/dryad.40sh435 [72]. A complete list of all used sequence vouchers, an extended description of the used methods as well as results from individual analyses are provided as the electronic supplementary material.
