# Supplementary material for: The evolution of fungal substrate specificity in a widespread group of crustose lichens
Source: Proc Biol Sci. 2018 Oct 17;285(1889):20180640. doi: 10.1098/rspb.2018.0640 (PMC6234878; doi:10.1098/rspb.2018.0640)
Supplement: Electronic supplements [file rspb20180640supp1.pdf]

Supplementary Files for the Manuscript:

The evolution of fungal substrate specificity in a widespread group of crustose lichens.
Proceedings of the Royal Society B: Biological Sciences. doi: 10.1098/rspb.2018.0640

Philipp Resl
Fernando Fernández Mendoza
Helmut Mayrhofer
Toby Spribille

Aug 24th, 2018

Contents

Information . . . 2
Extended Materials and Methods . . . 2
Figure S1 - Relative sampling completeness . . . 6
Figure S2 - Phylogenetic informativeness simulation 1: Pagel's Lambda . . . 7
Figure S3 - Phylogenetic informativeness simulation 2: Distance method . . . 9
Figure S4-S22 - Results of ancestral state estimation of the preferred substrate character with ace for 19 nodes of the trapelioid phylogeny . . . 11
Figure S23-S41 - Results of ancestral state estimation of the preferred substrate character with rayDISC for 19 nodes of the trapelioid phylogeny . . . 31
Figure S42-S60 - Results of ancestral state estimation of the preferred substrate character with make.simmap for 19 nodes of the trapelioid phylogeny . . . 51
Figure S61-S79 - Results of ancestral state estimation of ecological strategy character with ace for 19 nodes of the trapelioid phylogeny . . . 71
Figure S80-S98 - Results of ancestral state estimation of ecological strategy character with rayDISC for 19 nodes of the trapelioid phylogeny . . . 91
Figure S99-S117 - Results of ancestral state estimation of ecological strategy character with make-simmap for 19 nodes of the trapelioid phylogeny . . . 111
Figure S118 - Individual plots for speciation and extinction rate from MuSSE analyses . . . 131
Figure S119 - MuSSE validation simulation . . . 131
Figure S120 - BAMM rate through time plot for different trapelioid clades assuming 100% sampling completeness . . . 133
Figure S121 - BAMM rate through time plot for different trapelioid clades assuming sampling completeness according to indexfungorum.org . . . 134
Figure S122 - BAMM rate through time plot for different trapelioid clades assuming sampling completeness according to own diversity estimates . . . 135
Figure S123 - Results of blocked transition model comparison with ace based on AIC score comparison of thirty models fitted to 100 trapelioid trees . . . 136
Table S1 - Specimen and sequence vouchers used in this study . . . 137
Table S2 - Mean number of character state transitions for the preferred substrate character set . . . 137
Table S3 - Results from evolutionary dead end scenario analyses in tabular form. . . . 138
Table S4 - Individual models of dead end analyses for which ace failed to return results . . . 139
Table S5a - Summary MuSSE diversification rate 100% . . . 139
Table S5b - Summary MuSSE diversification own diversity estimates . . . 139
Table S6 - Overview about the number of methods which recovered the most frequent ancestral state for 10 nodes along the trapelioid tree . . . 139

Information

This is document includes supplementary information to accompany the manuscript.

Extended Materials and Methods

Study system

We focused on lichens formed by members of the ascomycete families Trapeliaceae and Xylographaceae (hereafter: trapelioid lichens). The constituent fungi of trapelioid lichens form a monophyletic group, which has been well studied from taxonomic and phylogenetic perspectives [1-5], Trapelioid fungi began diversifying about 150-200 Ma BP [6-7]. The lichens in which they occur are exclusively crust-forming and establish physical bonds with a wide range of mineral and organic substrates. They can occur on multiple (generalist) or only one (specialist) substrate type, which can be carbohydrate-rich (e.g. wood and bark) or carbohydrate-poor (rock). Our taxon set consists mostly of specimens and sequences published by [3] and [4], augmented with some new data (Suppl. Table 1). In Placopsis we have considered as separate species the OTUs estimated by [4] although they have yet to be formally described as species. Sequences were generated following methods and primers described in [3].

Estimating taxon sampling completeness

To account for bias in species capture, we estimated the number of known species in each group using one of the largest databases for fungal taxonomy, Index Fungorum (www.indexfungorum.org; accessed Jan. 2018). We checked every trapelioid genus except the recently described Ducatina and recorded the total number of described species. Additionally, taxonomic and evolutionary knowledge on trapelioid lichens accumulated over the years [8, 1-5]) allows us to estimate the expected total species number of the group with confidence. We then calculated percent ratios of total known versus included species per genus in our dataset (Figure S1). Whenever possible, we performed analyses under multiple sampling regimes.

Chronogram estimation

We assembled a data set of eight fungal loci including mitochondrial ribosomal (mtSSU), nuclear ribosomal (ITS, SSU, LSU) and nuclear protein-coding genes (RPB1, RPB2, MCM7 and EF1alpha; abbreviations following [3]). DNA isolation, PCR and Sanger sequencing were performed as in [3] and [4]. Alignments were generated for each locus using MAFFT [9] following our phylo-scripts pipeline [3, 10]. Using BEAST 2.2.4 [11] we estimated time-calibrated phylogenies for the concatenated dataset using under locus-independent site and clock models and a birth-death tree prior. We chose the best substitution models according to the Akaike Information Criterion (AIC) for each locus with JModelTest 2 [12]. We used relaxed log-normal clocks for ITS, SSU, LSU, RPB2, MCM7 and EF1alpha. For RPB1 we set a strict clock rate as estimated for Lecanoromycetes [13]. We set a normally distributed root prior for the studied group with a mean of 150 million years as estimated by [6] and [7]. After discarding a burn-in of 15%, we combined the log and tree files from two independent 108 generation runs (sampling every 20000th tree) with LogCombiner. Using Tracer 1.5 [14] we investigated convergence diagnostics and estimated sample size (ESS>200) of model parameters.

Tree selection and phylogenetic uncertainty

For downstream analyses we consistently used either a) a random subset of 100 trees selected from the BEAST posterior distribution for analyses using multiple trees to account for phylogenetic uncertainty, or b) a maximum clade credibility (MCC) tree when only single topologies could be used (BAMM). The MCC topology was estimated in TreeAnnotator 2.2.1 after discarding the first 15% of trees as burn-in.

Coding ecological and substrate preference characters

We coded ecological strategies as two sets of categorical variables. Specialization (GS) was treated as binary (generalist: growing on multiple substrates; specialist: growing on single substrate), while the preferred substrate (PS) was coded as multistate (rock, soil, bark, wood, other lichens). We derived substrate use data from our own collections as well as from herbarium collections (BG, GZU, UPS), species catalogs [15], identification keys [16-17] and recent monographs (Placopsis: [18], Xylographa: [2]). A species was considered a specialist when >95% of its global occurrences were from one substrate.

Testing for phylogenetic signal

We estimated phylogenetic signal of the PS variable using two simulation-based multi-tree approaches: a) recursive use of Pagel's λ [19], and b) comparison of the distribution of cophenetic distances. In the first approach, we transformed each tree for eleven different values of Pagel's λ given our own character data (Table S1), and retained a distribution of AIC scores of fitted maximum likelihood models of trait evolution [20] with the R package geiger [21]. To obtain null models, we simulated 100 random character distributions for each tree while maintaining the number of individuals with different character states and repeatedly fitted a model of trait evolution. We compared the AIC score of the original model to the distribution of AIC scores from simulations with one-sided Mann-Whitney tests (considered significant if $p<0.05$) and created density plots of the obtained p values. We consider phylogenetic signal strong when the real character distribution has better fitting models than the simulated datasets even for small values of λ (when the tree topology gets more star-like). λ tree transformations and model fitting were performed using rescale() and fitDiscrete() from geiger [21].

As a second measure for phylogenetic signal, we assume that closely related species are ecologically similar [22-23] due to shared evolutionary histories. When species with the same phenotypic character are clustered in the tree, their mean tip-to-tip distance should be smaller compared to when they are randomly dispersed. First, we calculated the mean per-tree distances between all species pairs having the same character state. Next, we shuffled the original character assignments to create randomly distributed tip states and calculated means as above. We repeated this procedure 100 times per tree and compared the obtained distributions to the mean distribution from the true character assignment with Mann-Whitney tests (significance considered for $p<0.05$). We calculated tip-to-tip distances for each tree with the function cophenetic.phylo from the R package ape [24] and summarized the obtained p-values in density plots.

Ancestral state reconstruction

Different methods of ancestral state reconstruction as well as the parametrization of the different models may result in divergent or contradictory results [25]. We used three alternative multi-tree methods to be able to compare reconstruction of the ancestral states for the GS and PS variables at the main 19 internal nodes of the Trapeliales phylogeny (Figure 1). (1) We employed a maximum likelihood approach based on [26] and implemented in the ace function in ape [24], imposing models with equal (ER), symmetric (SYM) and asymmetric rates (ASR) of character evolution. Ancestral states on internal nodes were estimated jointly [27] as well as marginally using only information from the tip states. (2) We used a second maximum likelihood approach described in [28] and implemented in function rayDISC of the R package corHMM [28]. rayDISC allows uncertainty of tip states to be incorporated by imposing probabilities on tip state distributions, in this case allowing us to account for species colonizing multiple substrates (Table S1). Again we used ER, SYM and ASR models as well as joint and marginal estimations of ancestral states. Furthermore, we employed methods proposed by [20], [29] and [30] to weight the probability of ancestral states at the root of the tree, as well as rayDISC's default, which assumes equal weighting among all states. (3) We used stochastic character mapping [31] to infer posterior probabilities of ancestral states using phytools [32]. Again we used models with equal, symmetric and asymmetric rates and we estimated rate parameters for each tree using a Markov Chain Monte Carlo (MCMC) approach (Q=”mcmc”) and a time-continuous Markov model (Q=”empirical”). For each character set we created 100 maps per tree.

To provide a summary of ancestral character reconstruction while accounting for bias introduced by methods, models and tree topologies, we developed a recursive strategy. First we fitted models with all possible parameter combinations available for each method to each tree. Then we only considered as the most probable ancestral state the one recovered most often across all analyses and tree topologies. The most probable ancestral states identified across all 24 different simulations are shown with the maximum clade credibility tree. For each node we created a plot indicating the number of trees for which a particular ancestral character state was estimated under all possible models for one method.

Reconstructing transitions between substrates

To investigate the evolutionary transitions between substrate types we used stochastic character mappings created for ancestral state reconstructions (see above) under an unconstrained model (ARD). Transitions between the different character states were counted and relativized by the total number of character transitions for each tree. The cumulative results of the 10,000 alternative transition histories were summarized numerically and are presented as histograms for binary ecological strategy characters (GS) and as circle plots for multi-state substrate characters (PS).

Testing substrate "no-switch" scenarios

To test whether models prohibiting certain substrate transitions are more likely given our set of trees, we created thirty transition rate matrices describing different scenarios of character change. We compared these constrained models on each of the 100 trees from the BEAST posterior distribution of trees. The tested models include all possible combinations of no-switch scenarios for our multistate substrate character. Each model and each tree was subjected to a maximum likelihood ancestral state estimation with ace() from ape [24]. We then calculated AIC scores and ranked models from best to worst according to AIC score comparisons for each tree. To see which models scored best over all trees, we calculated for how many trees a specific model would be the best, second best, third best and so on. Then we searched for the models for which the majority of trees were recovered in the first five ranks.

Modeling of diversification rates

To characterize the diversification dynamics of the trapelioid clade we used character-independent Bayesian analysis of macroevolutionary mixtures (BAMM) [33] as well as character-dependent SSE models [29-30]. Using BAMM 2.6.0 we studied the speciation rate dynamics of the MCC tree topology. The priors and initial values for λ, λ-shifts and µ used to initialize the Bayesian BAMM analyses were estimated with setBAMMpriors [34]. BAMM was run for 107 generations saving every 1000th step. We ran three independent analyses assuming different sampling regimes (100% and two more; see above). We analysed the BAMM output with BAMMtools [34] discarding a 20% burn-in. Finally, we summarized results graphically by plotting the 95% credibility sets of rate-shift configurations and rate through time plots for individual clades as well as for the whole tree.

To investigate effects of character realizations on diversification dynamics we utilized multi-state speciation extinction models (MuSSE; [29]) implemented in diversitree [35]. Unlike BAMM, MuSSE can be used as a multi-tree method, and we took advantage of this to analyse our sample of 100 tree topologies randomly chosen from the BEAST posterior distribution. For each tree we fitted a MuSSE model using an MCMC run of 1000 generations under exponential priors and sampling every 10th generation. We combined the posterior samples from all 100 runs and created density plots for λ, µ and diversification rate (λ-µ). We repeated the analysis for different sampling scenarios according to our species completeness information. To identify significantly different speciation rates, we compared the obtained probability distributions with Mann-Whitney tests for all possible combinations of characters. The lack of consensus on the performance and suitability of the SSE approach to model evolutionary trends [36] led us to test the extent to which the modelled diversification rates respond to the phylogenetic tree alone without a further connection to

the distribution of characters on the tree. For this we implemented the method and scripts developed by [36] to simulate 100 neutral binary characters on our MCC tree under different transition rate parameters (q=0.01/0.1/1/10). We tested if these neutral characters are associated with a speciation rate increase using likelihood ratio tests on models with $\lambda\, 0 = \lambda\, 1$ and $\lambda\, 0$, $\lambda\, 1$, respectively. We also simulated an alternative null distribution of pure-birth trees of the same size as our MCC tree without rate heterogeneity. We performed simulations for 100 neutral binary characters for different values of q as above and tested if the character distributions were associated with speciation rate changes.

References

1. Spribille T, Thor G, Bunnell F, Goward T. 2008 Lichens on dead wood: species-substrate relationships in the epiphytic lichen floras of the Pacific Northwest and Fennoscandia. Ecography 34, 741–750. (doi: 10.1111/j.1600-0587.2008.05503.x)
2. Spribille T, Resl P, Ahti T, Pérez-Ortega S, Tønsberg T, Mayrhofer H, Lumbsch HT 2014 Molecular systematics of the wood-inhabiting, lichen-forming genus Xylographa (Baeomycetales, Ostropomycetidae) with eight new species. Symb Bot Upsal 37, 1–93.
3. Resl P, Schneider K, Westberg M, Printzen C, Palice Z, Thor G, Fryday A, Mayrhofer H, Spribille T. 2015 Diagnostics for a troubled backbone: testing topological hypotheses of trapelioid lichenized fungi in a large-scale phylogeny of Ostropomycetidae (Lecanoromycetes). Fungal Divers 73, 239–258. (http://doi.org/10.1007/s13225-015-0332-y)
4. Schneider K, Resl P, Spribille T. 2016 Escape from the cryptic species trap: lichen evolution on both sides of a cyanobacterial acquisition event. Mol Ecol 25, 3453–3468. (doi: 10.1111/mec.13636)
5. Orange A. 2018 A new species-level taxonomy for Trapelia (Trapeliaceae, Ostropomycetidae) with special reference to Great Britain and the Falkland Islands. The Lichenologist 50, 3–42. (http://doi.org/10.1017/S0024282917000639)
6. Prieto M, Wedin M. 2013 Dating the diversification of the major lineages of Ascomycota (Fungi). PLoS ONE 8, e65576. (https://doi.org/10.1371/journal.pone.0065576)
7. Beimforde C, Feldberg K, Nylinder S, Rikkinen J, Tuovila H, Dörfelt H, Gube M, Jackson DJ, Reitner J, Seyfullah LJ, Schmidt, AR. 2014 Estimating the Phanerozoic history of the Ascomycota lineages: Combining fossil and molecular data. Mol Phylogenet Evol 78, 386–398. (https://doi.org/10.1016/j.ympev.2014.04.024)
8. Heininger C, Spribille T. 2009 The sorediate species of Xylographa in Austria (Baeomycetales, lichenized Ascomycetes). Herzogia 22, 129–134.
9. Katoh K, Standley DM. 2013 MAFFT multiple sequence alignment software version 7: improvements in performance and usability. Mol Biol Evol 30, 772–780. (doi:10.1093/molbev/mst010)
10. Resl P. 2015 phylo-scripts: Python scripts for phylogenetics. (doi:10.5281/zenodo.15983)
11. Bouckaert R, Heled, J, Kühnert D, Vaughan T, Wu C-H, Xie D, Suchard MA, Rambaut A, Drummond AJ. 2014 BEAST 2: A software platform for bayesian evolutionary analysis. PLoS Comput Biol 10, e1003537–6. (https://doi.org/10.1371/journal.pcbi.1003537)
12. Posada D 2008 jModelTest: phylogenetic model averaging. Mol Biol and Evol 25 (7): 1253–1256 (cit. on p. 6). (doi: 10.1093/molbev/msn083)
13. Amo de Paz G, Cubas P, Divakar PK, Lumbsch HT, Crespo A. 2011 Origin and diversification of major clades in parmelioid lichens (Parmeliaceae, Ascomycota) during the Paleogene inferred by Bayesian analysis. PLoS ONE 6, e28161–13 (https://doi.org/10.1371/journal.pone.0028161)
14. Rambaut A, Suchard MA, Xie D, Drummond AJ. 2014 "Tracer 1.6." Available from http://beast.bio.ed.ac.uk/Tracer
15. Santesson R, Moberg R, Nordin A, Tønsberg T, Vitikainen O. 2004 Lichen-forming and lichenicolous fungi of Fennoscandia. Museum of Evolution, Uppsala University
16. Smith CW, Aptroot A, Coppins BJ, Fletcher A, Gilbert OL, James PW, Wolseley PA. eds. 2009 The Lichens of Great Britain and Ireland. London: The British Lichen Society
17. Wirth V, Hauck M, Schultz M. 2013 Die Flechten Deutschlands. Stuttgart: Ulmer
18. Galloway DJ. 2013 The lichen genera Aspiciliopsis, and Placopsis (Trapeliales: Trapeliaceae: Ascomycota) in New Zealand. Phytotaxa 120, 1–194. (http://dx.doi.org/10.11646/phytotaxa.120.1.1)

19. Pagel M. 1999a Inferring the historical patterns of biological evolution. Nature 401, 877–884. (doi: 10.1038/44766)
20. Yang Z. 2006 Computational Molecular Evolution. Oxford: University Press.
21. Pennell MW, Eastman JM, Slater GJ, Brown JW, Uyeda JC, FitzJohn RG, Alfaro ME, Harmon LJ. 2014 geiger v2.0: an expanded suite of methods for fitting macroevolutionary models to phylogenetic trees. Bioinformatics 30, 2216–2218. (doi: 10.1093/bioinformatics/btu181)
22. Burns JH, Strauss SY. 2011 More closely related species are more ecologically similar in an experimental test. P Natl Acad Sci USA 108, 5302–5307. (doi: 10.1073/pnas.1013003108)
23. Münkemüller T, Lavergne S, Bzeznik B, Dray S, Jombart T, Schiffers K, Thuiller W. 2012 How to measure and test phylogenetic signal. Methods Ecol Evol 3, 743–756. (http://doi.org/10.1111/j.2041-210X.2012.00196.x)
24. Paradis E, Claude J, Strimmer K. 2004 APE: analyses of phylogenetics and evolution in R language. Bioinformatics 20, 289–290. (DOI: 10.1093/bioinformatics/btg412)
25. Ekman S, Andersen HL, Wedin M. 2008 The limitations of ancestral state reconstruction and the evolution of the ascus in the Lecanorales (lichenized Ascomycota). Syst Biol 57, 141–156. (http://doi.org/10.1080/10635150801910451)
26. Pagel M. 1999b The maximum likelihood approach to reconstructing ancestral character states of discrete characters on phylogenies. Syst Biol 48, 612–622. (https://doi.org/10.1080/106351599260184)
27. Pupko T, Itsik P, Shamir R, Graur D. 2000 A fast algorithm for joint reconstruction of ancestral amino acid sequences. Mol Biol Evol 17, 890–896. (https://doi.org/10.1093/oxfordjournals.molbev.a026369)
28. Beaulieu JM, O'Meara BC, Donoghue, MJ. 2013 Identifying hidden rate changes in the evolution of a binary morphological character: the evolution of plant habit in campanulid angiosperms. Syst Biol 62, 725–737. (https://doi.org/10.1093/sysbio/syt034)
29. Maddison WP, Midford PE, Otto SP 2007 Estimating a Binary Character's Effect on Speciation and Extinction. Syst Biol 56, 701–710. (https://doi.org/10.1080/10635150701607033)
30. FitzJohn RG, Maddison WP, Otto SP. 2009 Estimating trait-dependent speciation and extinction rates from incompletely resolved phylogenies. Syst Biol 58, 595-611. (https://doi.org/10.1093/sysbio/syp067)
31. Huelsenbeck JP, Nielsen R, Bollback, JP. 2003 Stochastic mapping of morphological characters. Syst Biol 52, 131–158. (https://doi.org/10.1080/10635150390192780)
32. Revell LJ 2011 phytools: An R package for phylogenetic comparative biology (and other things). Methods Ecol and Evol 3, 217–223. (doi: 10.1111/j.2041-210X.2011.00169.x)
33. Rabosky DL 2014 Automatic detection of key innovations, rate shifts, and diversity-dependence on phylogenetic trees. PLoS ONE 9, e89543. (https://doi.org/10.1371/journal.pone.0089543)
34. Rabosky DL, Grundler M, Anderson C, Title P, Shi JJ, Brown JW, Huang H, Larson JG. 2014 BAMMtools: An R package for the analysis of evolutionary dynamics on phylogenetic trees. Methods Ecol Evol 5, 701–707. (doi: 10.1111/2041-210X.12199)
35. FitzJohn RG. 2012 Diversitree: comparative phylogenetic analyses of diversification in R. Methods Ecol Evol 3, 1084–1092. (DOI: 10.1111/j.2041-210X.2012.00234.x)
36. Rabosky DL, Goldberg EE. 2015 Model Inadequacy and Mistaken Inferences of Trait- Dependent Speciation. Syst Biol 64, 340–355. (https://doi.org/10.1093/sysbio/syu131)

Figure S1 - Relative sampling completeness

Sampling completeness of sampled taxa used in this study. (a) Relative number of species sampled and grouped by substrates. Numbers above bars indicate number of species sampled in our dataset and total number of known trapelioid species growing on a particular substrate. (b) Relative number of species sampled per genus. Numbers above bars indicate sampled number of species per genus and total number of species per genus according to different information sources.

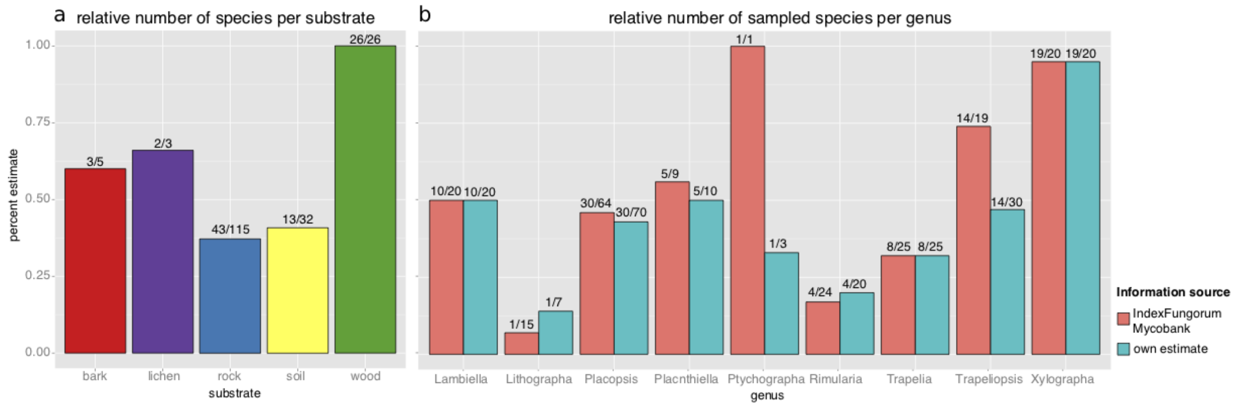

Figure S2 - Phylogenetic informativeness simulation 1: Pagel's Lambda

p-value distributions of phylogenetic signal analyses relying on Pagel's lambda: We transformed each of the 100 trees from the BEAST posterior tree distribution for eleven values of lambda and fitted models using tip distributions of the preferred substrate character on each. To obtain a null-model we simulated 100 random character distributions and fitted models for each simulated character on each tree. We then compared AIC scores of fitted models for the preferred substrate character set to the distribution of AIC scores obtained from random character sets for each tree with a Mann-Whitney test. p-values < 0.05 indicate significantly better fit (higher phylogenetic signal) for the real character distribution compared to simulated characters. y-axis = number of trees, x-axis = p-value

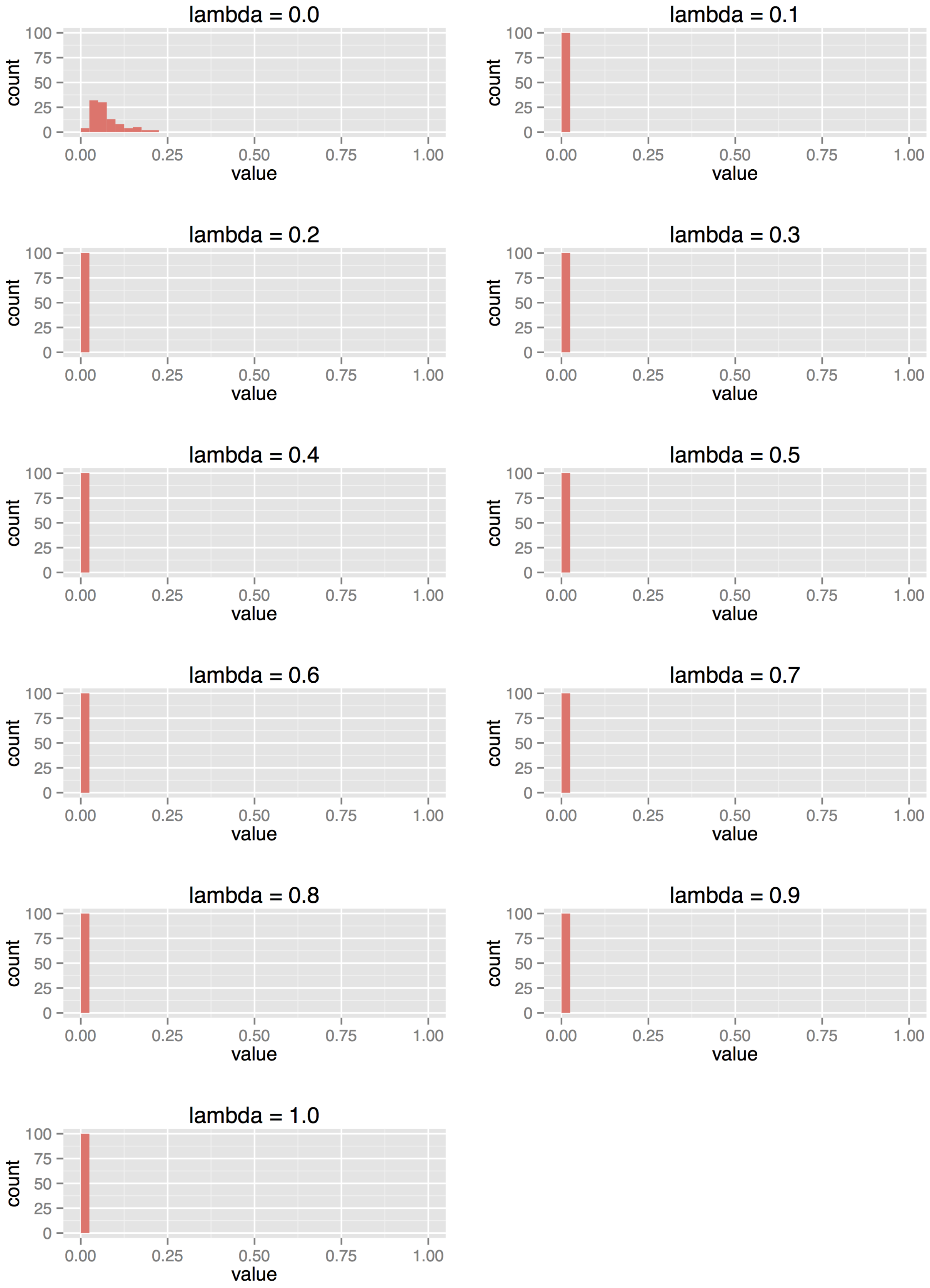
lambda = 0.0
lambda = 0.1
lambda = 0.2
lambda = 0.3
lambda = 0.4
lambda = 0.5
lambda = 0.6
lambda = 0.7
lambda = 0.8
lambda = 0.9
lambda = 1.0
count
0
25
50
75
100
value
0.00
0.25
0.50
0.75
1.00

Figure S3 - Phylogenetic informativeness simulation 2: Distance method

Results of phylogenetic signal test assuming that closely related species are ecologically more similar than distantly related species. Plots show p-value distributions of Mann-Whitney tests of differences between mean tip-to-tip distances of real character distribution and simulated character distributions for each tree. blue = rock, yellow= soil, green= wood, red = bark, purple= lichen.

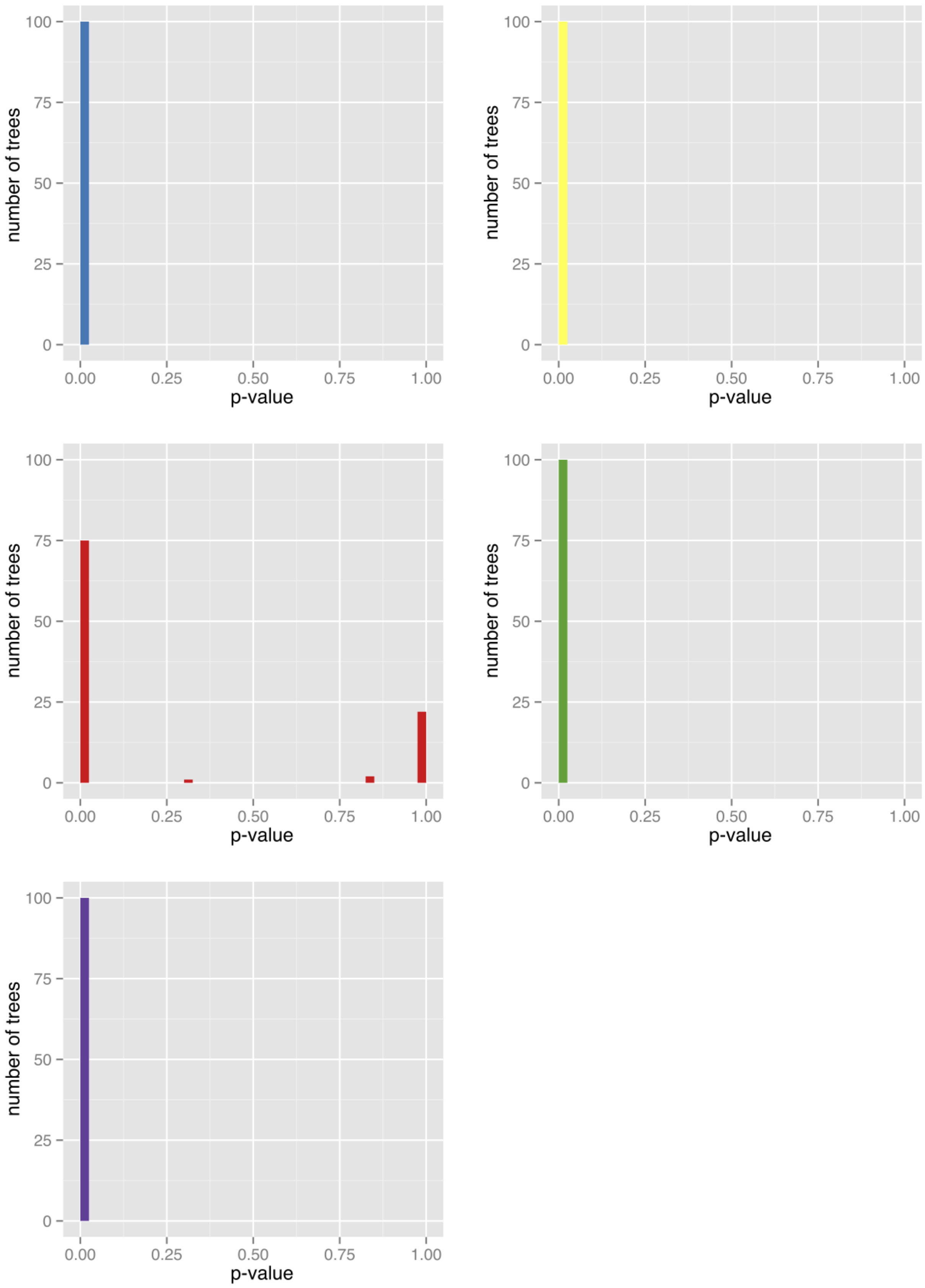
number of trees
100
75
50
25
0
0.00
0.25
0.50
0.75
1.00
p-value

Figure S4-S22 - Results of ancestral state estimation of the preferred substrate character with ace for 19 nodes of the trapelioid phylogeny

Ancestral state estimations of the preferred substrate character for 19 nodes of the trapelioid phylogeny based on the method implemented in the ace function in the R package ape imposing 6 different models. Please refer to the main text for details.

Figure S4: Ancestral states for node 1

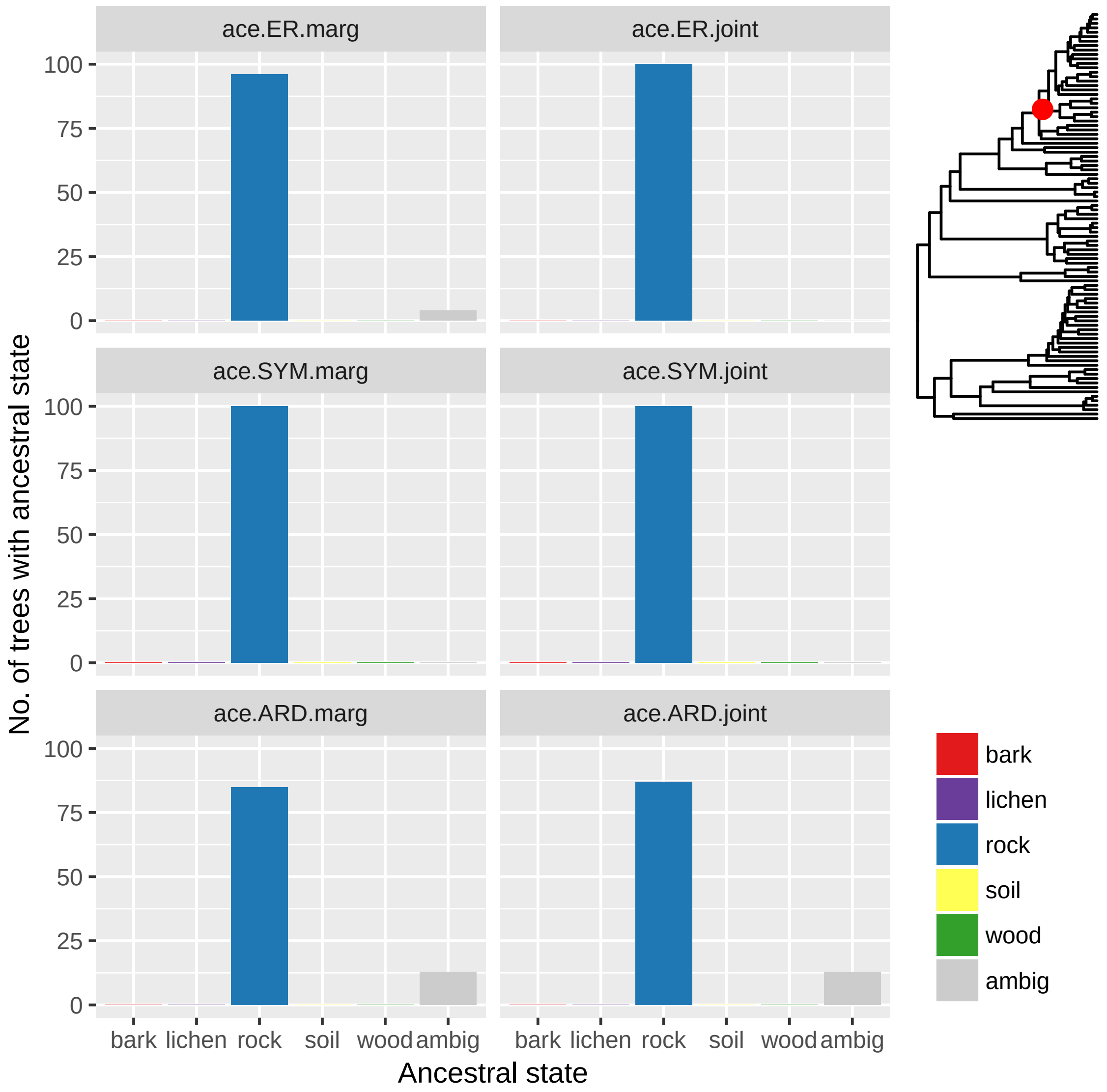

Figure S5: Ancestral states for node 2

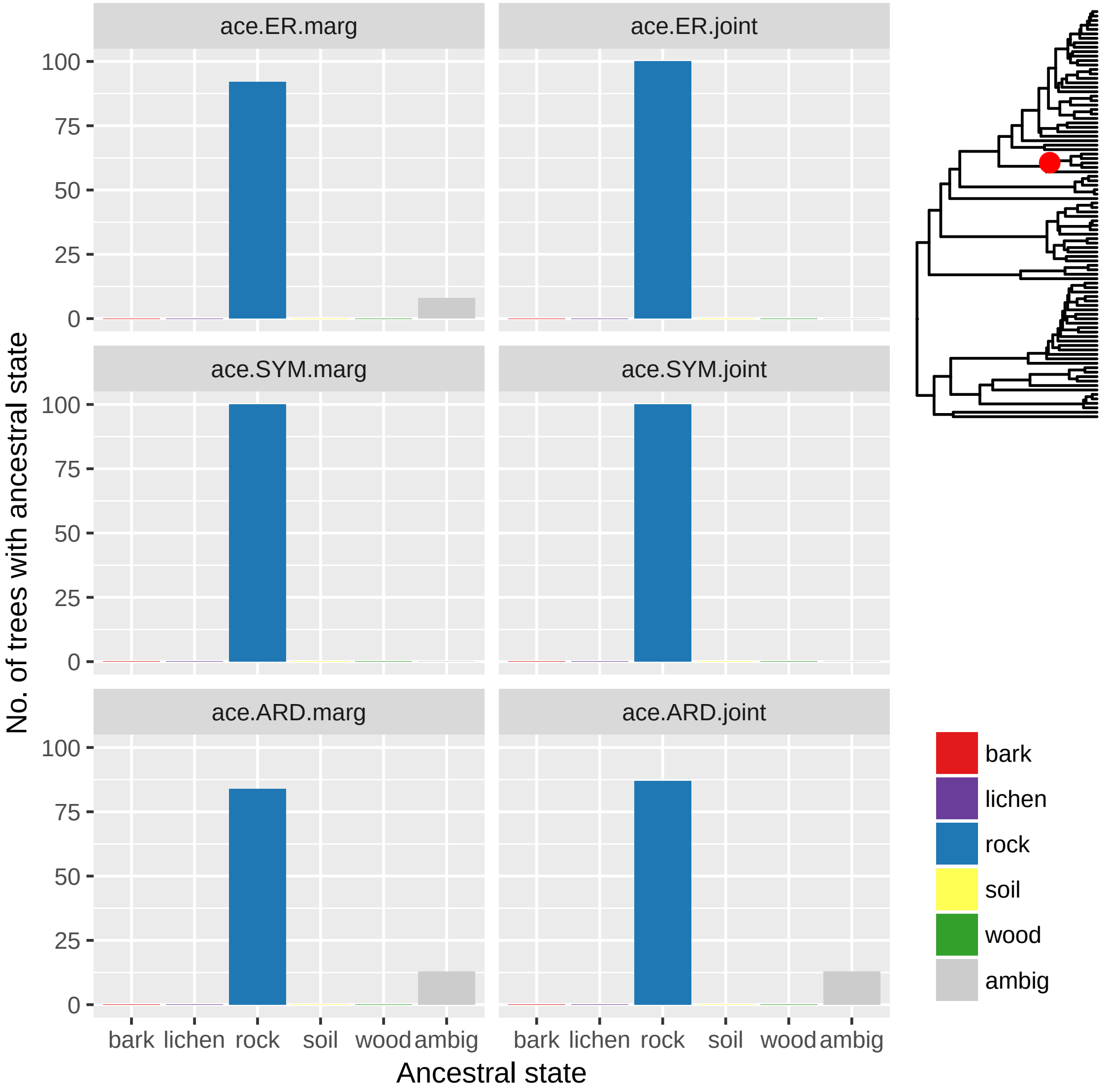

Figure S6: Ancestral states for node 3

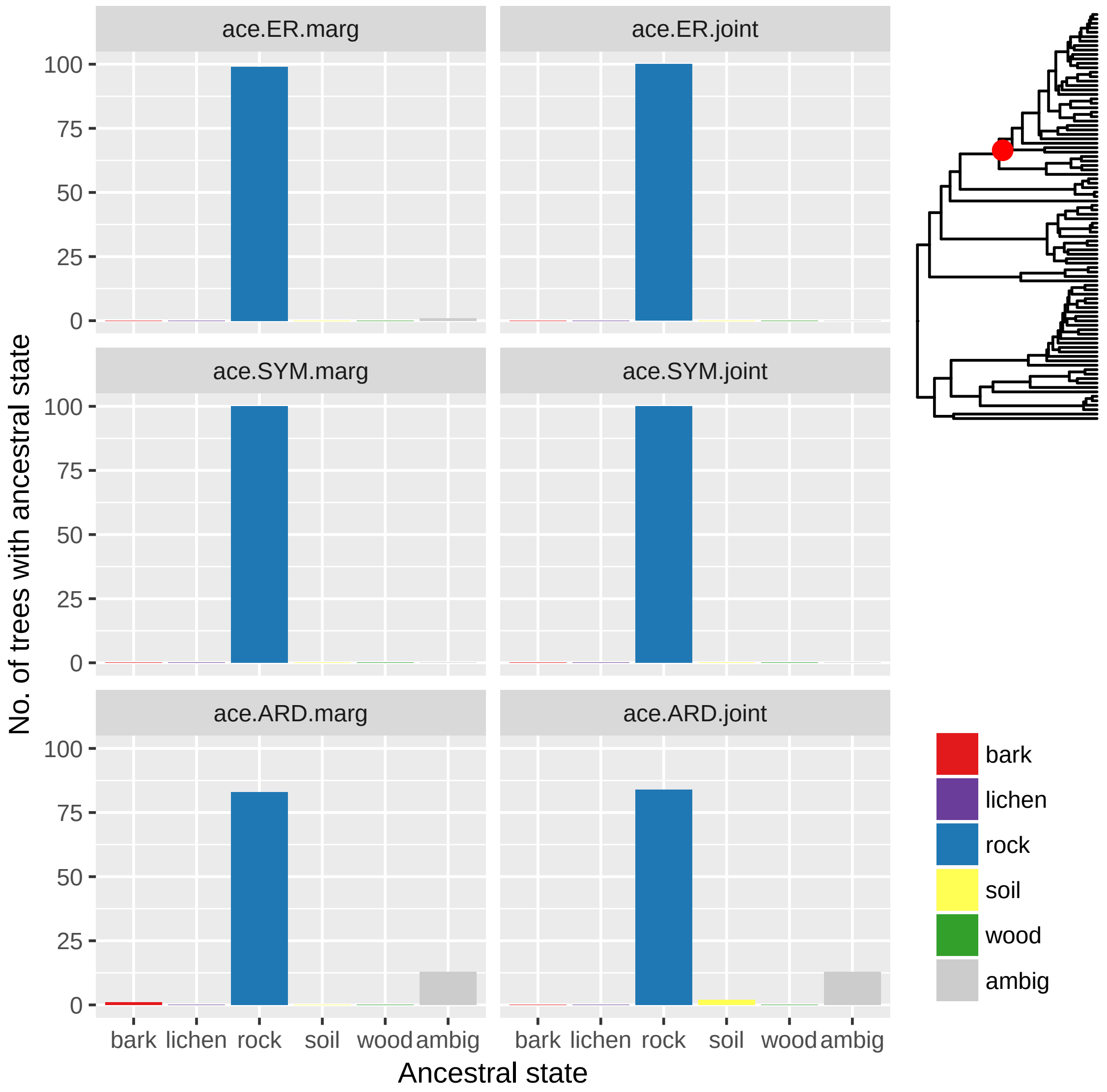

Figure S7: Ancestral states for node 4

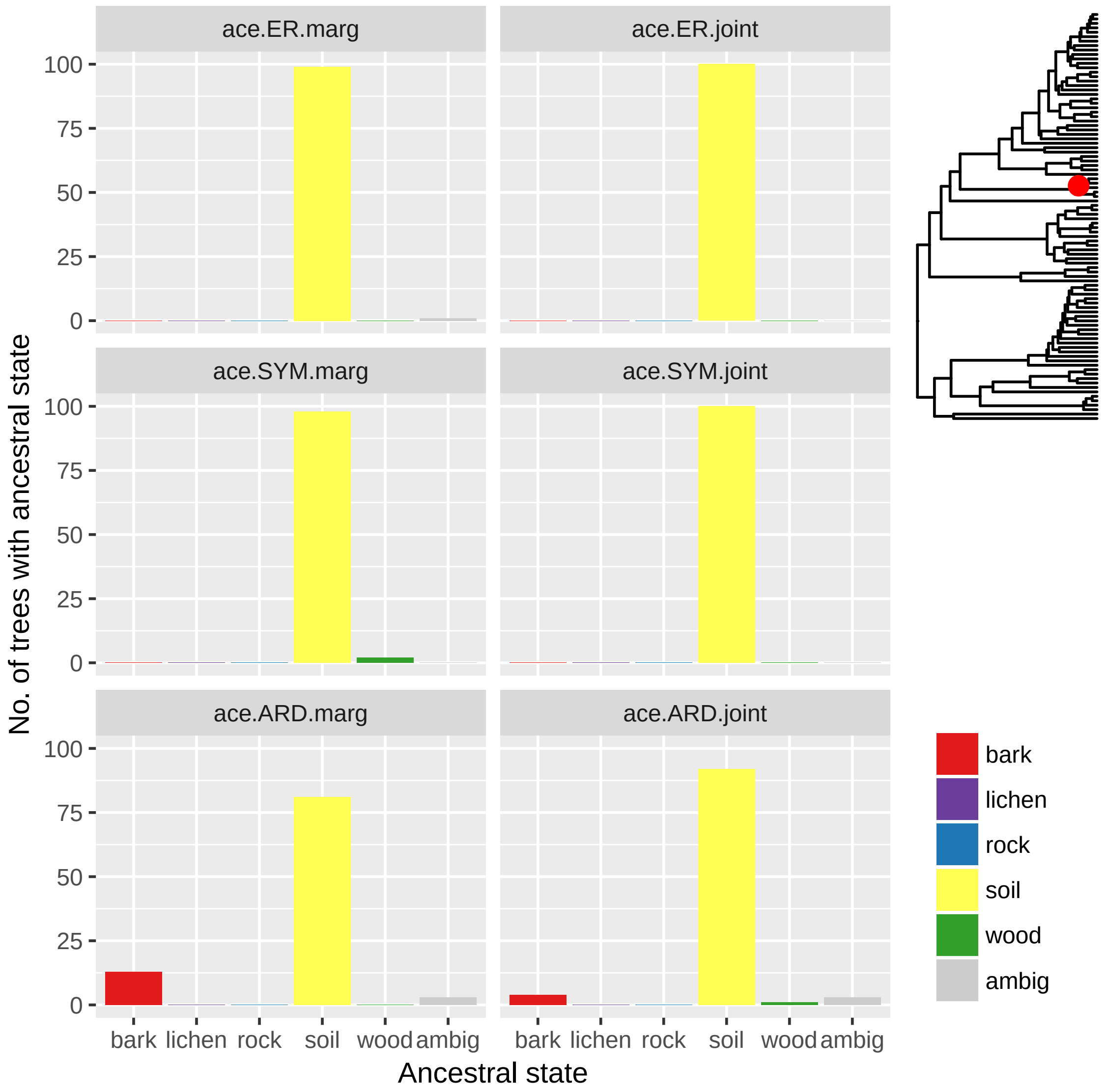

Figure S8: Ancestral states for node 5

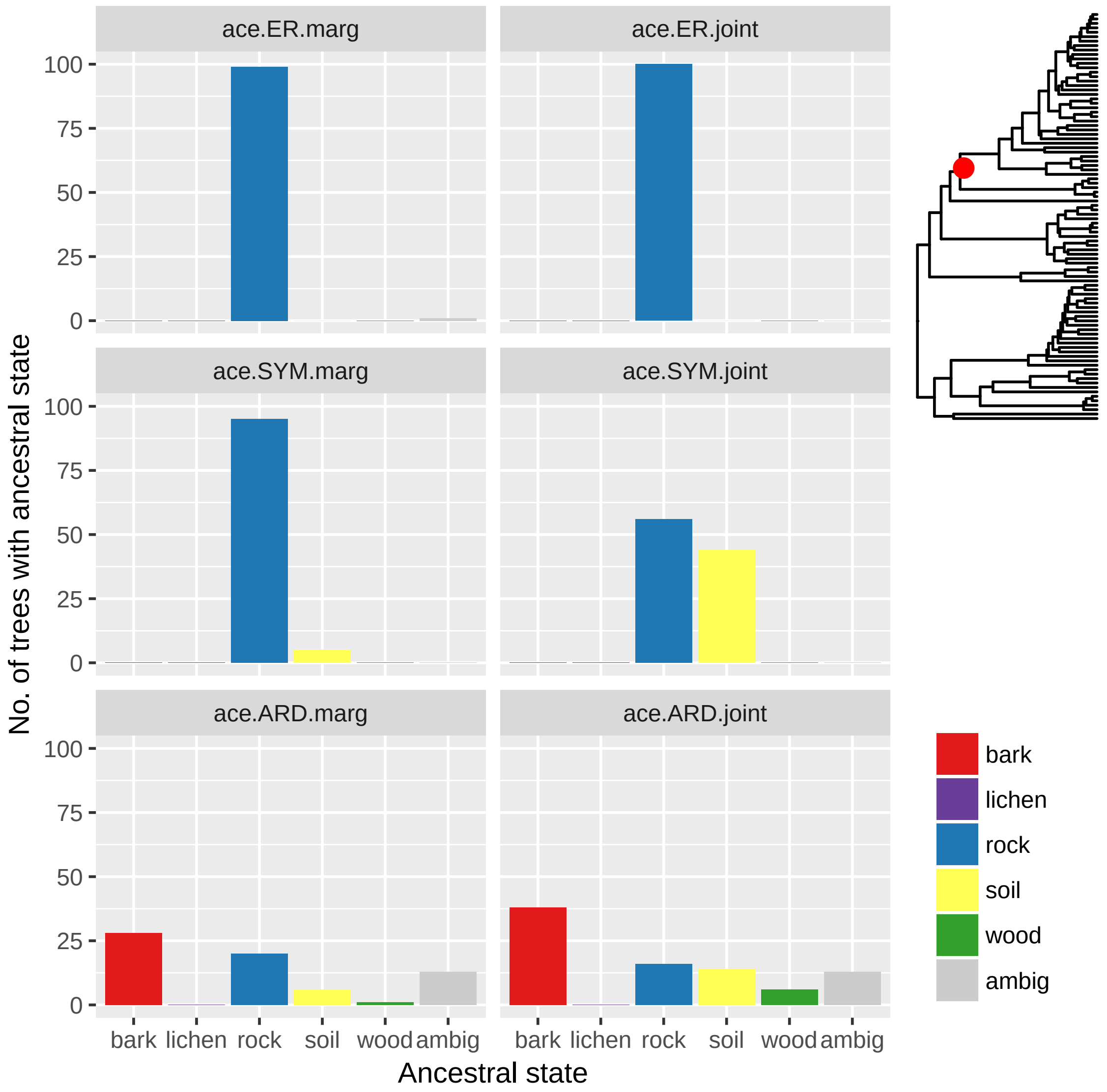

Figure S9: Ancestral states for node 6

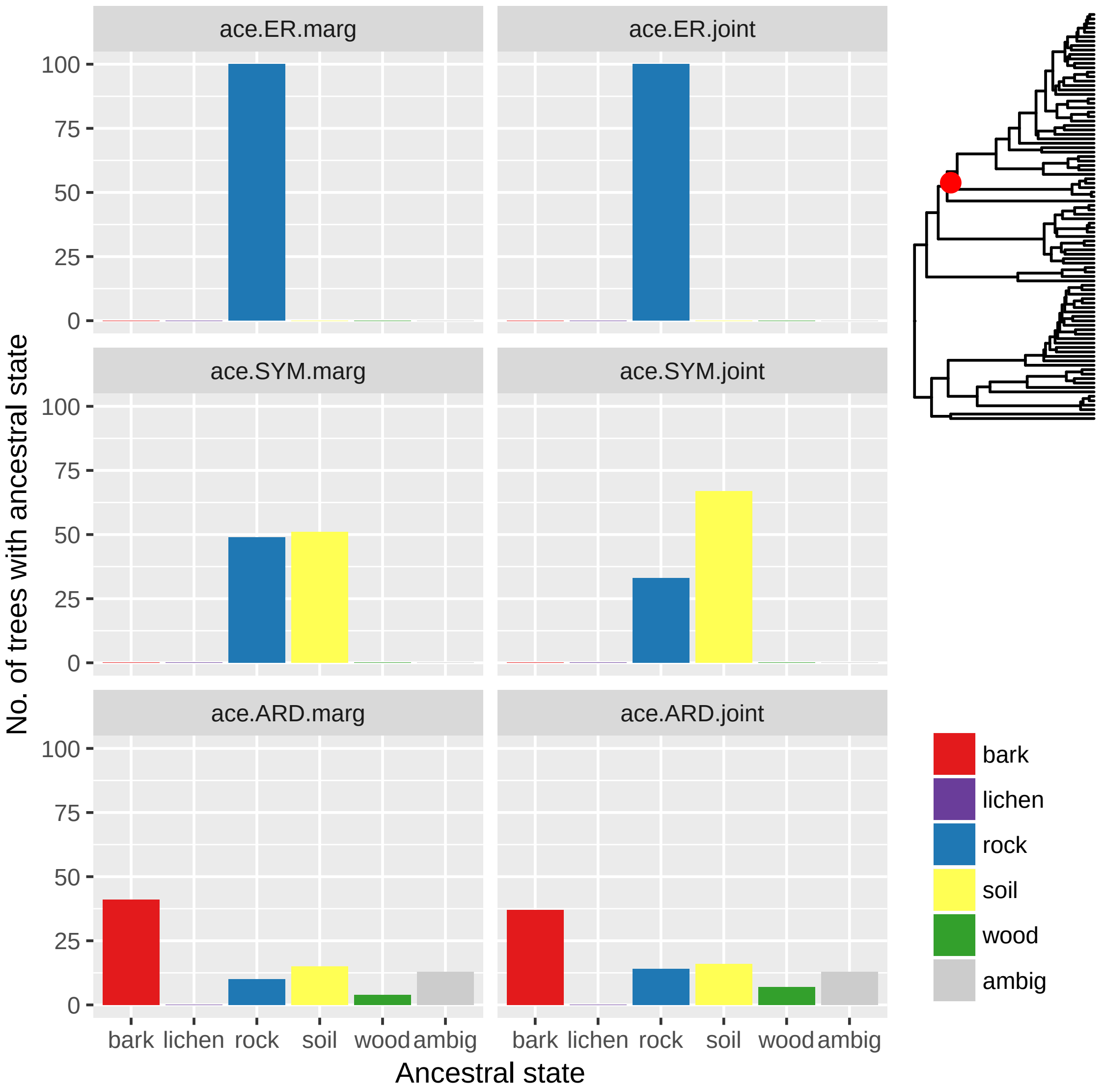

Figure S10: Ancestral states for node 7

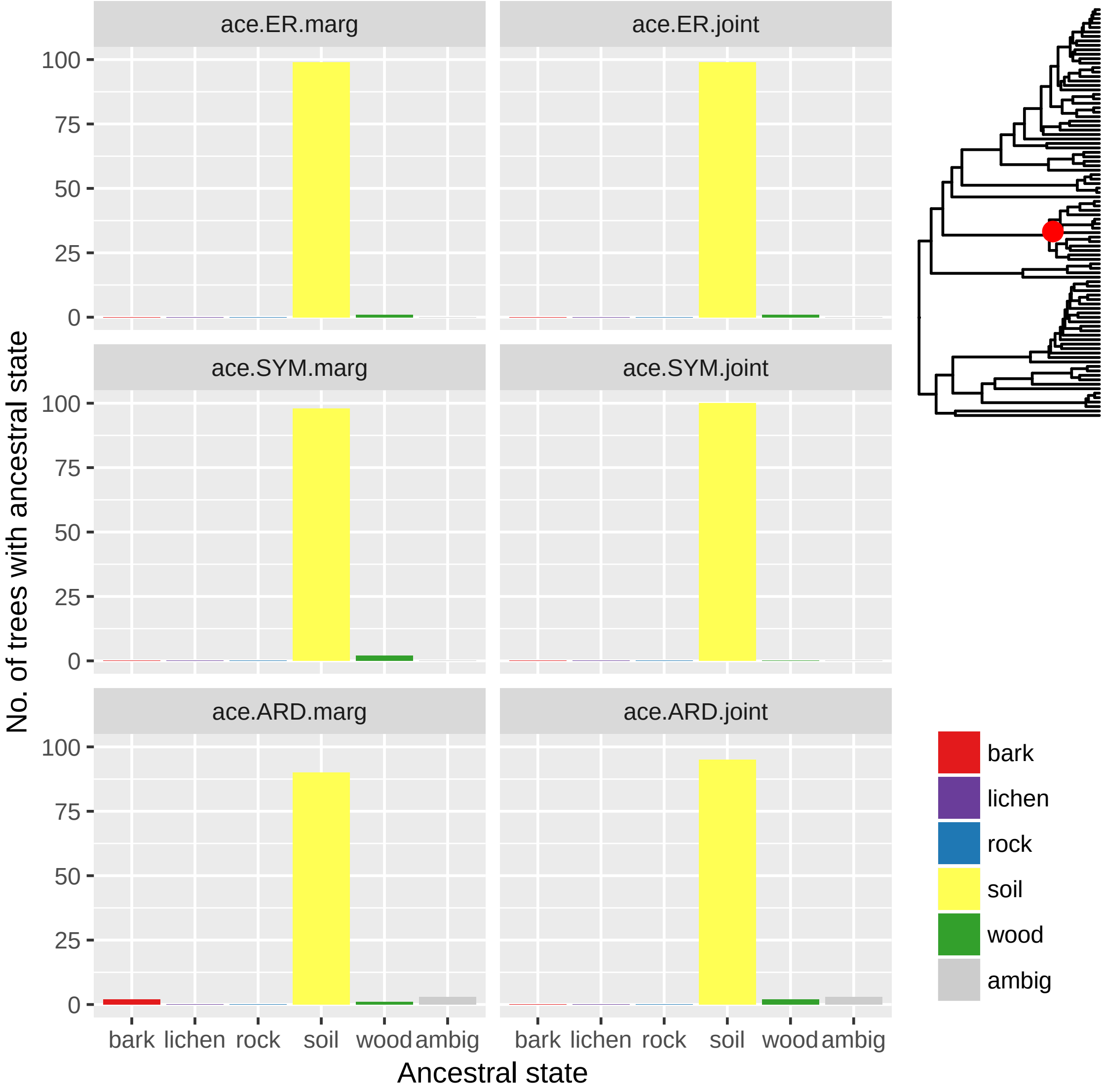

Figure S11: Ancestral states for node 8

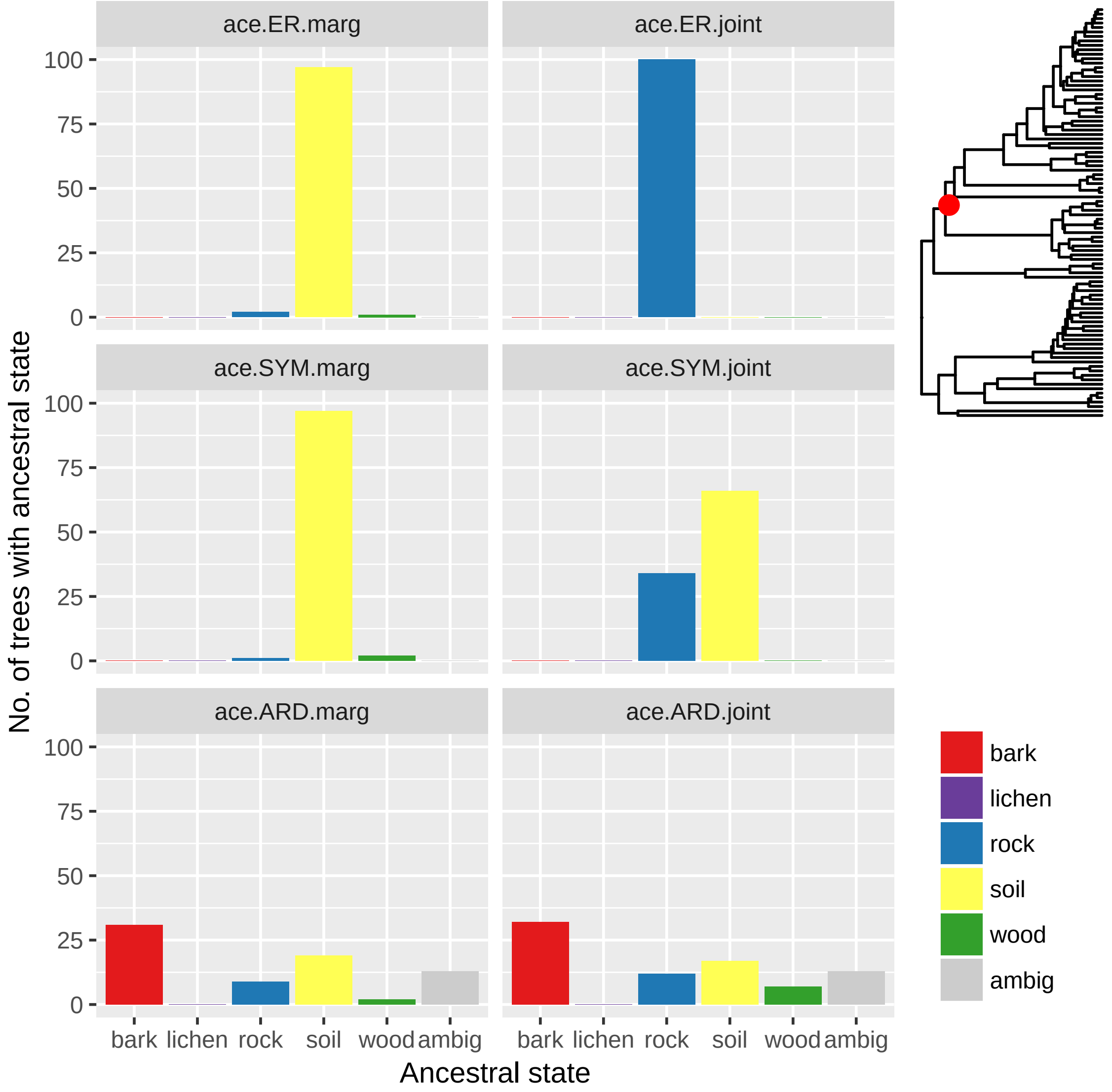

Figure S12: Ancestral states for node 9

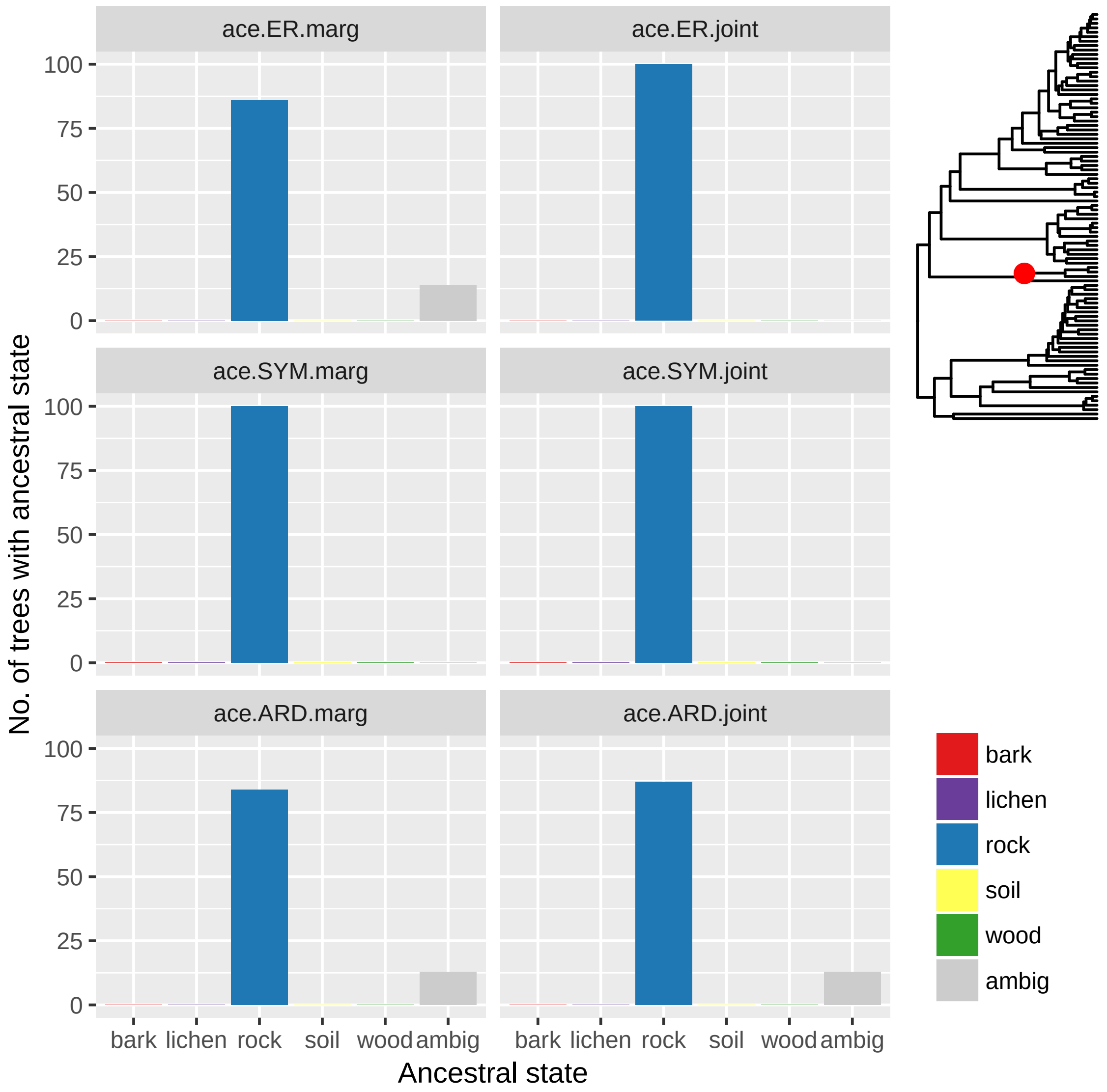

Figure S13: Ancestral states for node 10

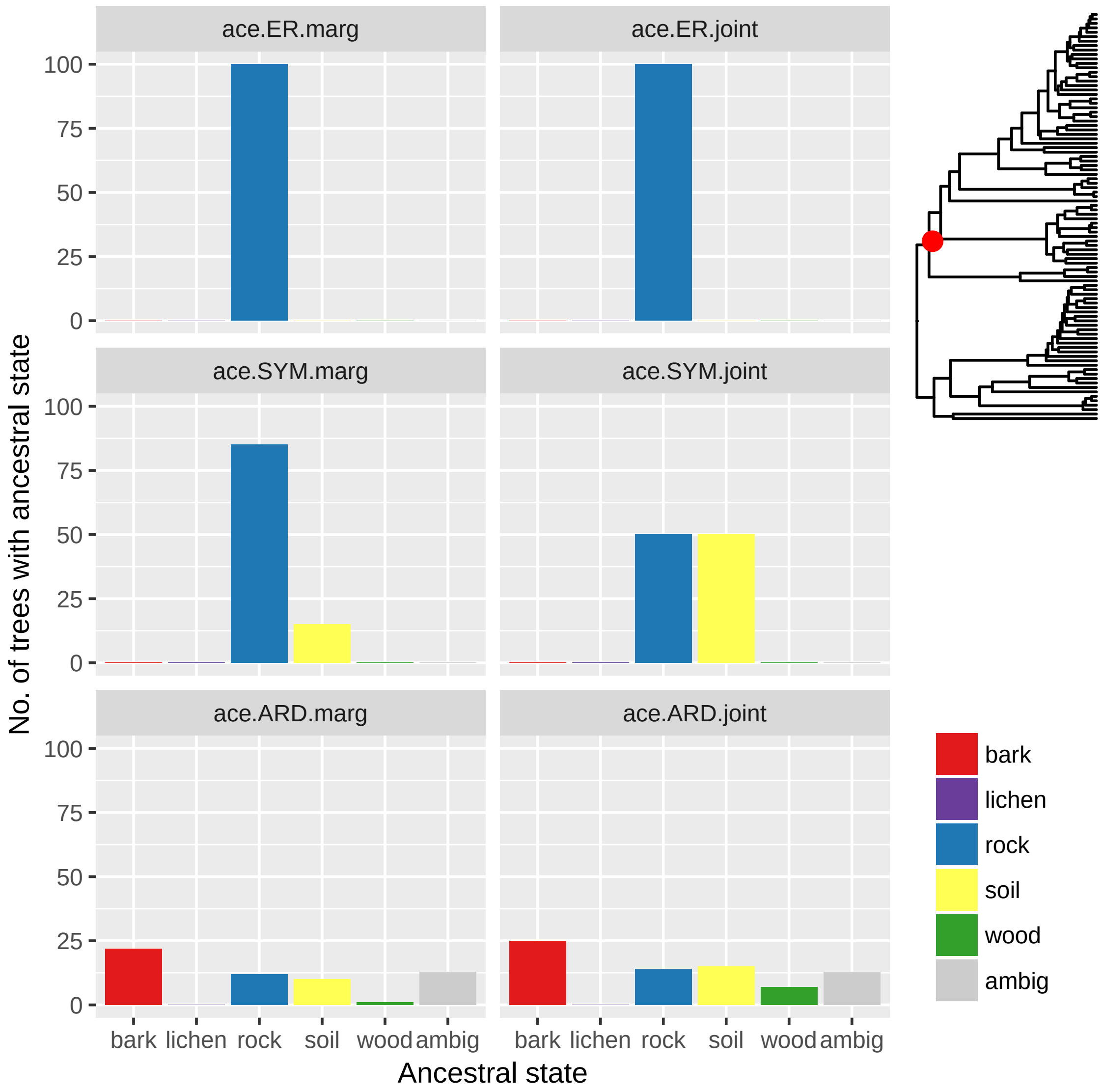

Figure S14: Ancestral states for node 11

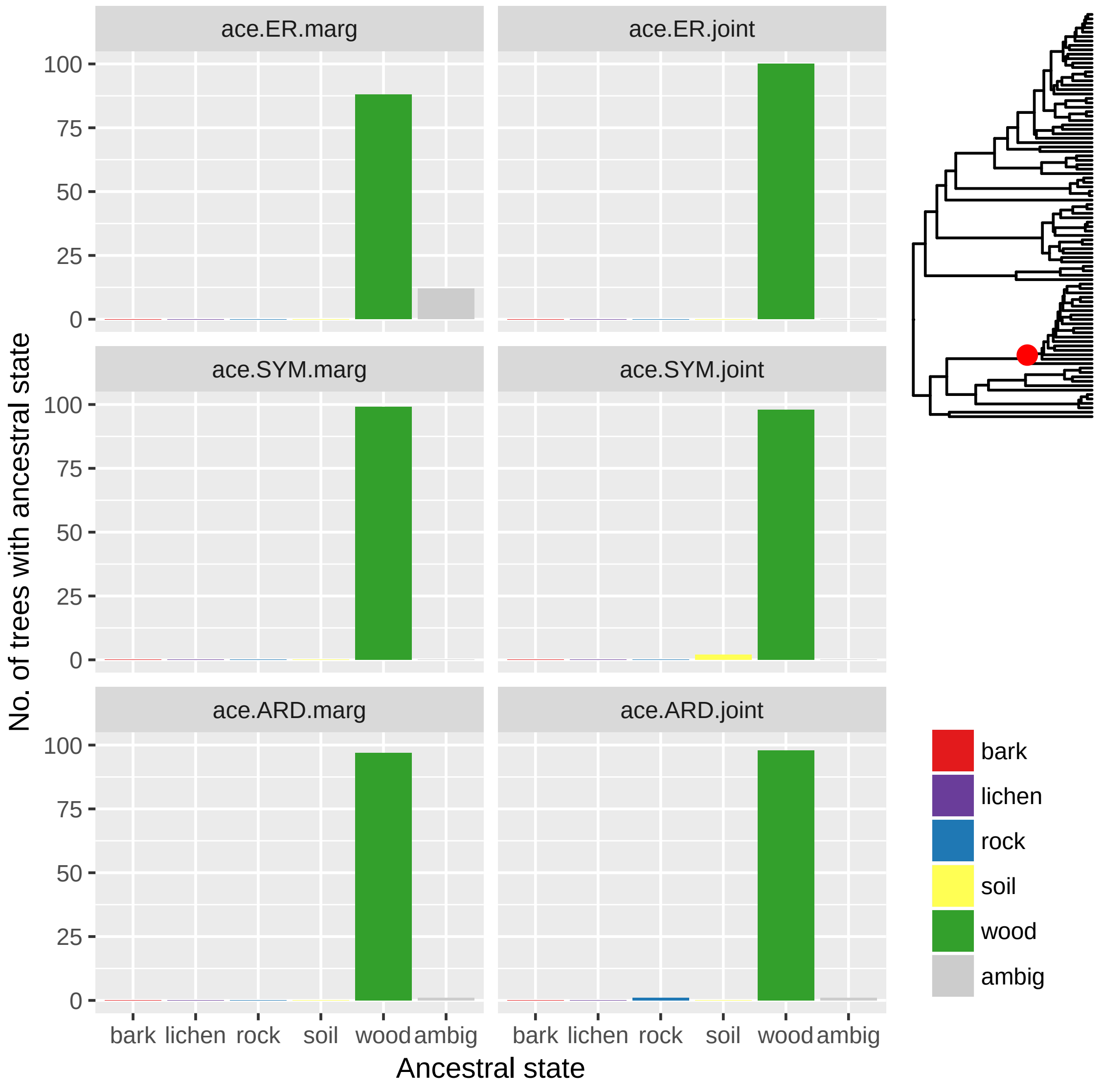

Figure S15: Ancestral states for node 12

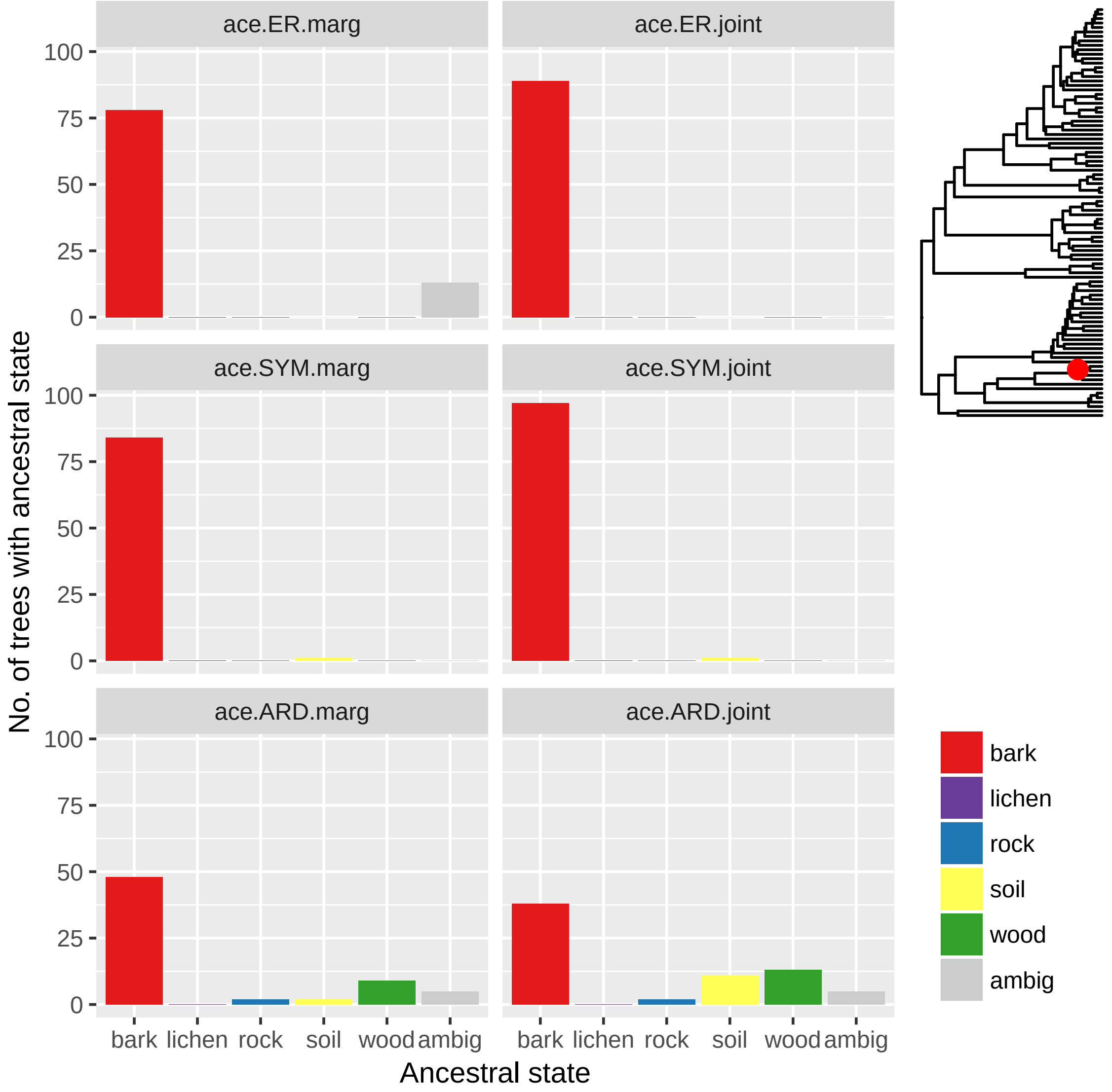

Figure S16: Ancestral states for node 13

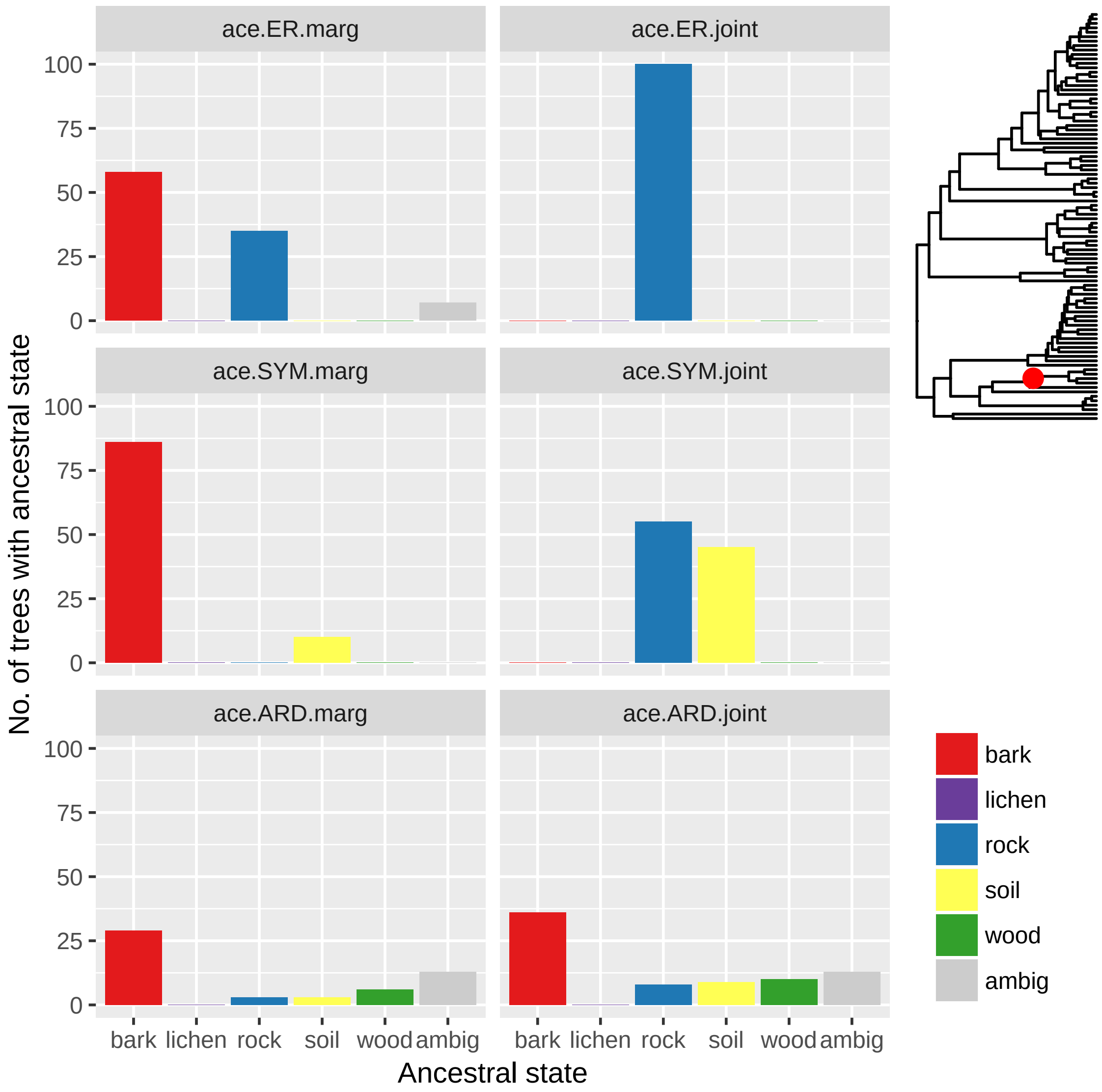

Figure S17: Ancestral states for node 14

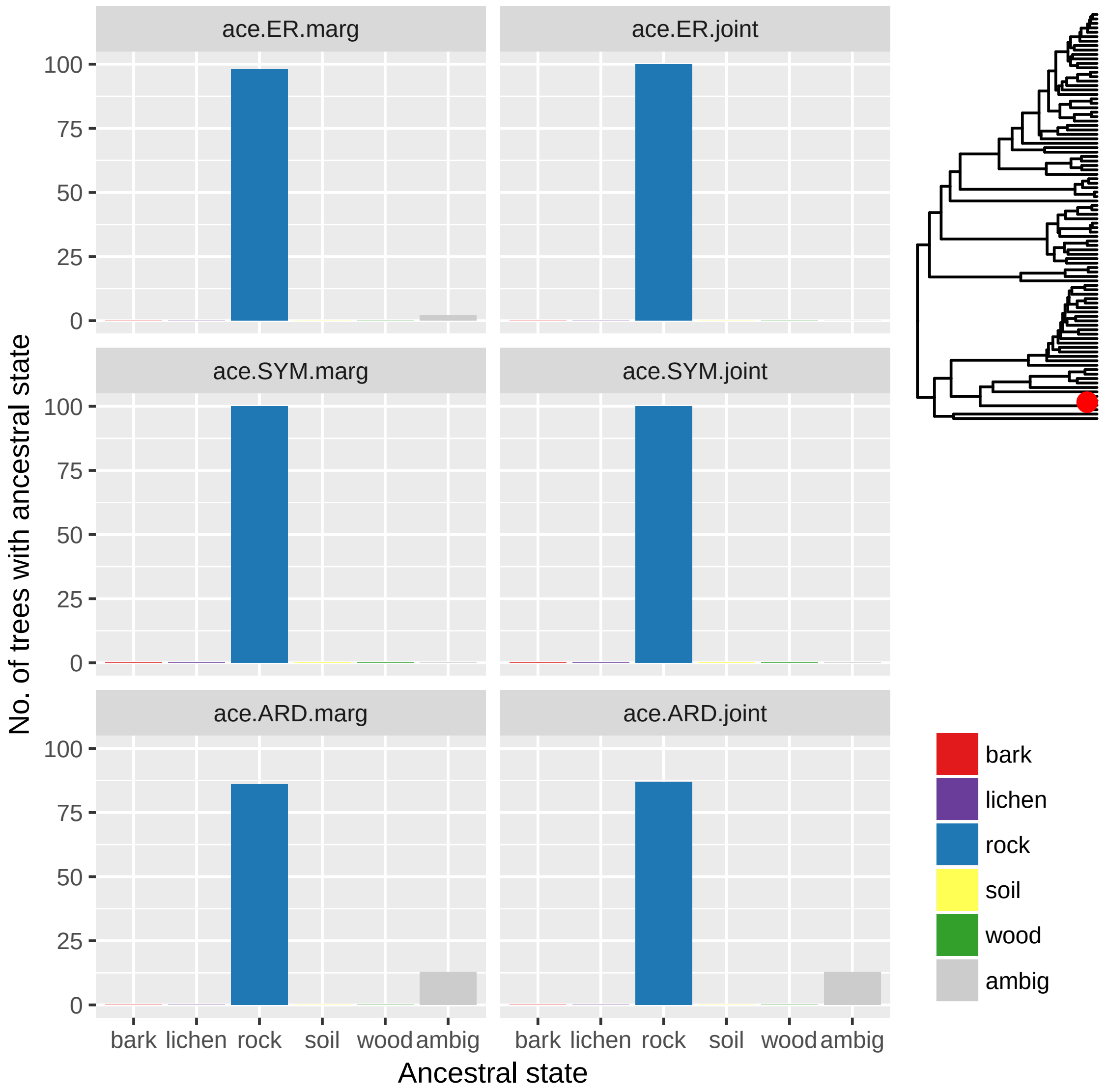

Figure S18: Ancestral states for node 15

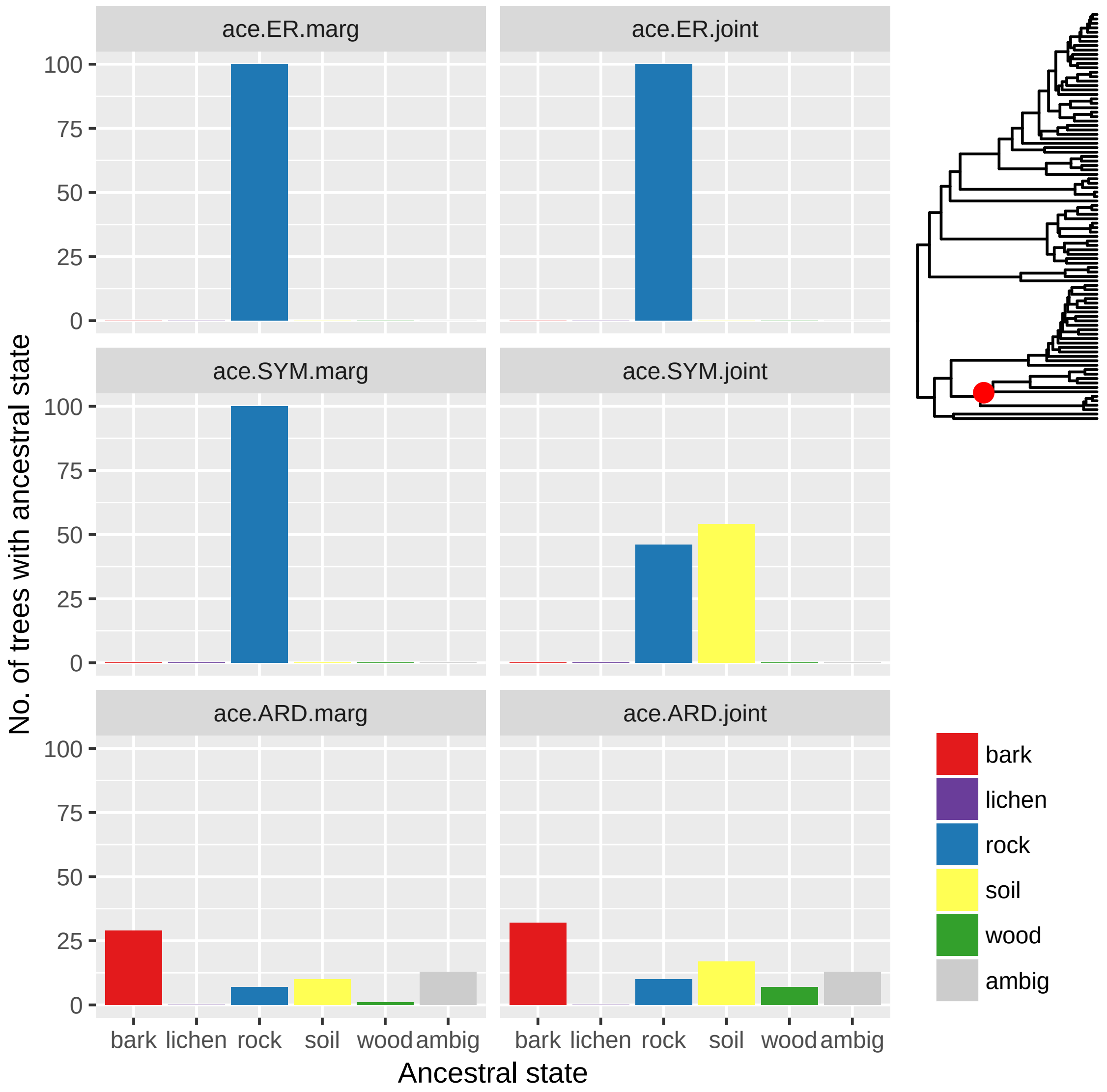

Figure S19: Ancestral states for node 16

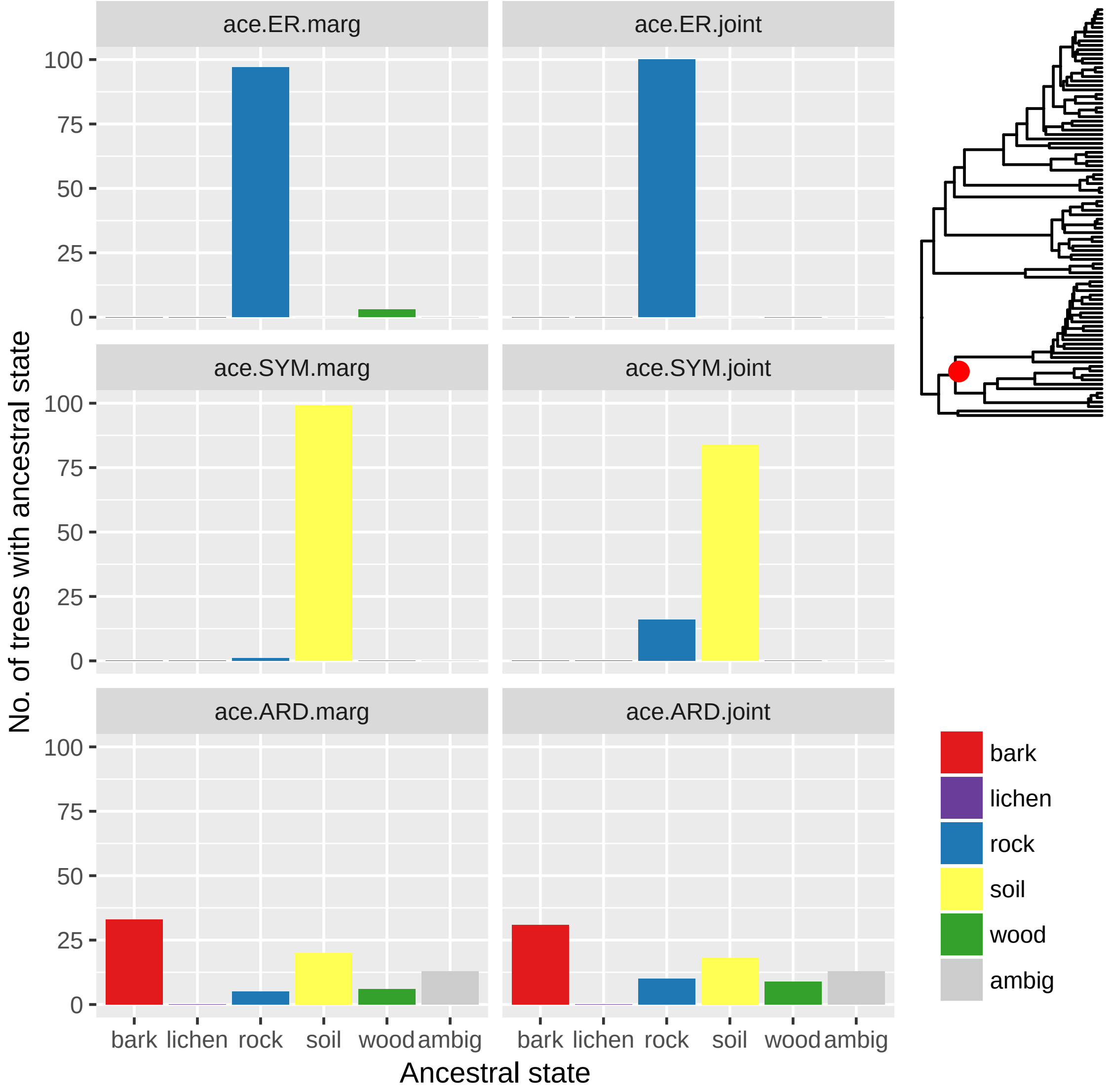

Figure S20: Ancestral states for node 17

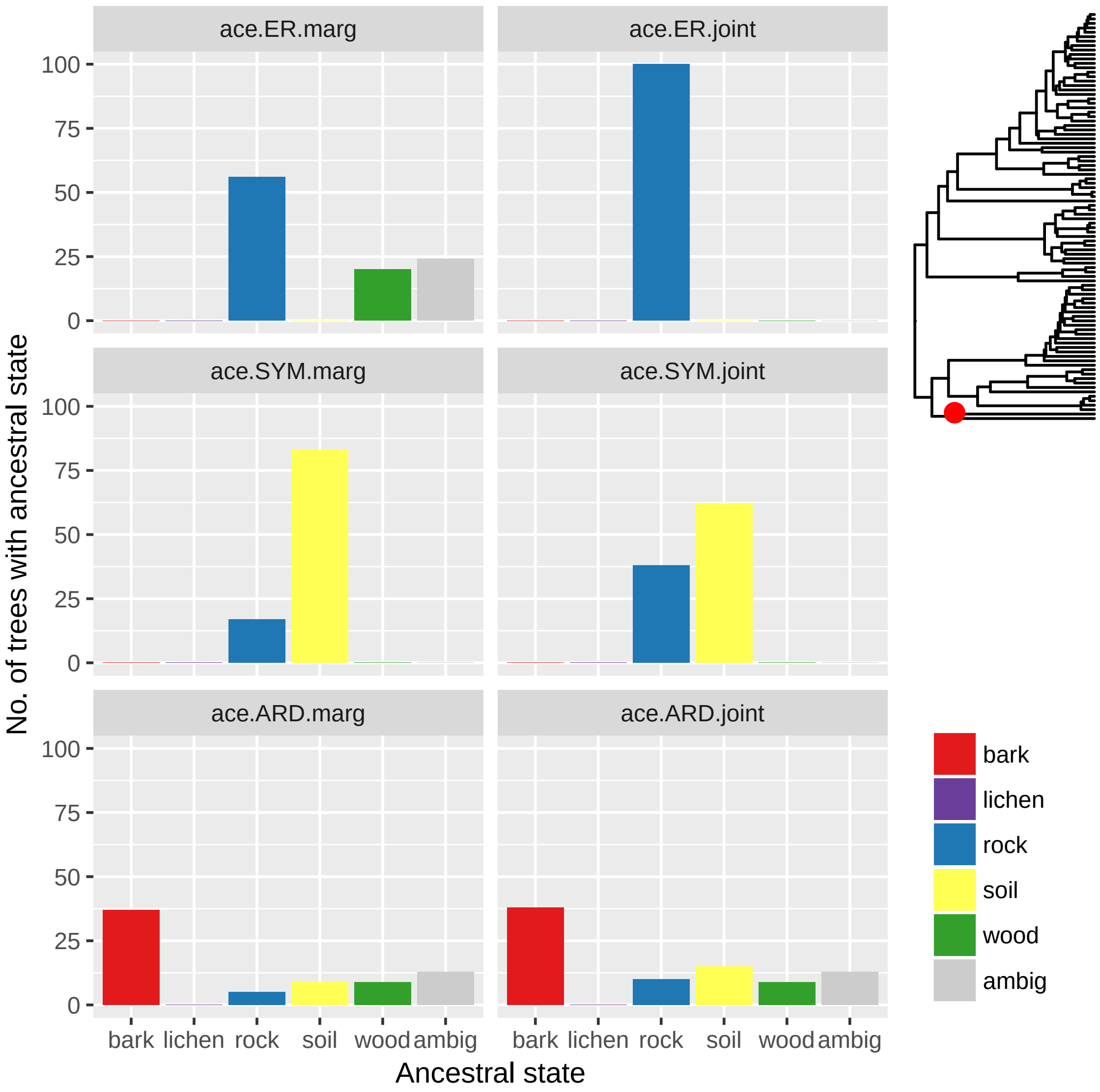

Figure S21: Ancestral states for node 18

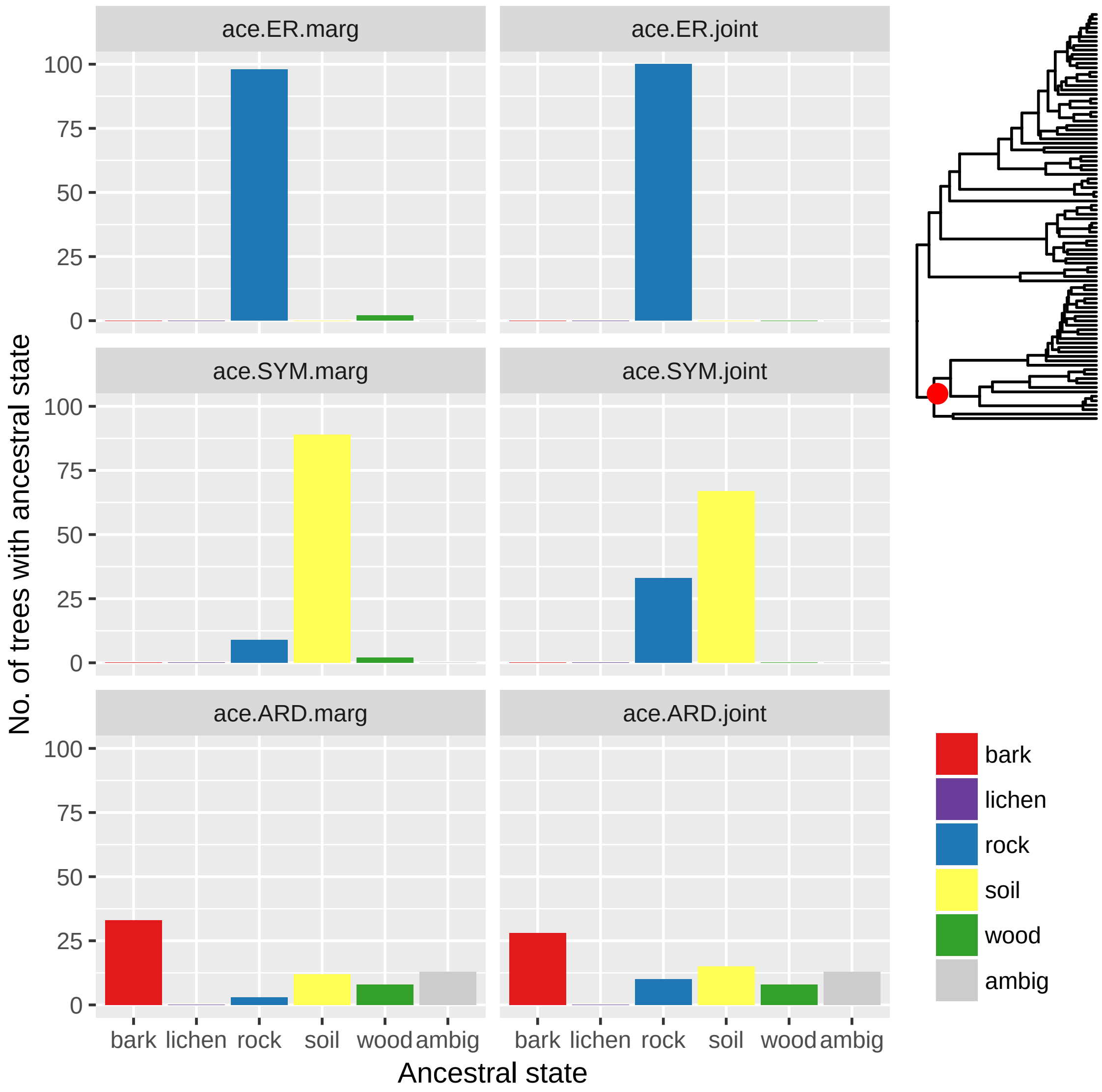

Figure S22: Ancestral states for node 19

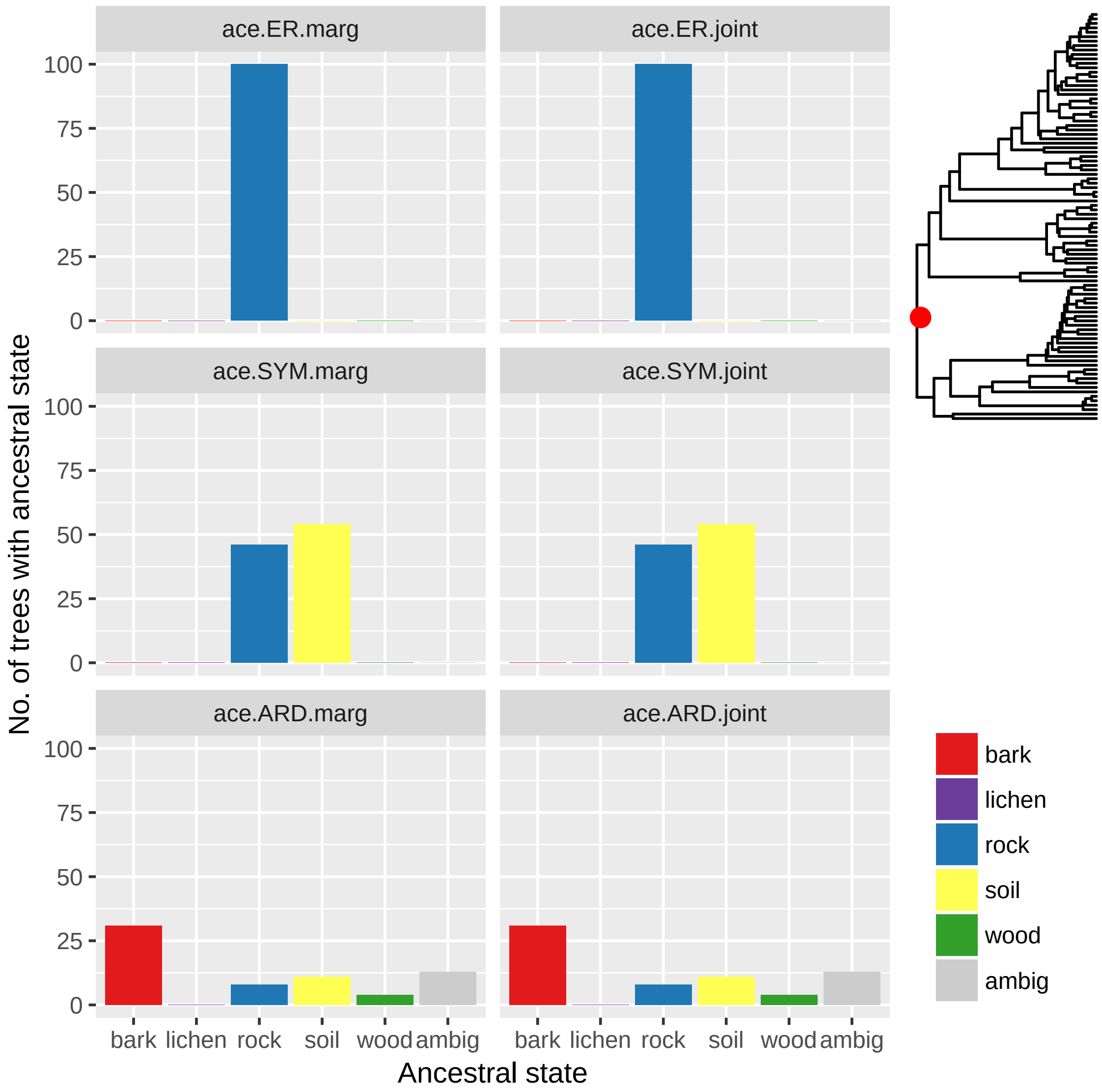

Figure S23-S41 - Results of ancestral state estimation of the preferred substrate character with rayDISC for 19 nodes of the trapelioid phylogeny

Ancestral state estimations of the preferred substrate character for 19 nodes of the trapelioid phylogeny based on the method implemented in rayDISC in the R package corHMM imposing 18 different models. Please refer to the main text for details.

Figure S23: Ancestral states for node 1

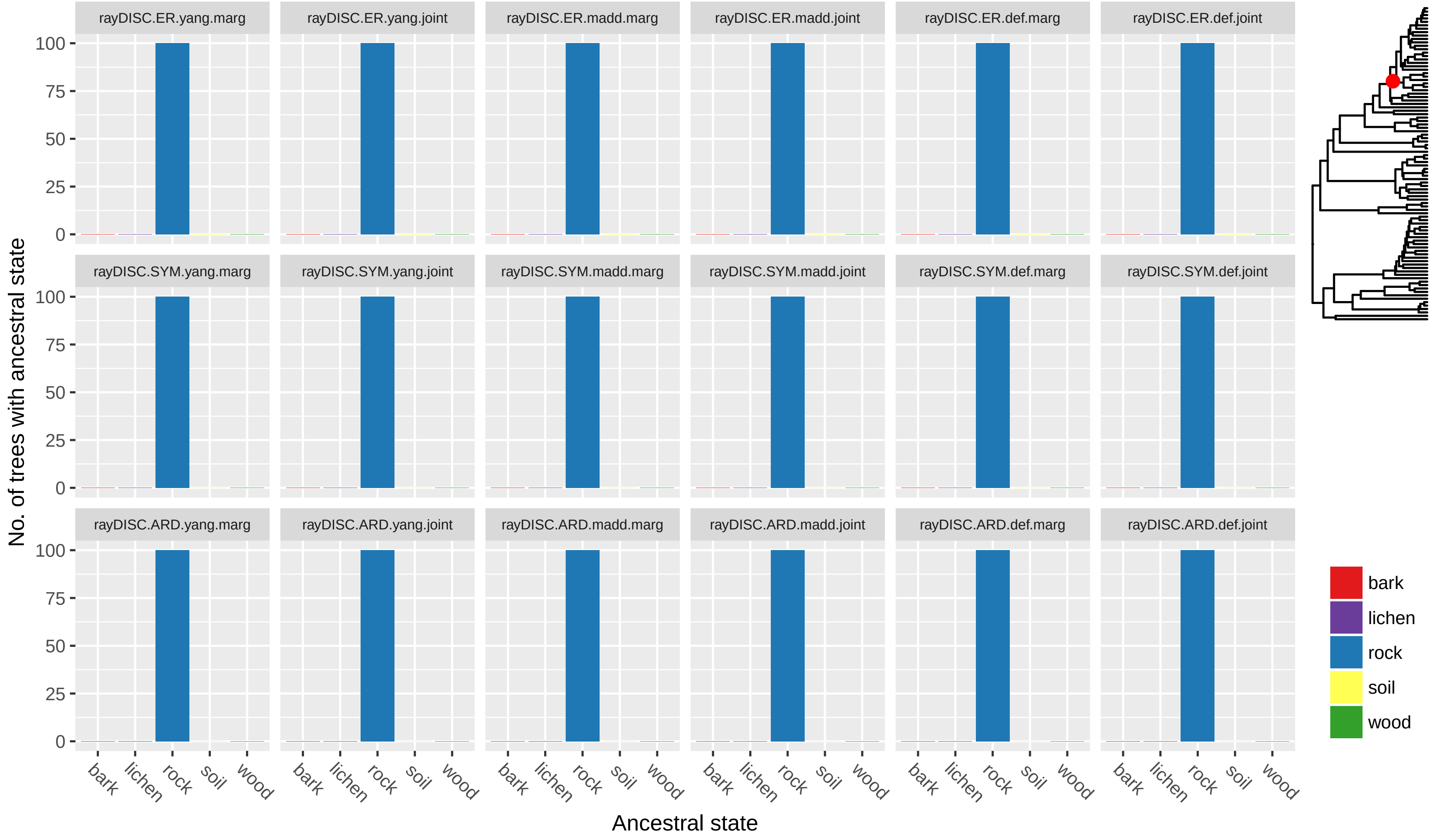

Figure S24: Ancestral states for node 2

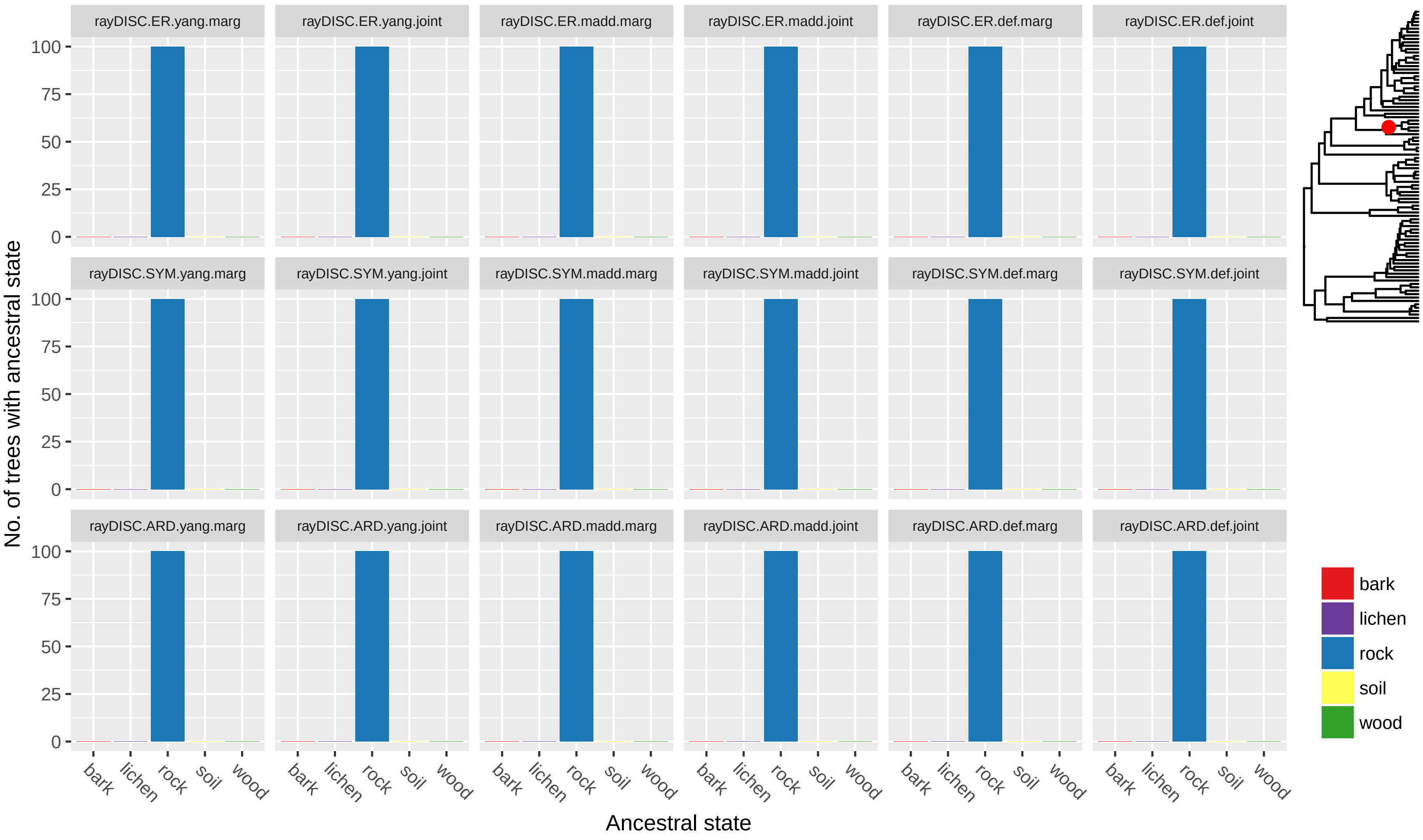

Figure S25: Ancestral states for node 3

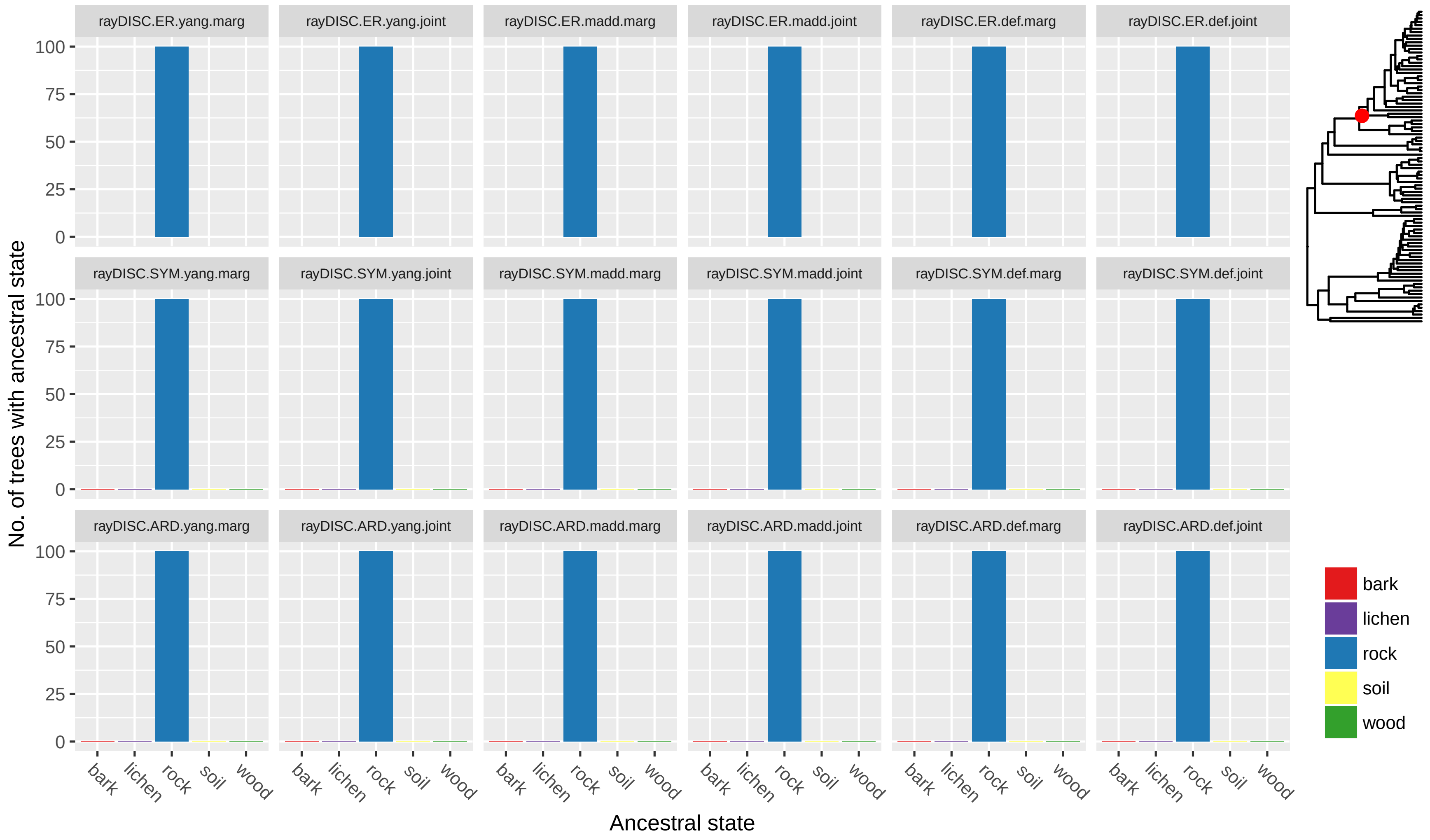

Figure S26: Ancestral states for node 4

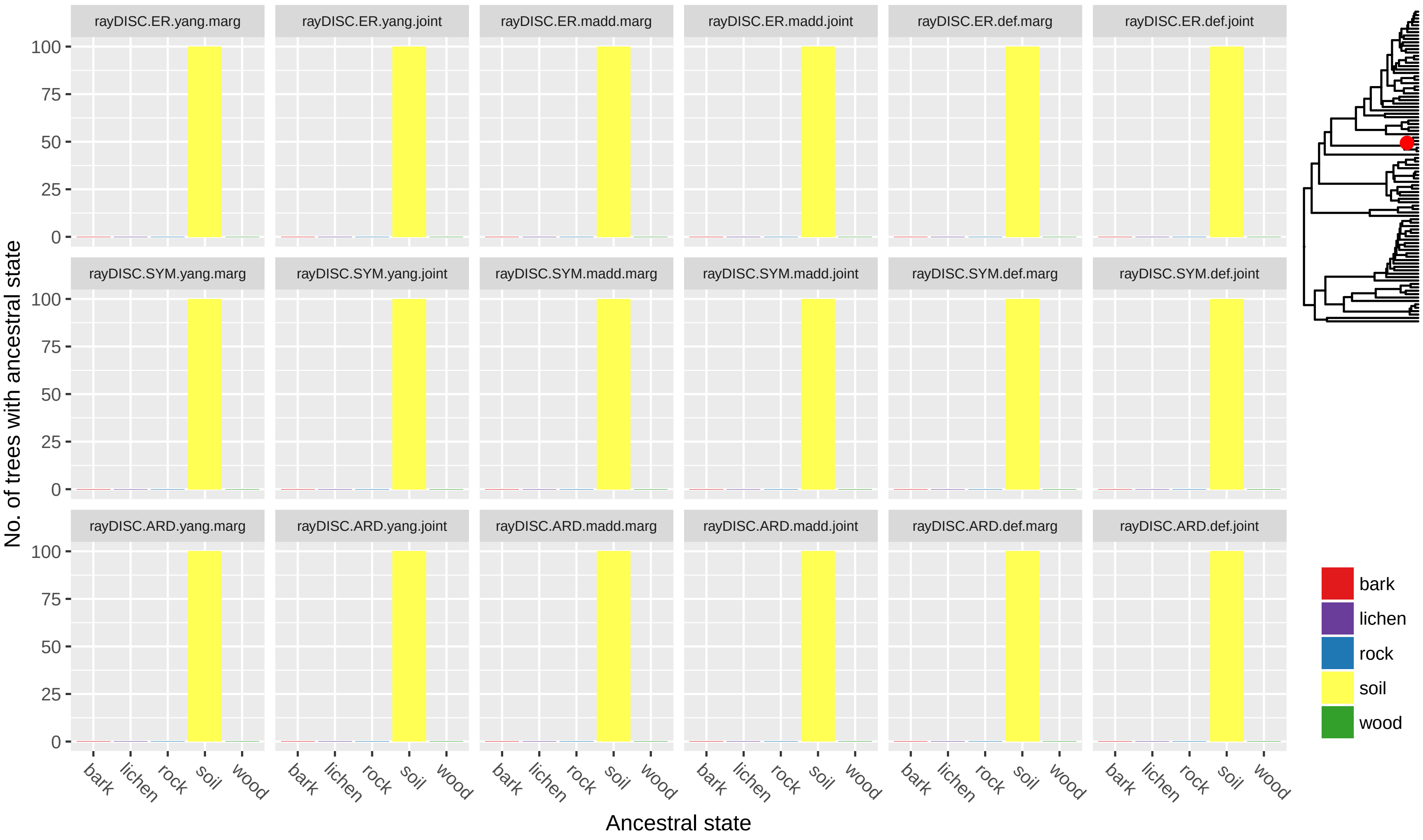

Figure S27: Ancestral states for node 5

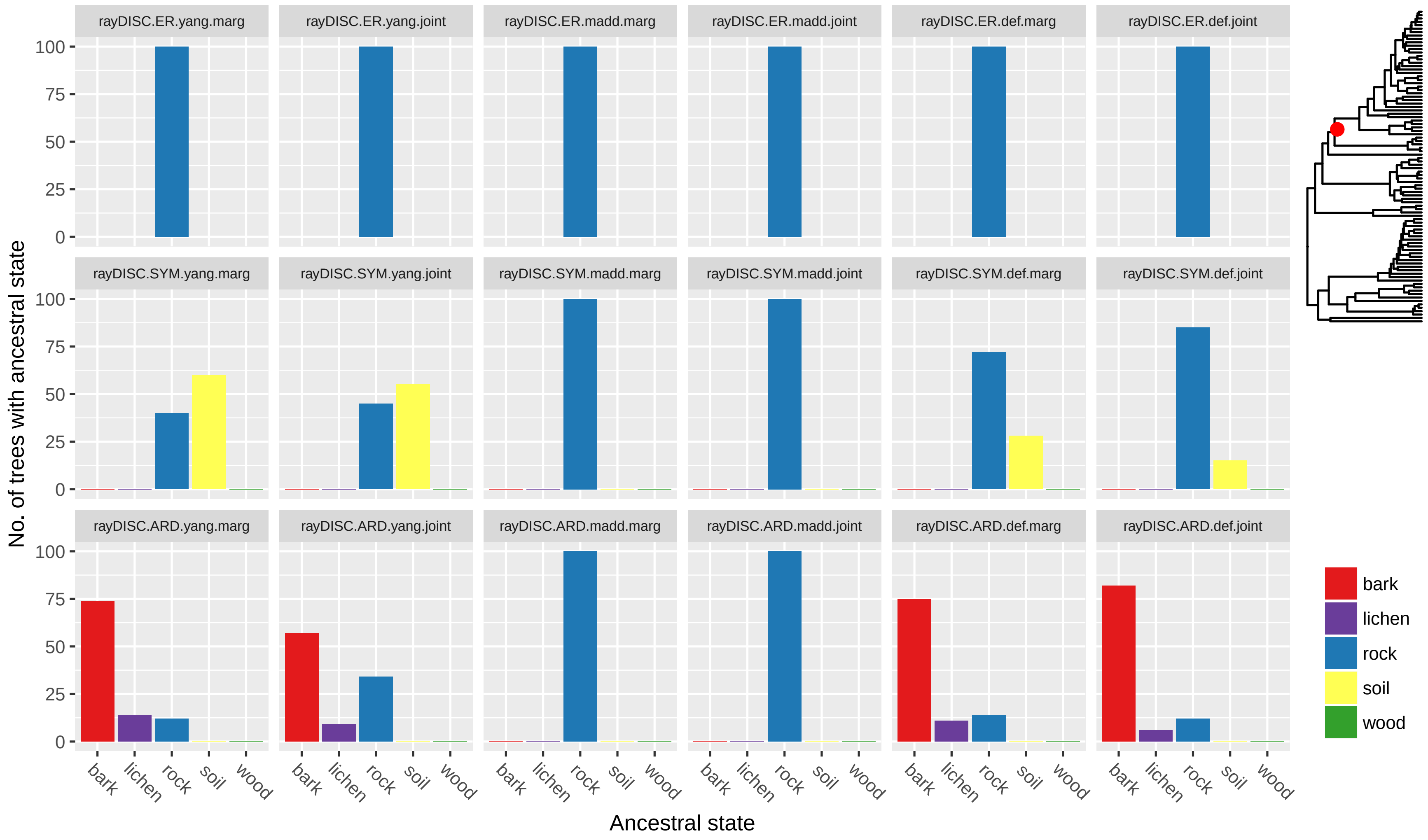

Figure S28: Ancestral states for node 6

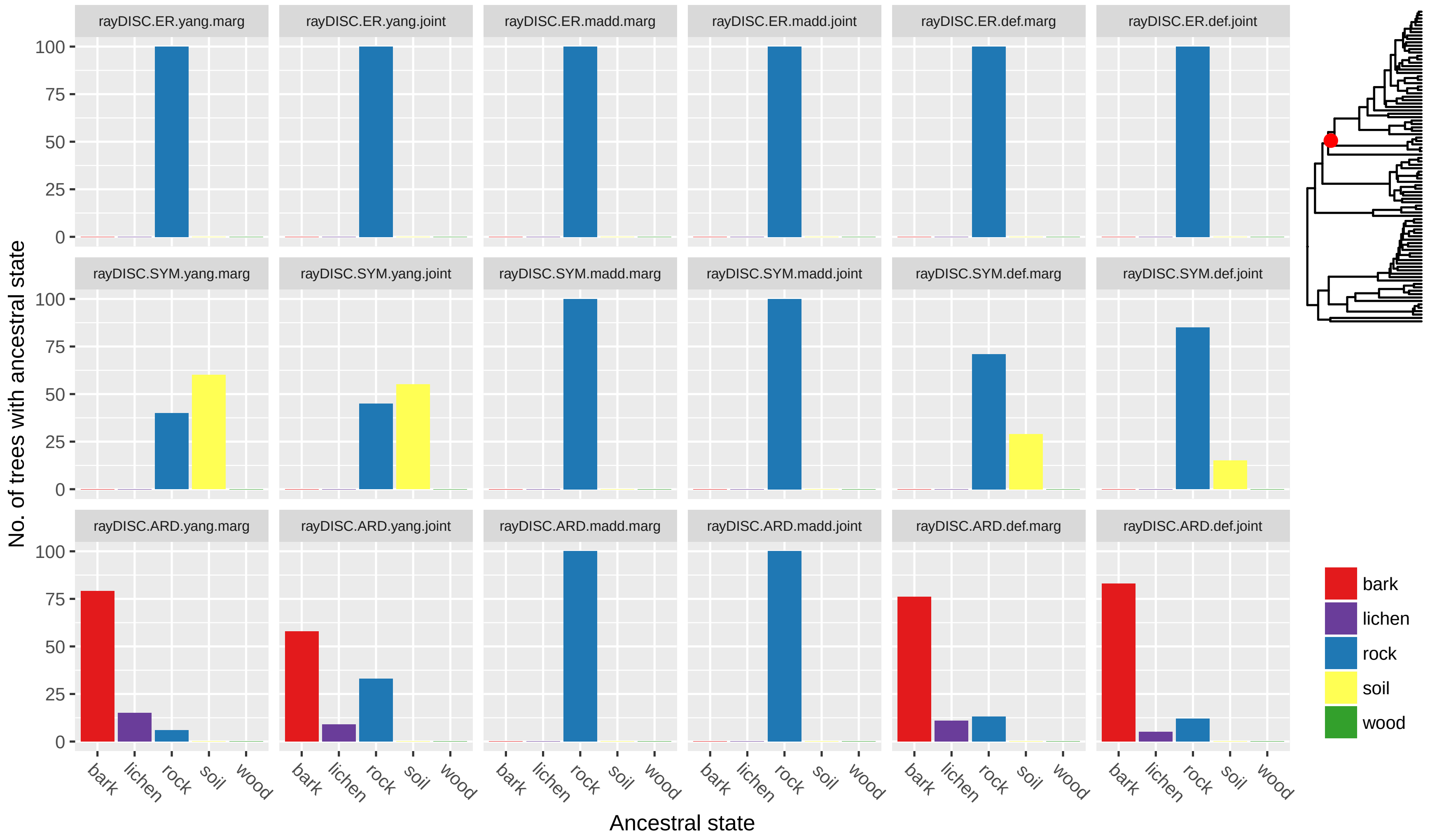

Figure S29: Ancestral states for node 7

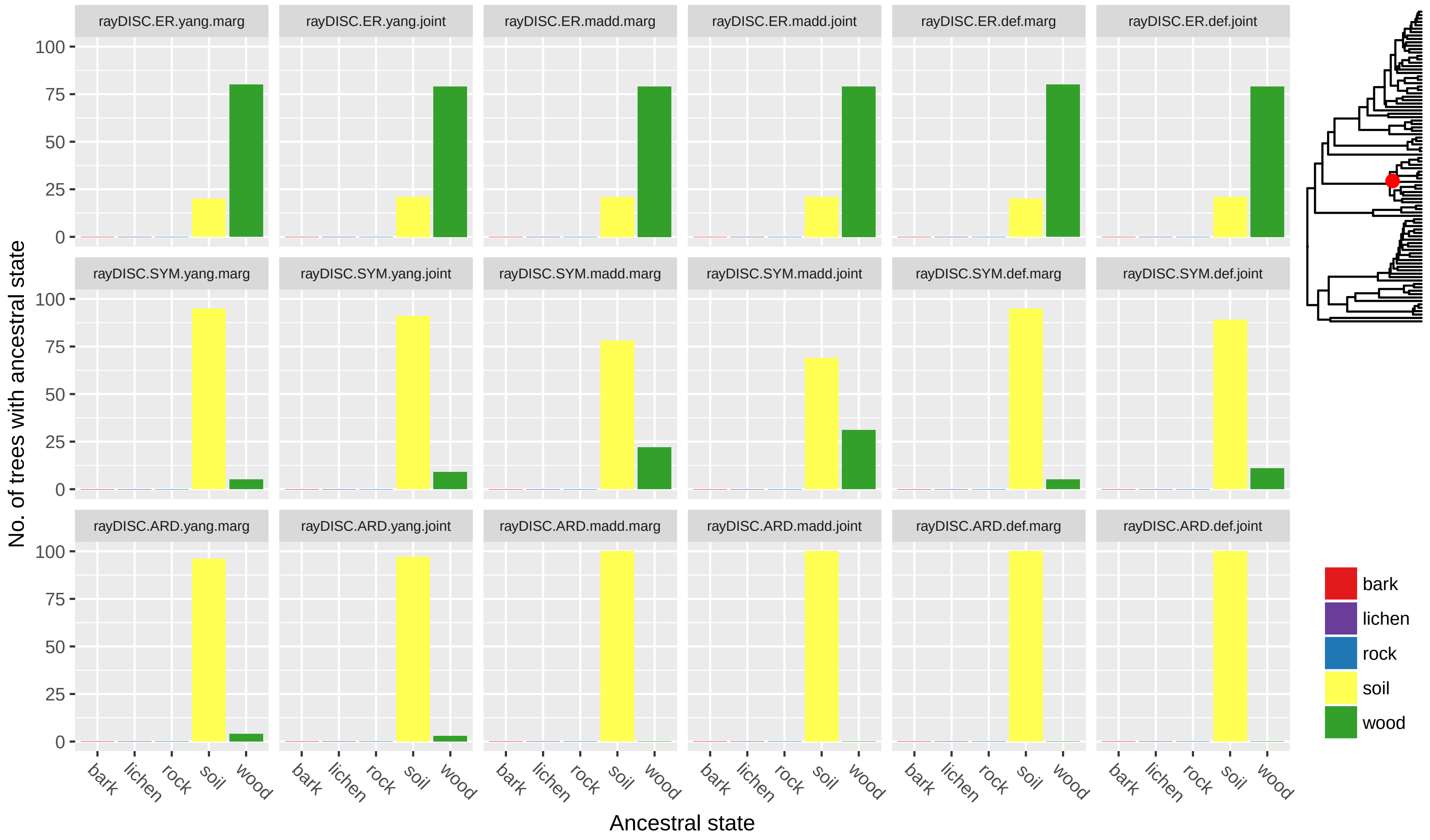

Figure S30: Ancestral states for node 8

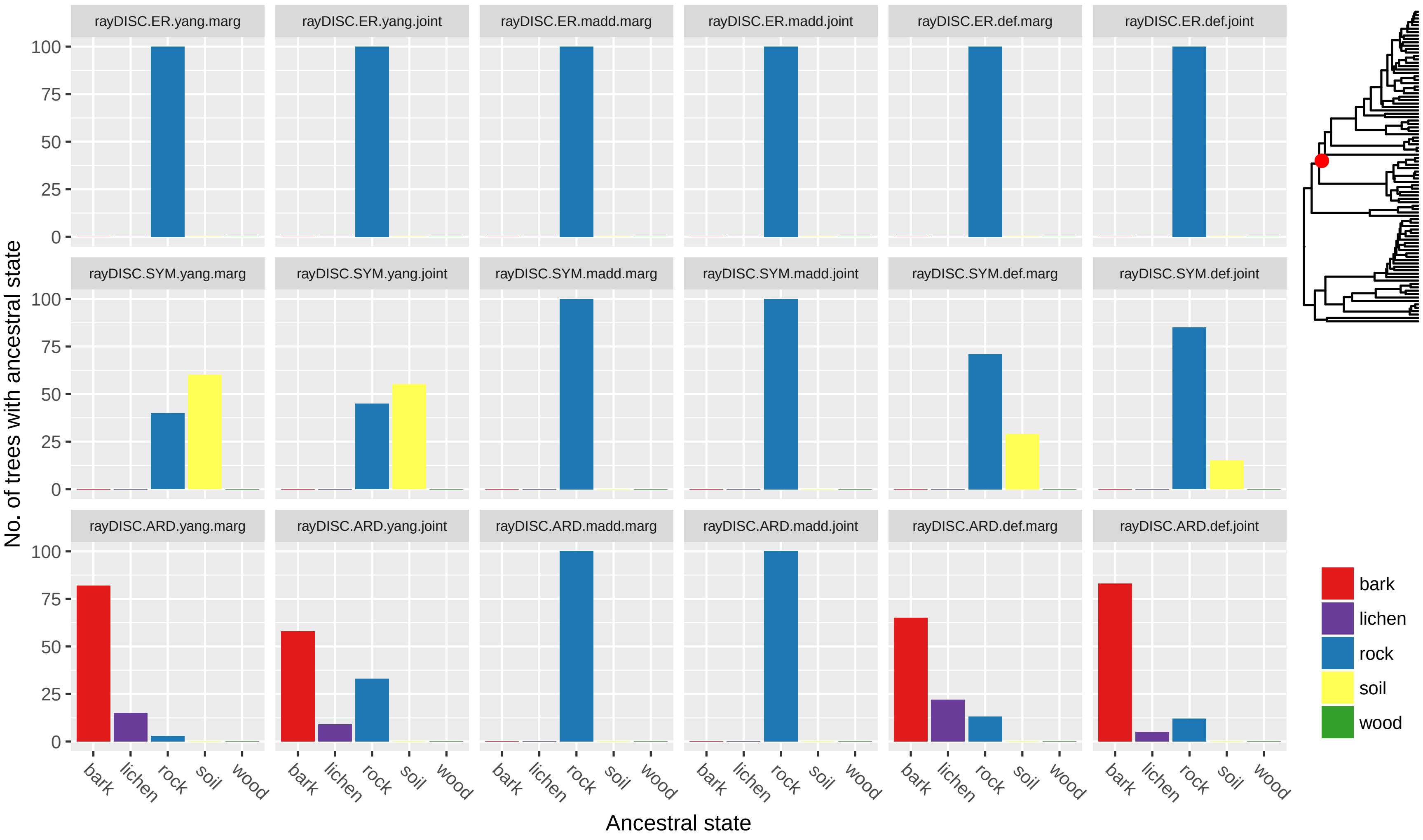

Figure S31: Ancestral states for node 9

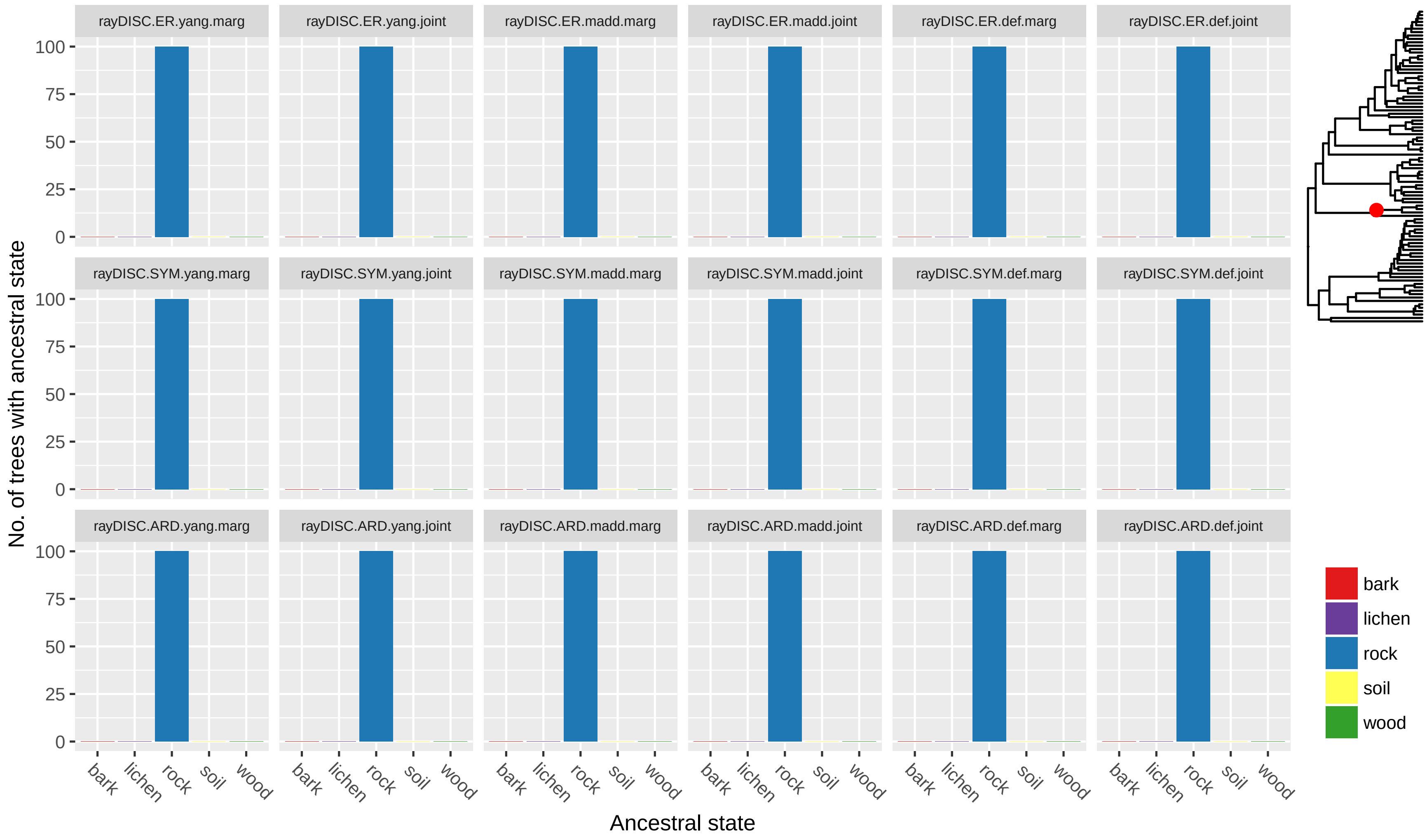

Figure S32: Ancestral states for node 10

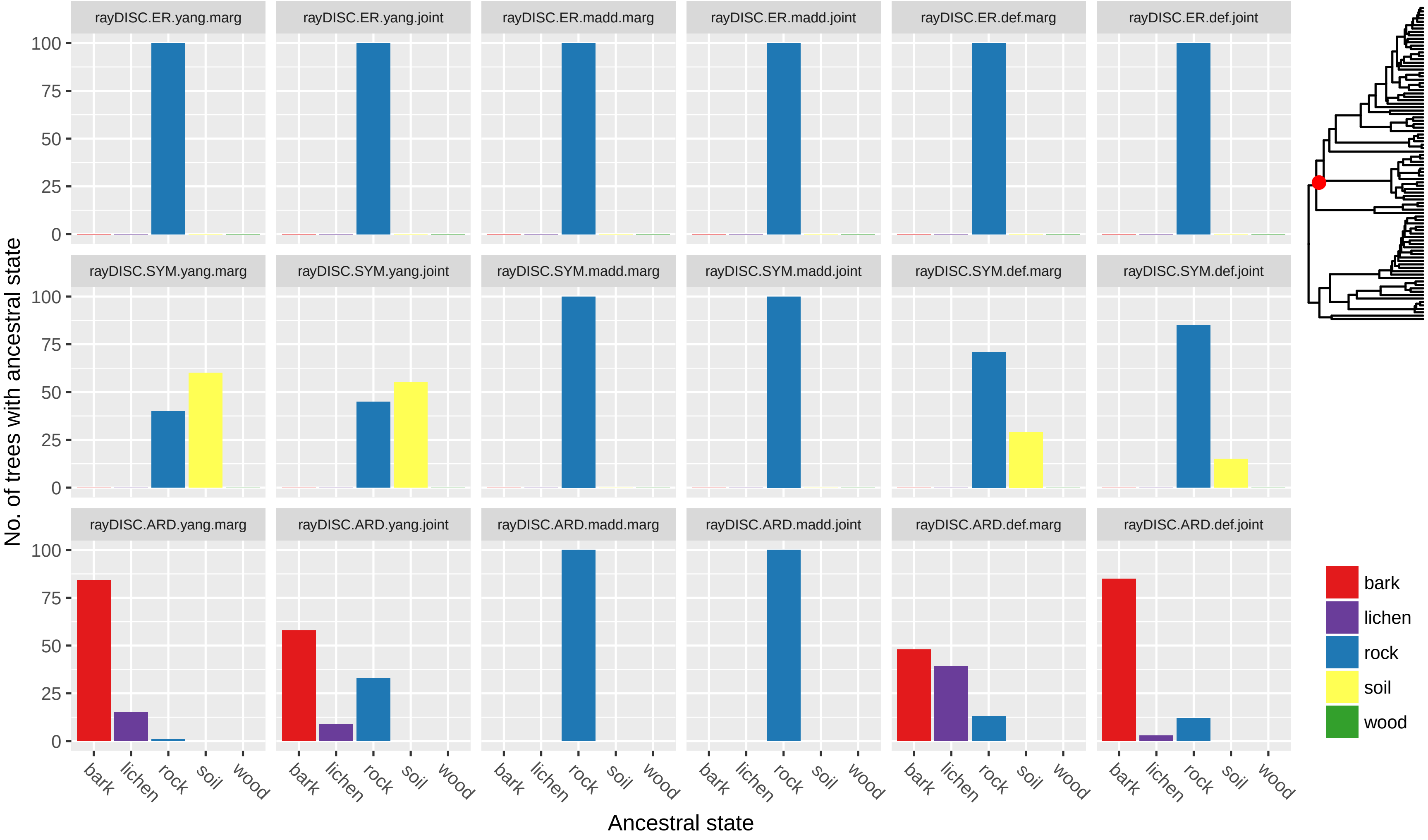

Figure S33: Ancestral states for node 11

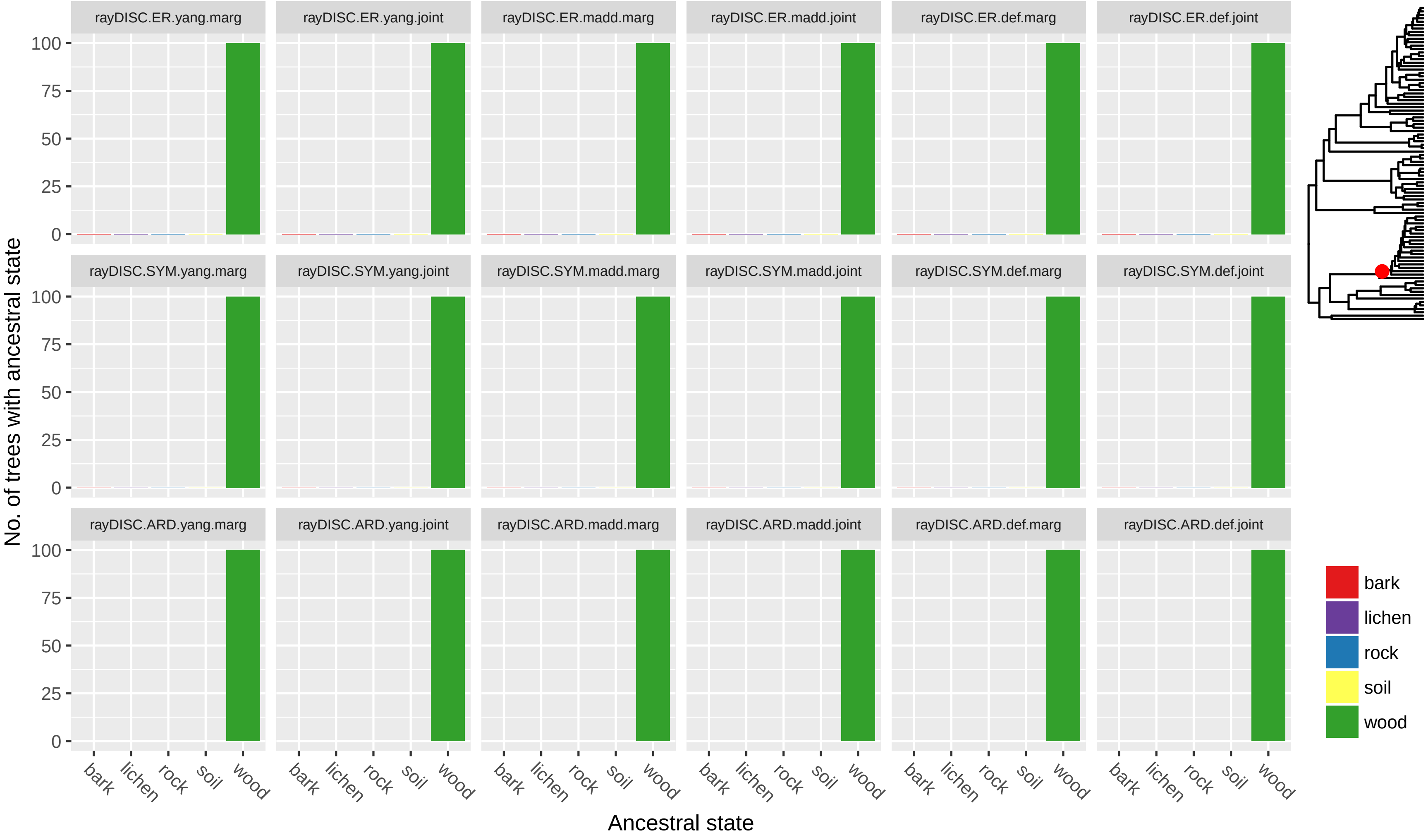

Figure S34: Ancestral states for node 12

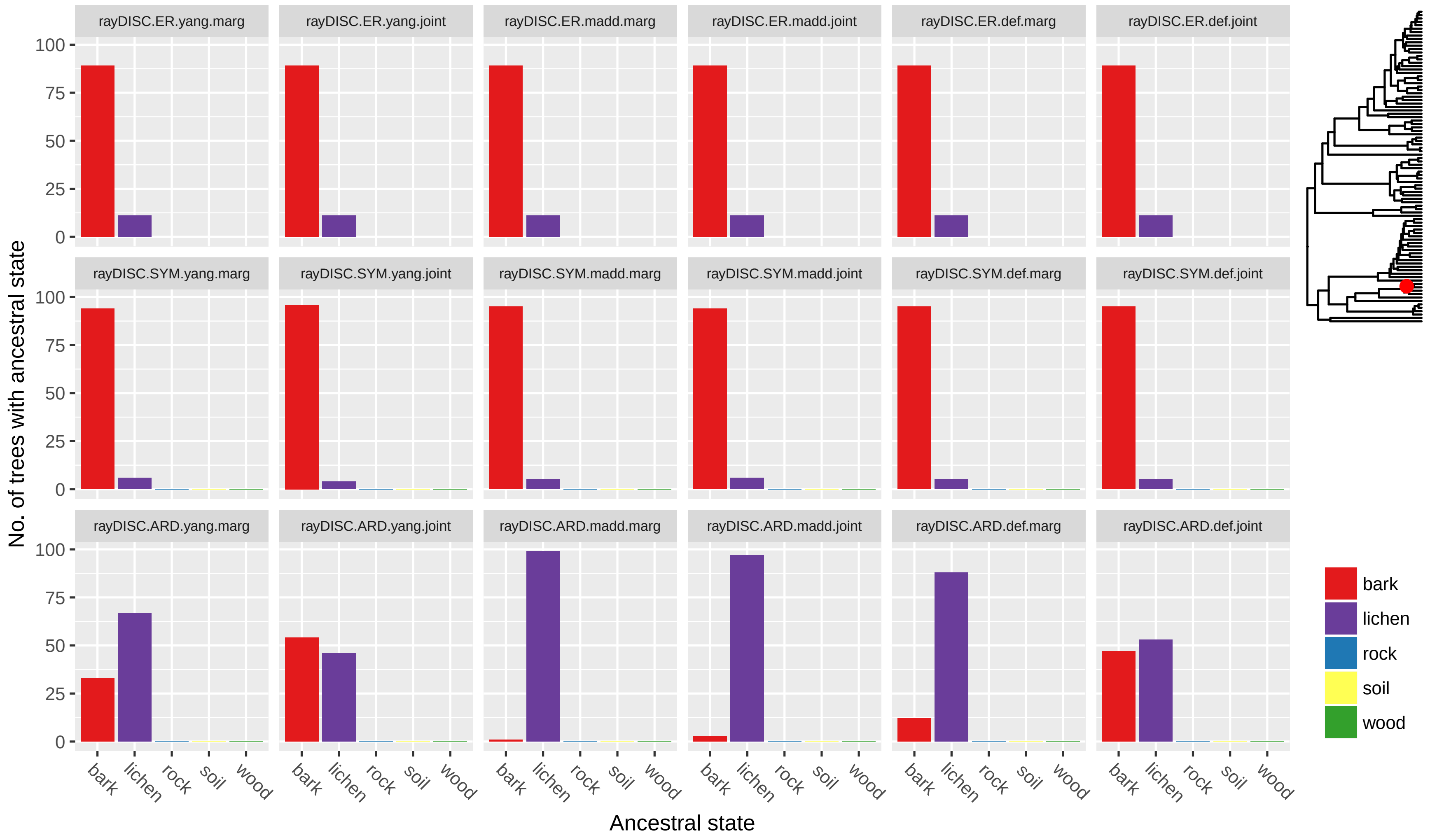

Figure S35: Ancestral states for node 13

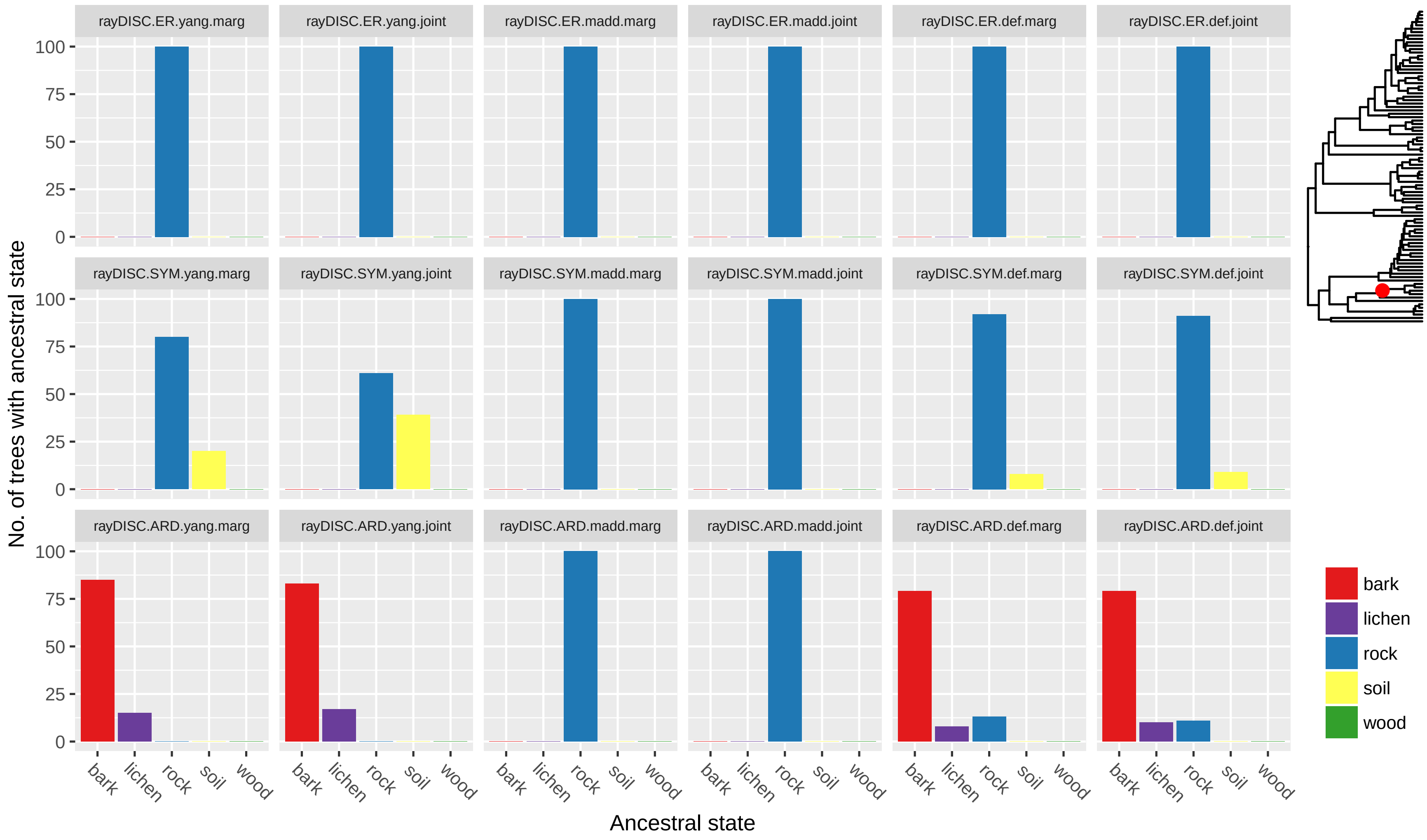

Figure S36: Ancestral states for node 14

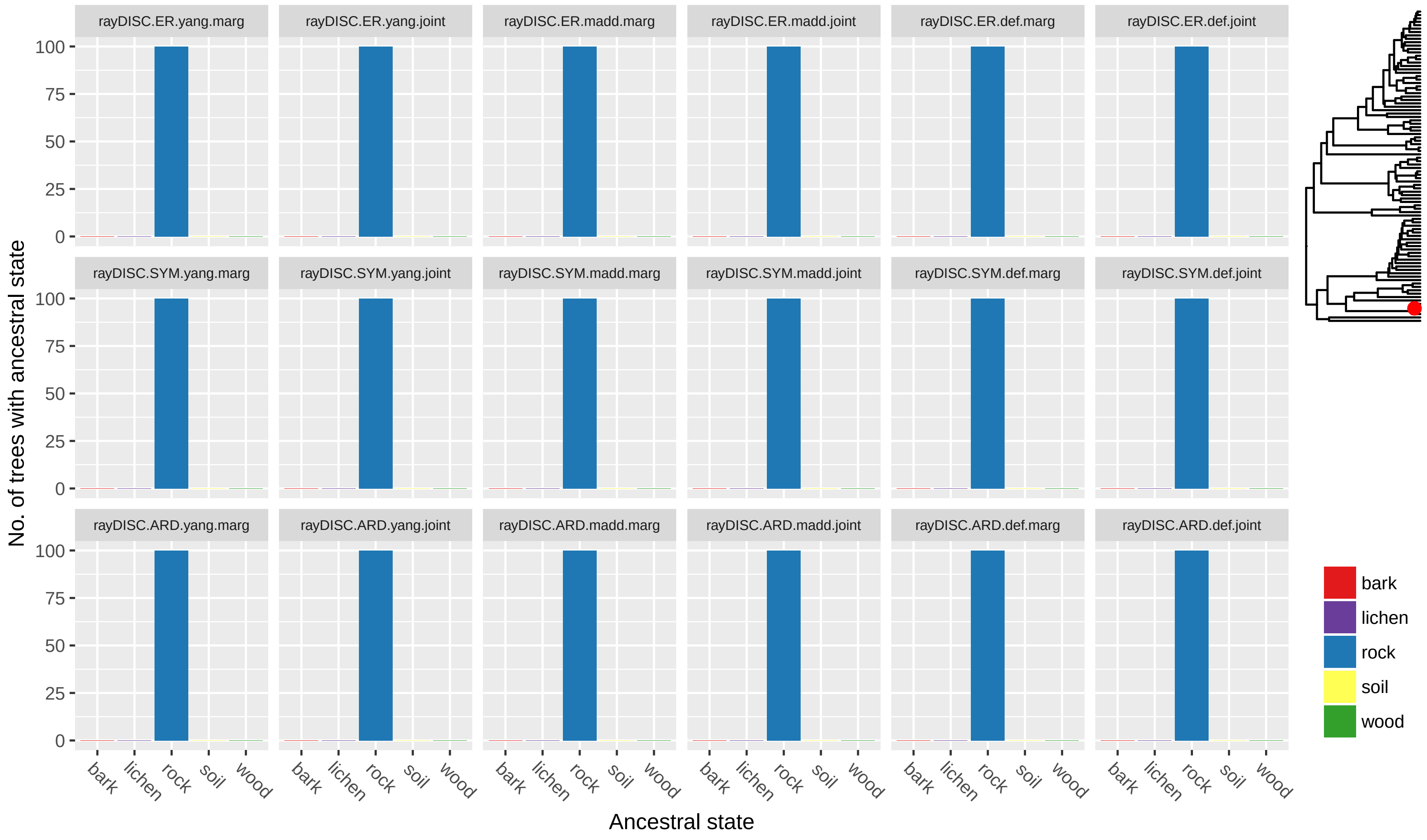

Figure S37: Ancestral states for node 15

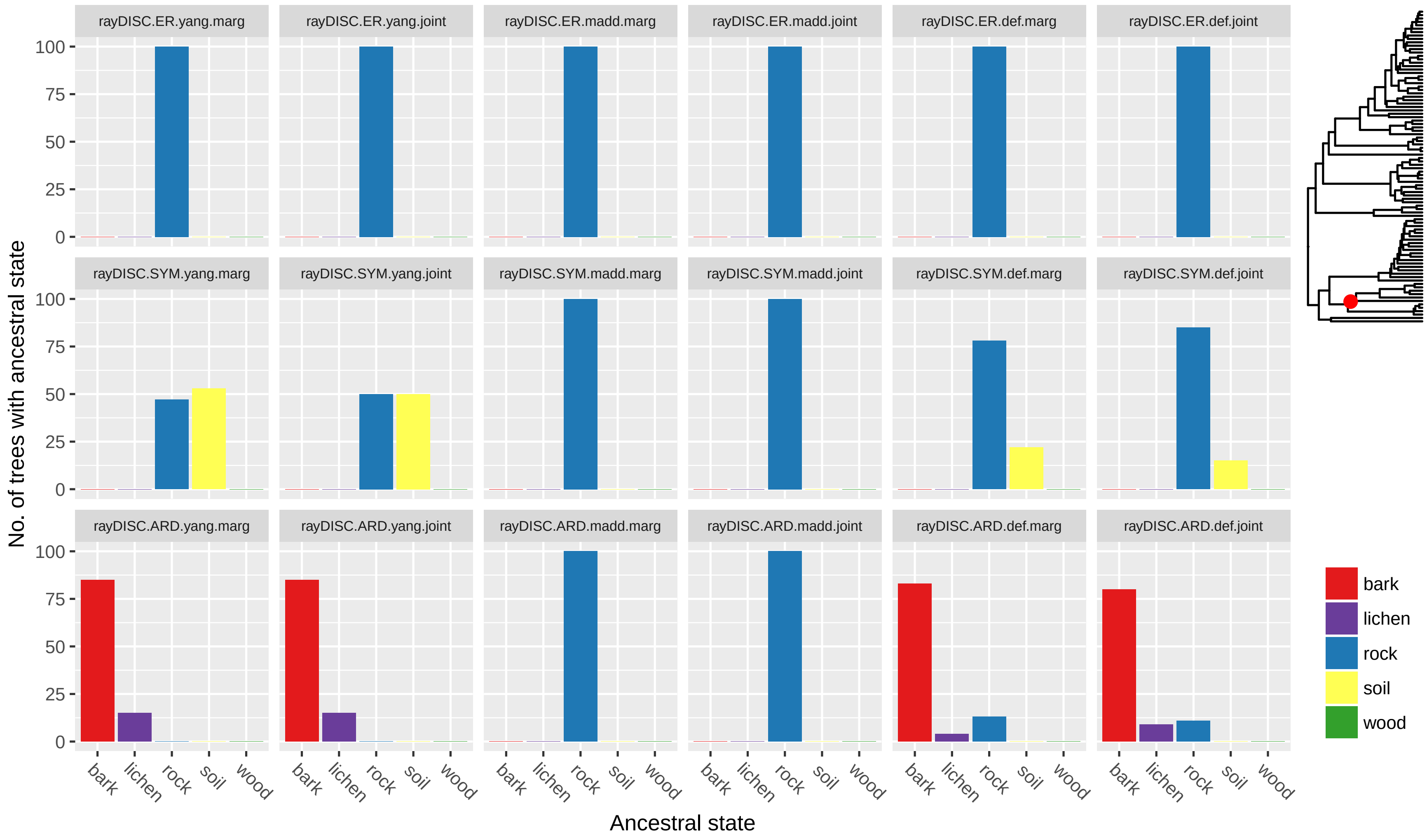

Figure S38: Ancestral states for node 16

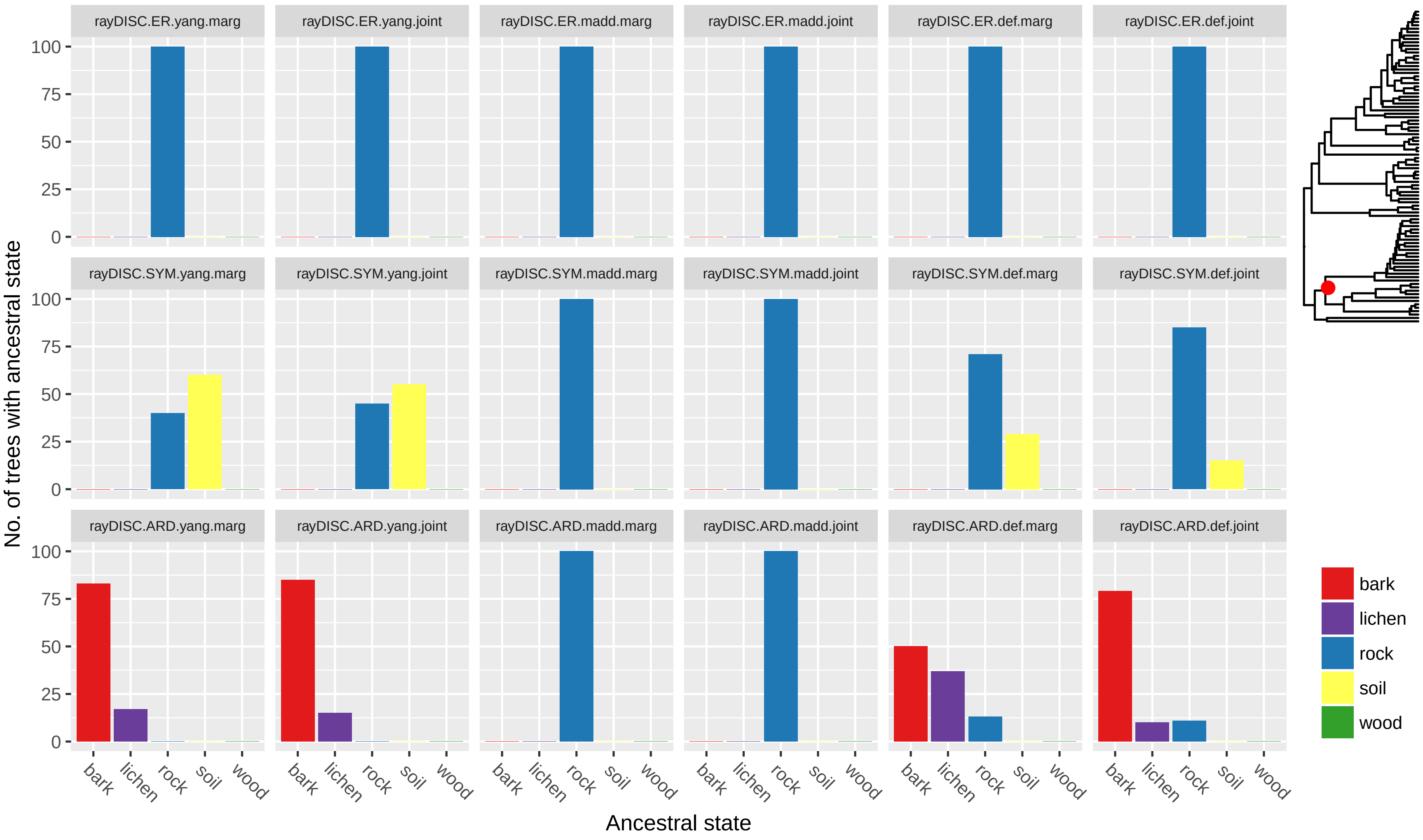

Figure S39: Ancestral states for node 17

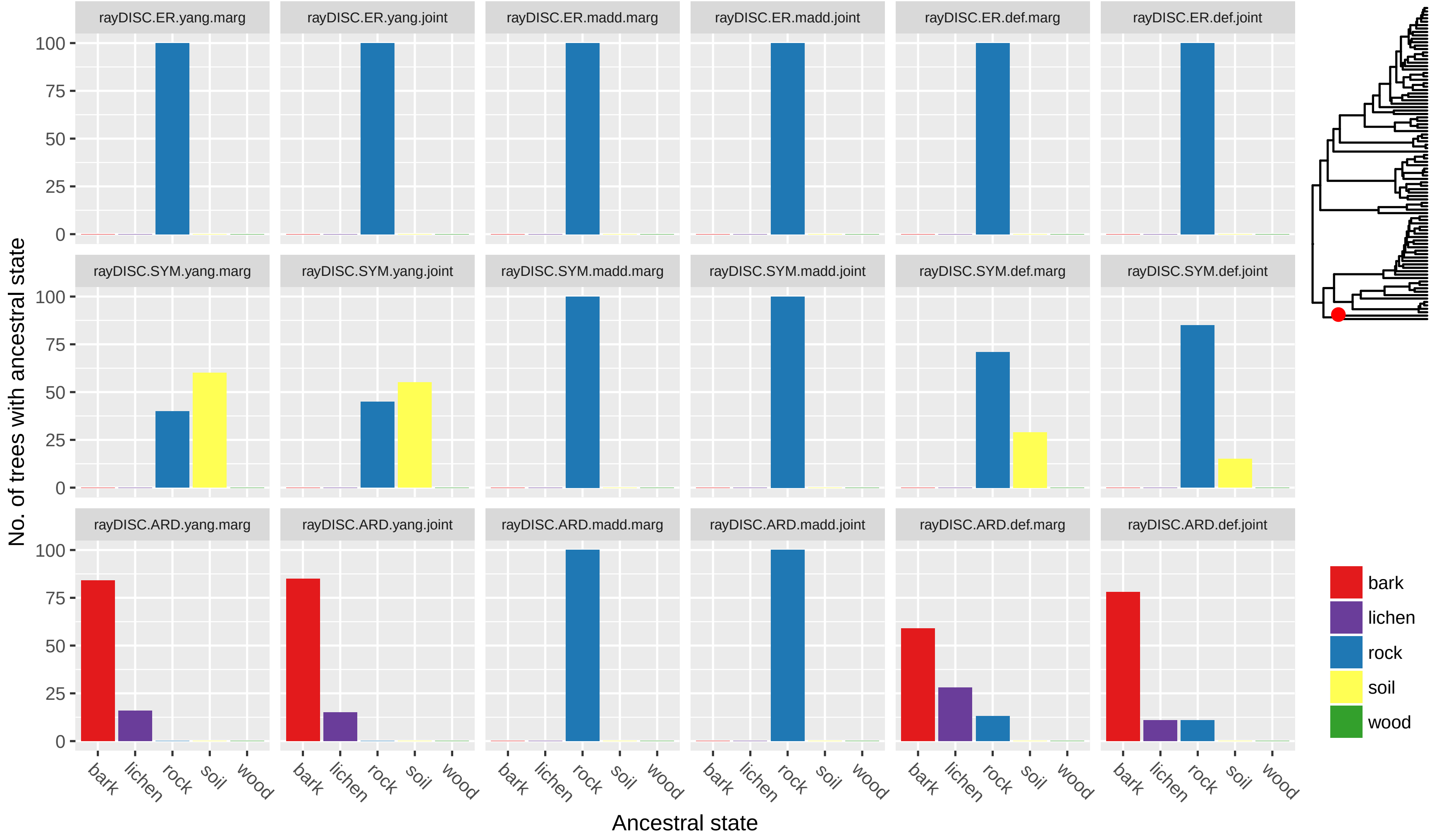

Figure S40: Ancestral states for node 18

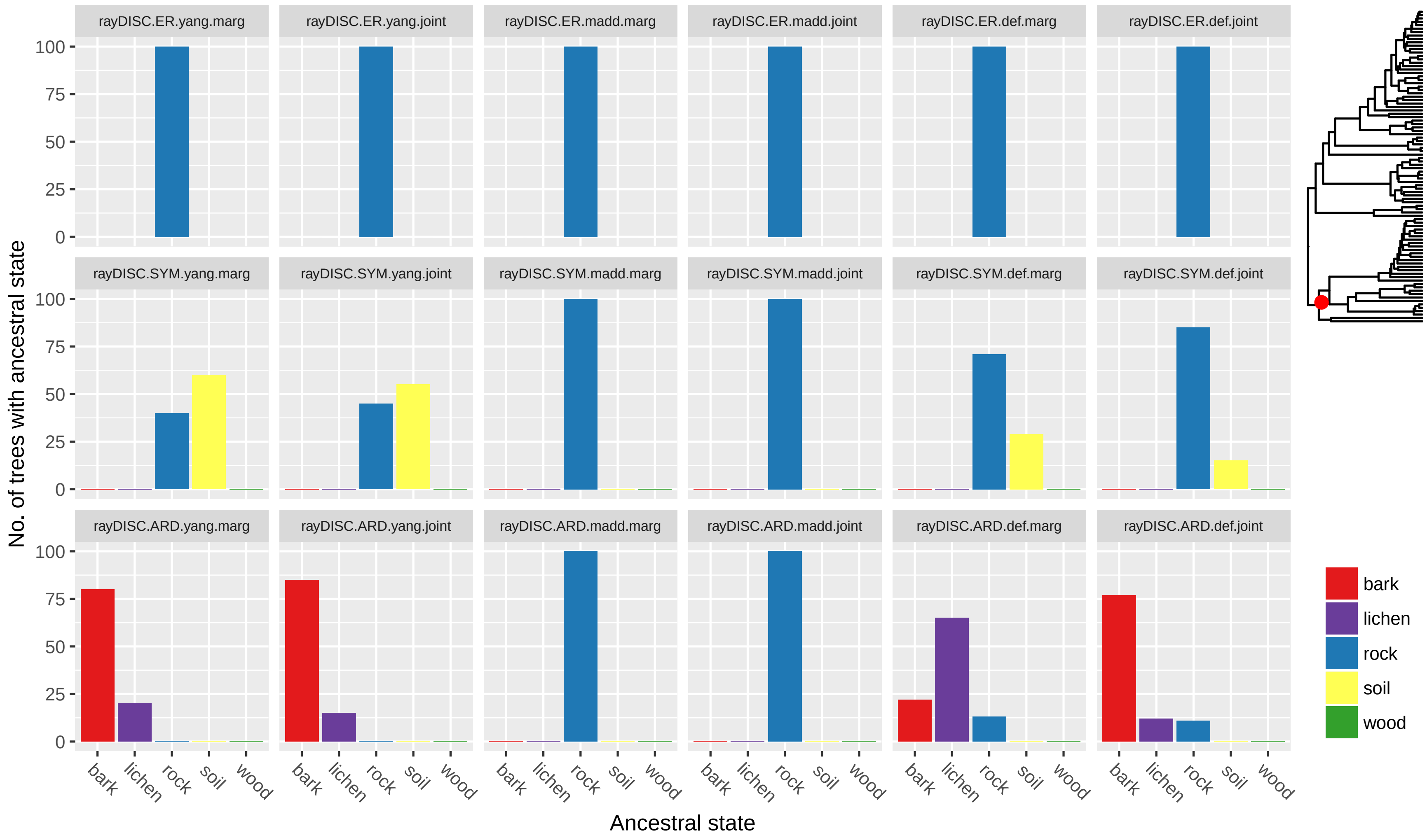

Figure S41: Ancestral states for node 19

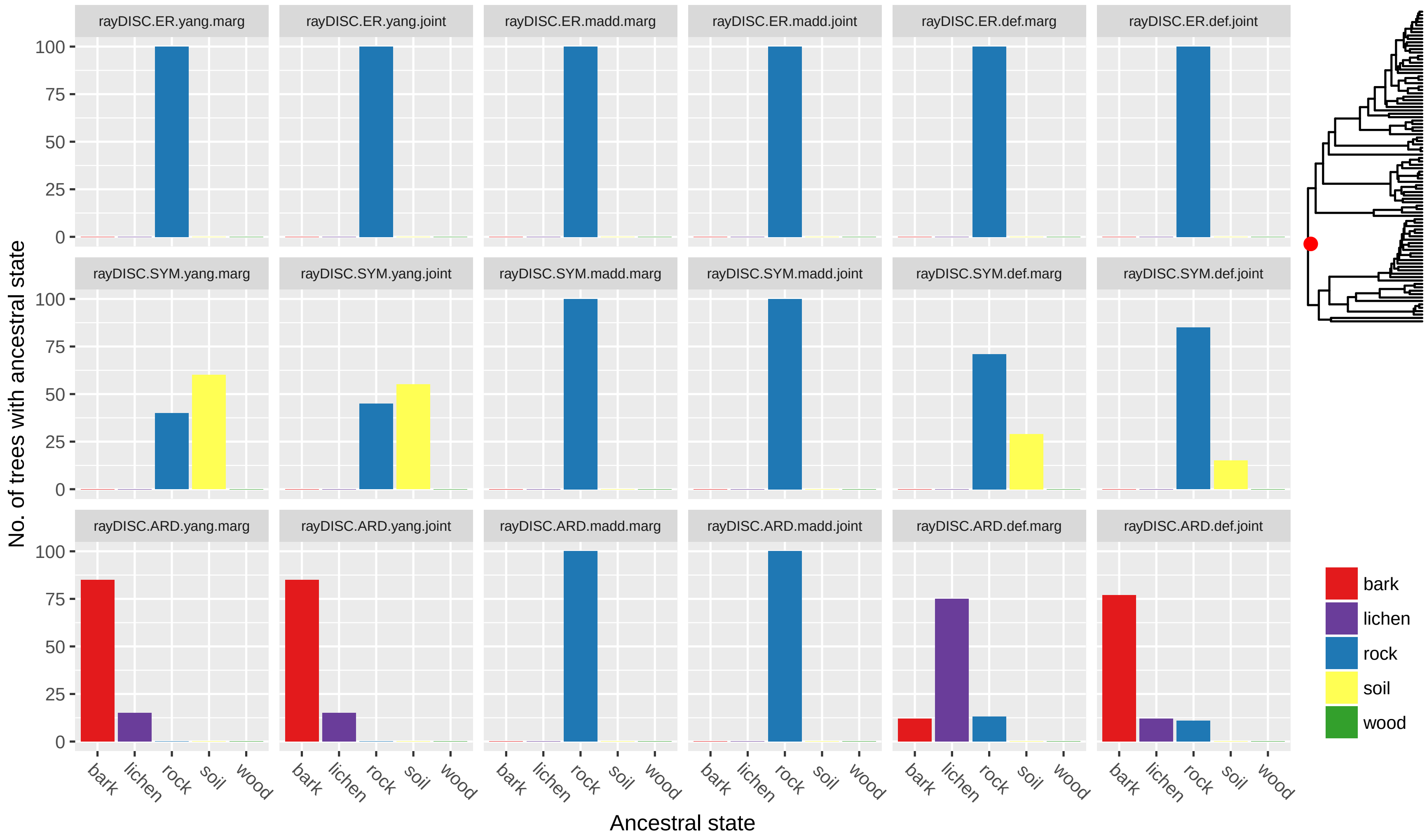

Figure S42-S60 - Results of ancestral state estimation of the preferred substrate character with make.simmap for 19 nodes of the trapelioid phylogeny

Ancestral state estimations of the preferred substrate character for 19 nodes of the trapelioid phylogeny based on stochastic character mapping using phytools imposing 6 different models. Please refer to the main text for details.

Figure S42: Ancestral states for node 1

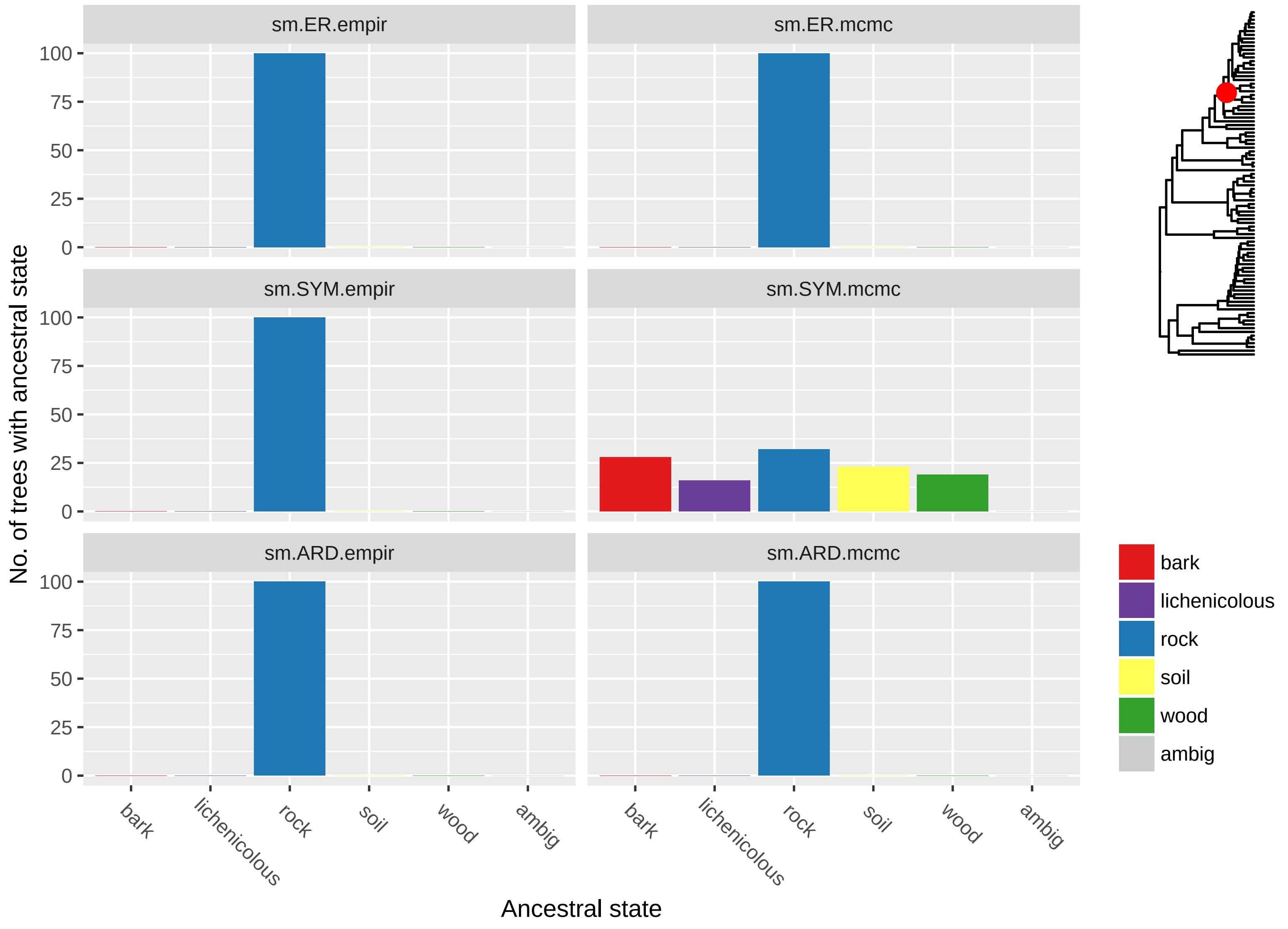

Figure S43: Ancestral states for node 2

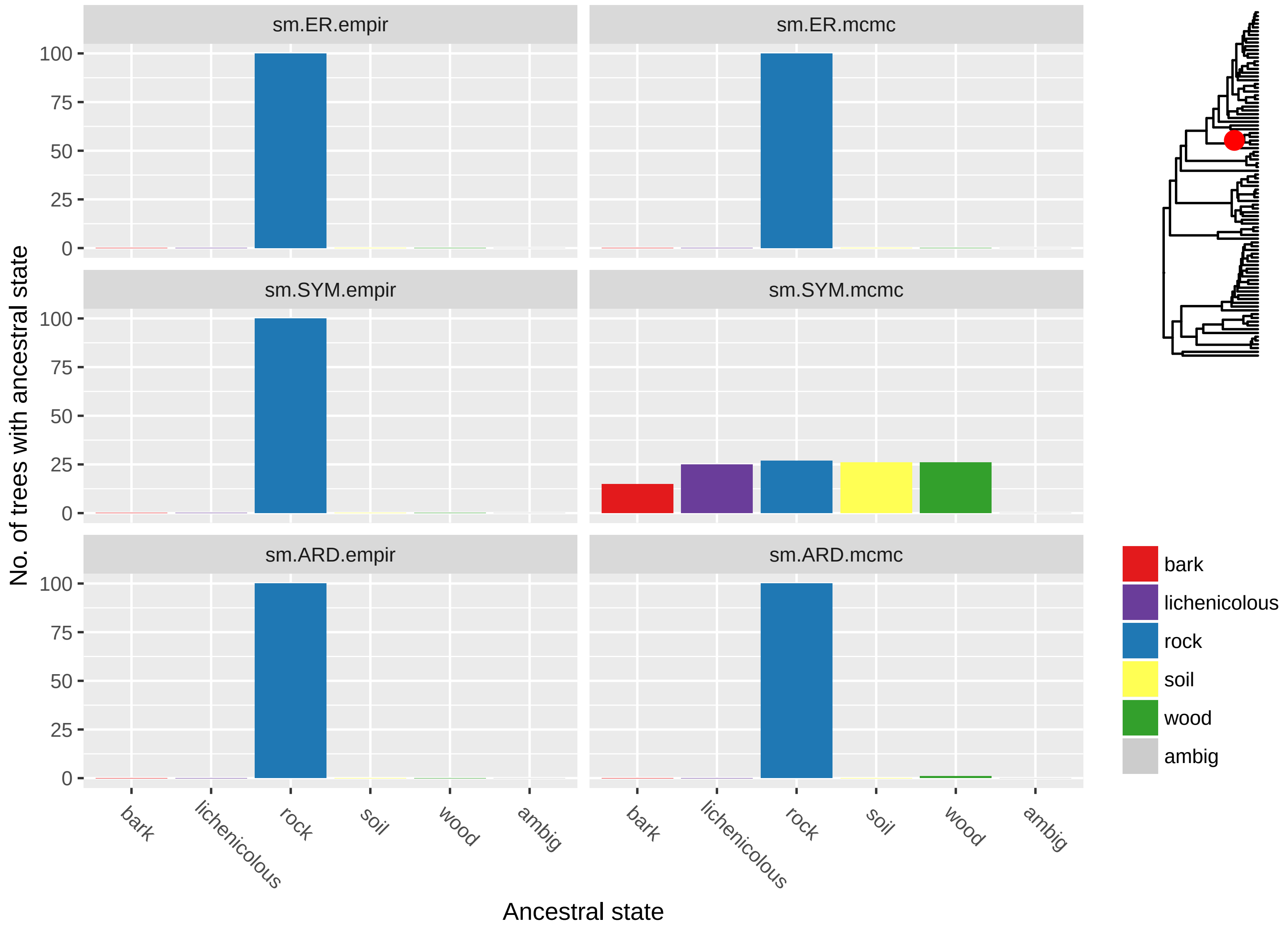

Figure S44: Ancestral states for node 3

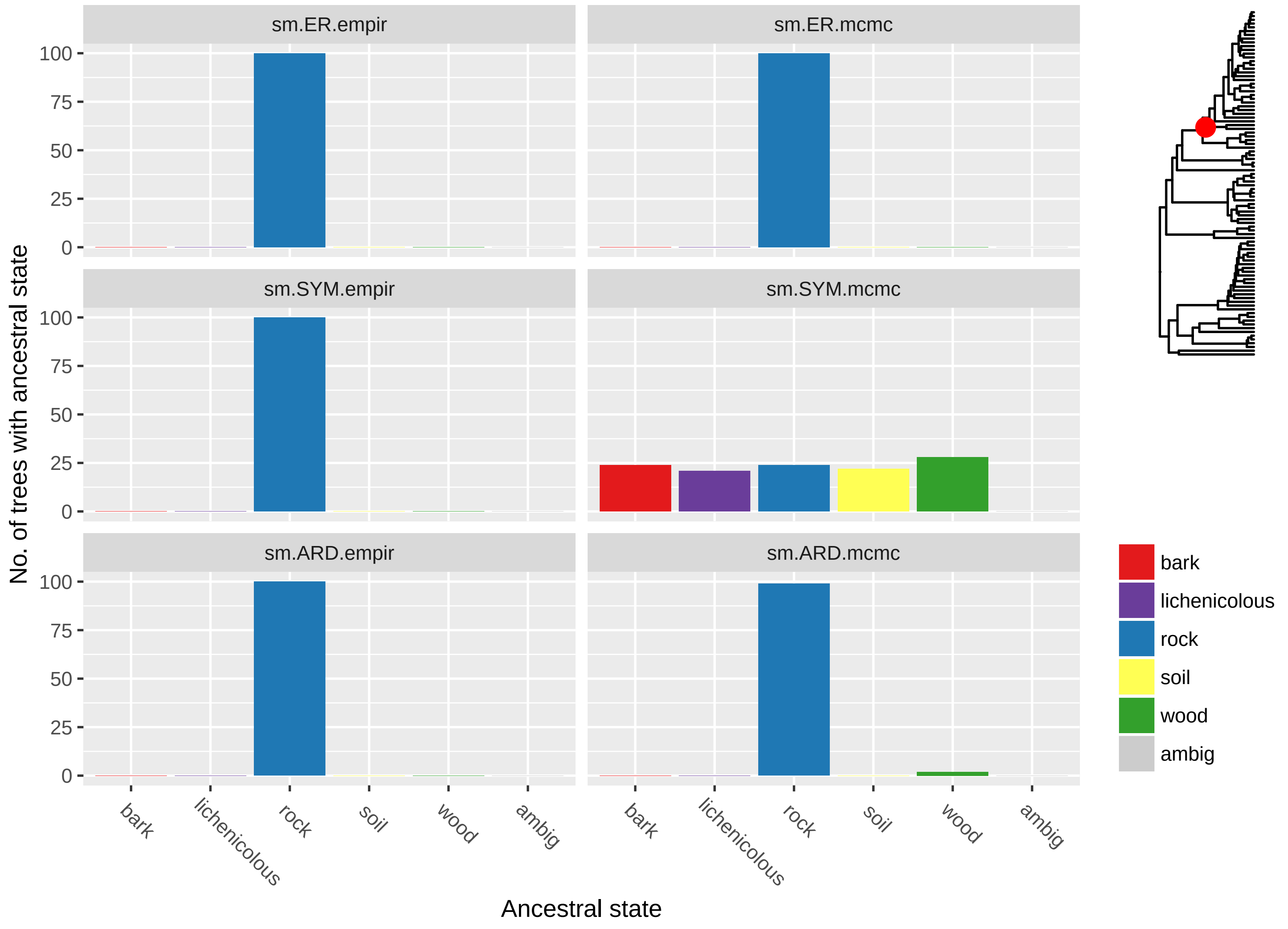

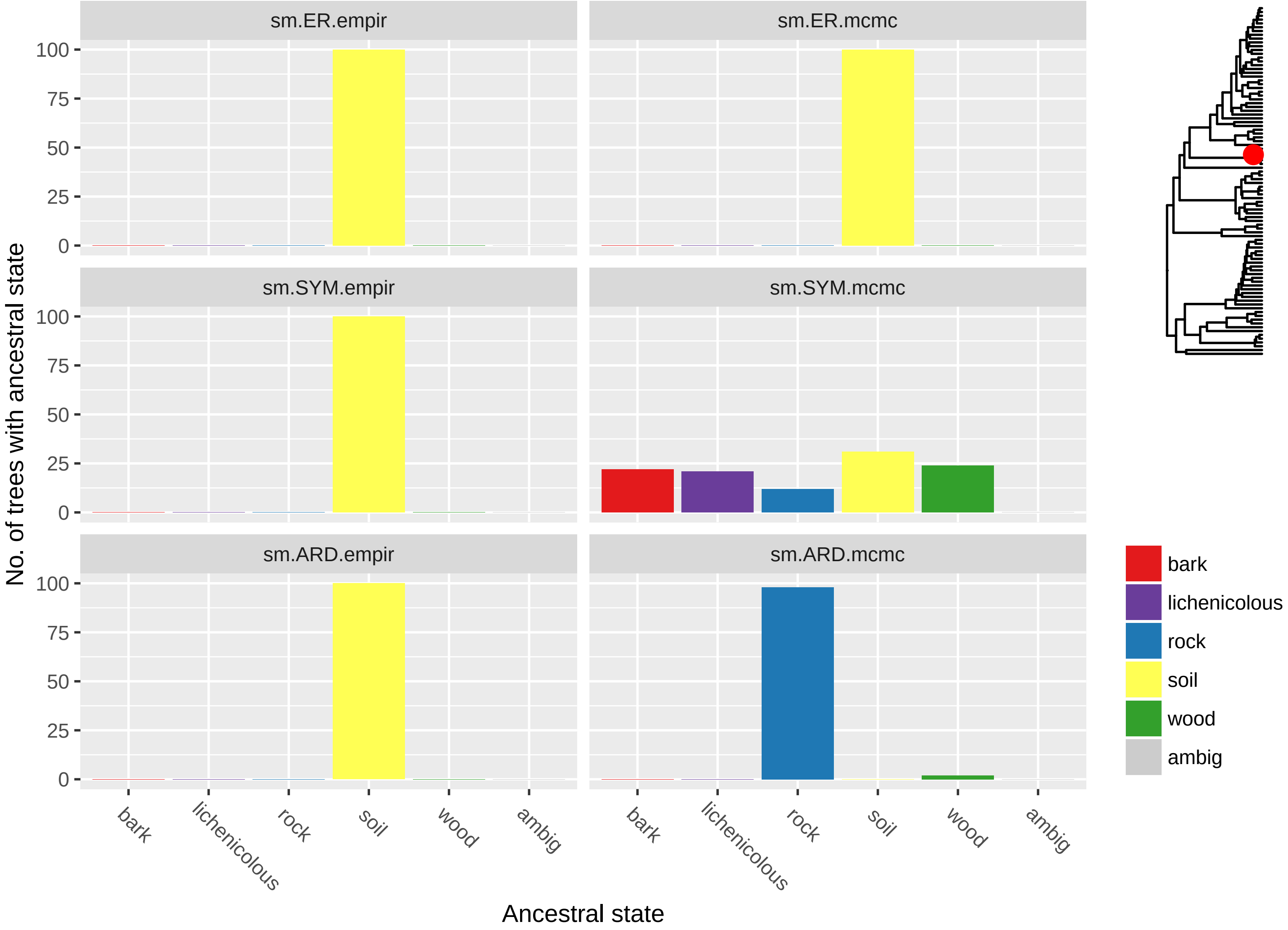

Figure S45: Ancestral states for node 4

Figure S46: Ancestral states for node 5

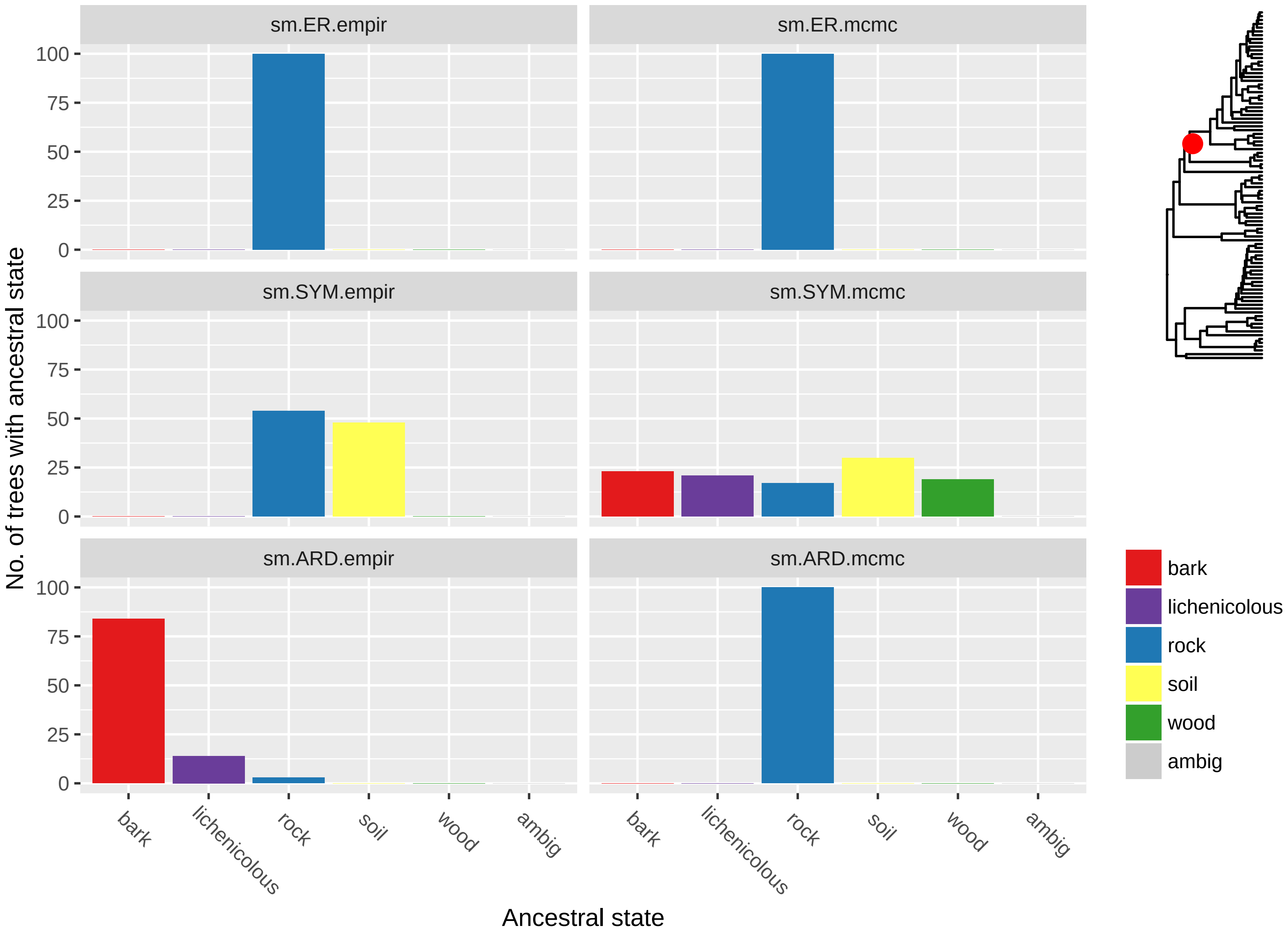

Figure S47: Ancestral states for node 6

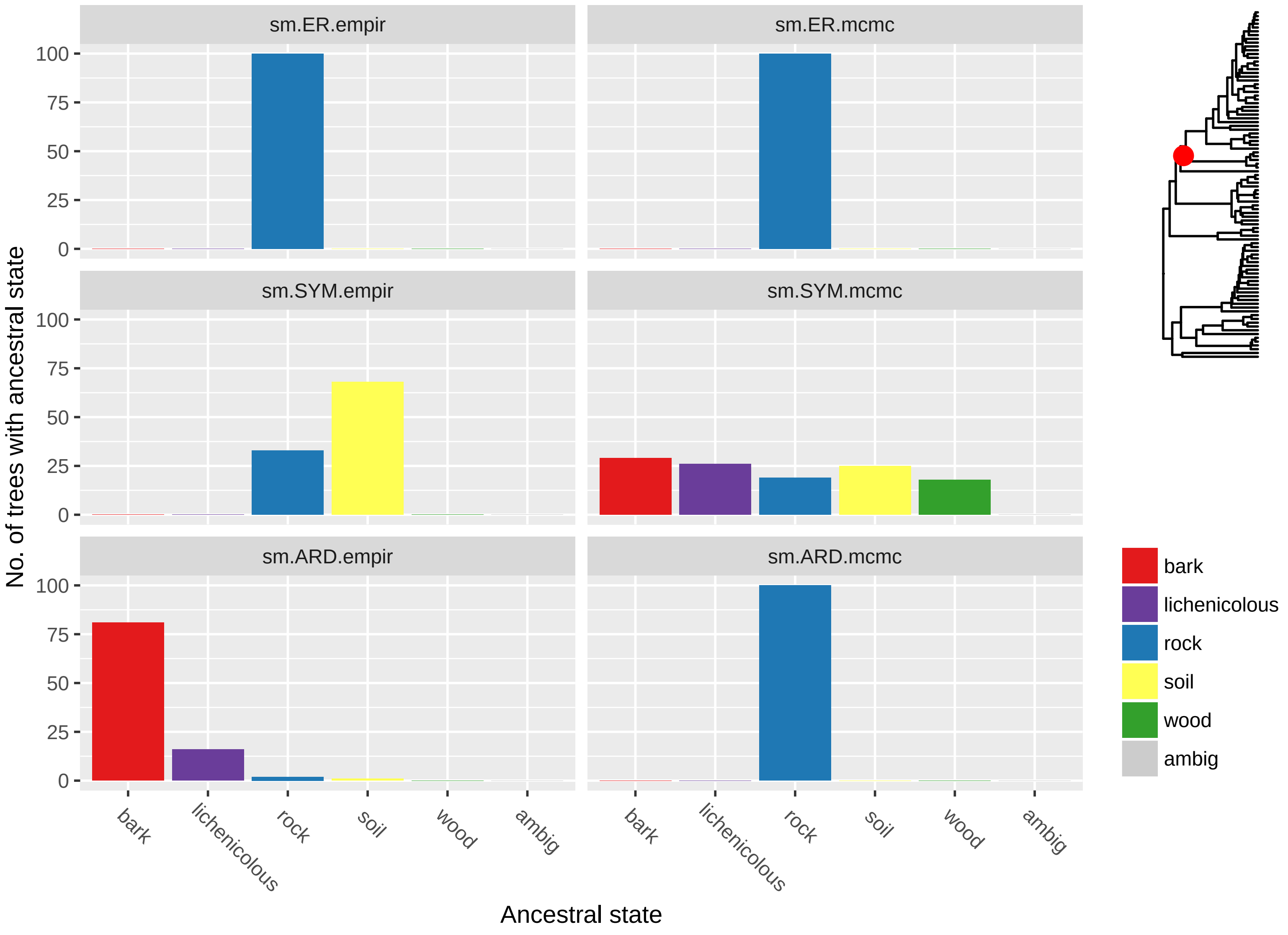

Figure S48: Ancestral states for node 7

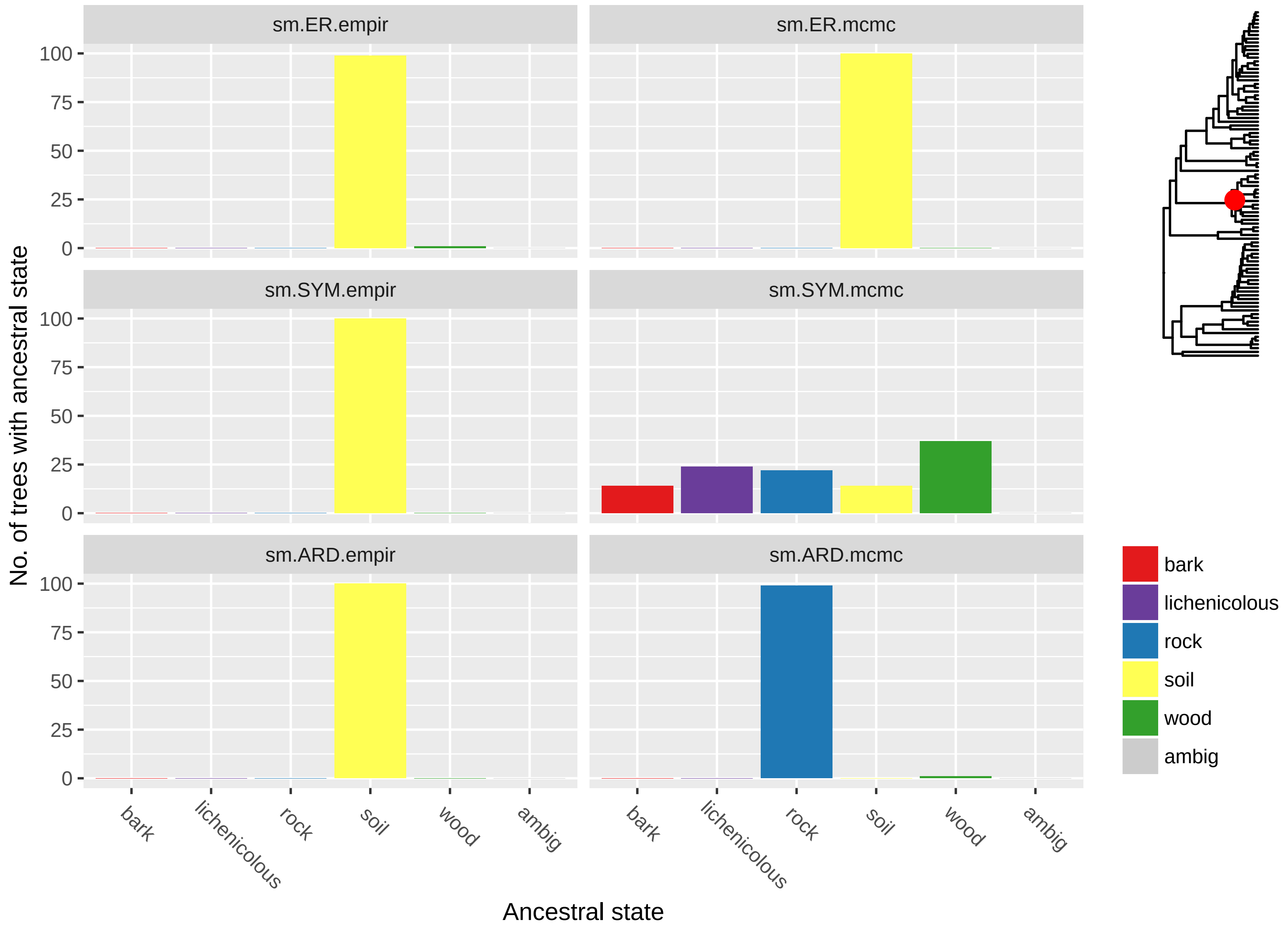

Figure S49: Ancestral states for node 8

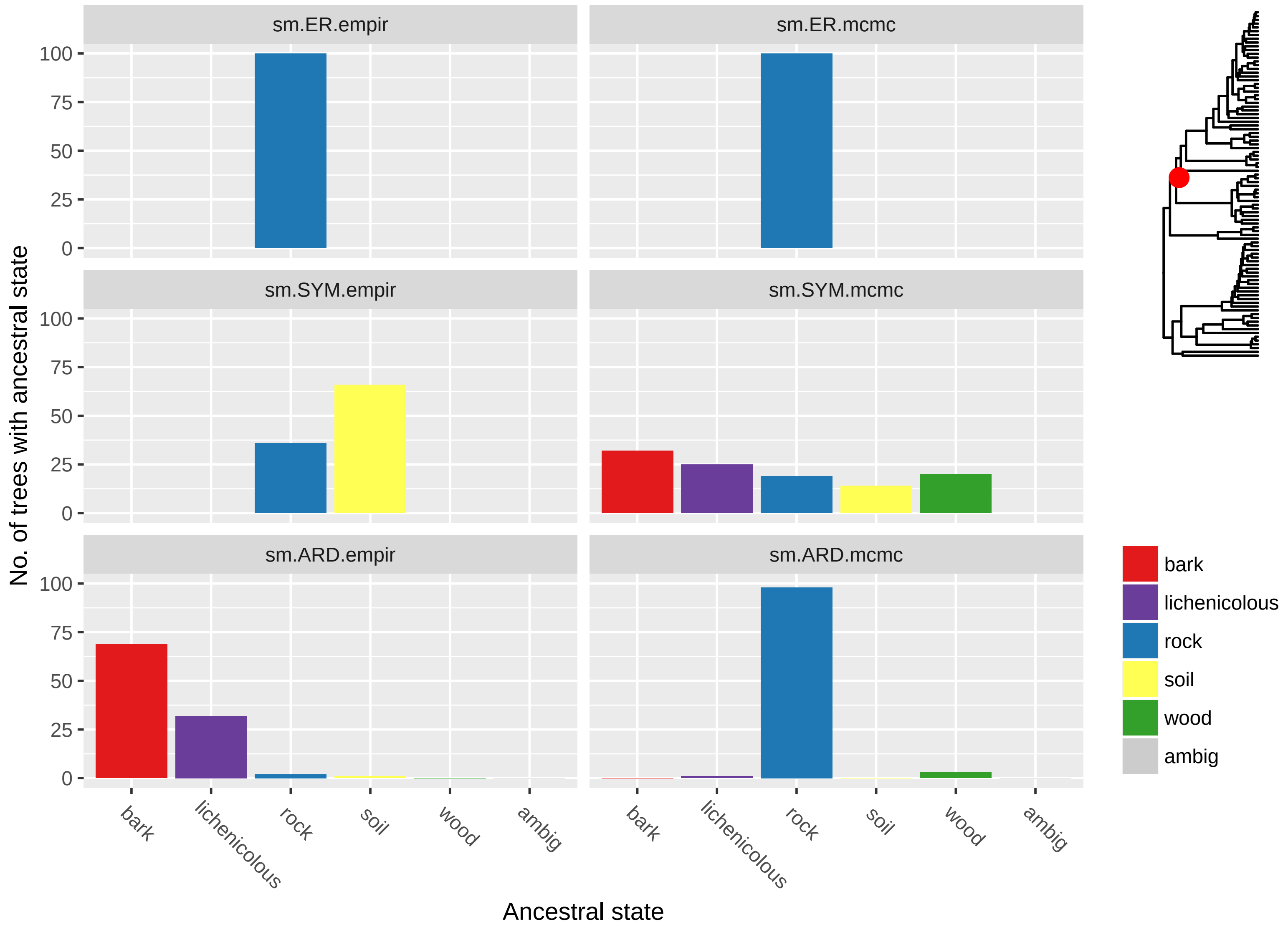

Figure S50: Ancestral states for node 9

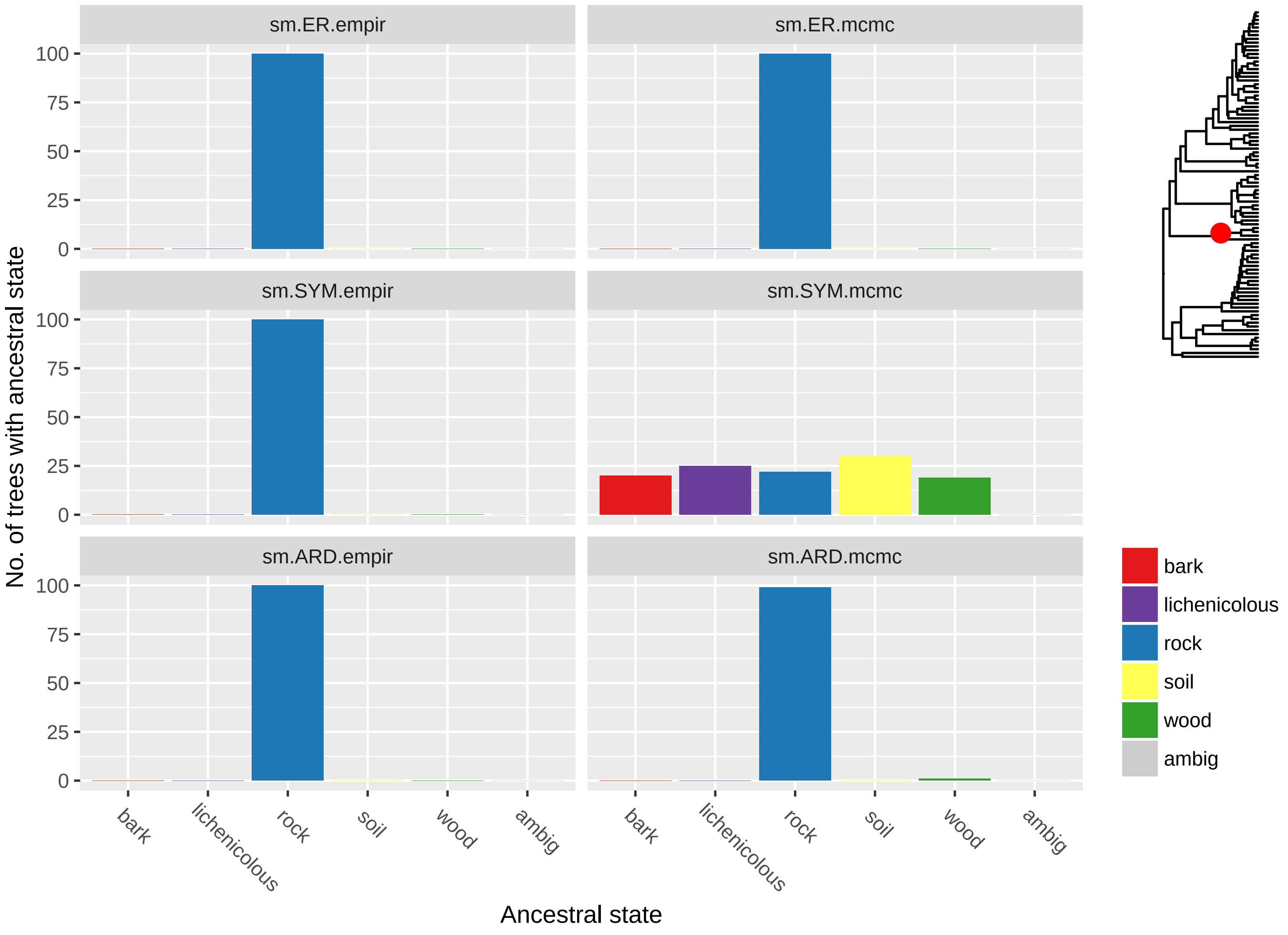

Figure S51: Ancestral states for node 10

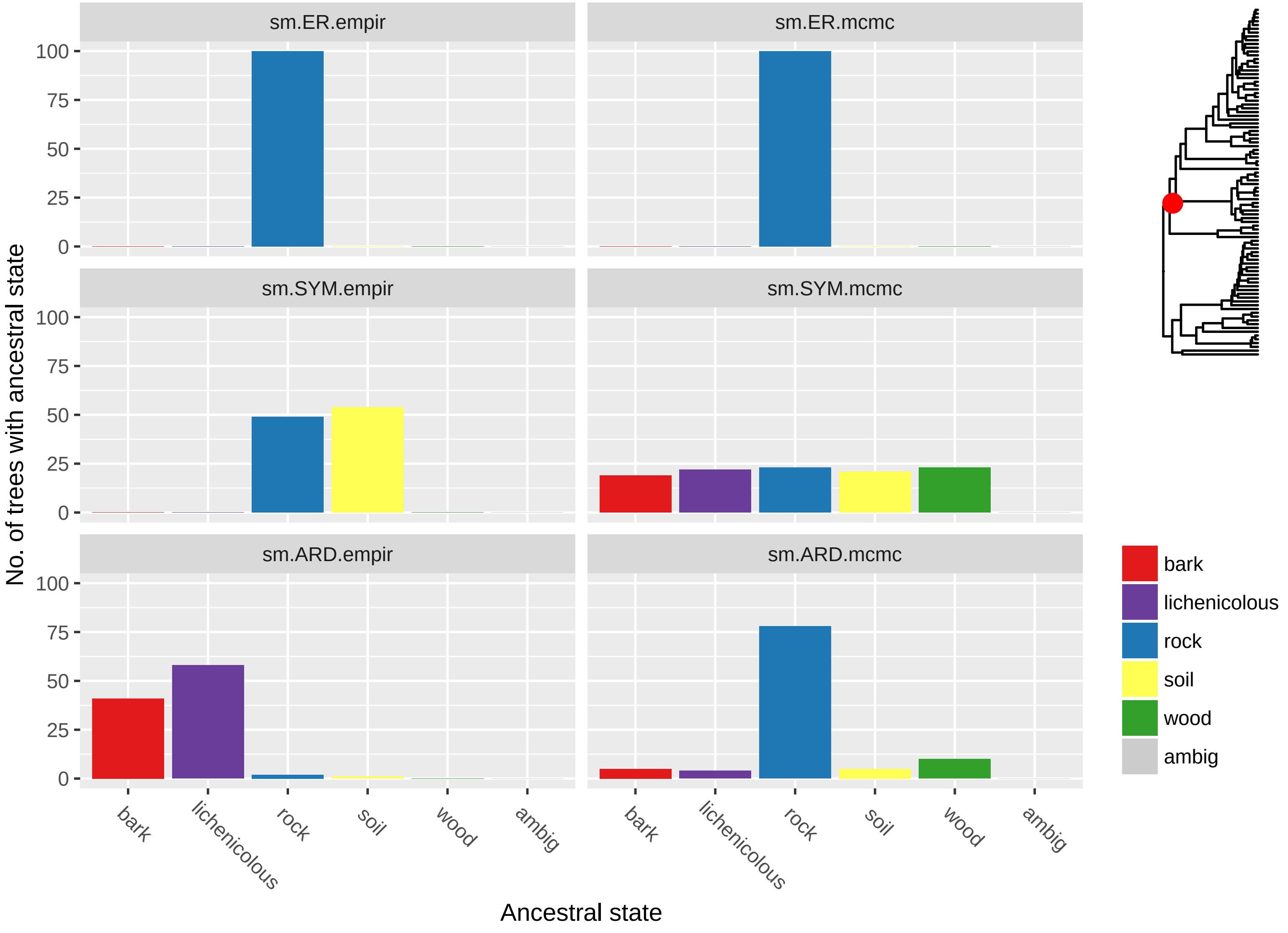

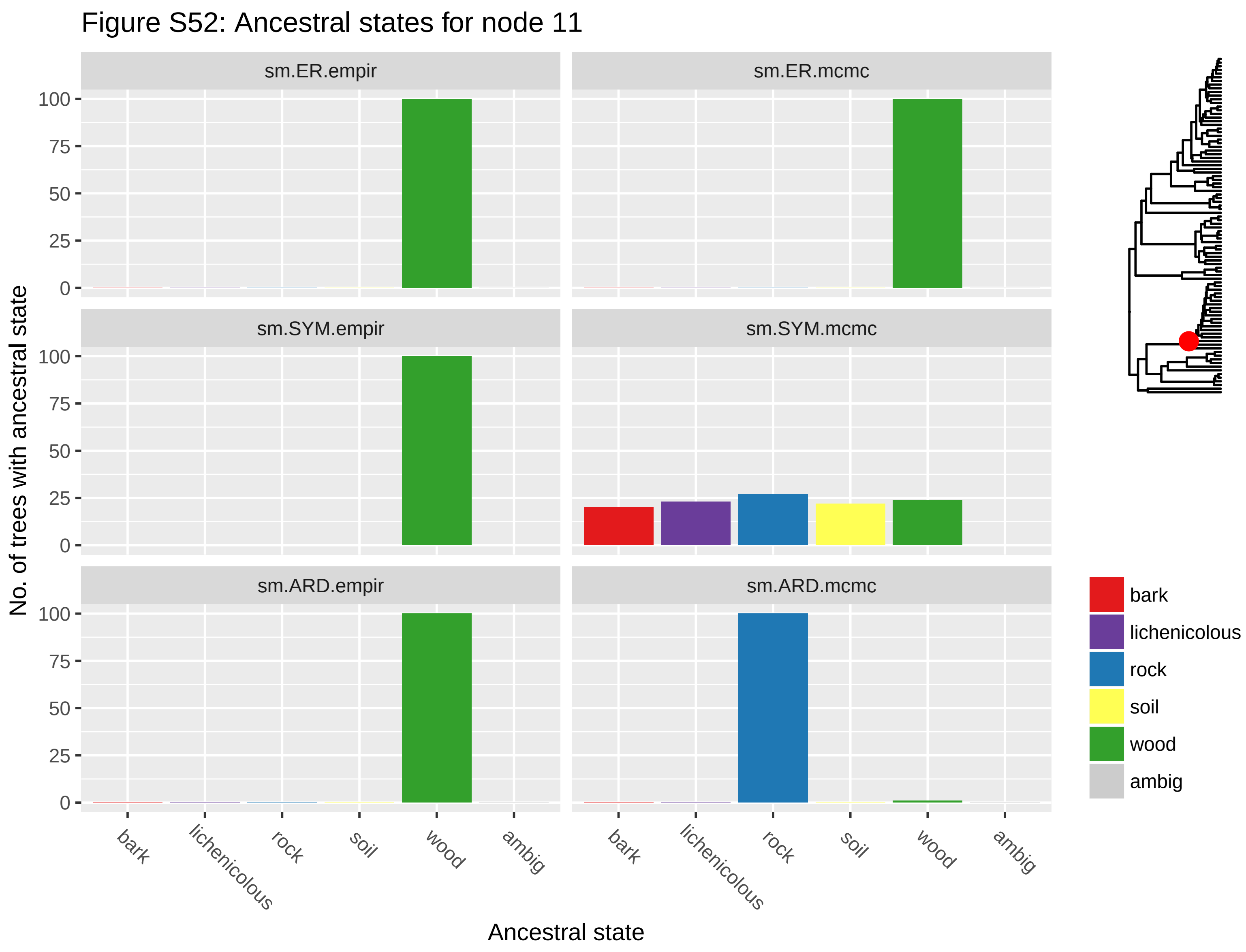

Figure S52: Ancestral states for node 11
sm.ER.empir
sm.ER.mcmc
sm.SYM.empir
sm.SYM.mcmc
sm.ARD.empir
sm.ARD.mcmc
No. of trees with ancestral state
100
75
50
25
0
bark
lichenicolous
rock
soil
wood
ambig
Ancestral state

Figure S53: Ancestral states for node 12

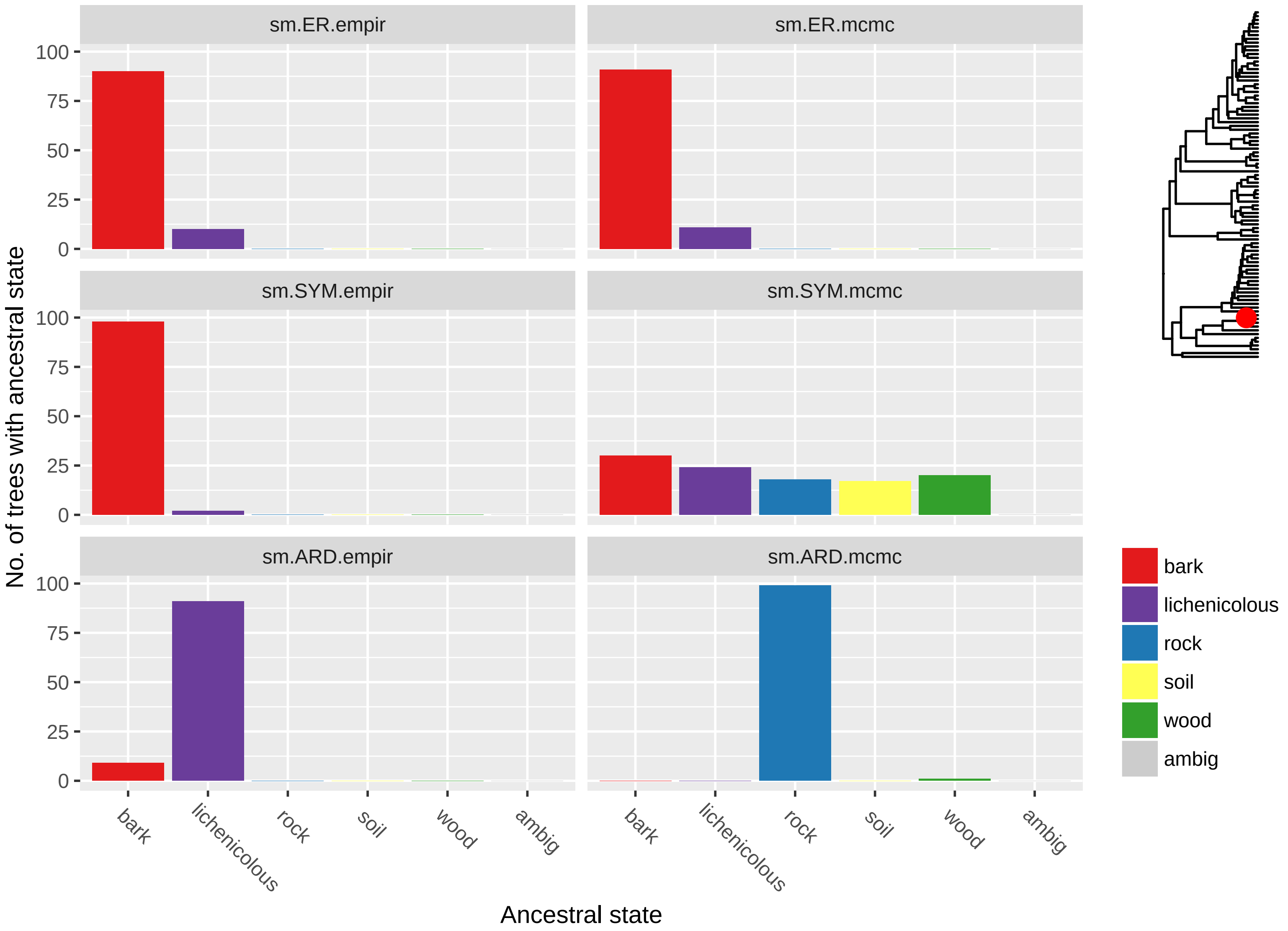

Figure S54: Ancestral states for node 13

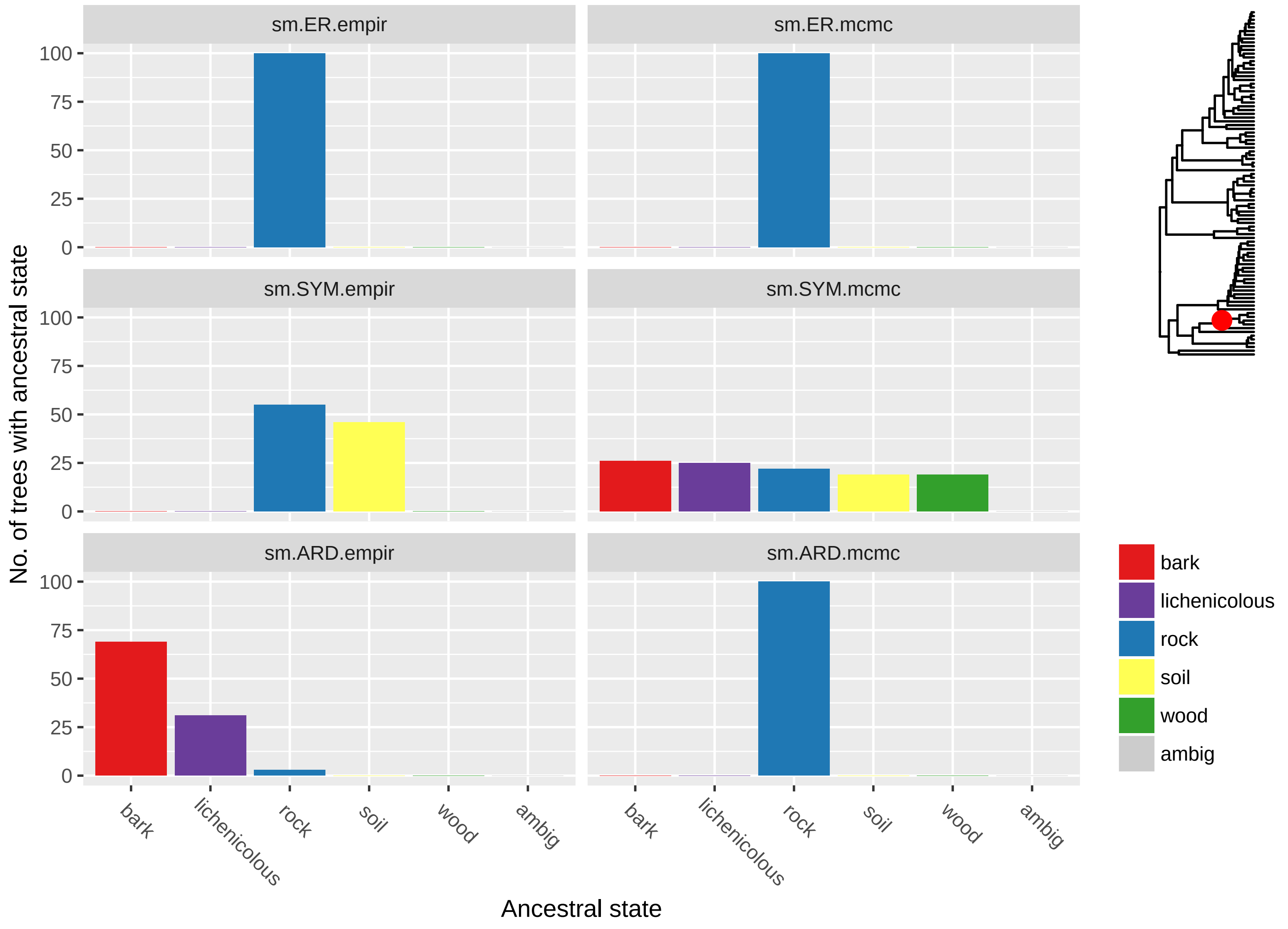

Figure S55: Ancestral states for node 14

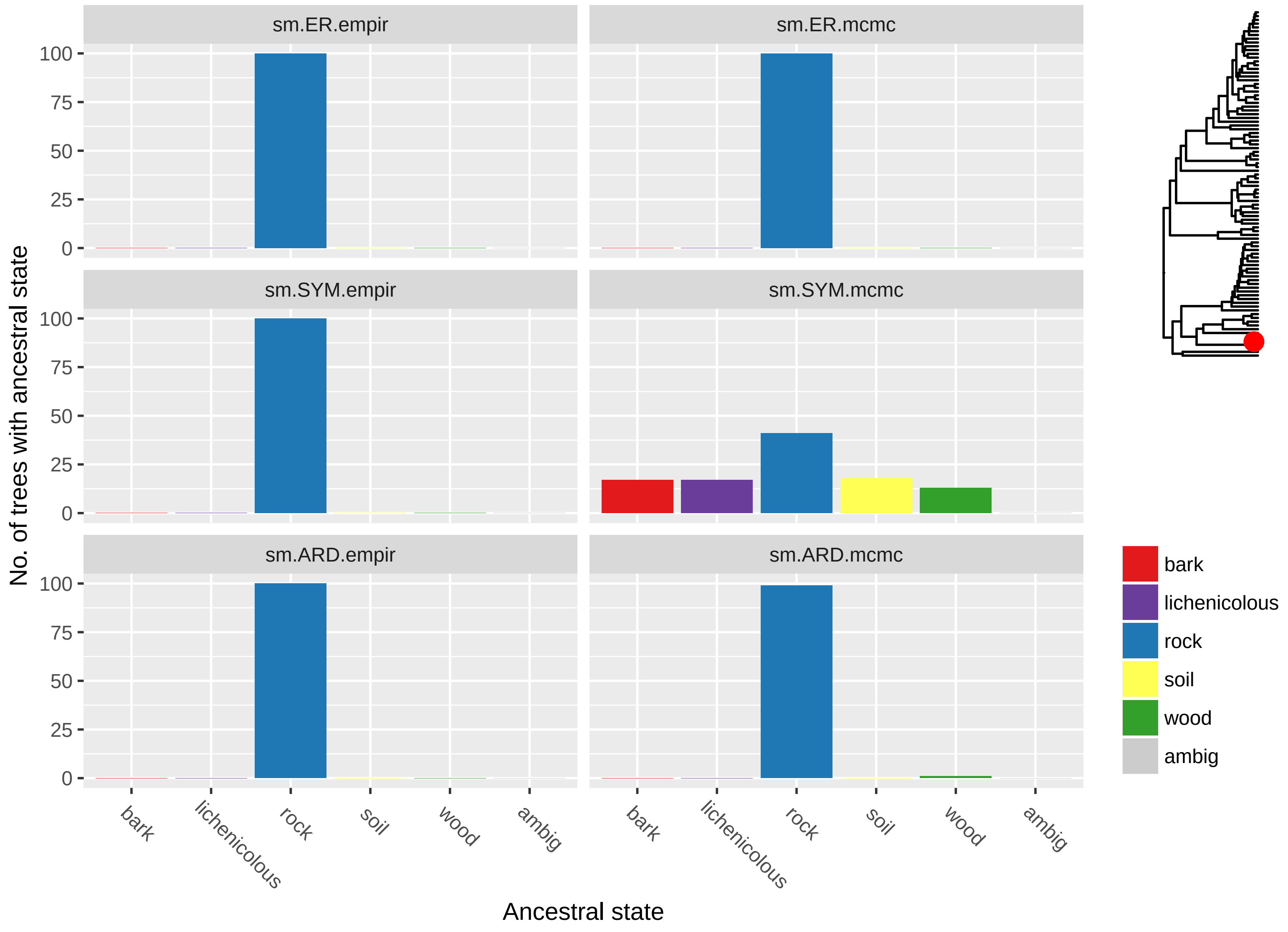

Figure S56: Ancestral states for node 15

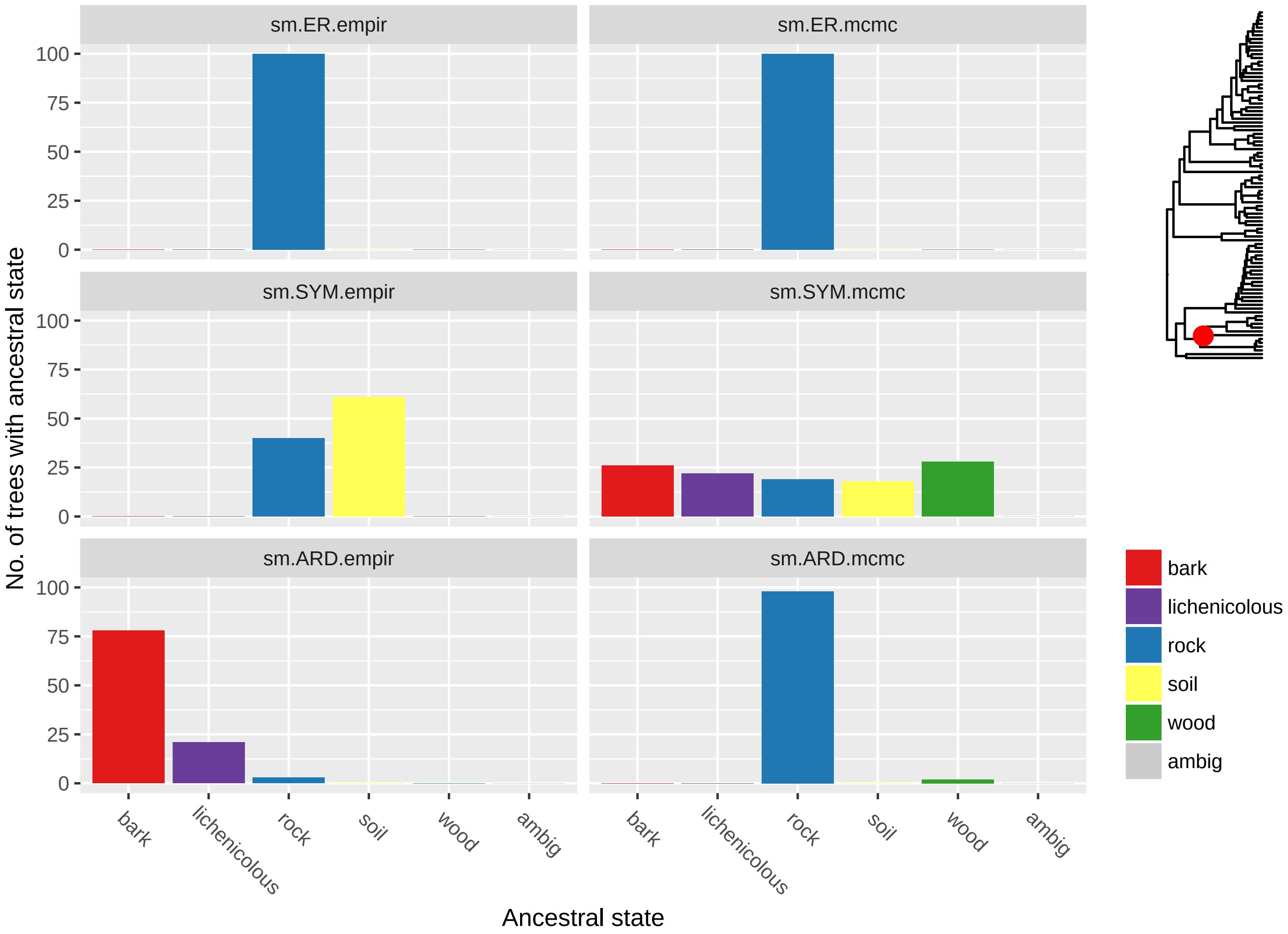

Figure S57: Ancestral states for node 16

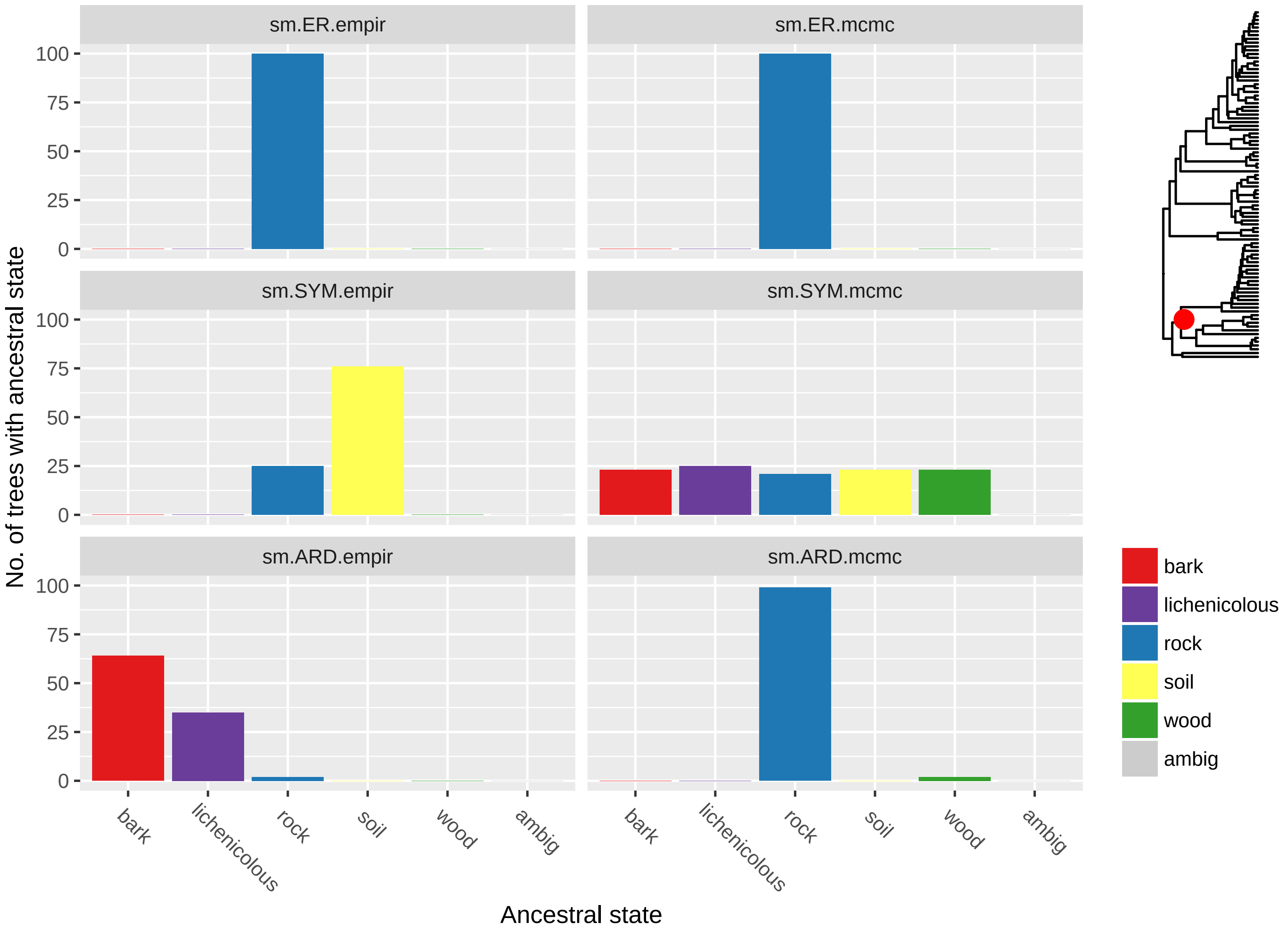

Figure S58: Ancestral states for node 17

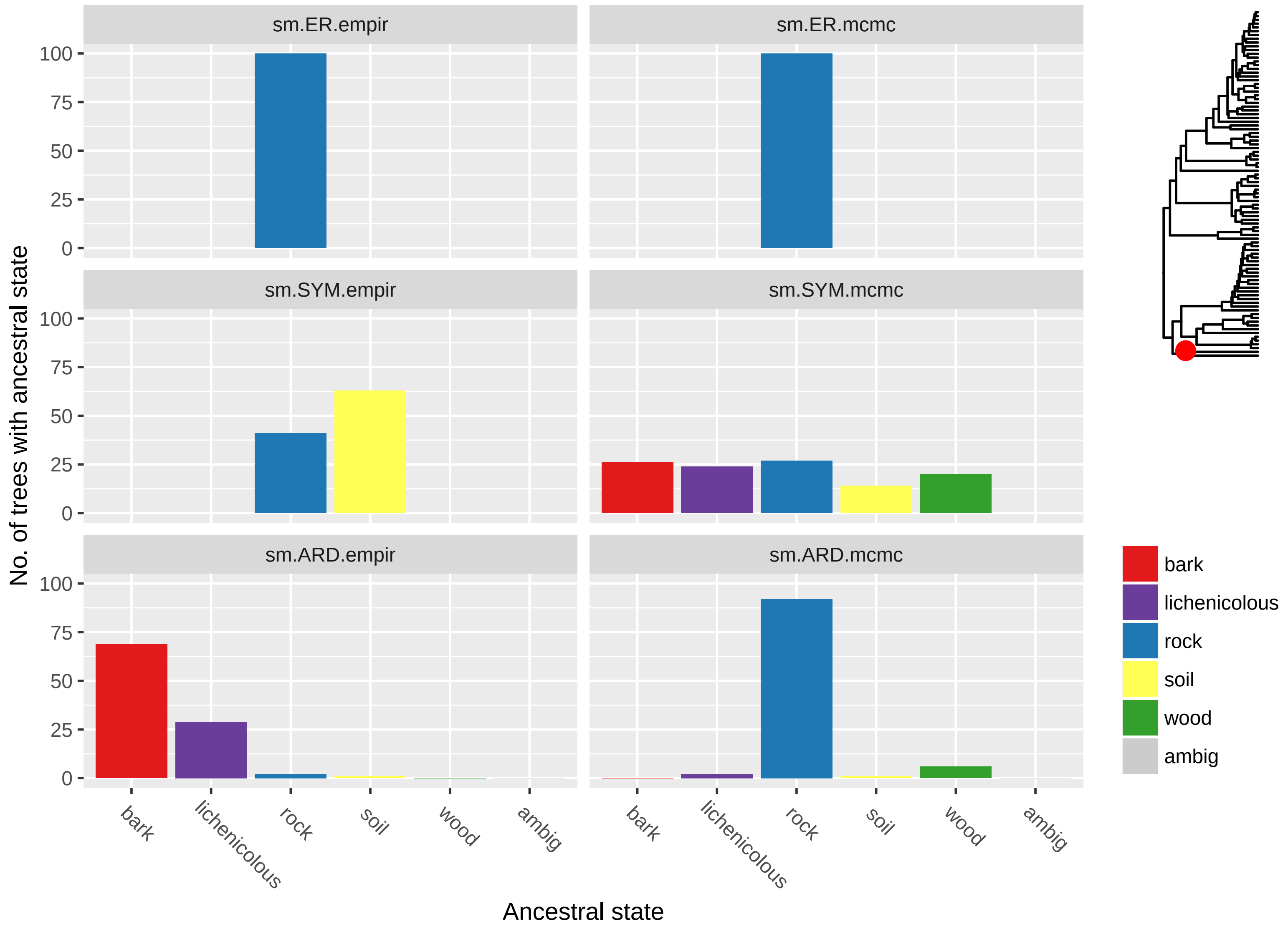

Figure S59: Ancestral states for node 18

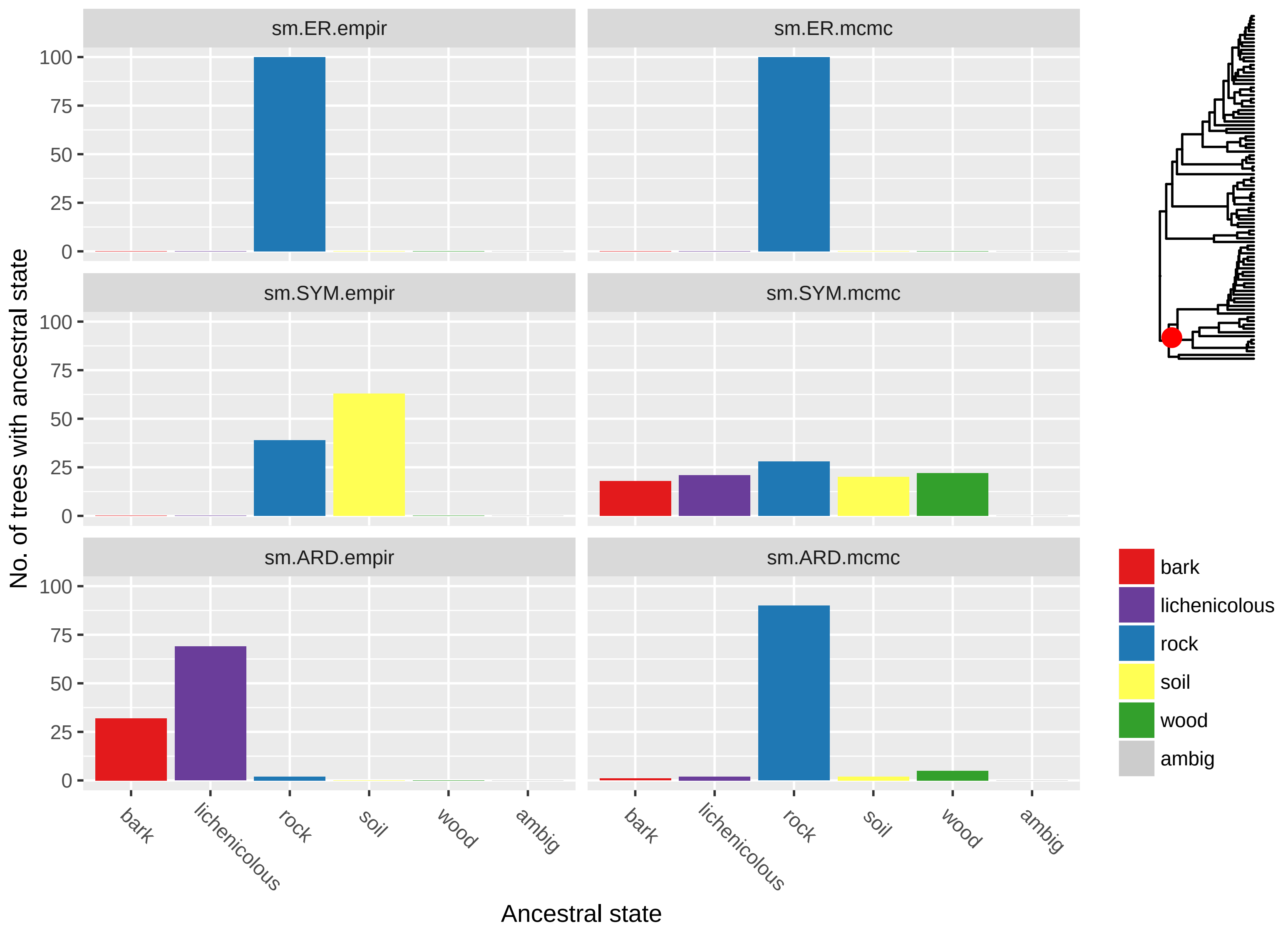

Figure S60: Ancestral states for node 19

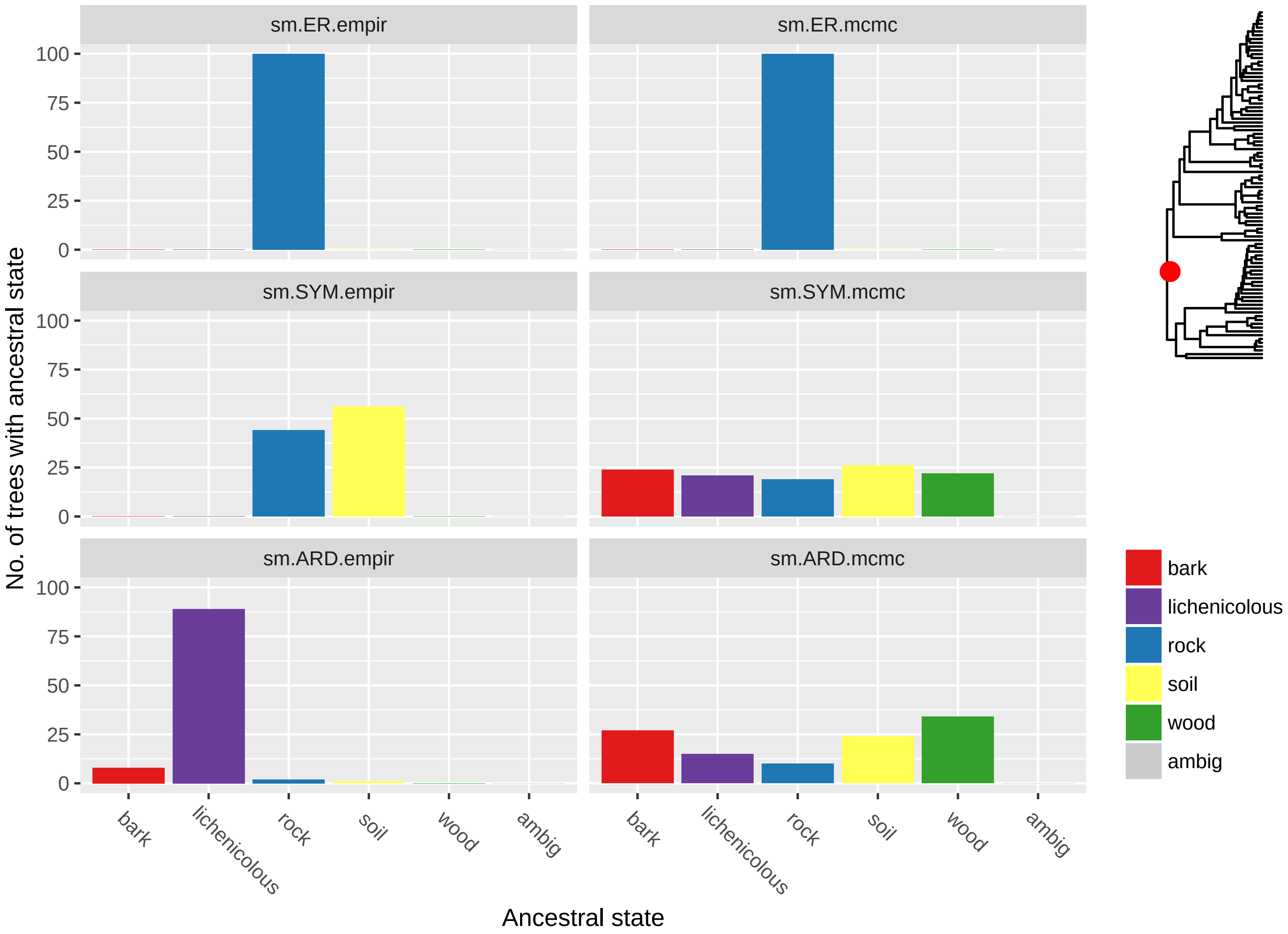

Figure S61-S79 - Results of ancestral state estimation of ecological strategy character with ace for 19 nodes of the trapelioid phylogeny

Ancestral state estimations of the ecological strategy character for 19 nodes of the trapelioid phylogeny based on the method implemented in the ace function in R package ape imposing 4 different models. Please refer to the main text for details.

Figure S61: Ancestral states for node 1

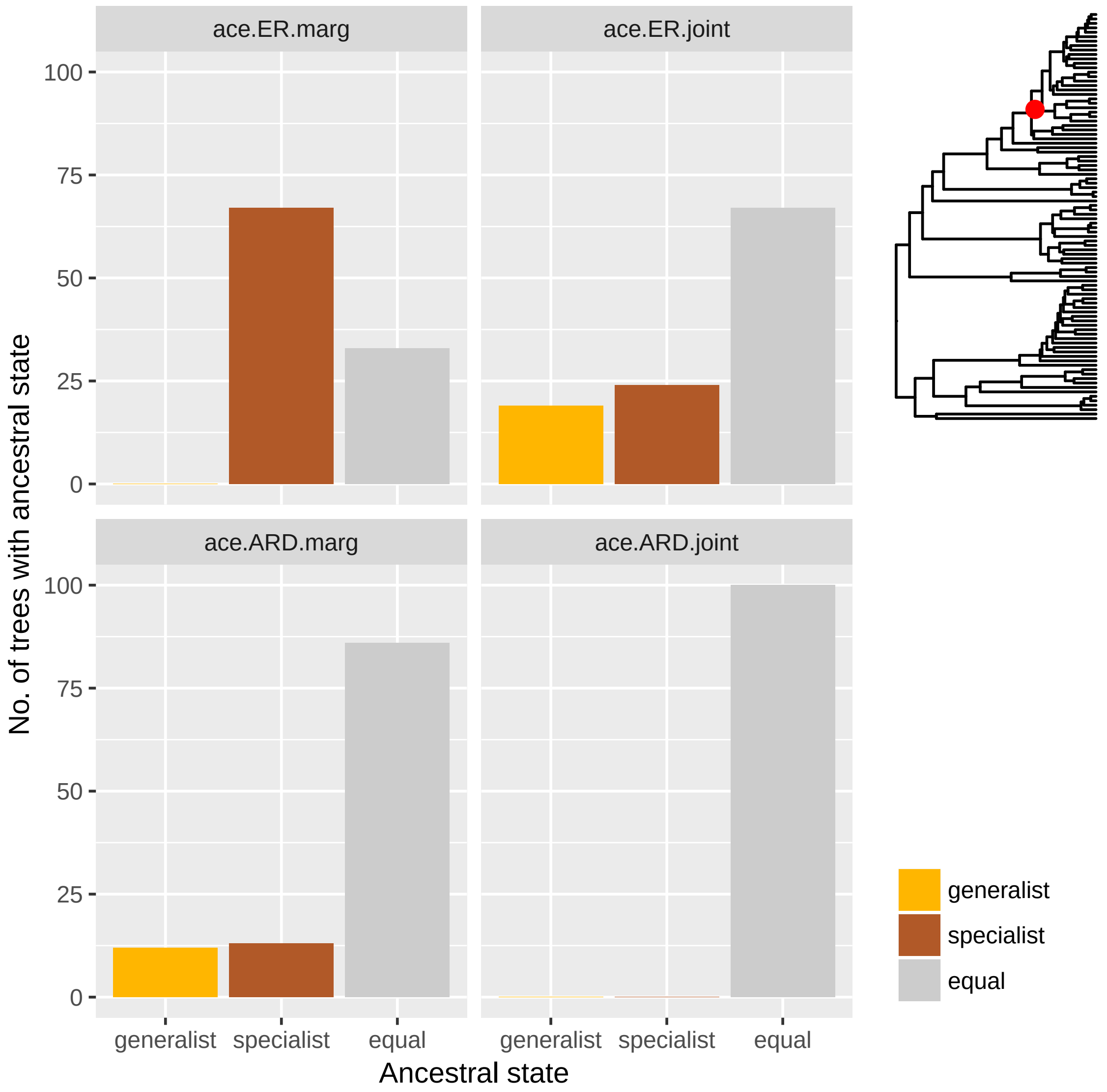

Figure S62: Ancestral states for node 2

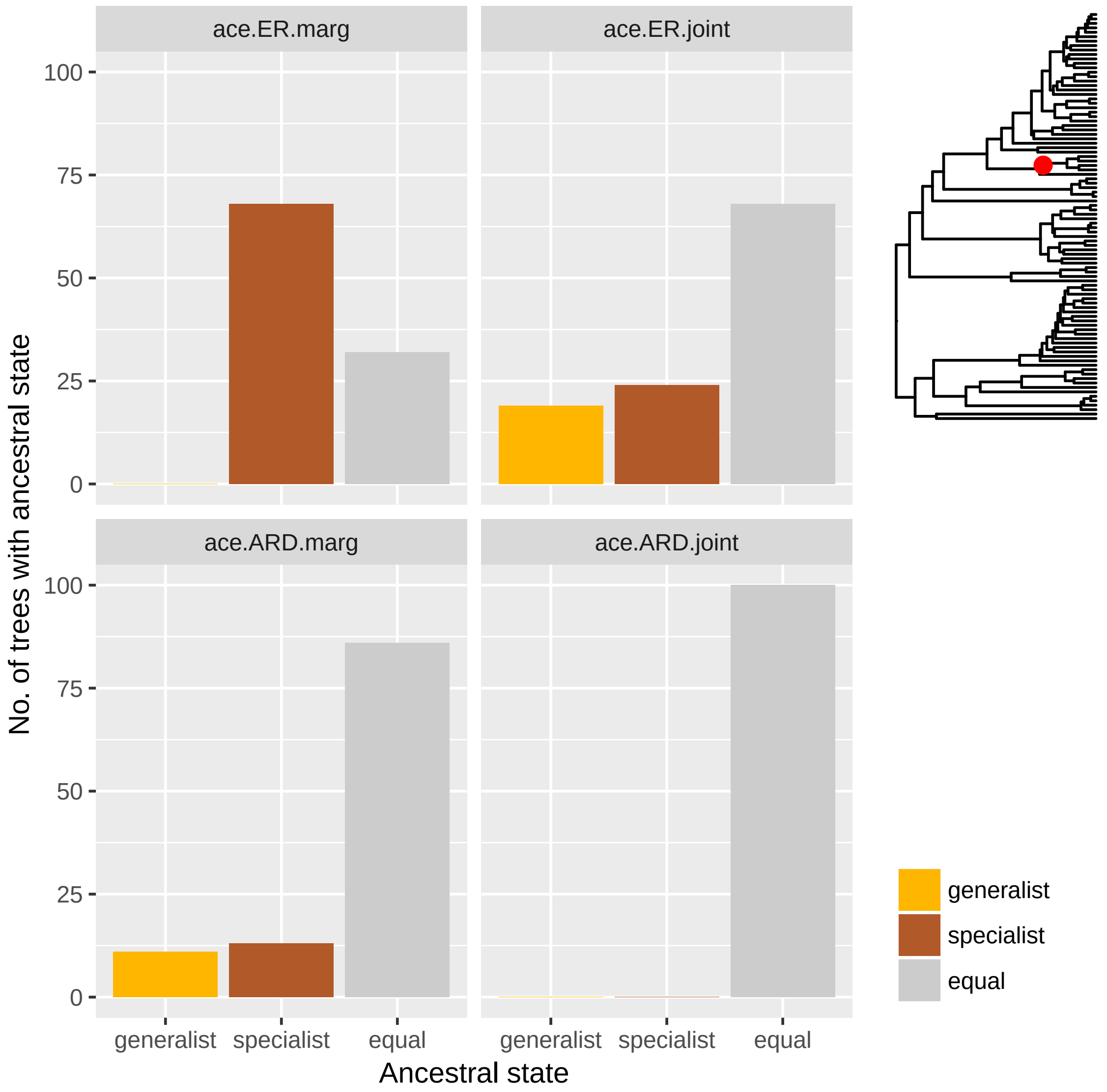

Figure S63: Ancestral states for node 3

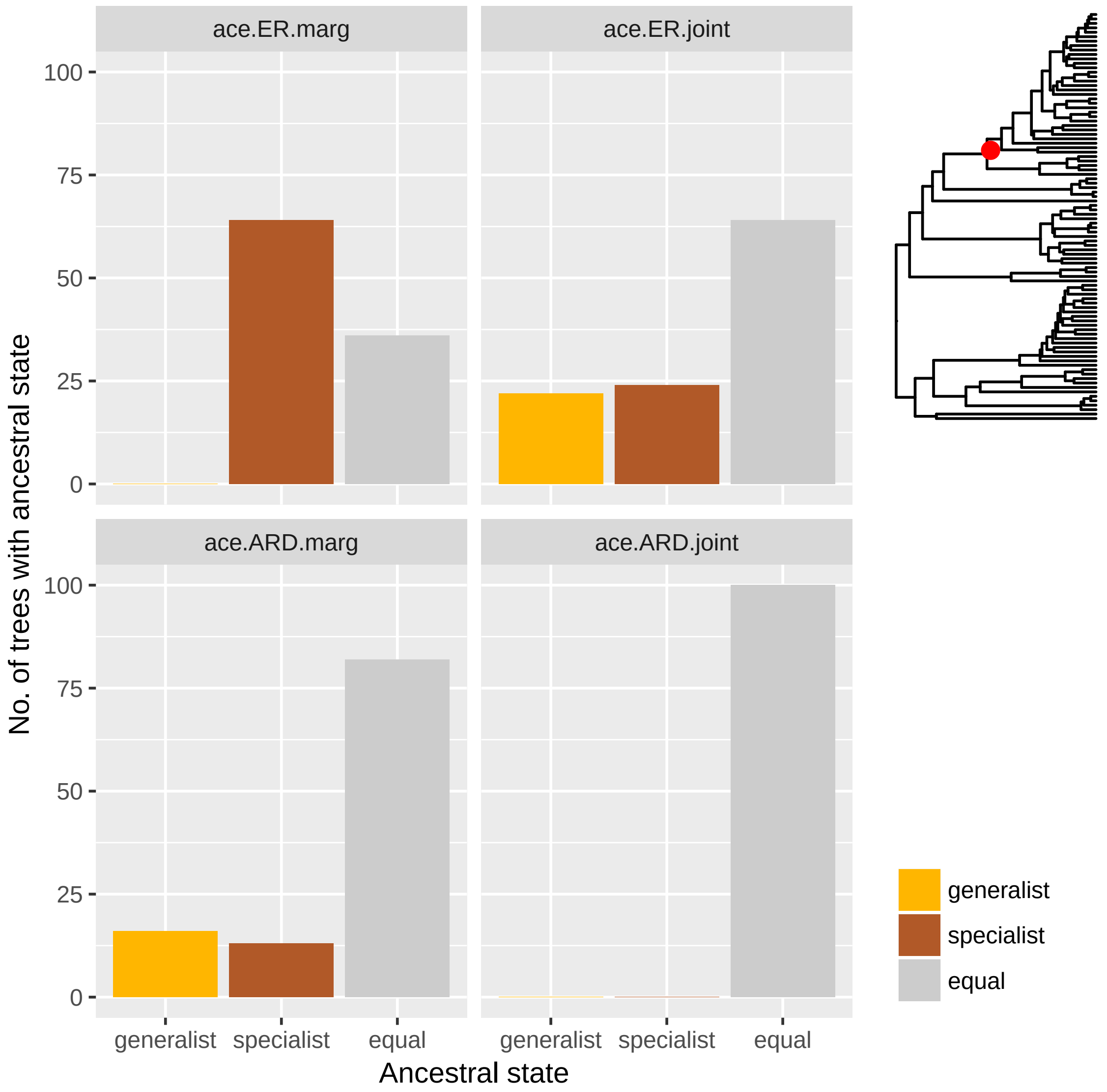

Figure S64: Ancestral states for node 4

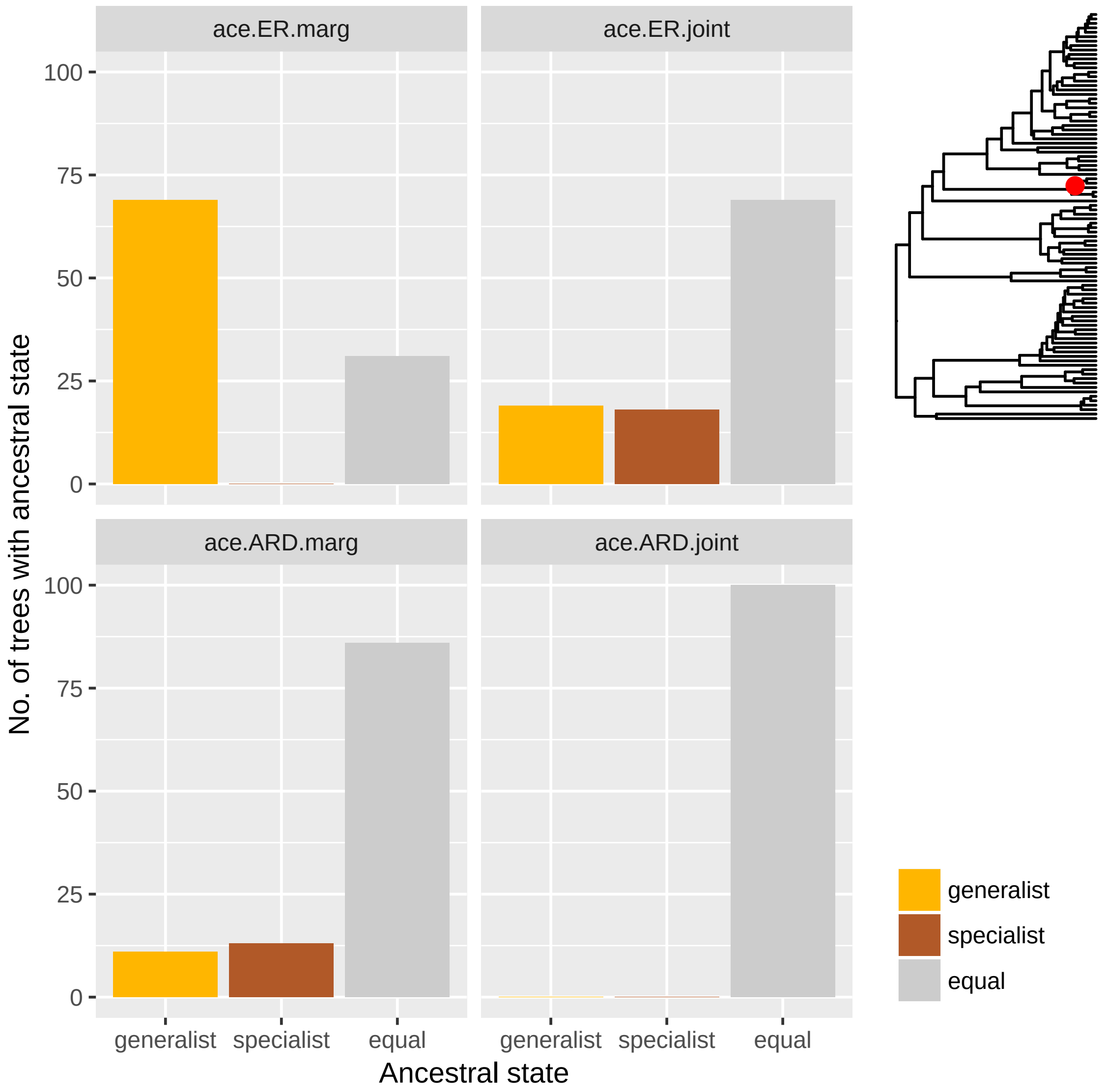

Figure S65: Ancestral states for node 5

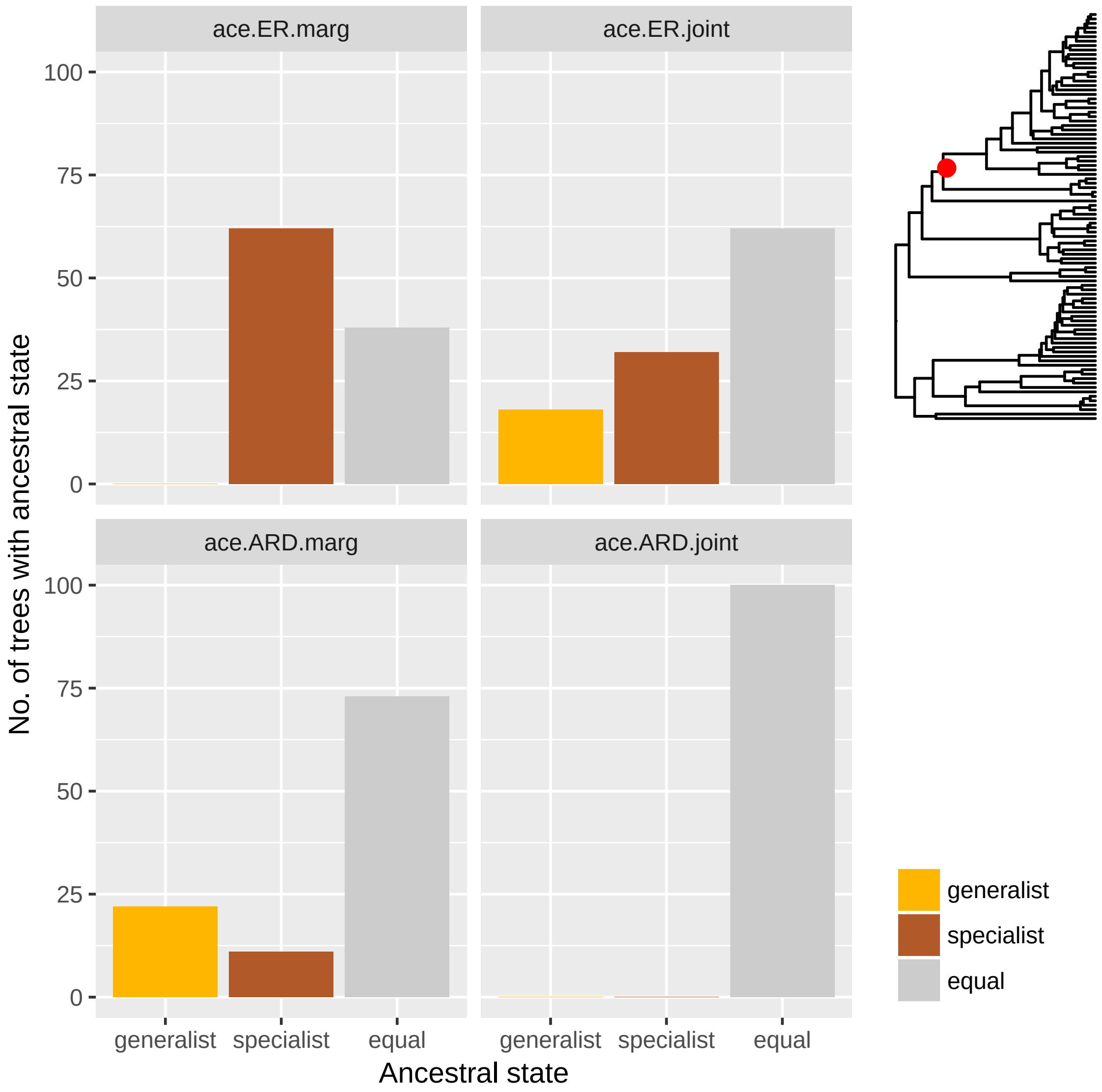

Figure S66: Ancestral states for node 6

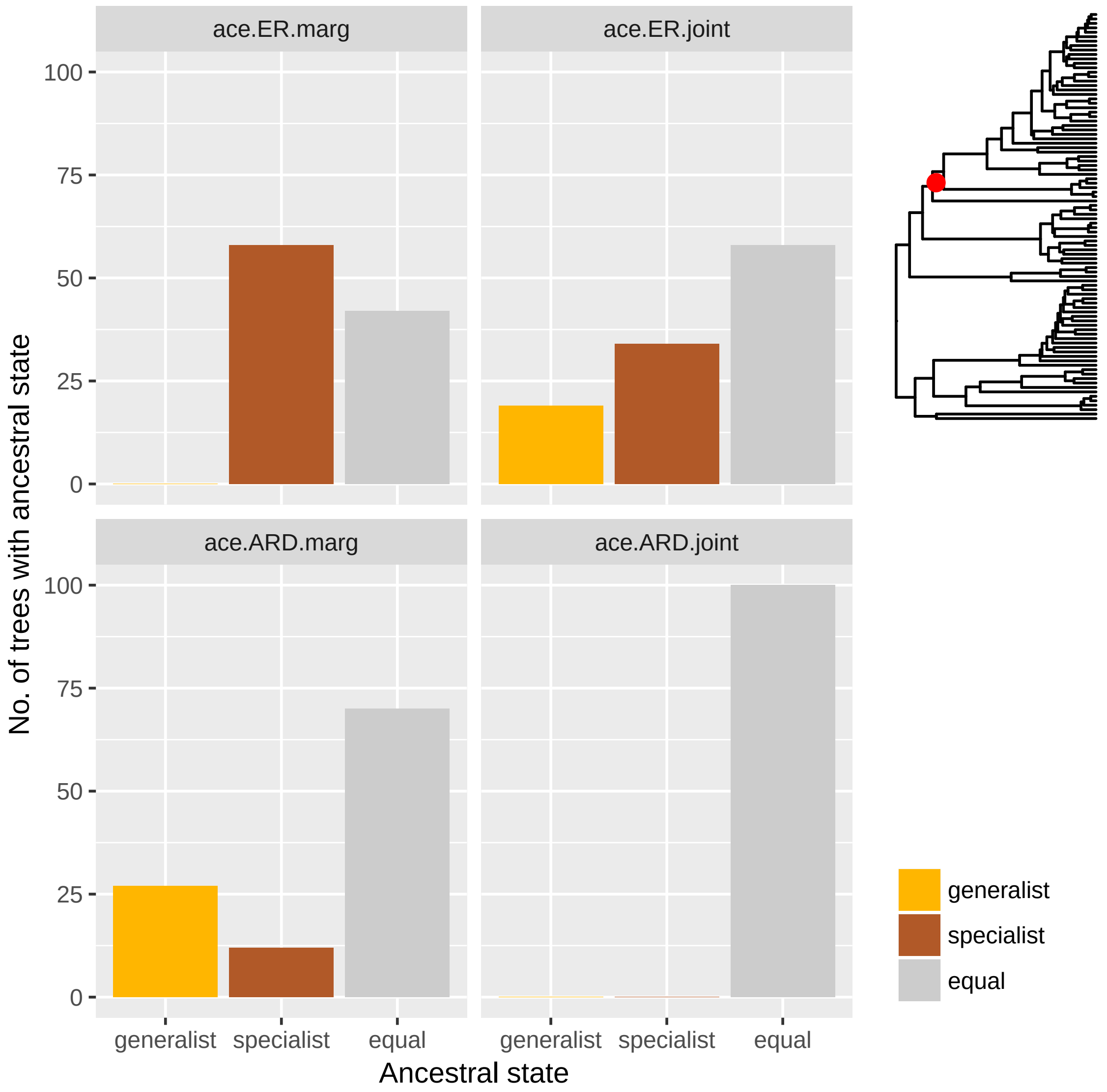

Figure S67: Ancestral states for node 7

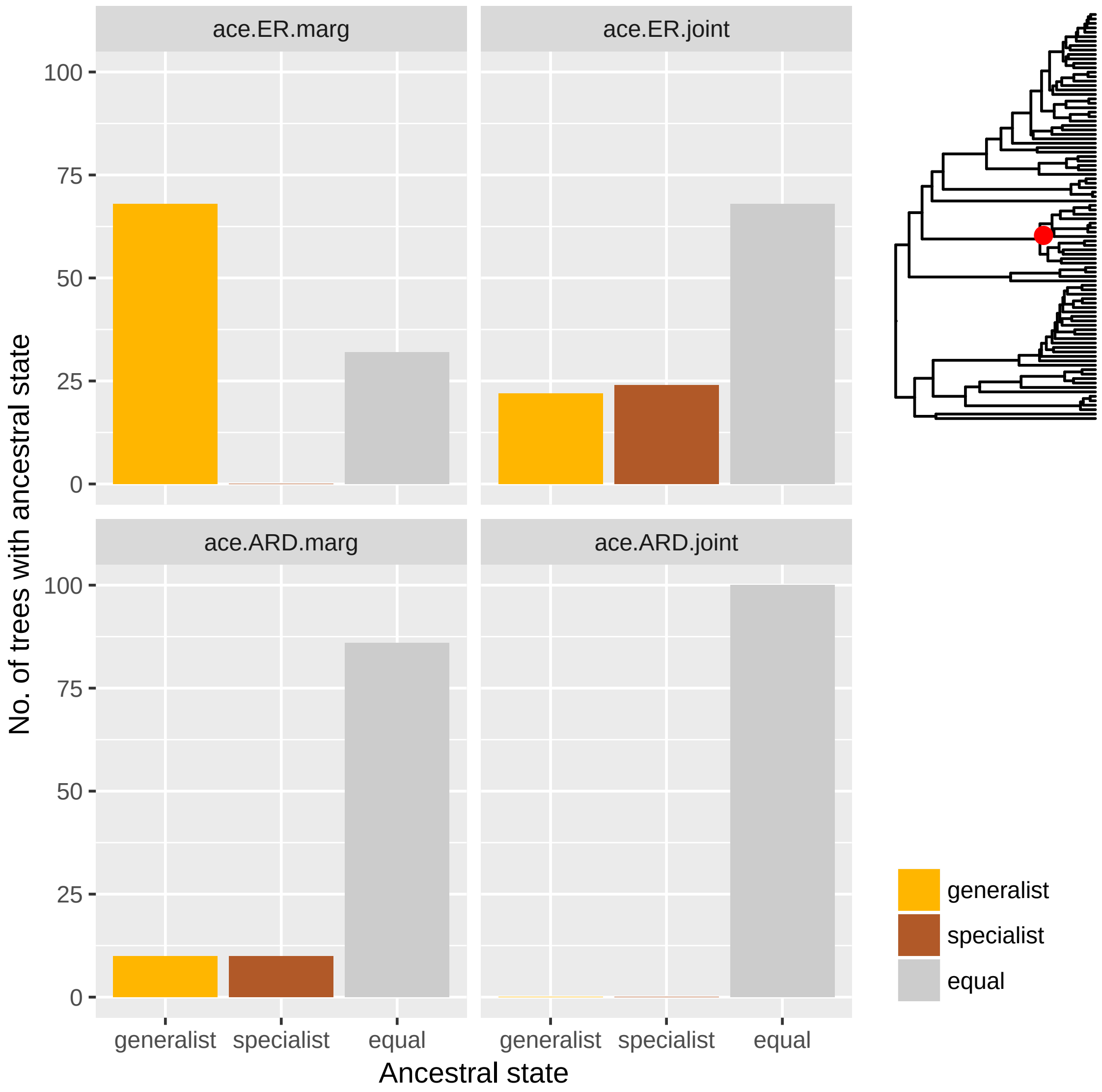

Figure S68: Ancestral states for node 8

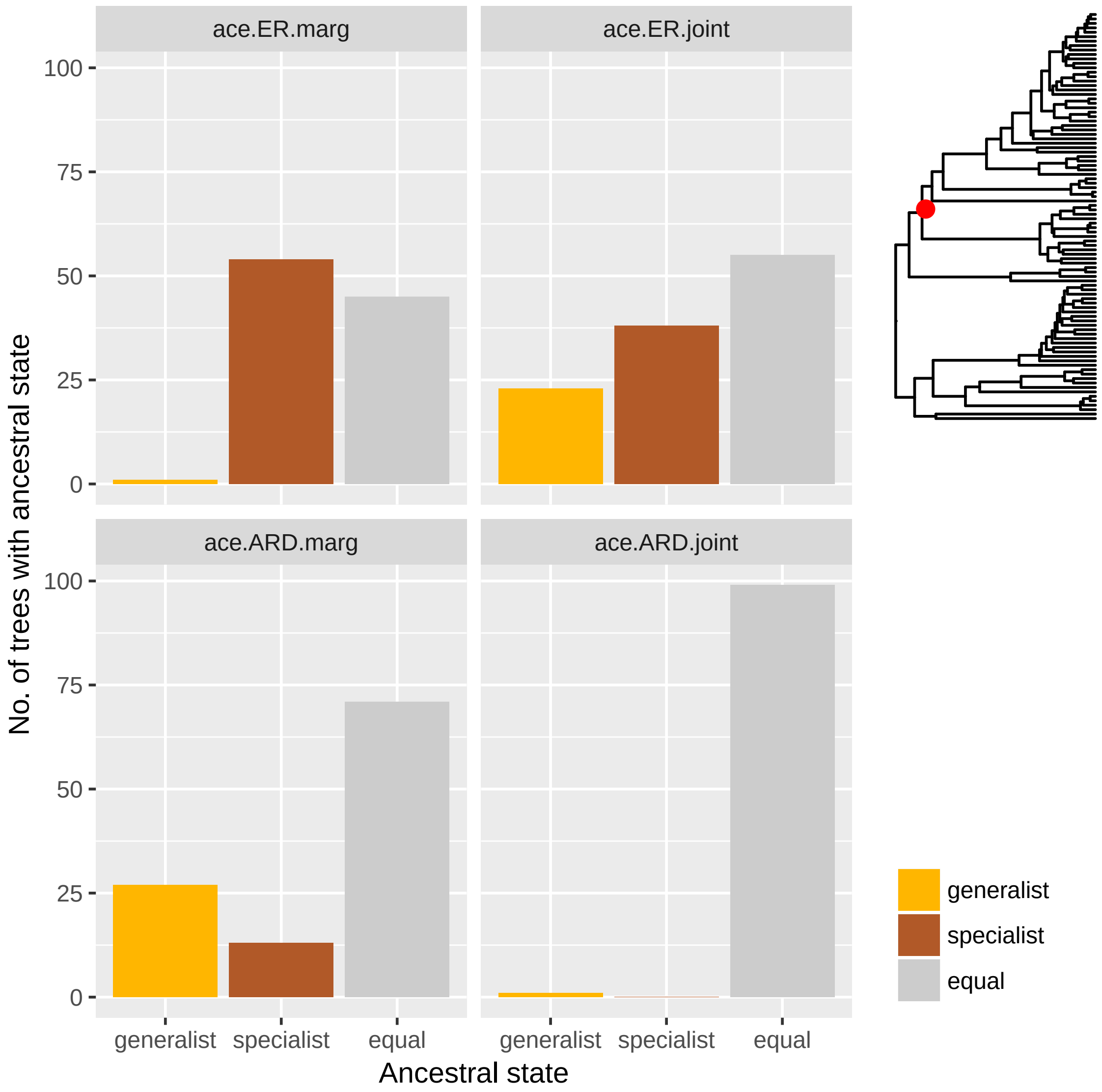

Figure S69: Ancestral states for node 9

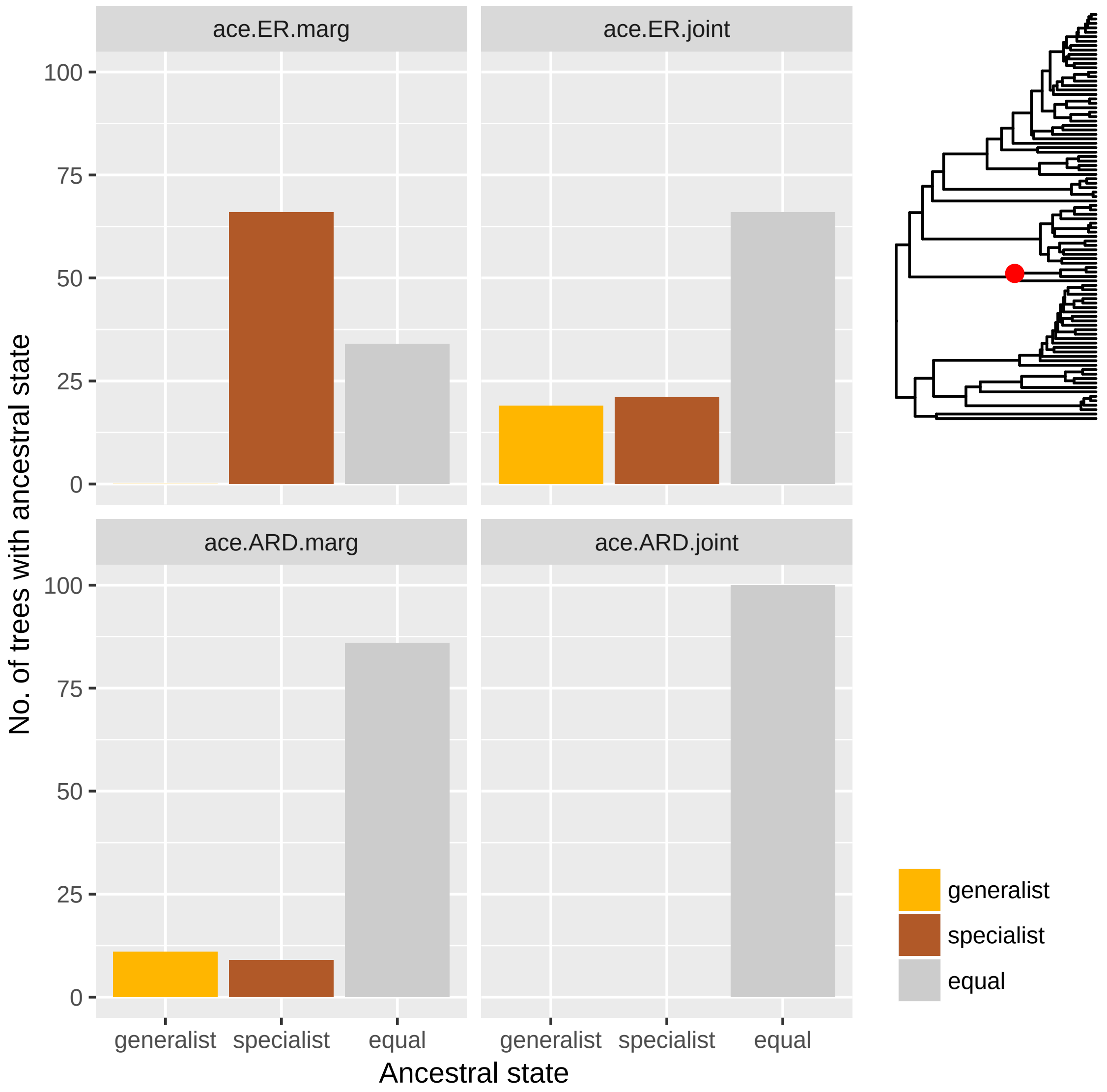

Figure S70: Ancestral states for node 10

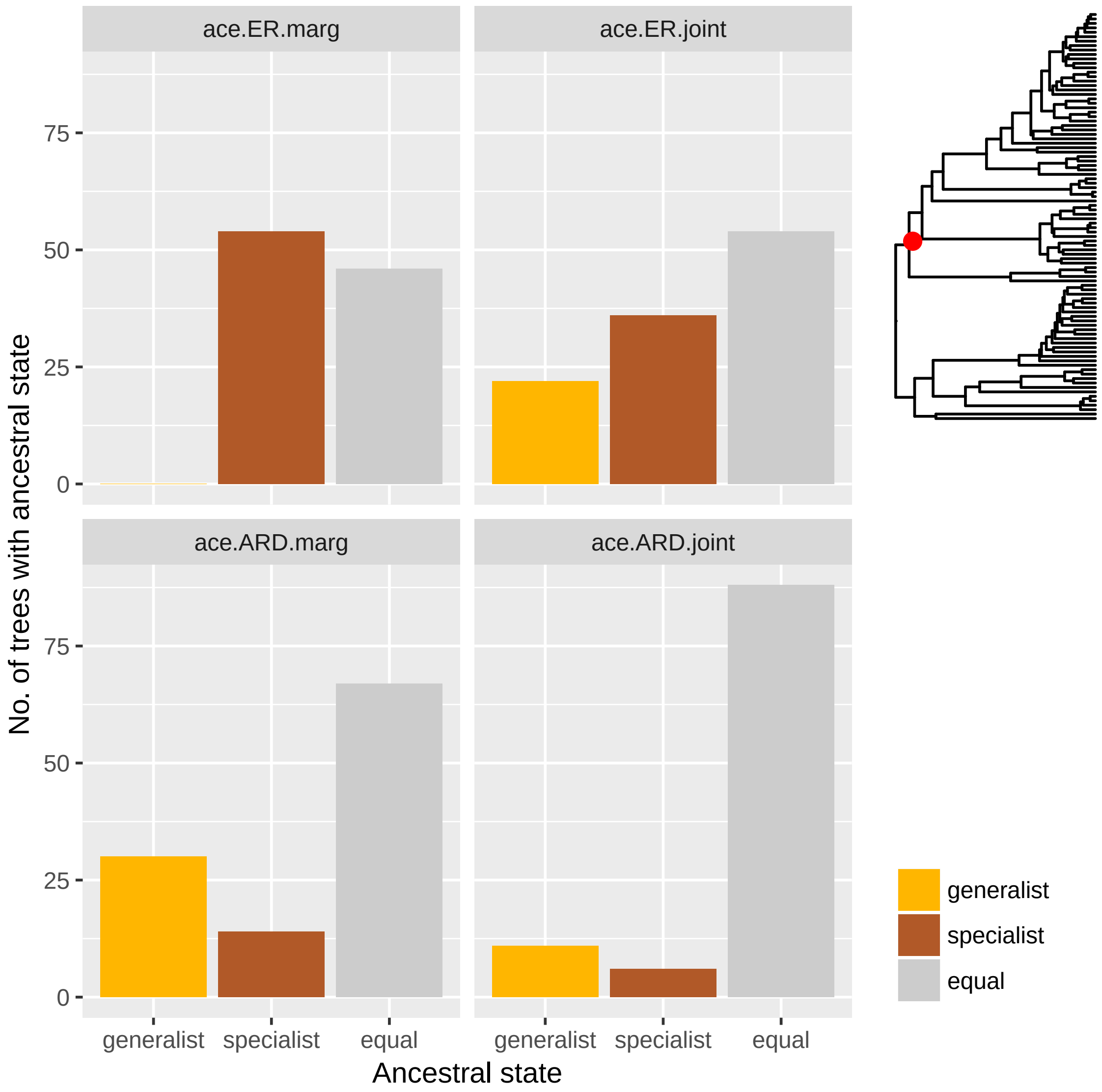

Figure S71: Ancestral states for node 11

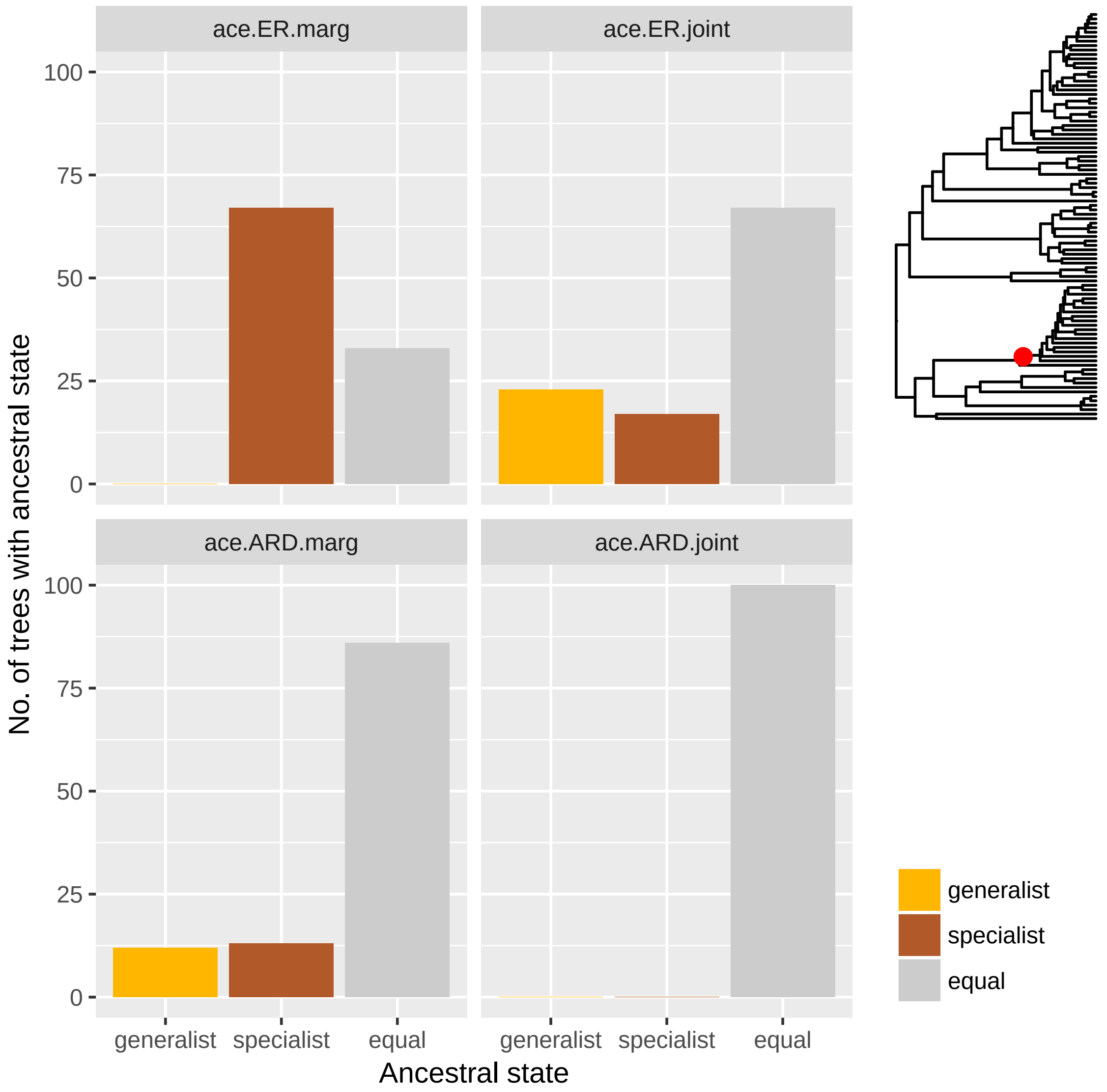

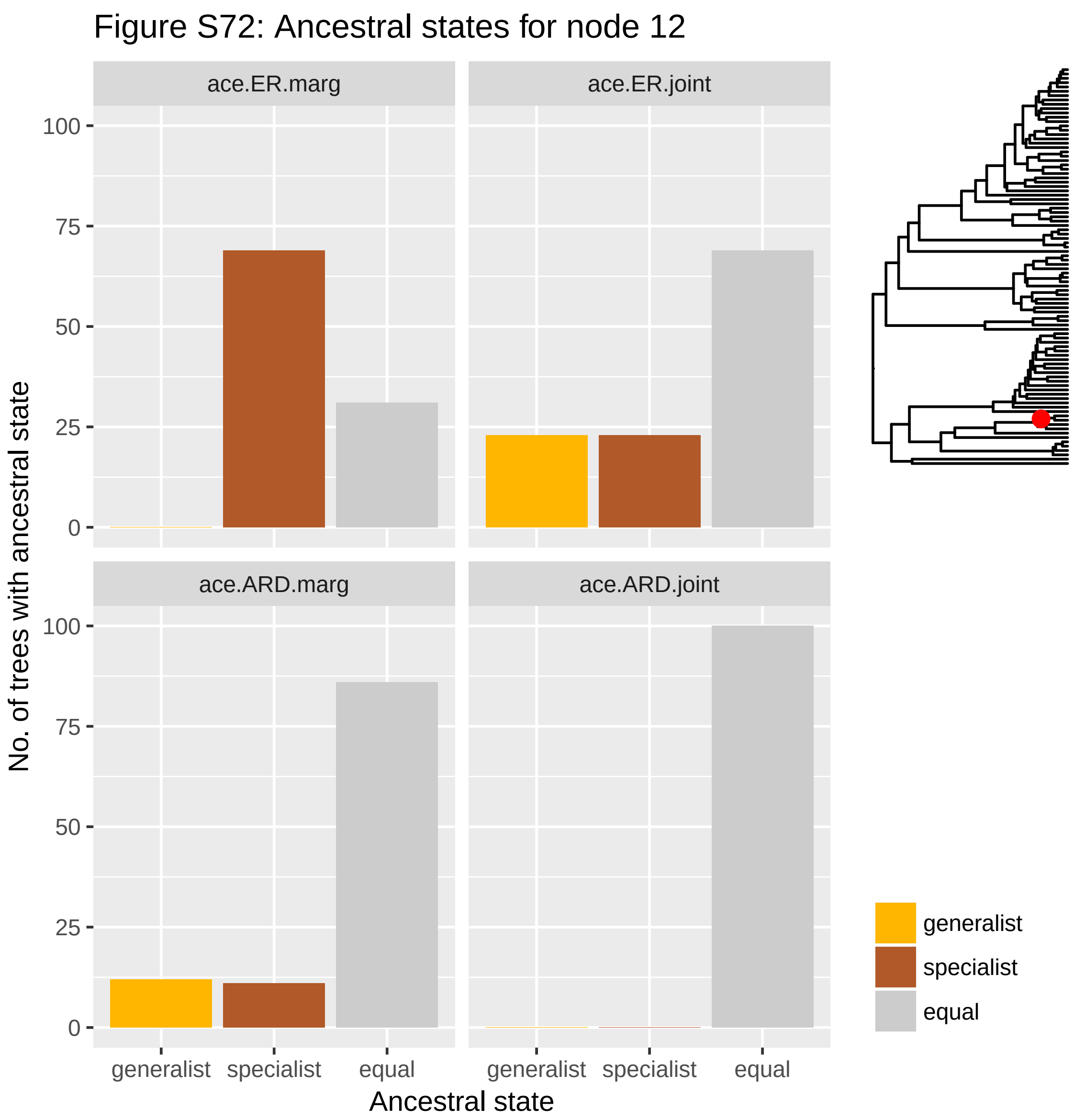

Figure S72: Ancestral states for node 12
ace.ER.marg
ace.ER.joint
ace.ARD.marg
ace.ARD.joint
100
75
50
25
0
No. of trees with ancestral state
generalist
specialist
equal
Ancestral state

Figure S73: Ancestral states for node 13

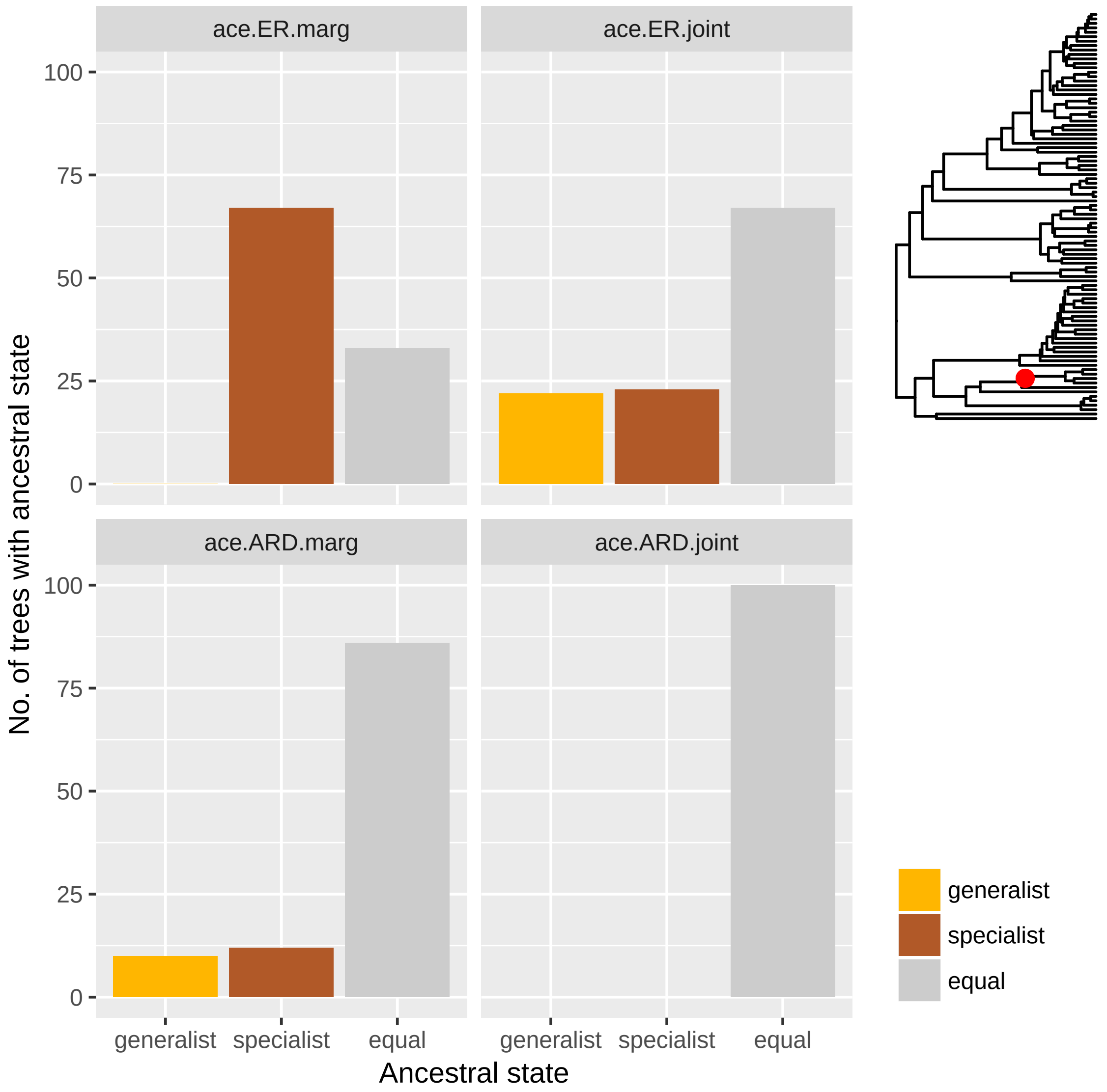

Figure S74: Ancestral states for node 14

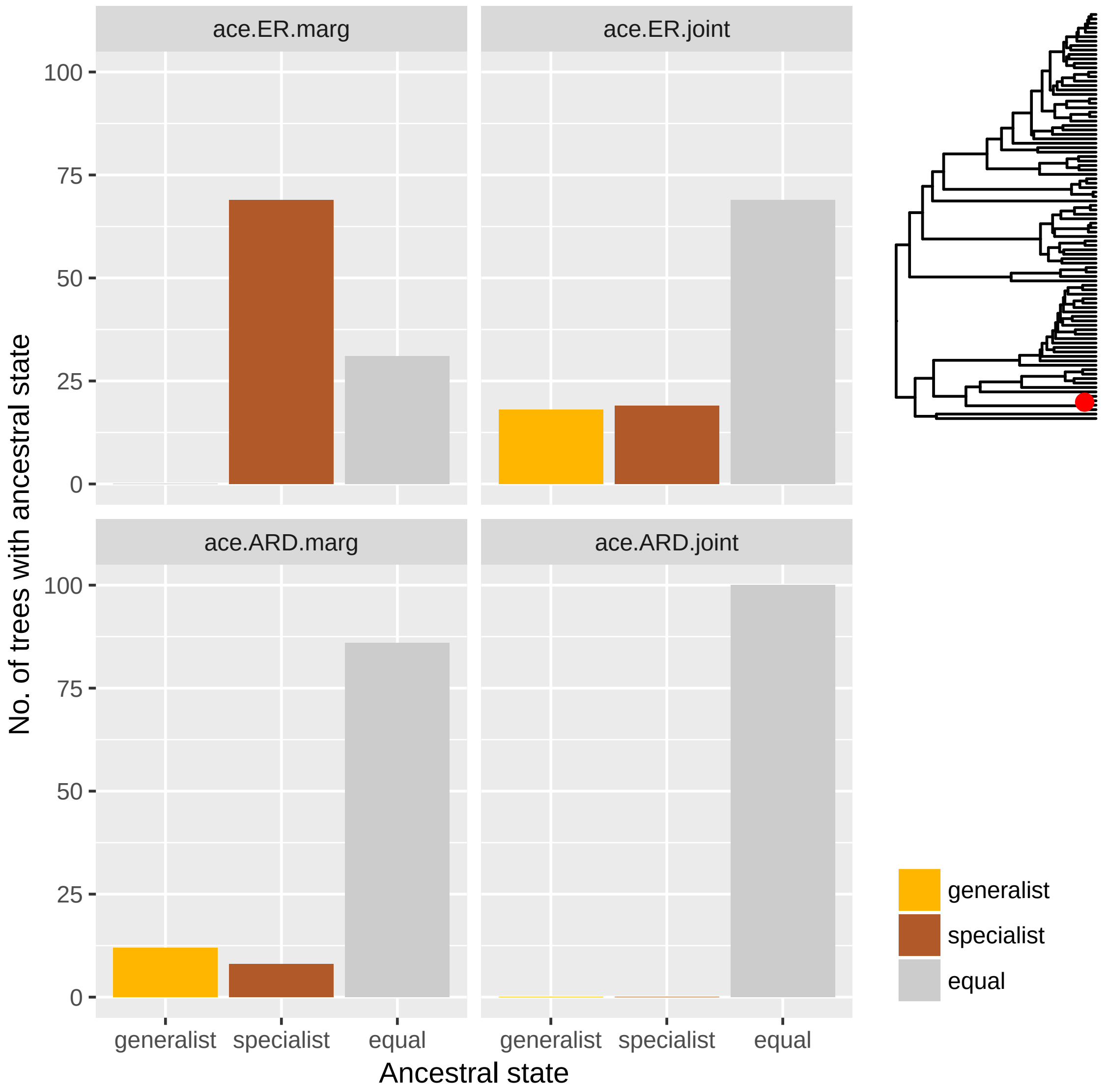

Figure S75: Ancestral states for node 15

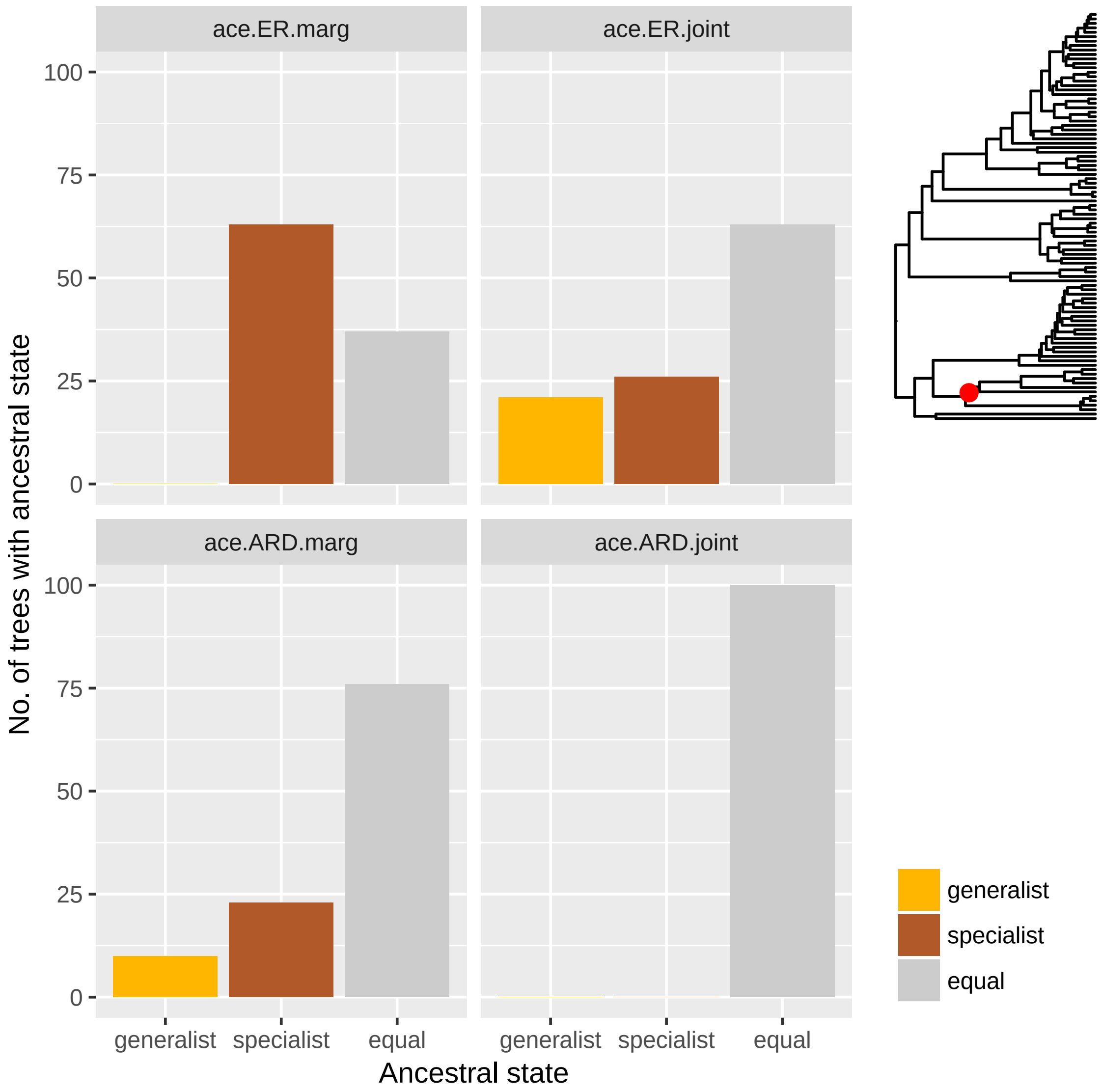

Figure S76: Ancestral states for node 16

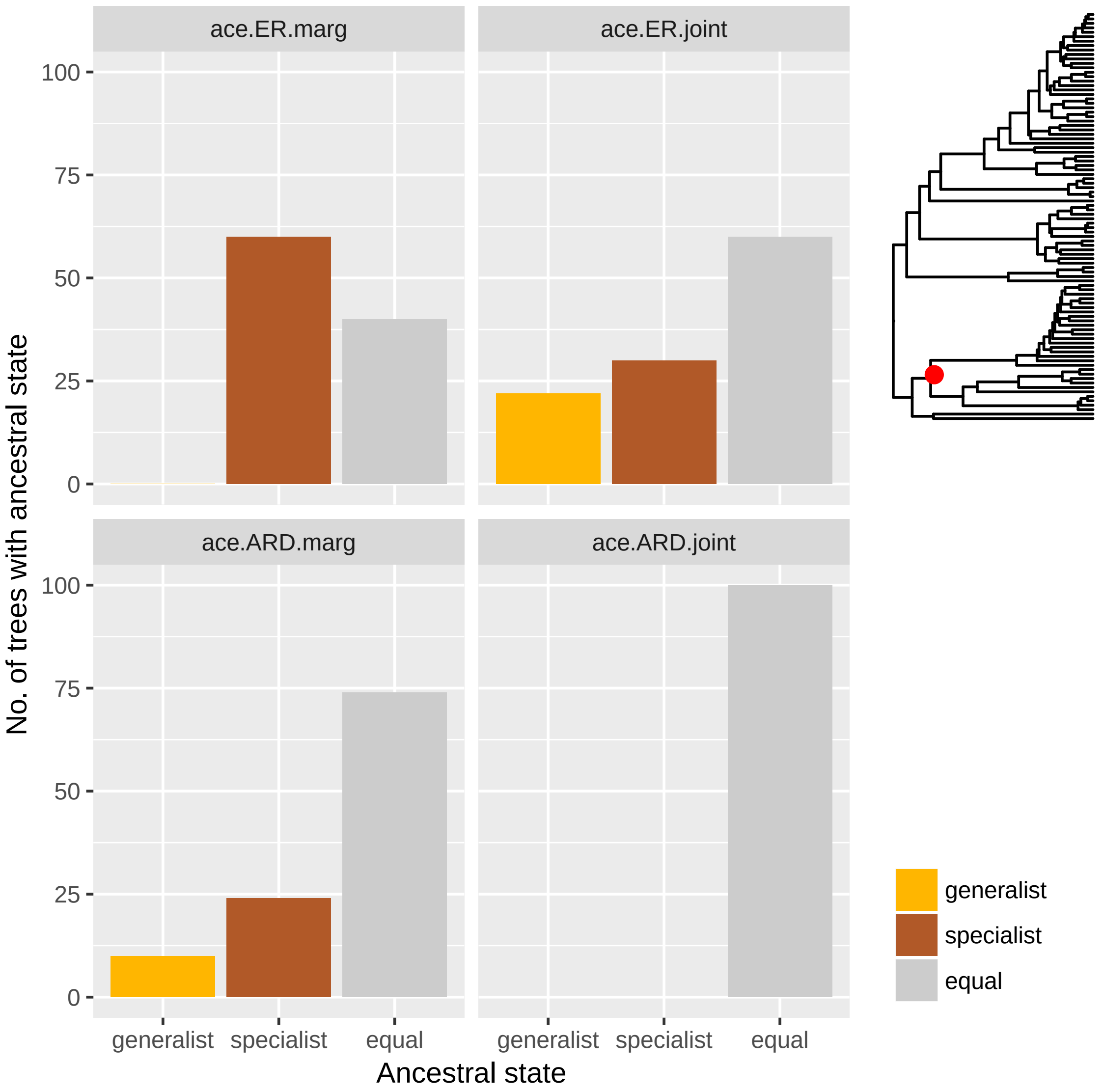

Figure S77: Ancestral states for node 17

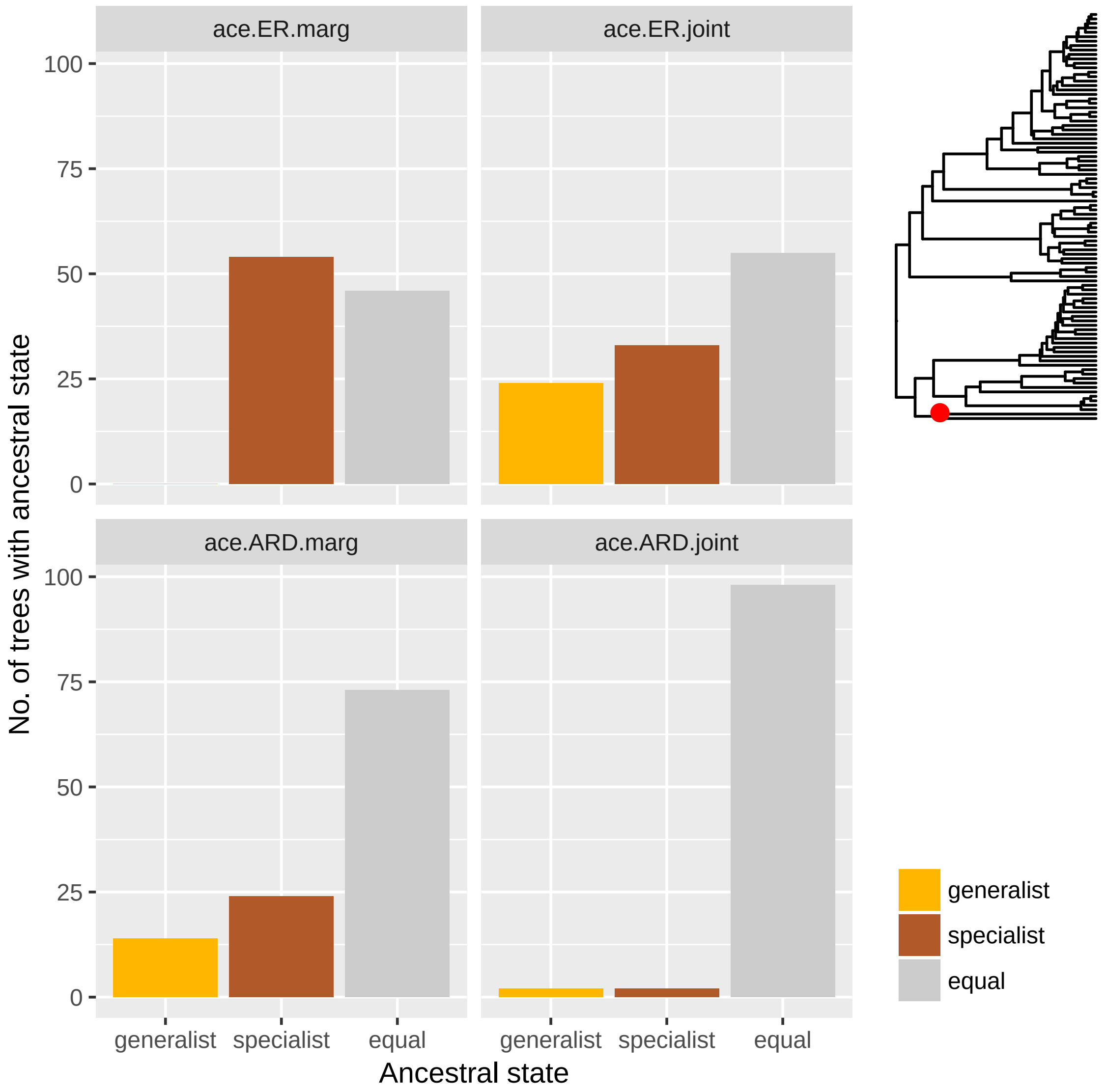

Figure S78: Ancestral states for node 18

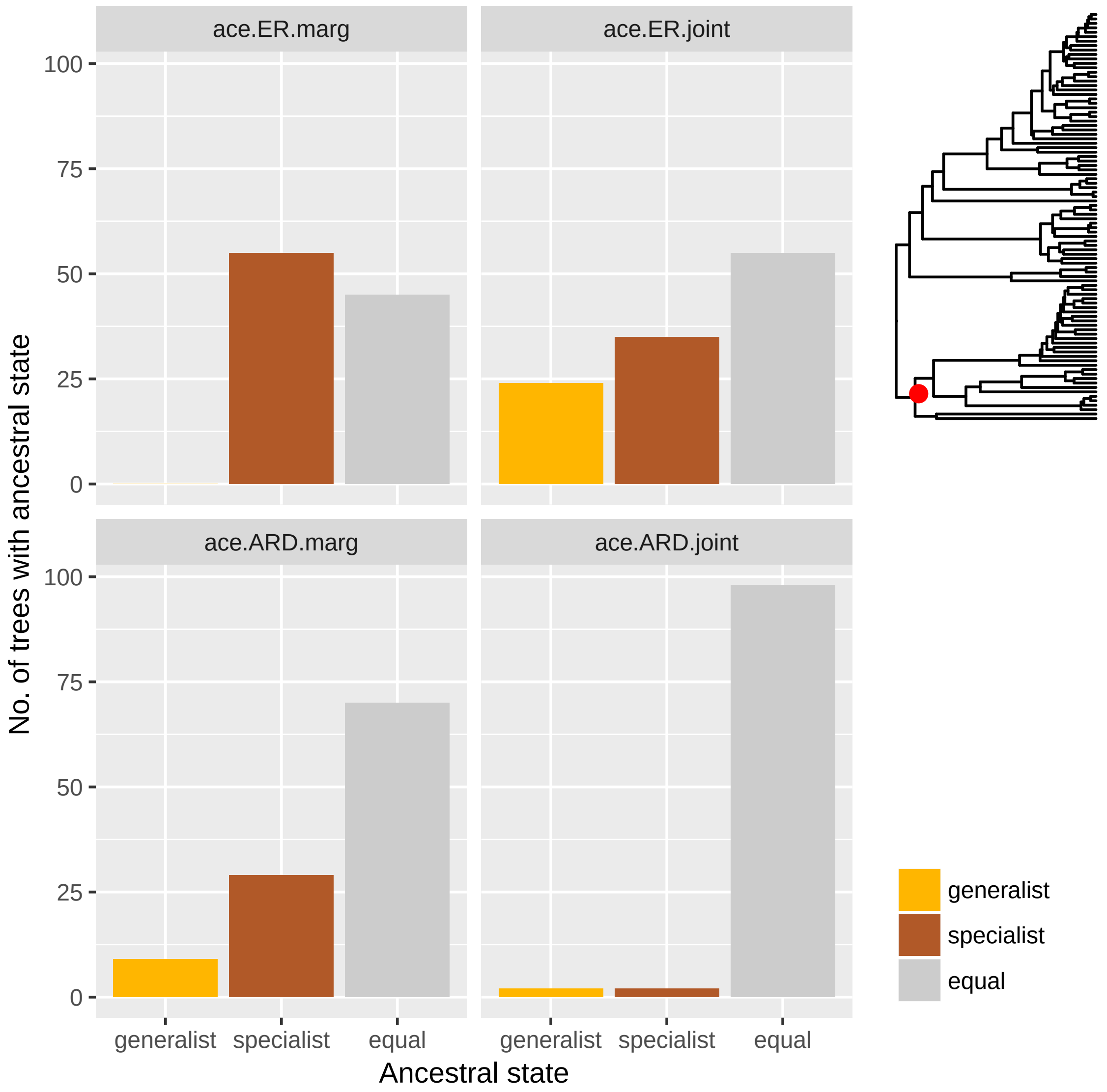

Figure S79: Ancestral states for node 19

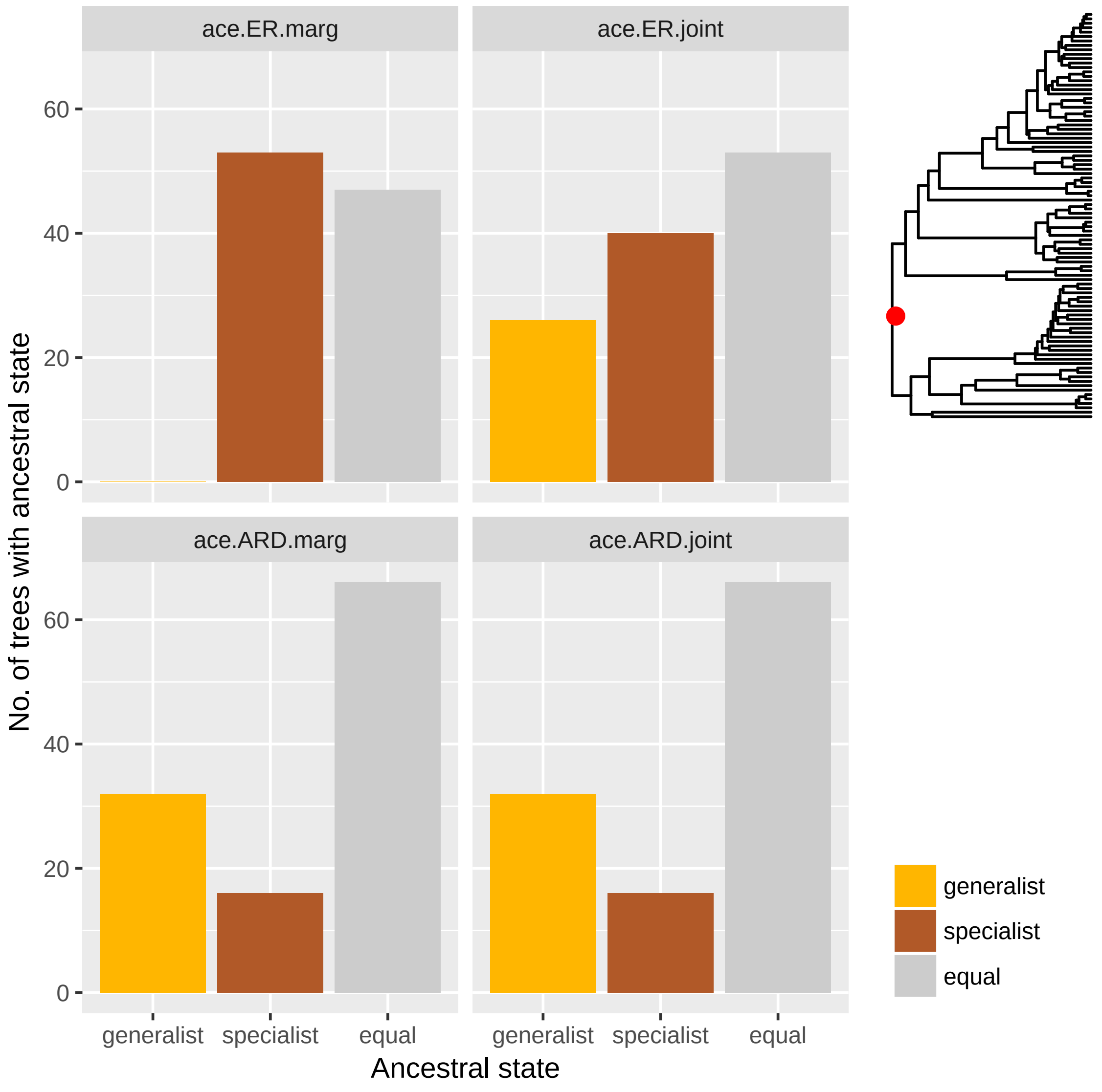

Figure S80-S98 - Results of ancestral state estimation of ecological strategy character with rayDISC for 19 nodes of the trapelioid phylogeny

Ancestral state estimations of the preferred substrate character for 19 nodes of the trapelioid phylogeny based on the method implemented in rayDISC in the R package corHMM imposing 12 different models. Please refer to the main text for details.

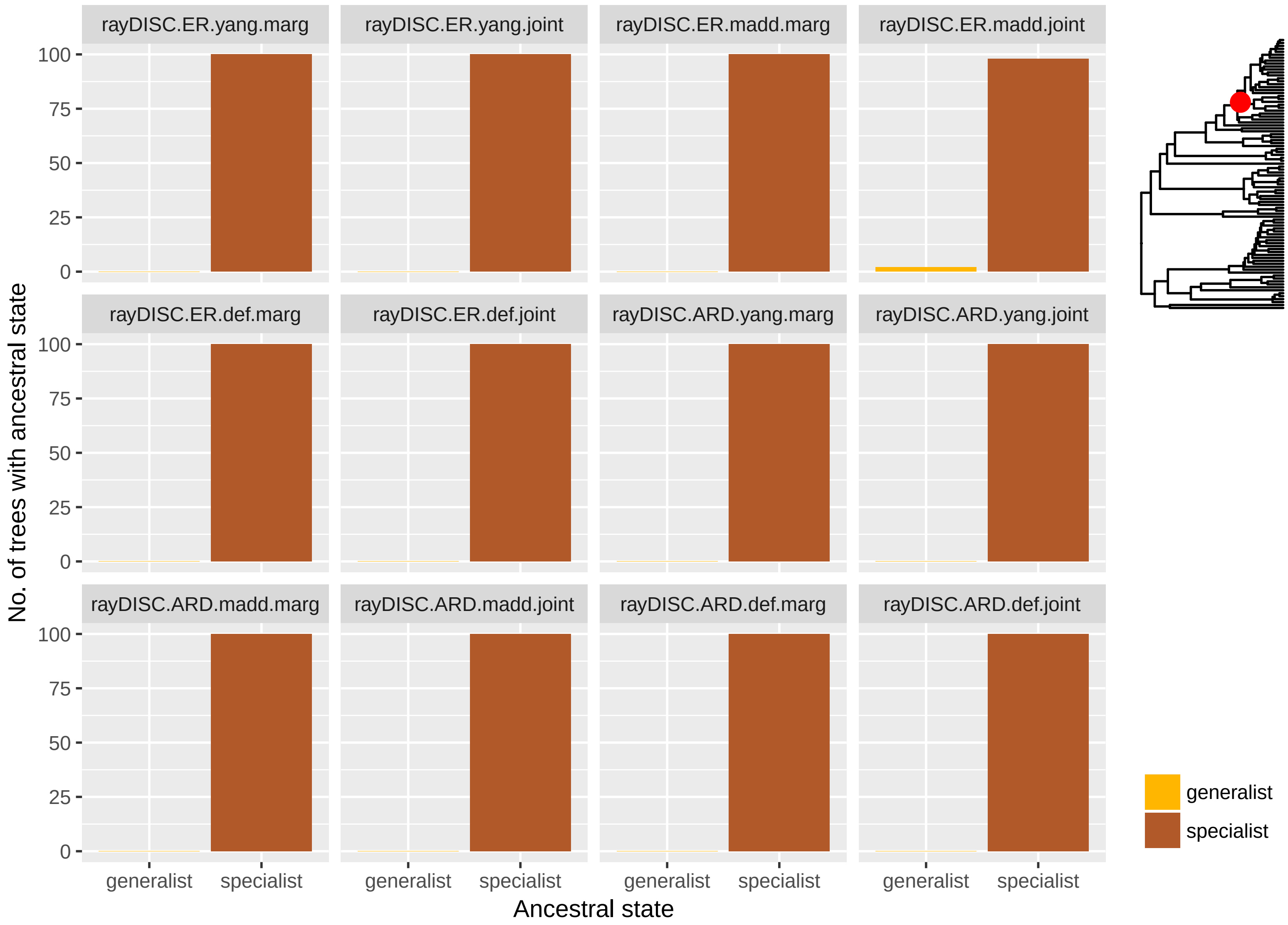

FigureS 80: Ancestral states for node 1

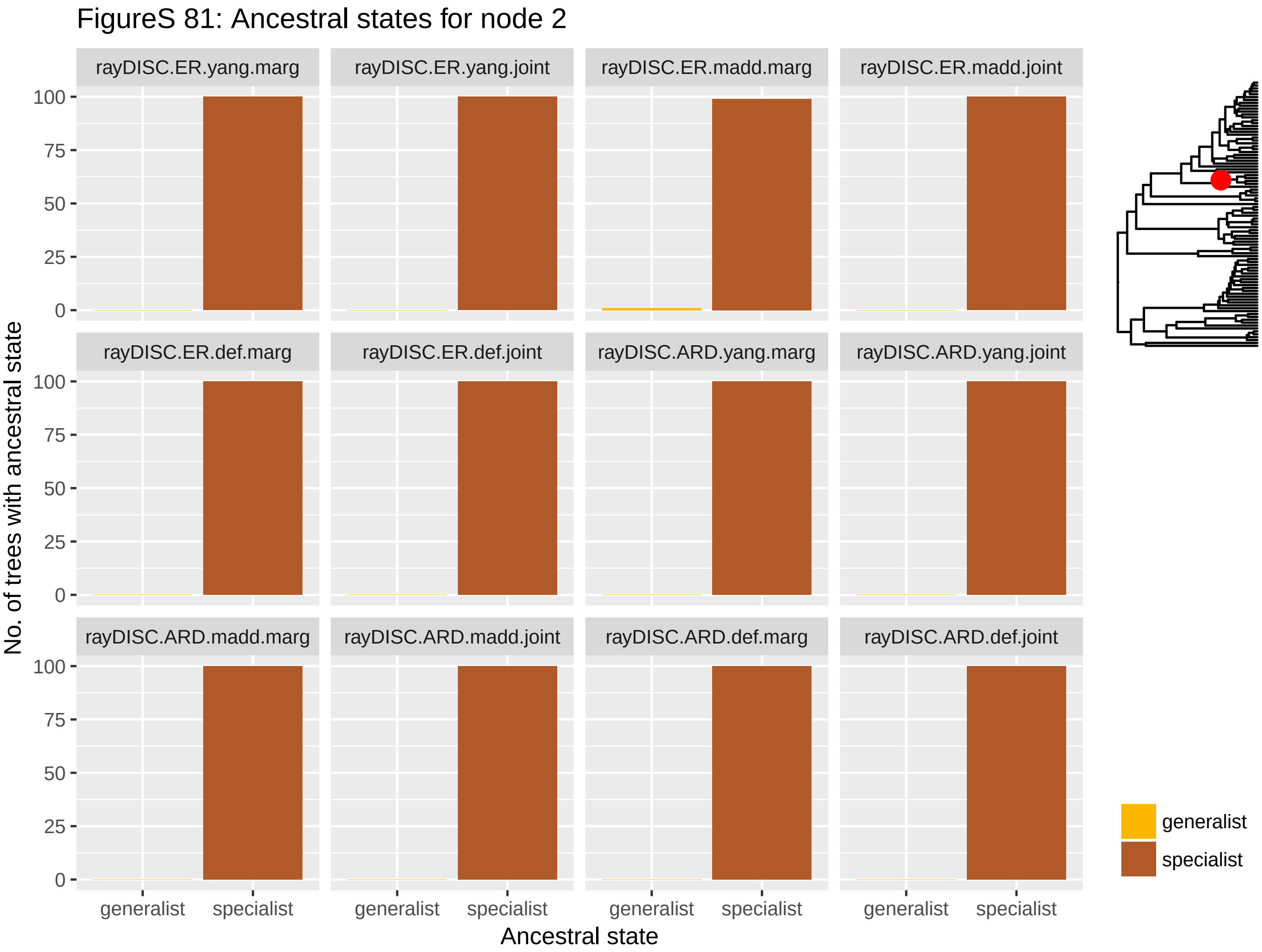

FigureS 81: Ancestral states for node 2
rayDISC.ER.yang.marg
rayDISC.ER.yang.joint
rayDISC.ER.madd.marg
rayDISC.ER.madd.joint
rayDISC.ER.def.marg
rayDISC.ER.def.joint
rayDISC.ARD.yang.marg
rayDISC.ARD.yang.joint
rayDISC.ARD.madd.marg
rayDISC.ARD.madd.joint
rayDISC.ARD.def.marg
rayDISC.ARD.def.joint
No. of trees with ancestral state
100
75
50
25
0
generalist
specialist
Ancestral state

FigureS 82: Ancestral states for node 3

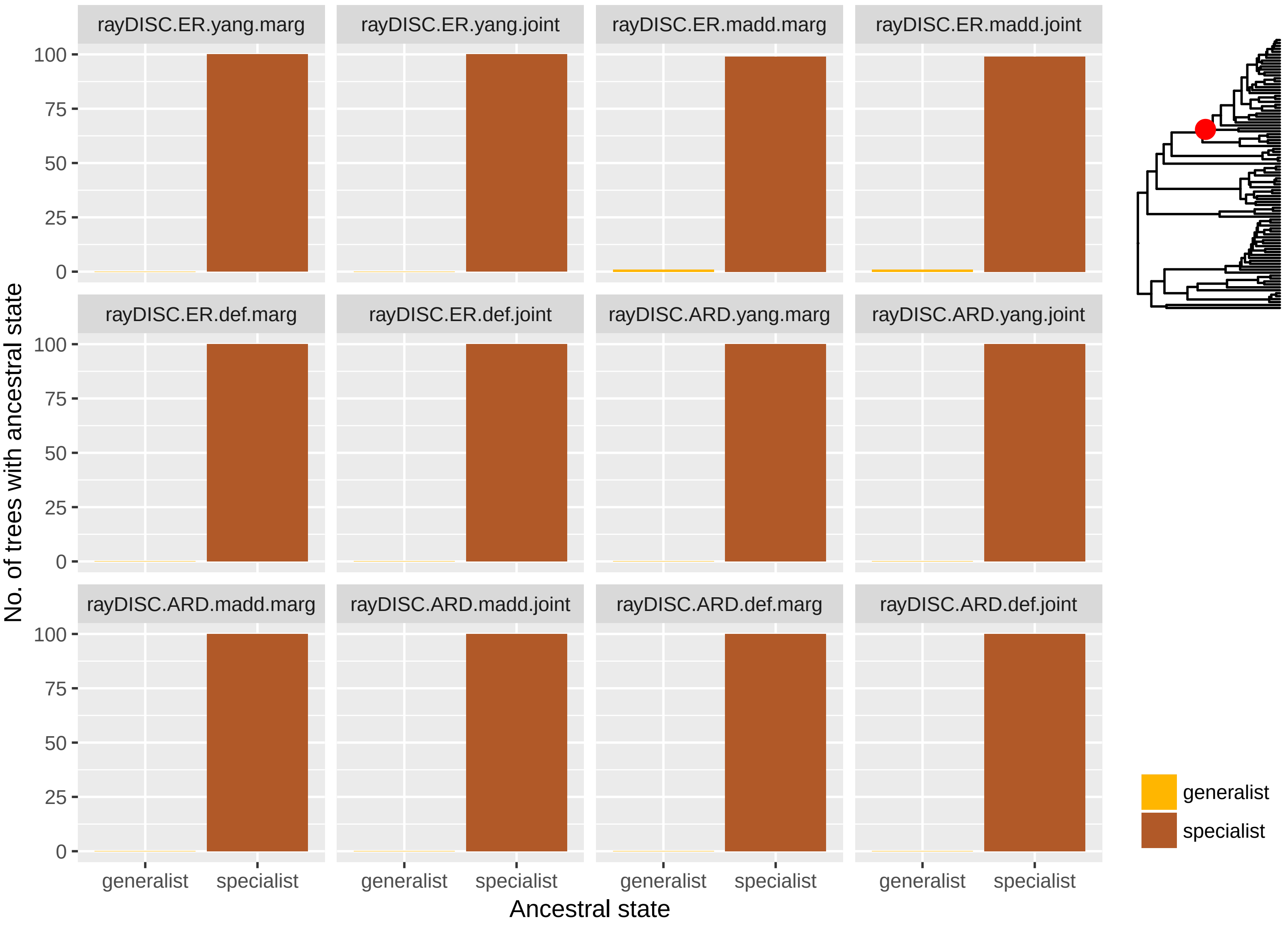

FigureS 83: Ancestral states for node 4

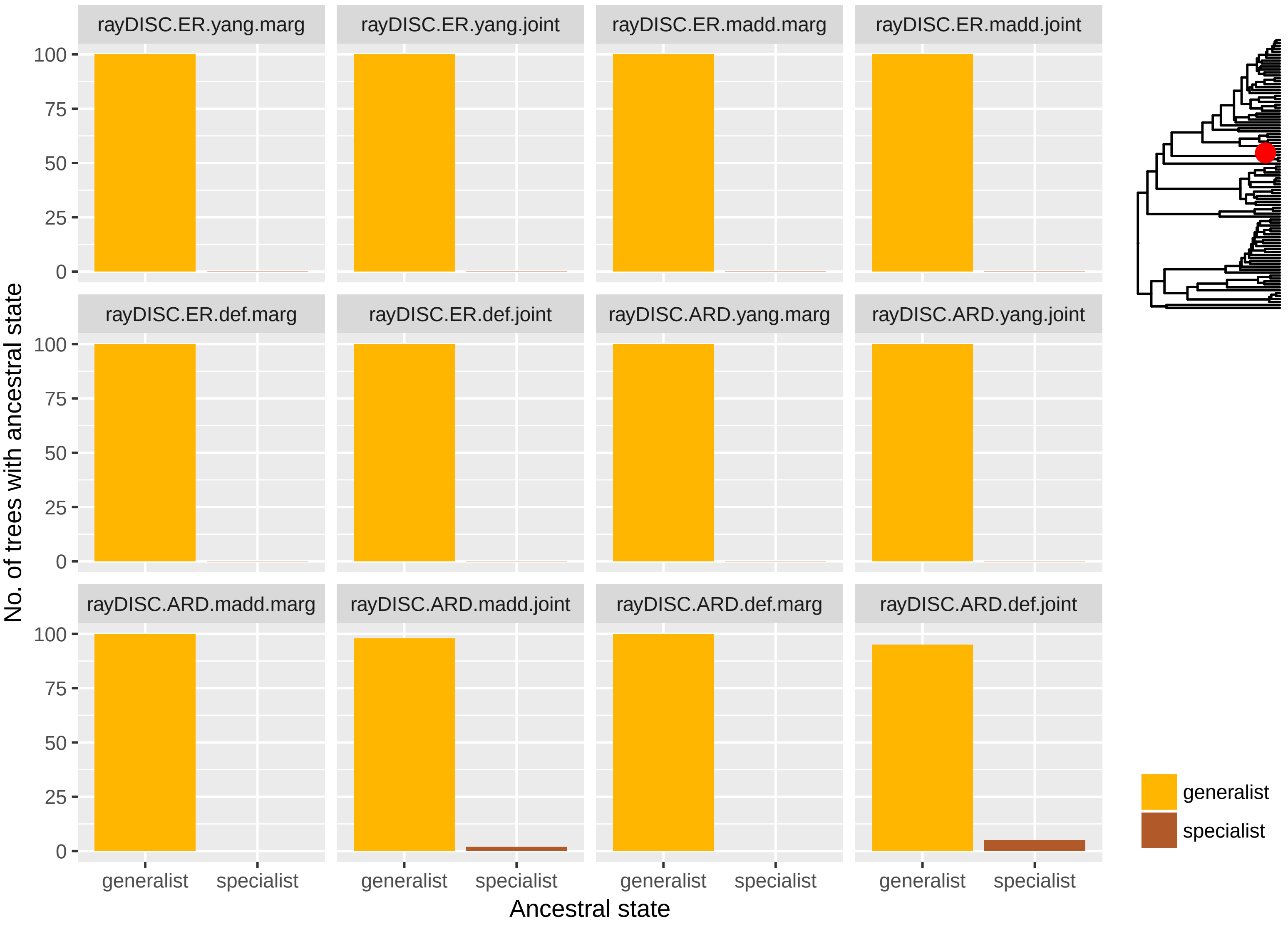

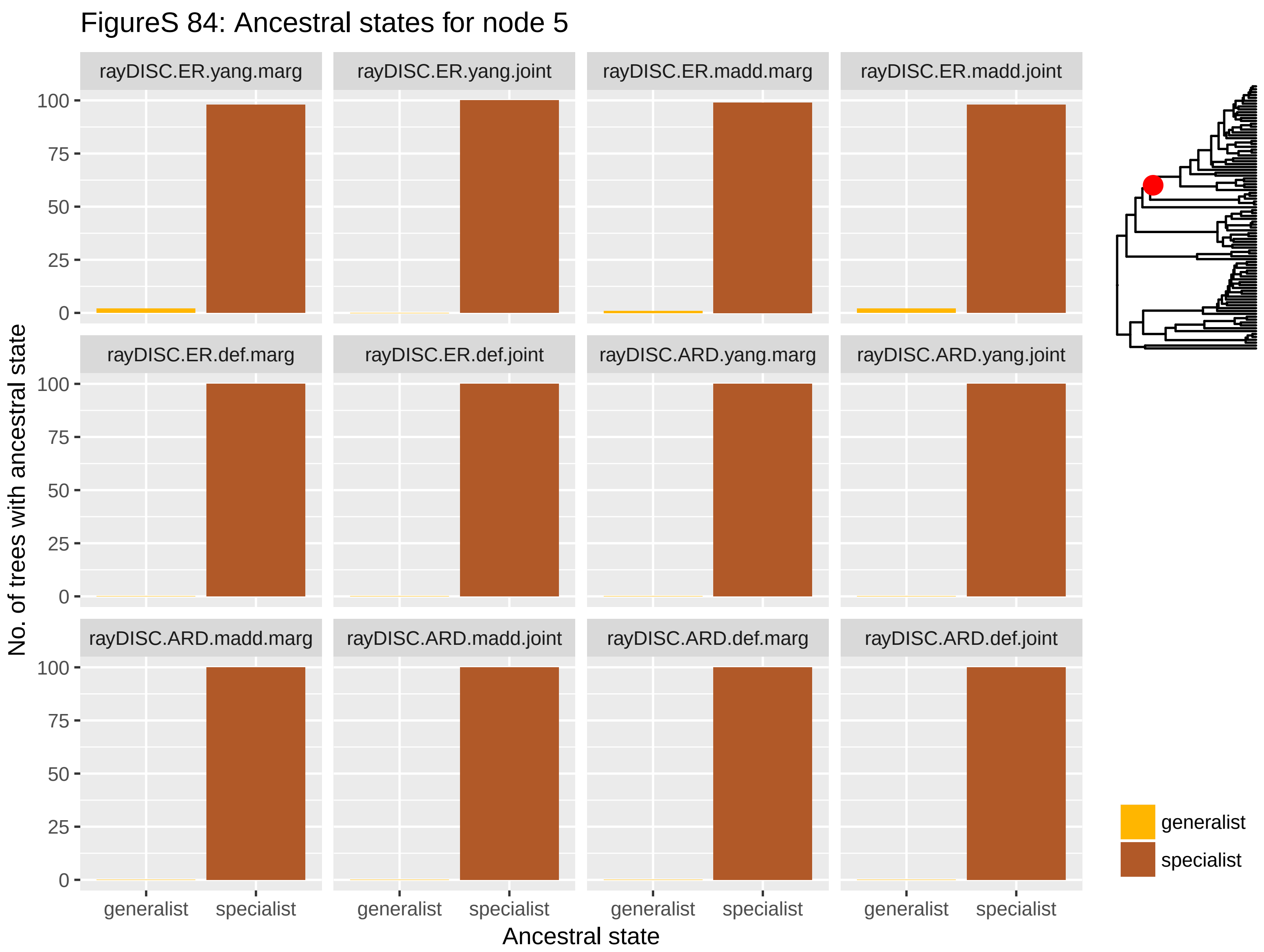

FigureS 84: Ancestral states for node 5
rayDISC.ER.yang.marg
rayDISC.ER.yang.joint
rayDISC.ER.madd.marg
rayDISC.ER.madd.joint
rayDISC.ER.def.marg
rayDISC.ER.def.joint
rayDISC.ARD.yang.marg
rayDISC.ARD.yang.joint
rayDISC.ARD.madd.marg
rayDISC.ARD.madd.joint
rayDISC.ARD.def.marg
rayDISC.ARD.def.joint
No. of trees with ancestral state
0
25
50
75
100
generalist
specialist
Ancestral state
generalist
specialist

FigureS 85: Ancestral states for node 6

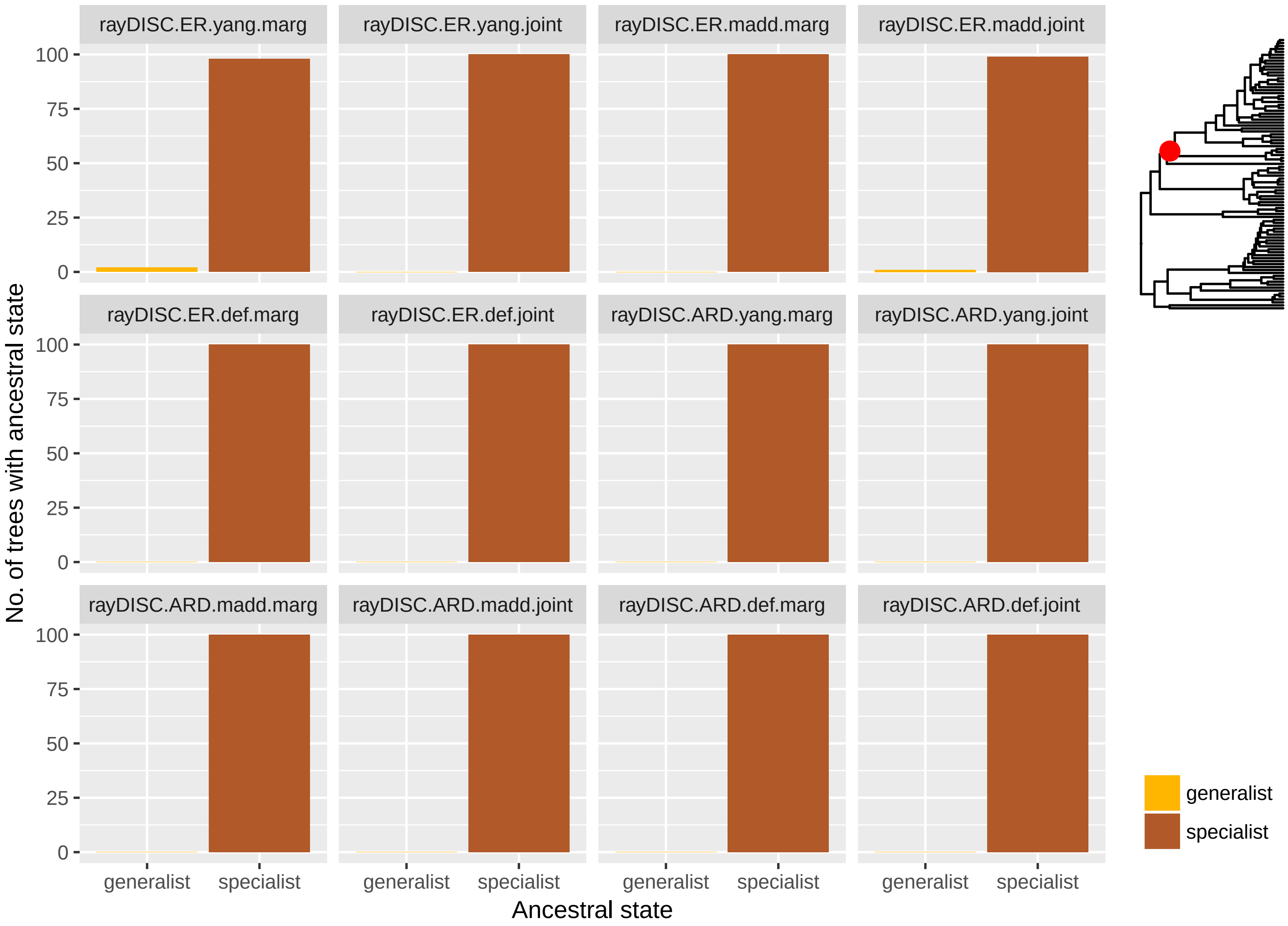

FigureS 86: Ancestral states for node 7

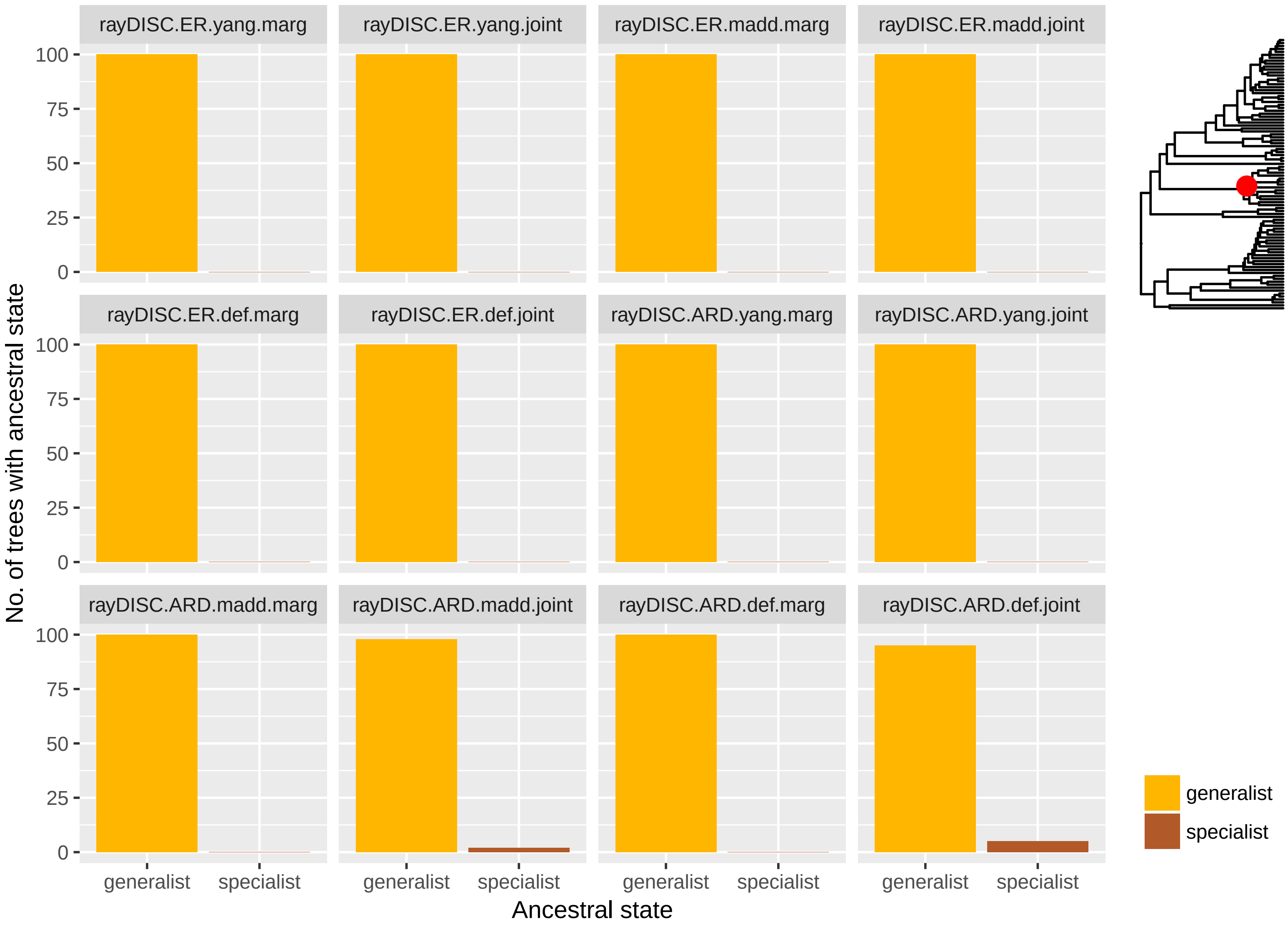

FigureS 87: Ancestral states for node 8

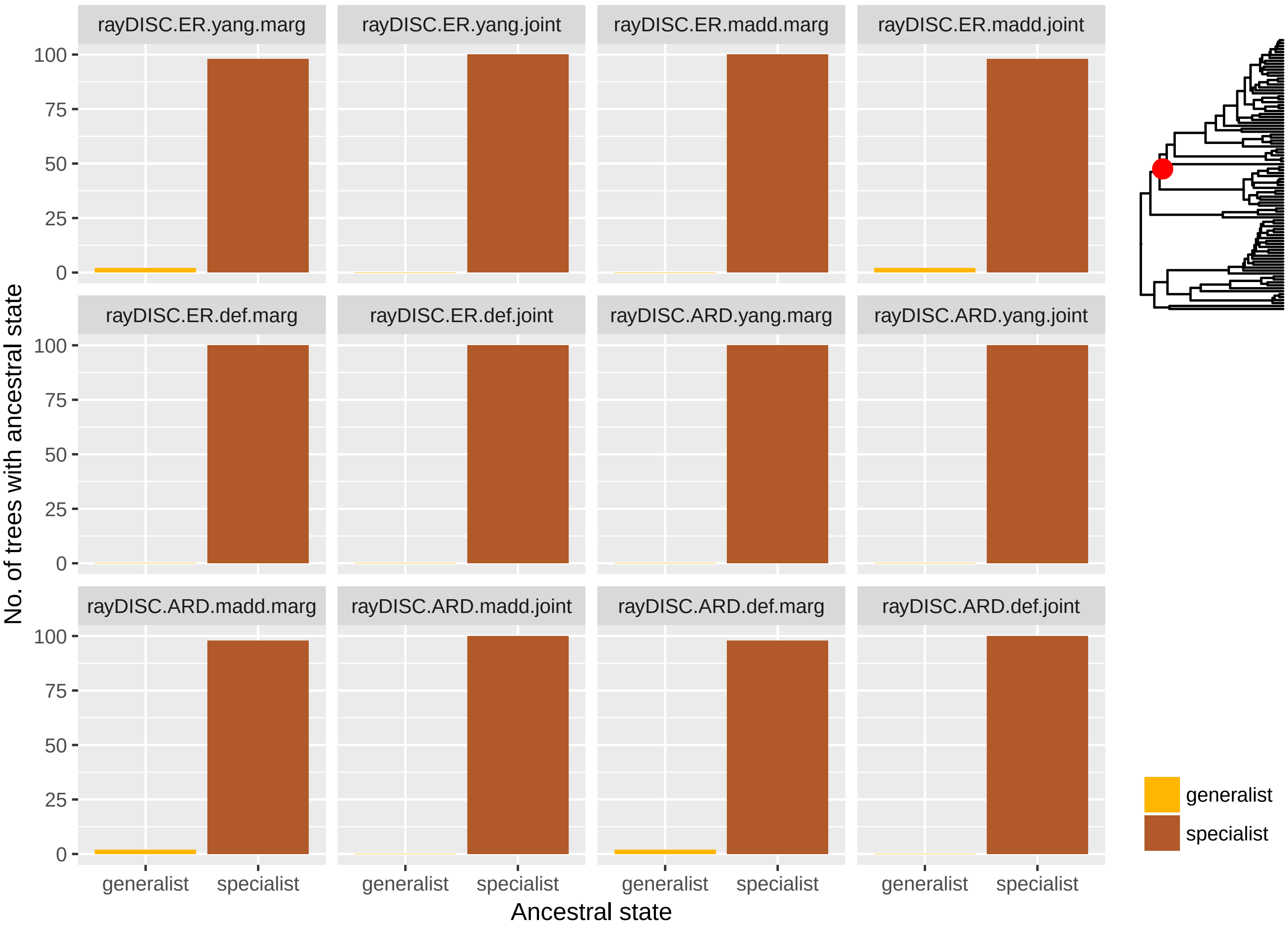

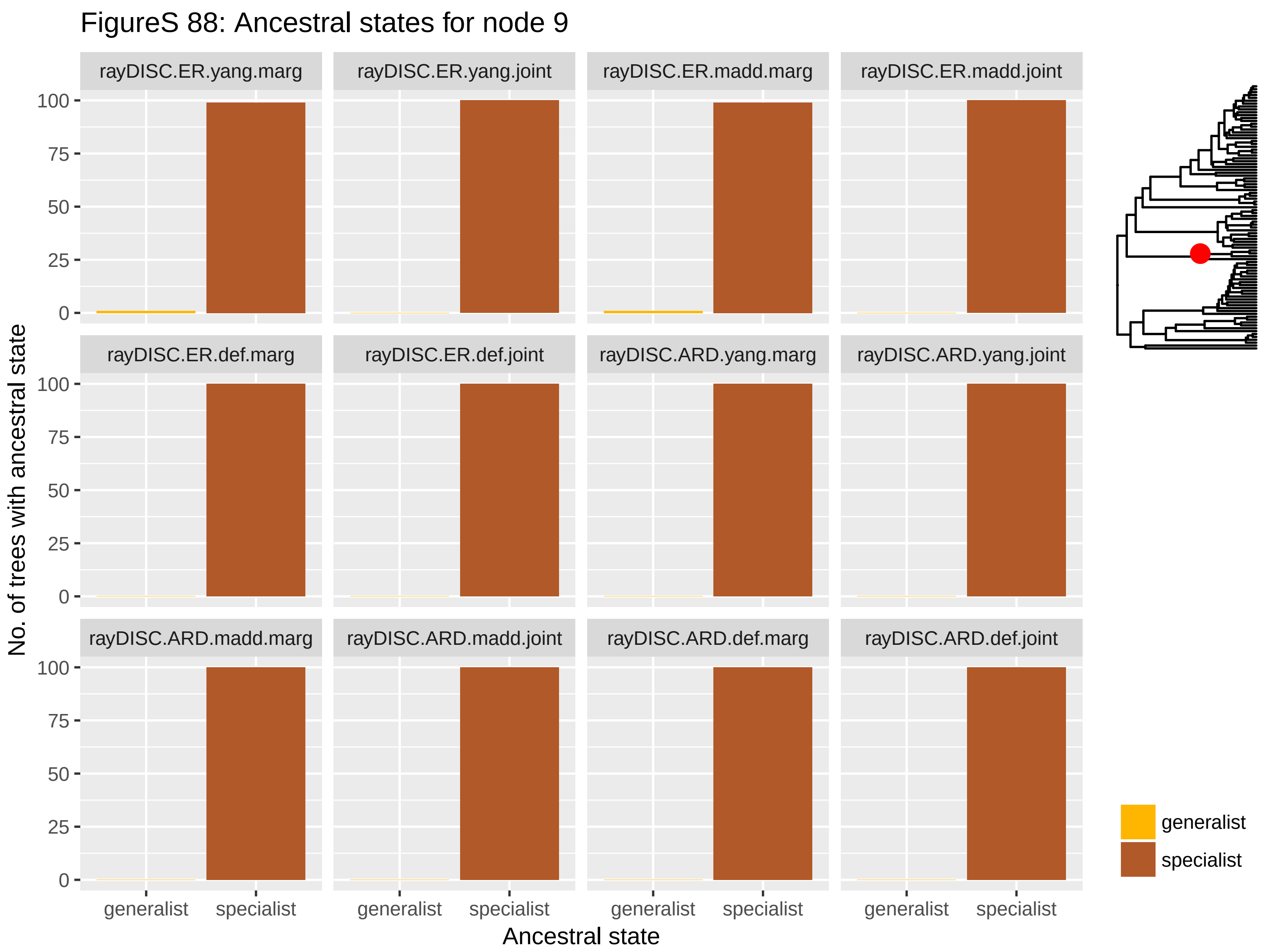
FigureS 88: Ancestral states for node 9
rayDISC.ER.yang.marg
rayDISC.ER.yang.joint
rayDISC.ER.madd.marg
rayDISC.ER.madd.joint
rayDISC.ER.def.marg
rayDISC.ER.def.joint
rayDISC.ARD.yang.marg
rayDISC.ARD.yang.joint
rayDISC.ARD.madd.marg
rayDISC.ARD.madd.joint
rayDISC.ARD.def.marg
rayDISC.ARD.def.joint
No. of trees with ancestral state
0
25
50
75
100
generalist
specialist
Ancestral state
generalist
specialist

FigureS 89: Ancestral states for node 10

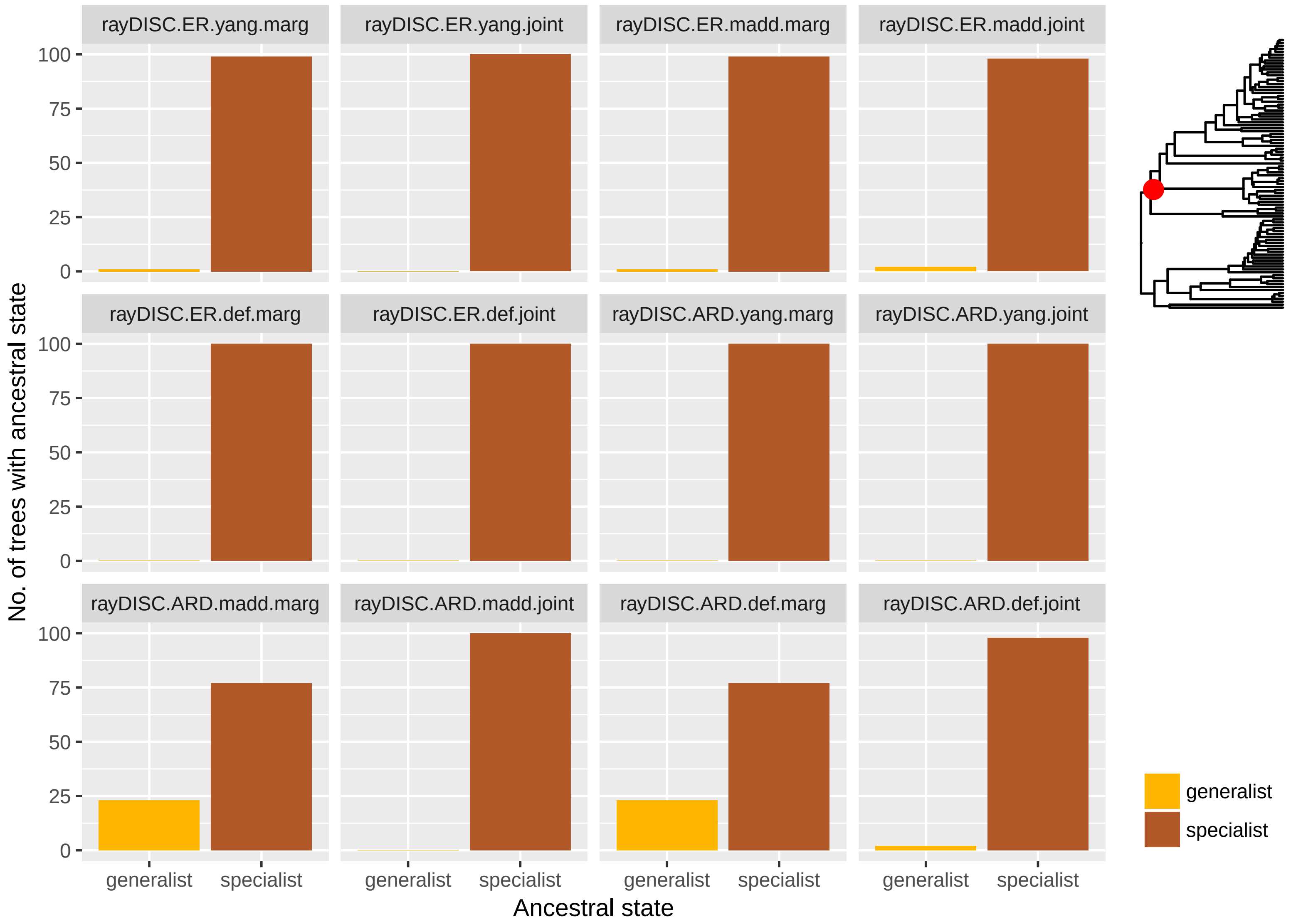

FigureS 90: Ancestral states for node 11

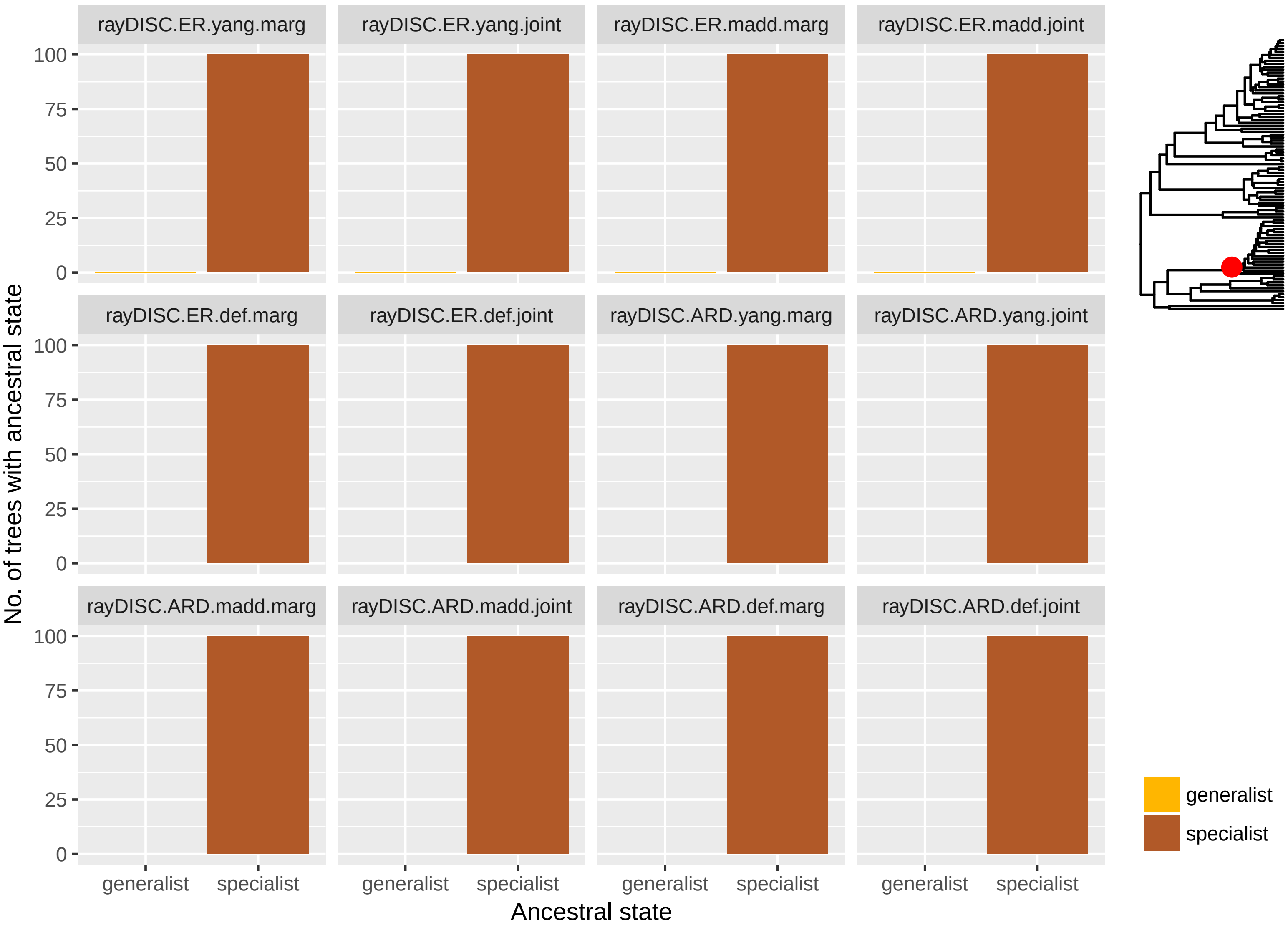

FigureS 91: Ancestral states for node 12

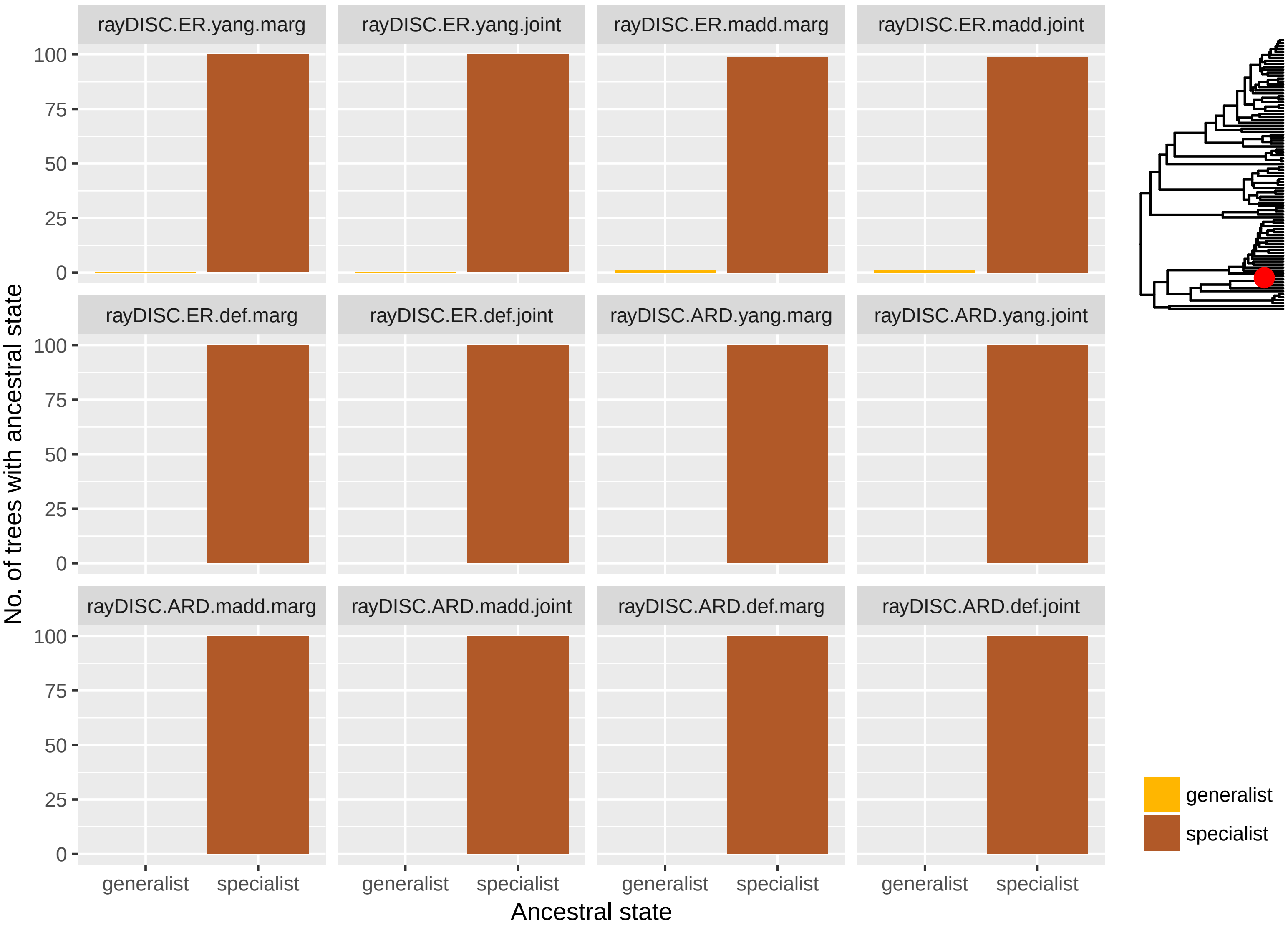

FigureS 92: Ancestral states for node 13

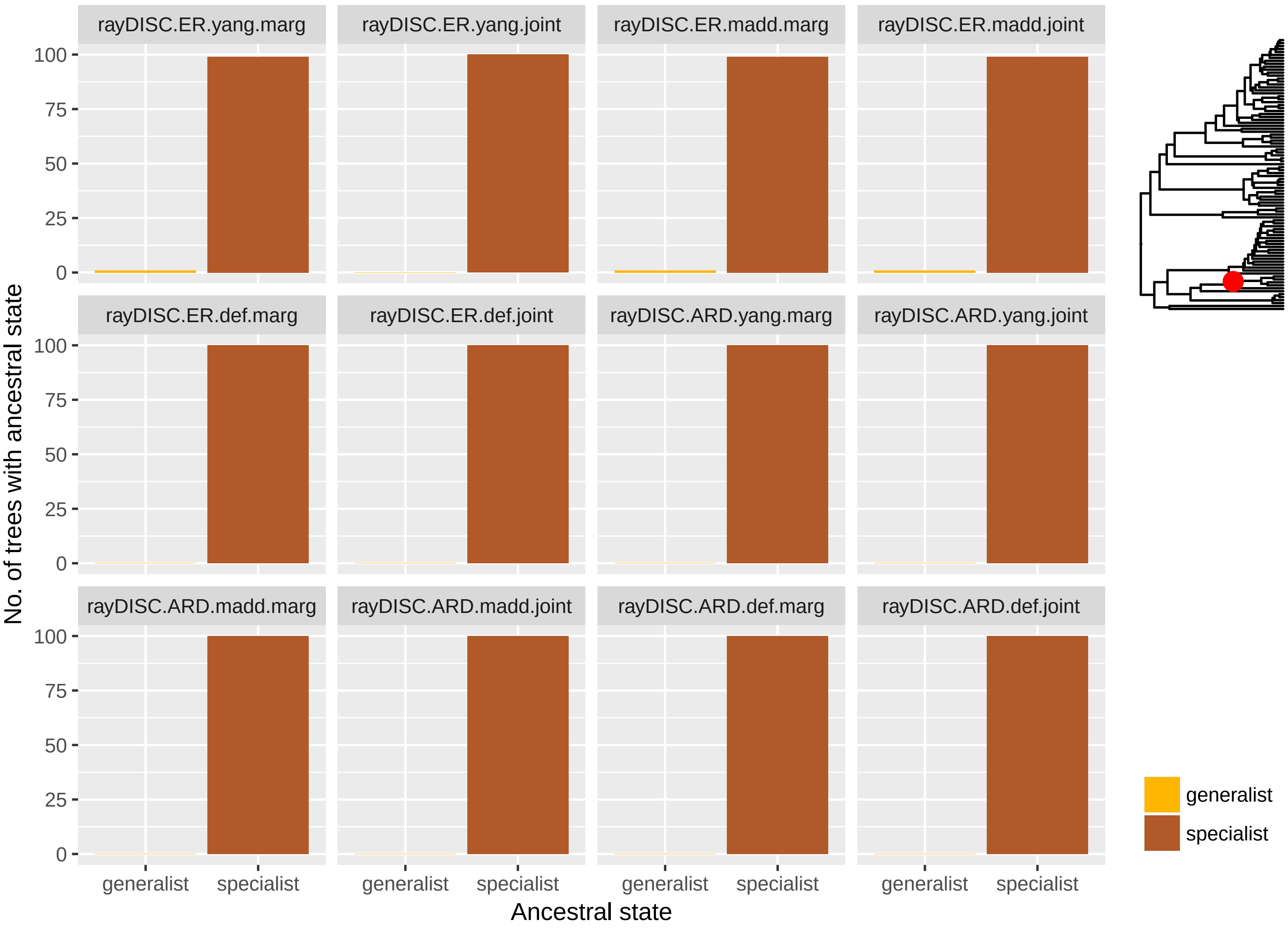

FigureS 93: Ancestral states for node 14

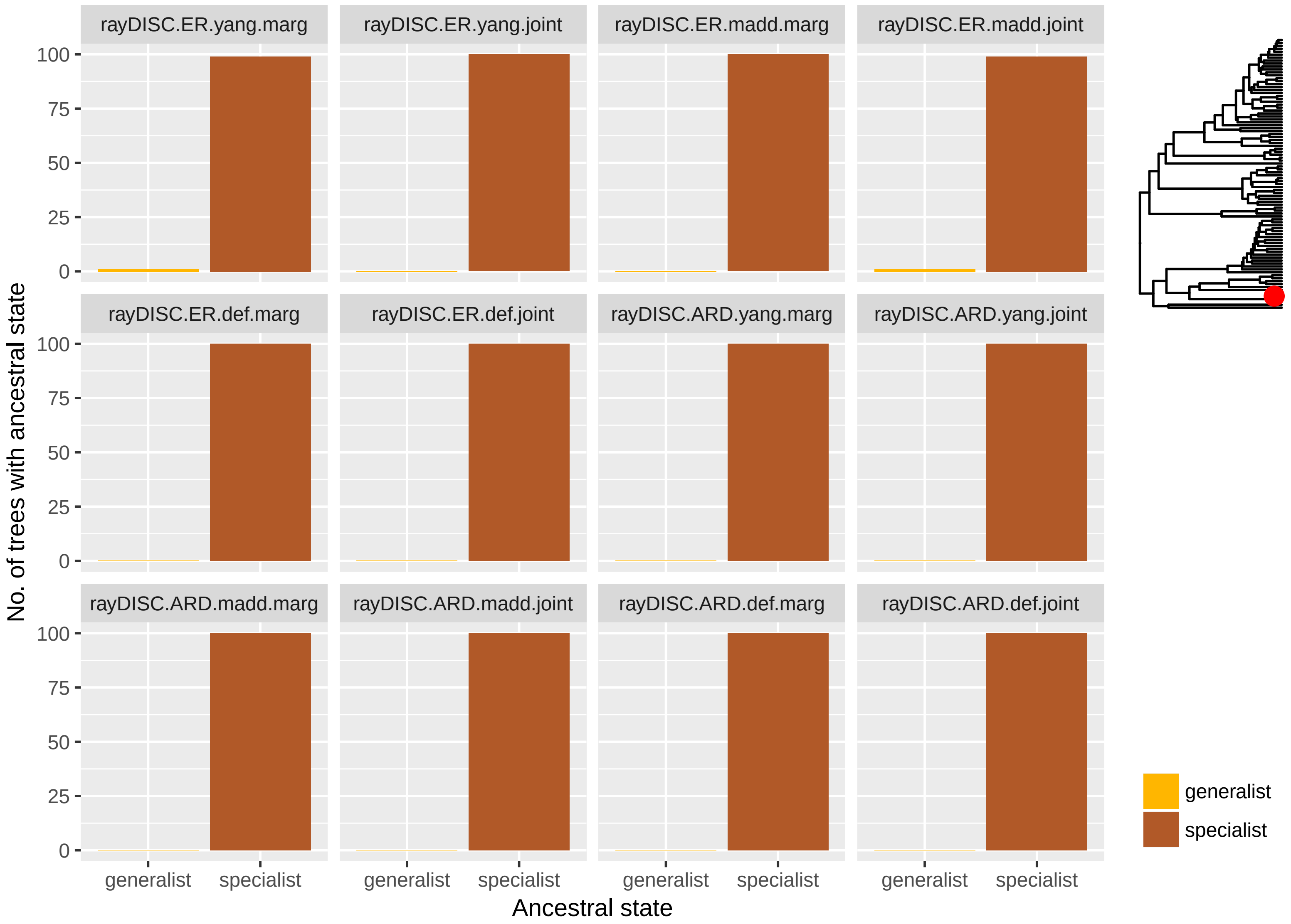

FigureS 94: Ancestral states for node 15

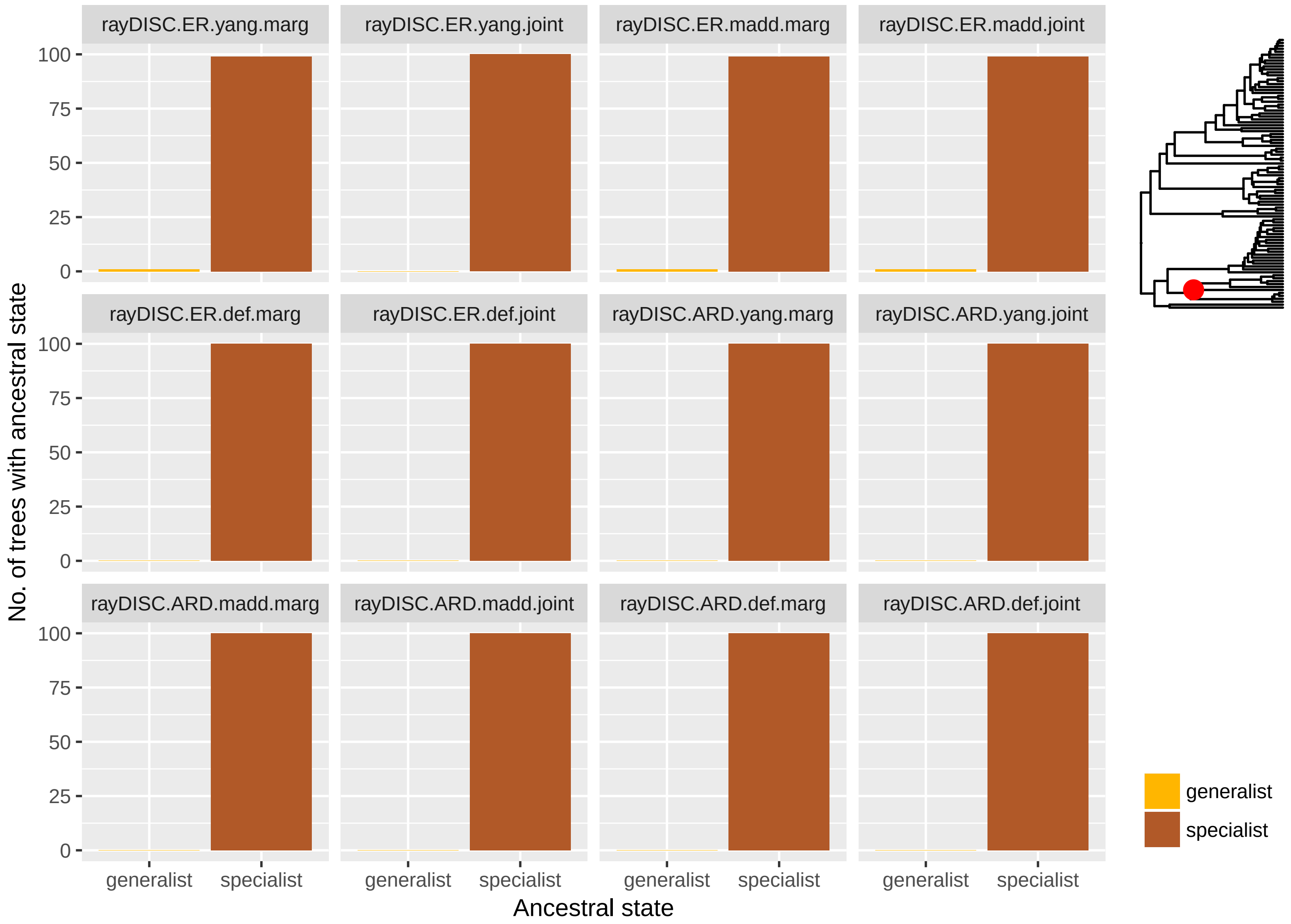

FigureS 95: Ancestral states for node 16

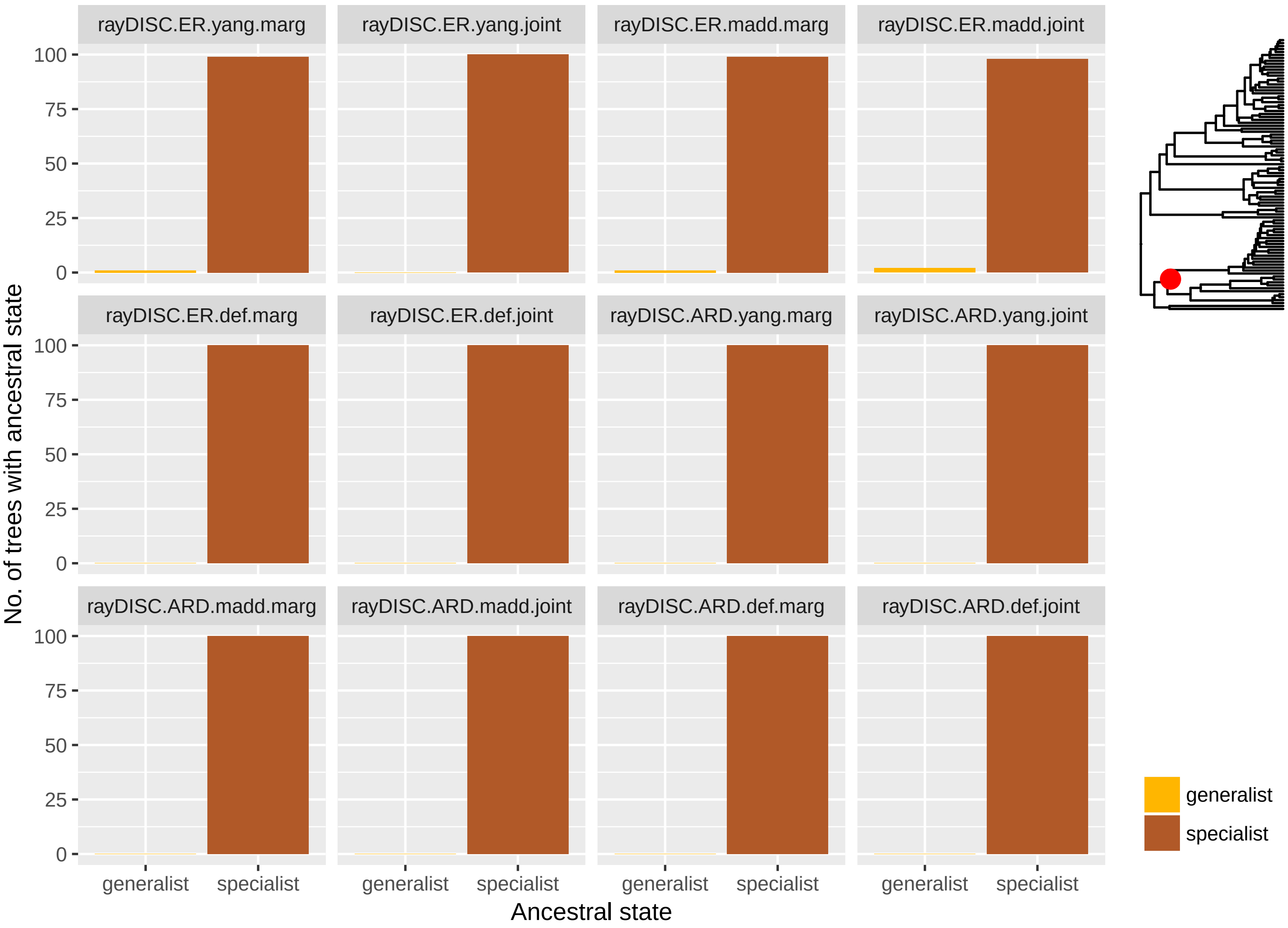

FigureS 96: Ancestral states for node 17

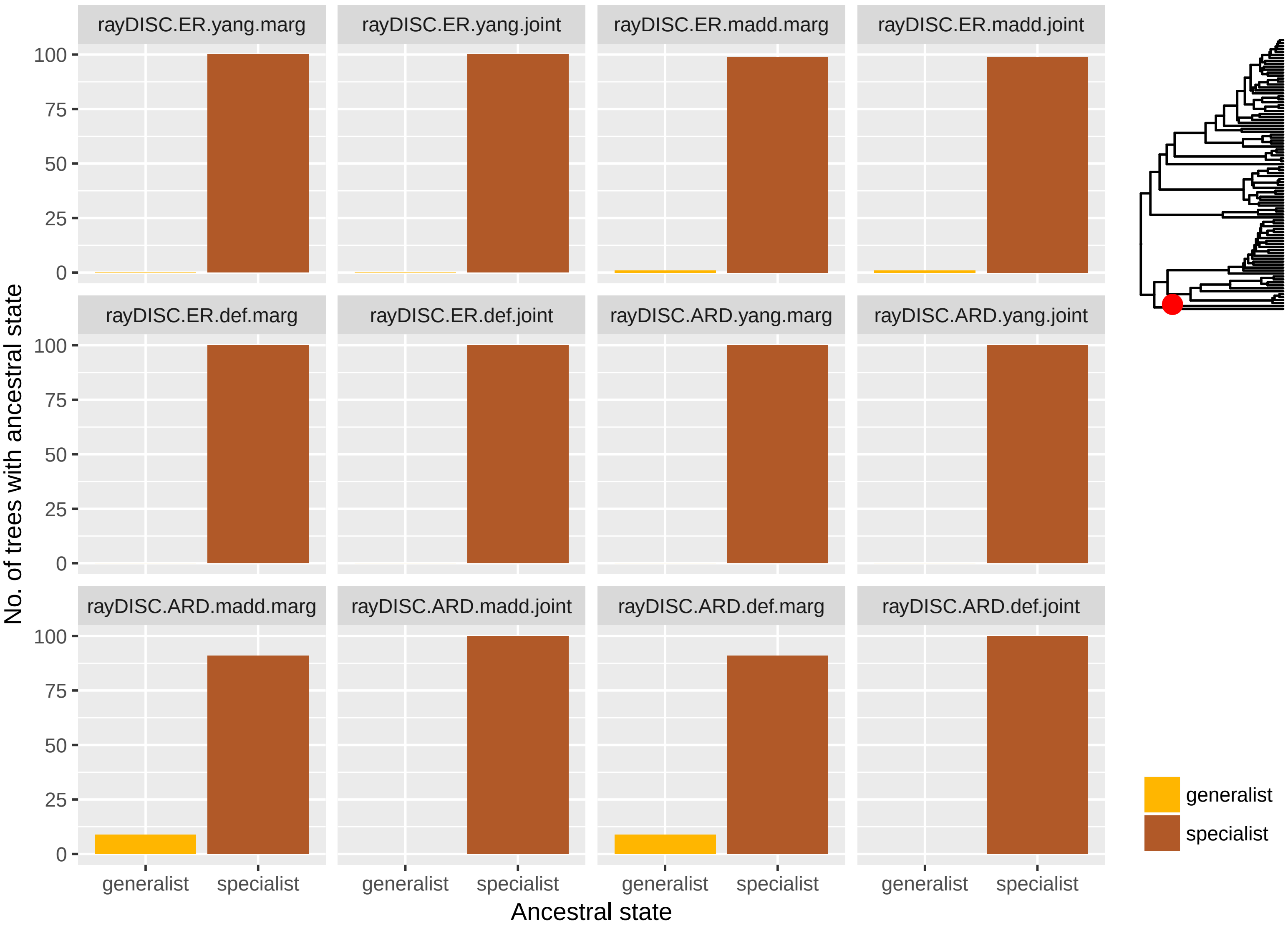

FigureS 97: Ancestral states for node 18

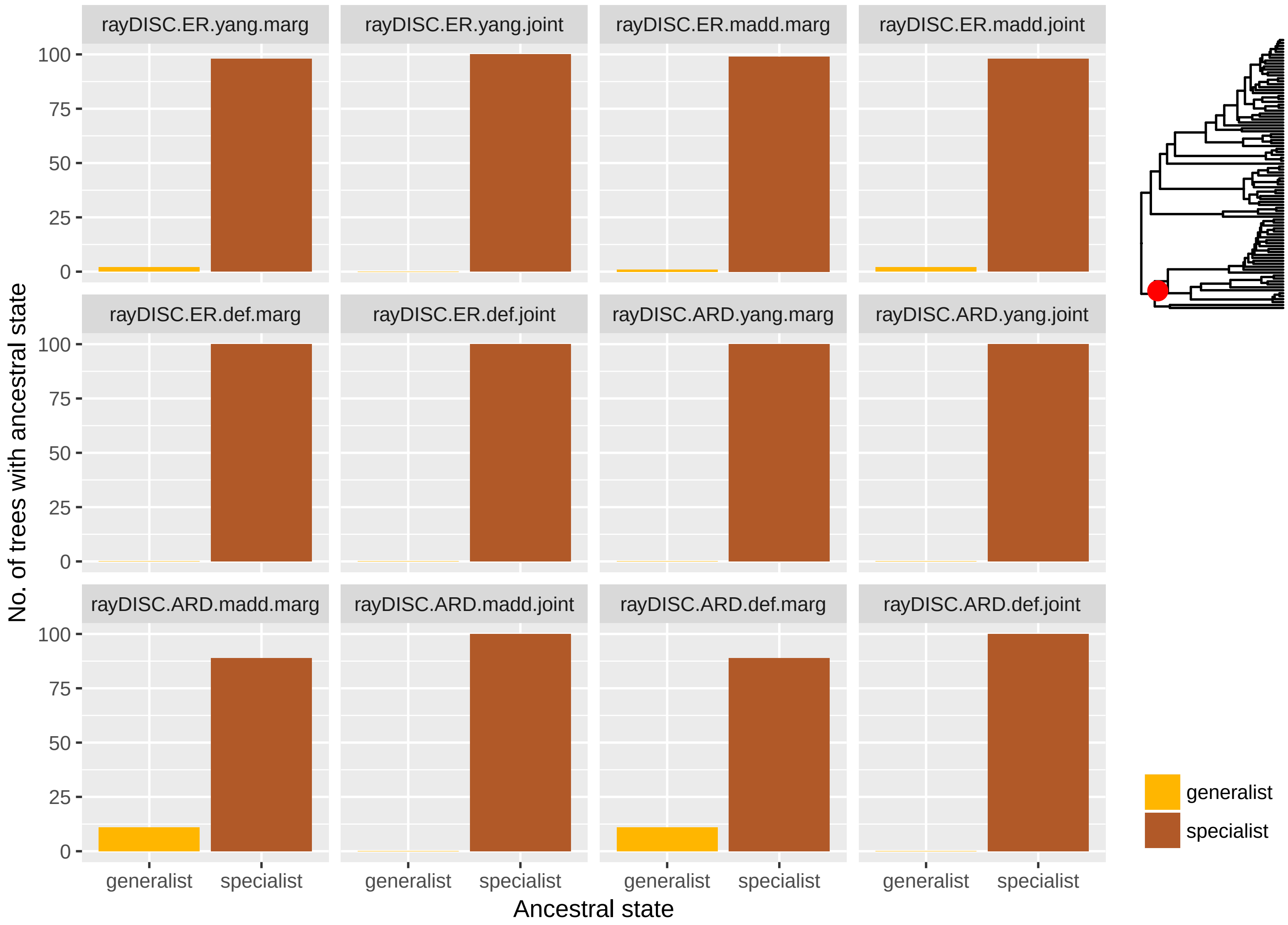

FigureS 98: Ancestral states for node 19

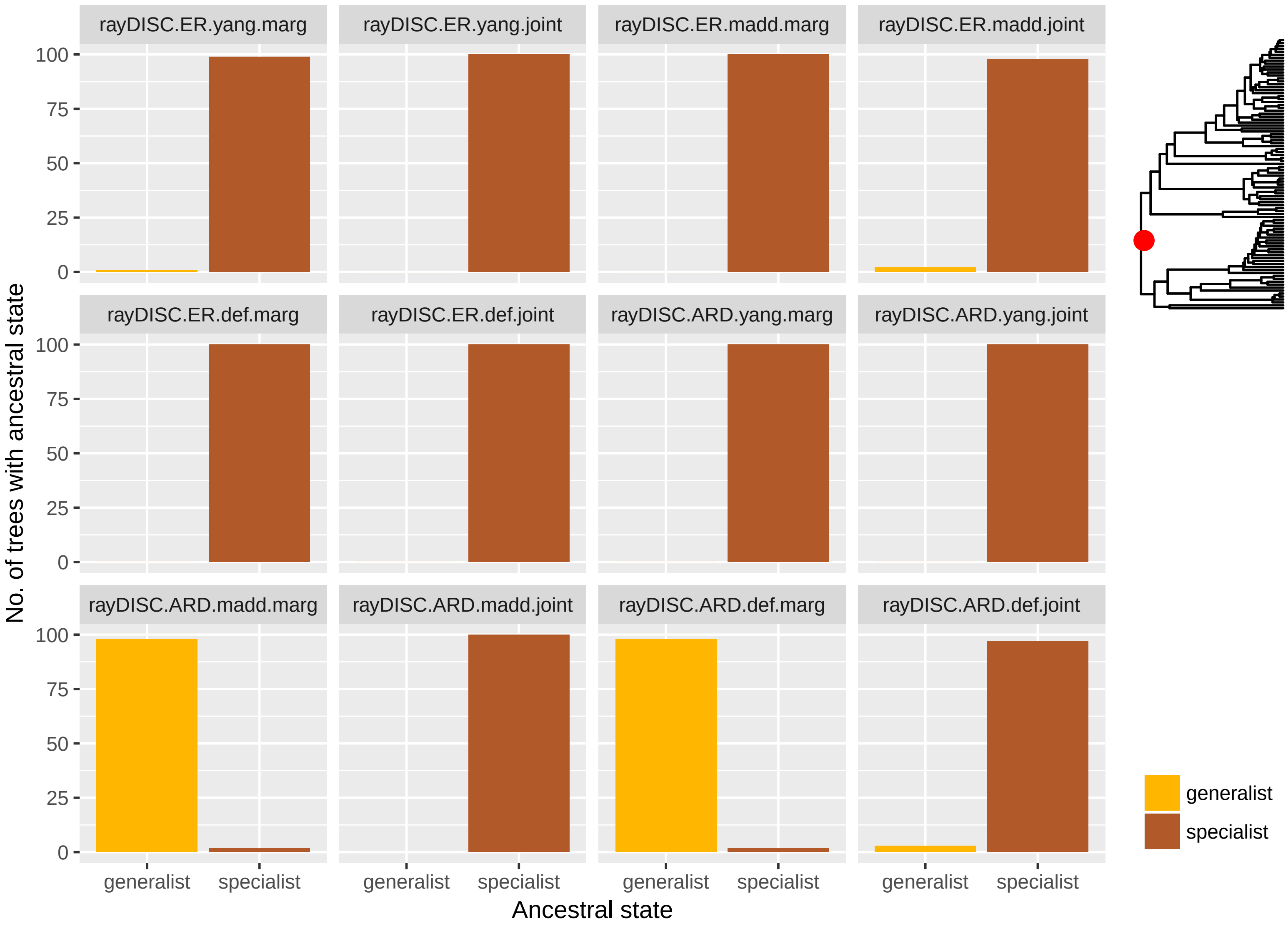

Figure S99-S117 - Results of ancestral state estimation of ecological strategy character with make-simmap for 19 nodes of the trapelioid phylogeny

Ancestral state estimations of the preferred substrate character for 19 nodes of the trapelioid phylogeny based on stochastic character mapping using the R package phytools imposing 4 different models. Please refer to the main text for details.

FigureS 99: Ancestral states for node 1

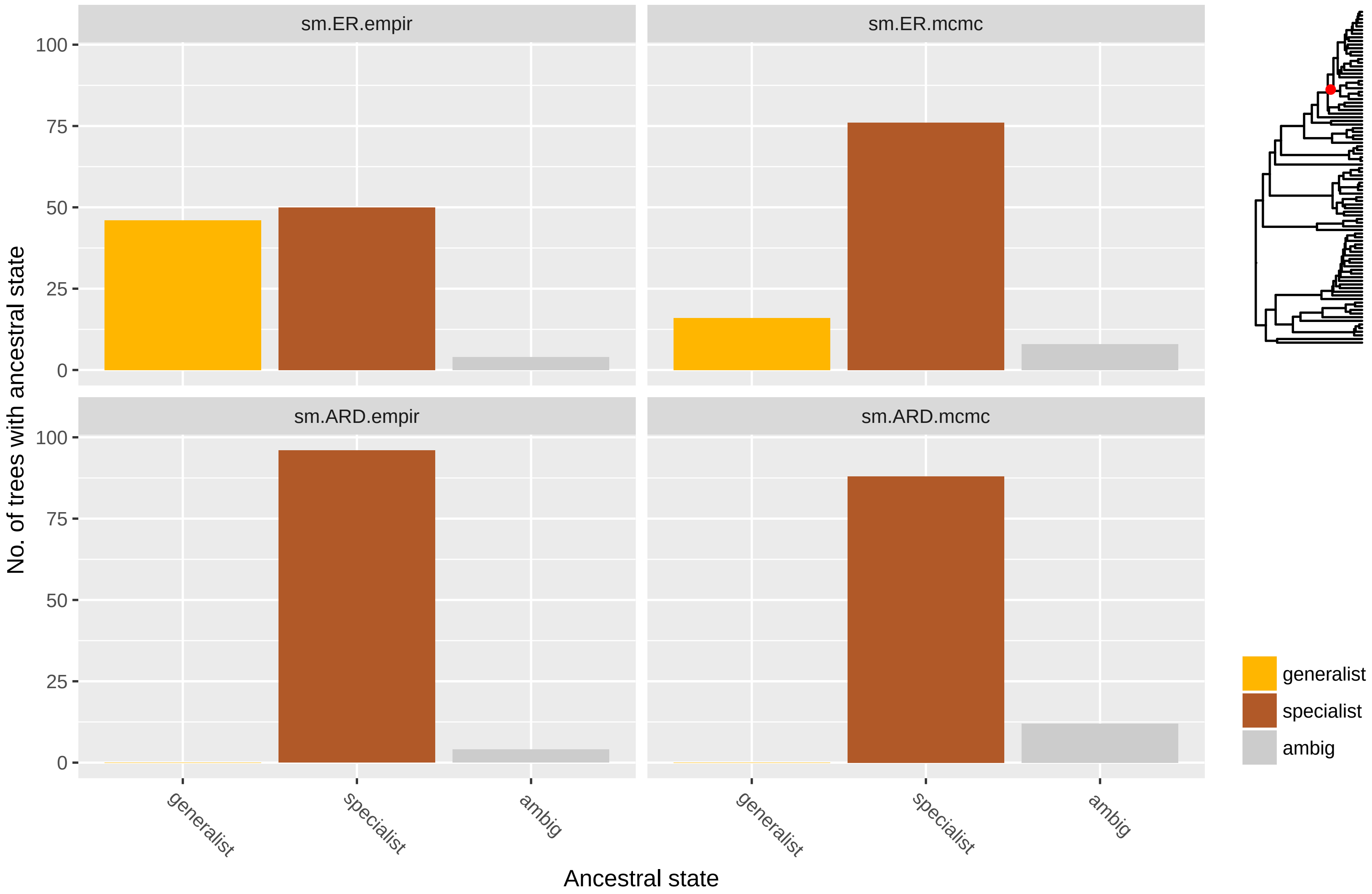

FigureS 100: Ancestral states for node 2

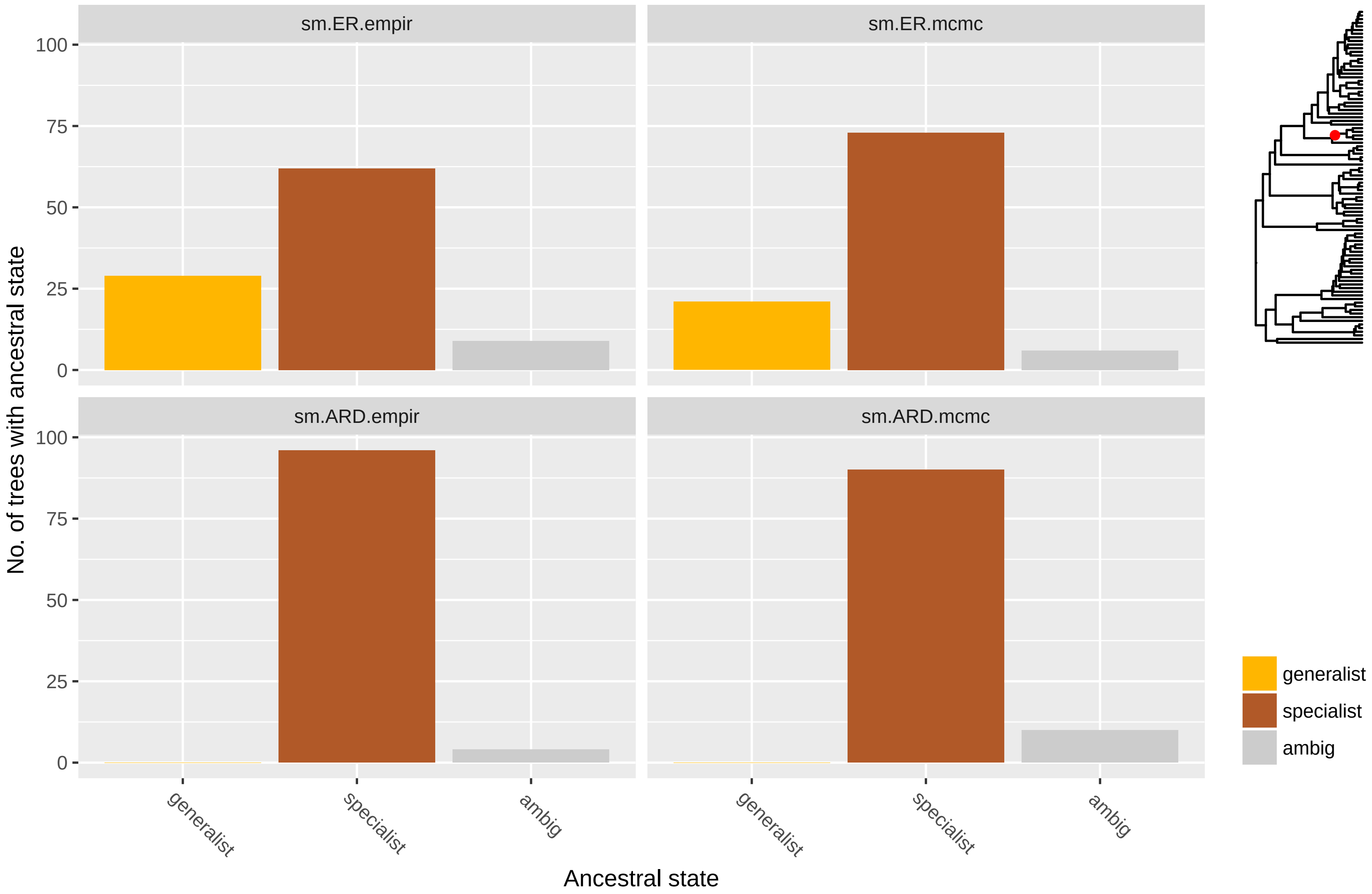

FigureS 101: Ancestral states for node 3

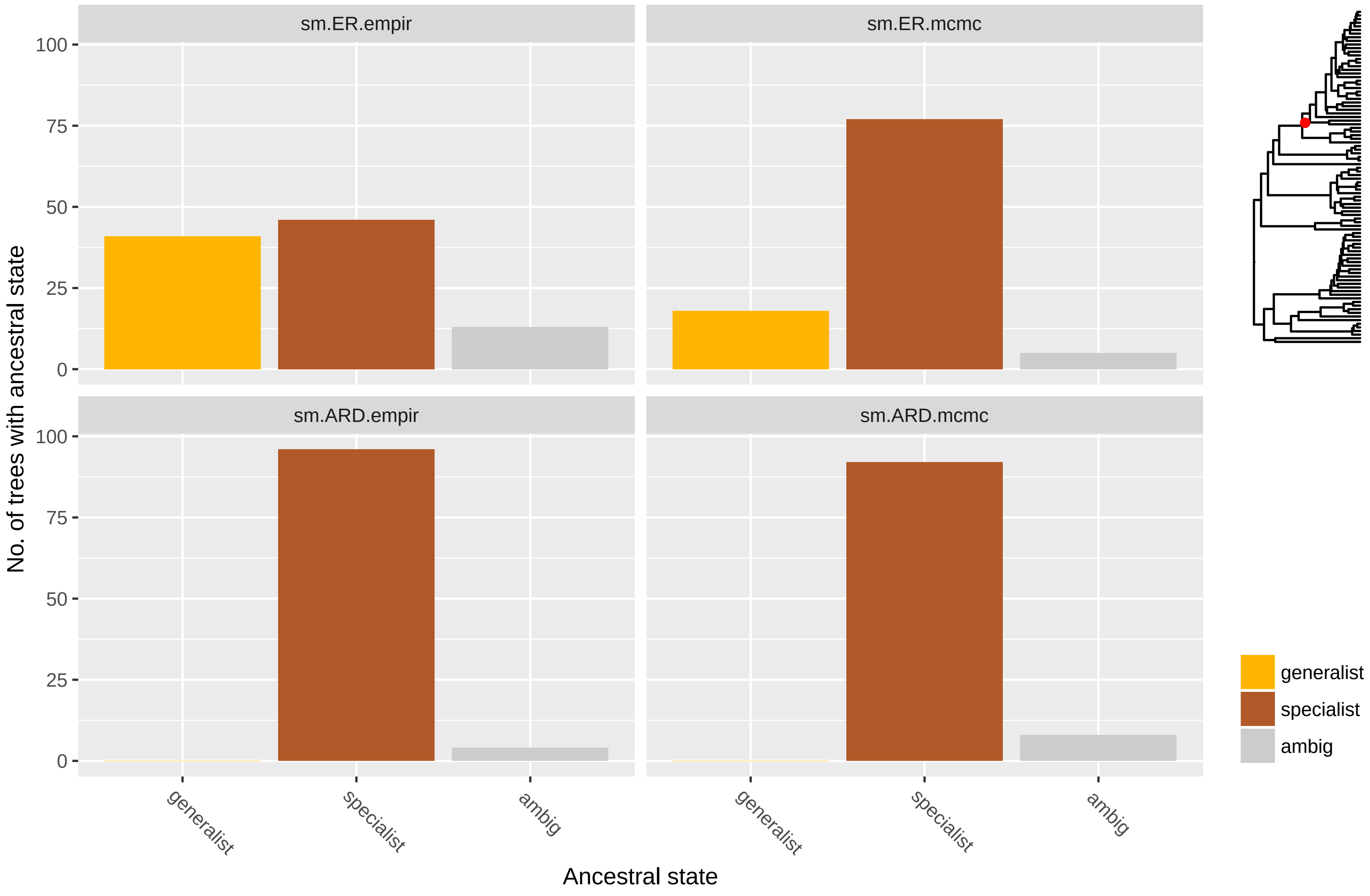

FigureS 102: Ancestral states for node 4

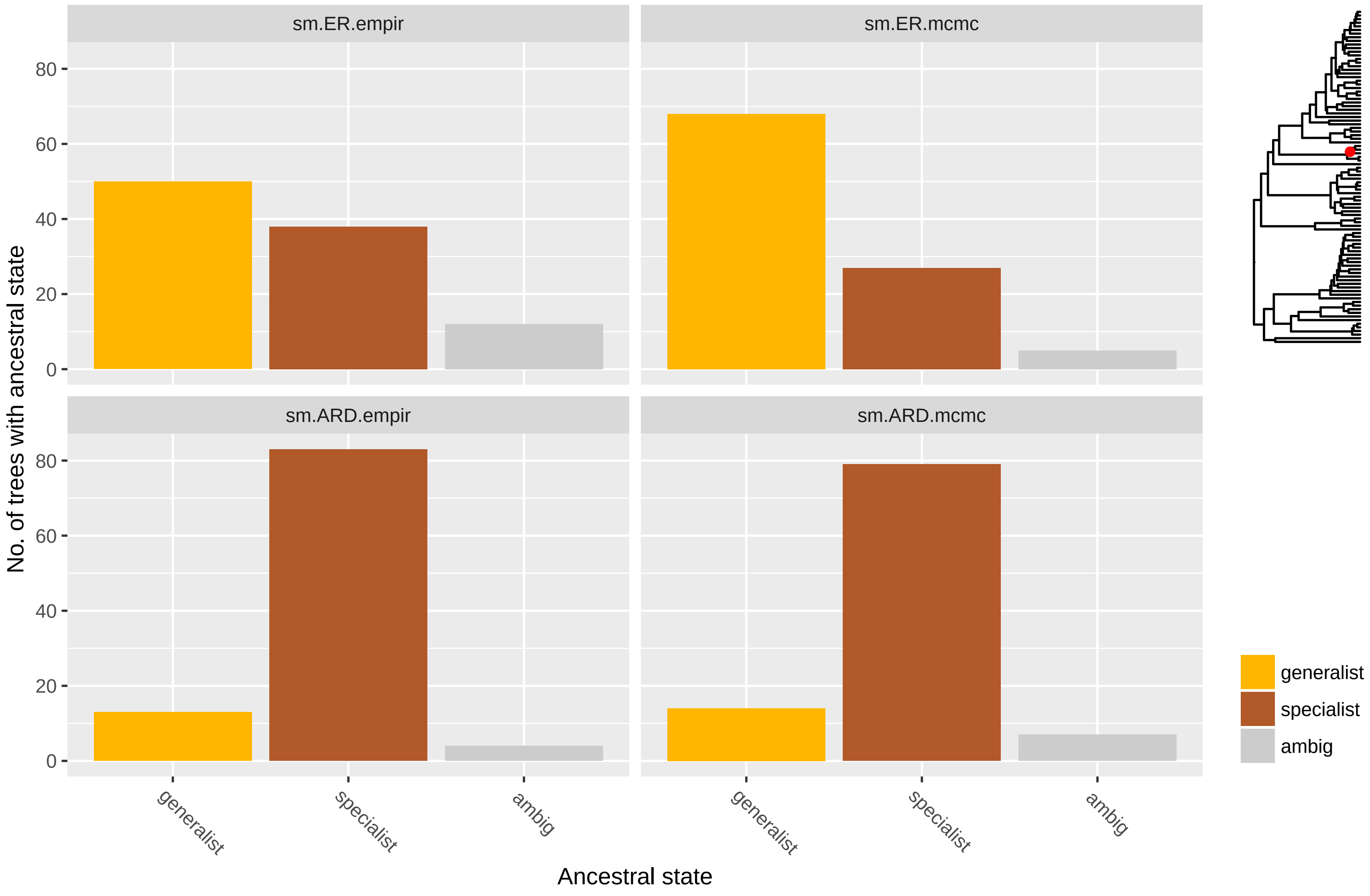

FigureS 103: Ancestral states for node 5

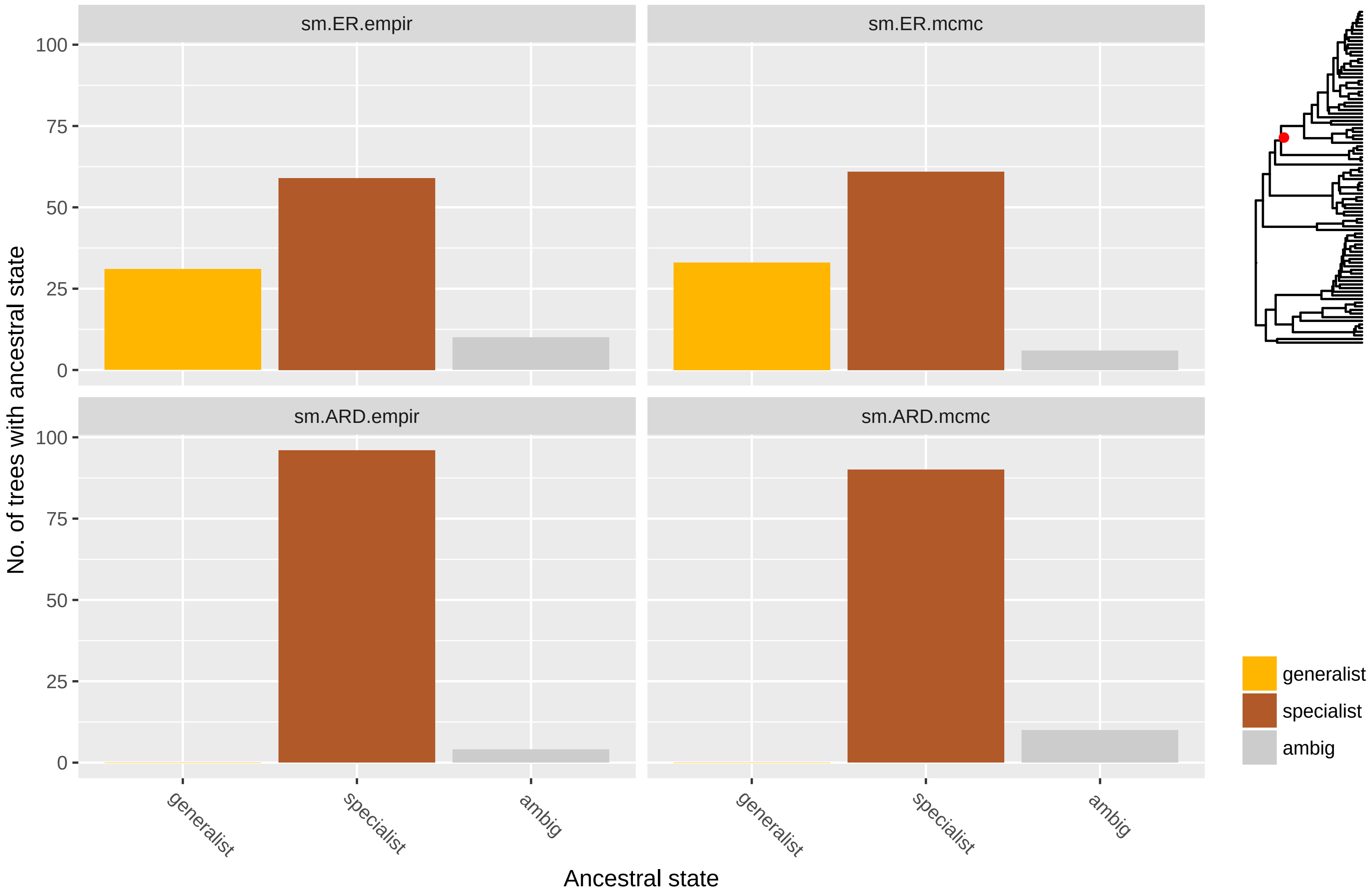

FigureS 104: Ancestral states for node 6

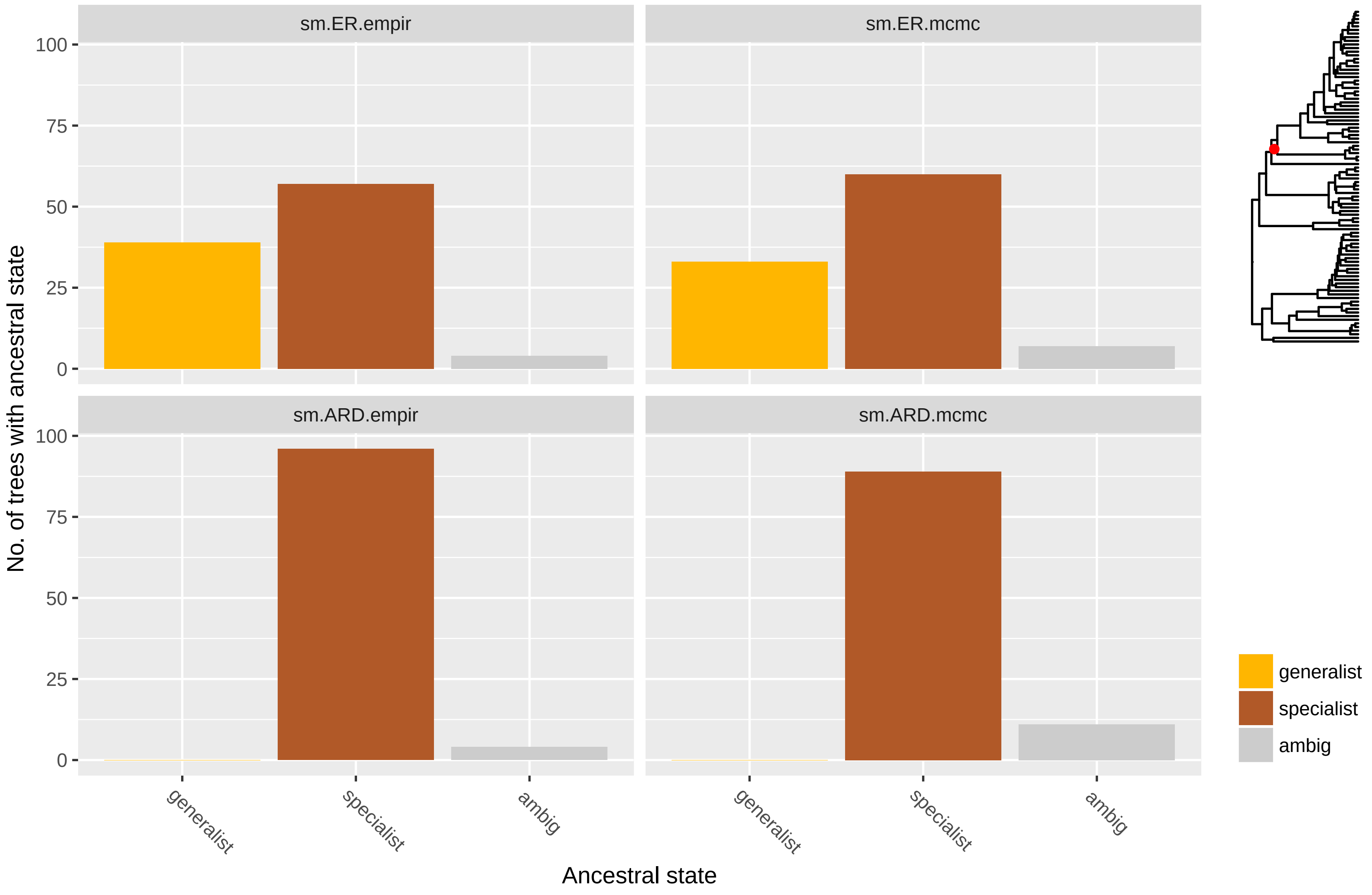

FigureS 105: Ancestral states for node 7

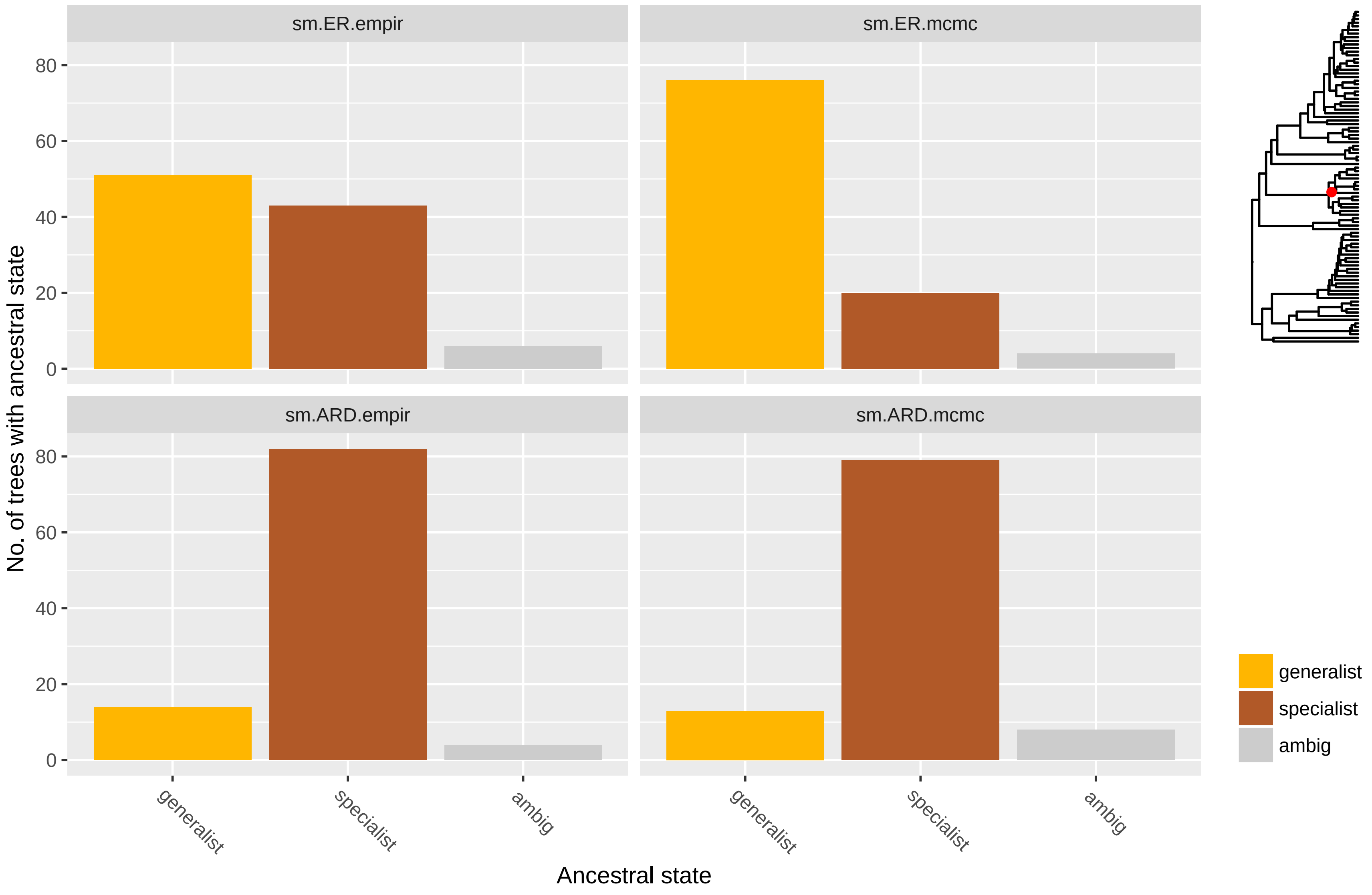

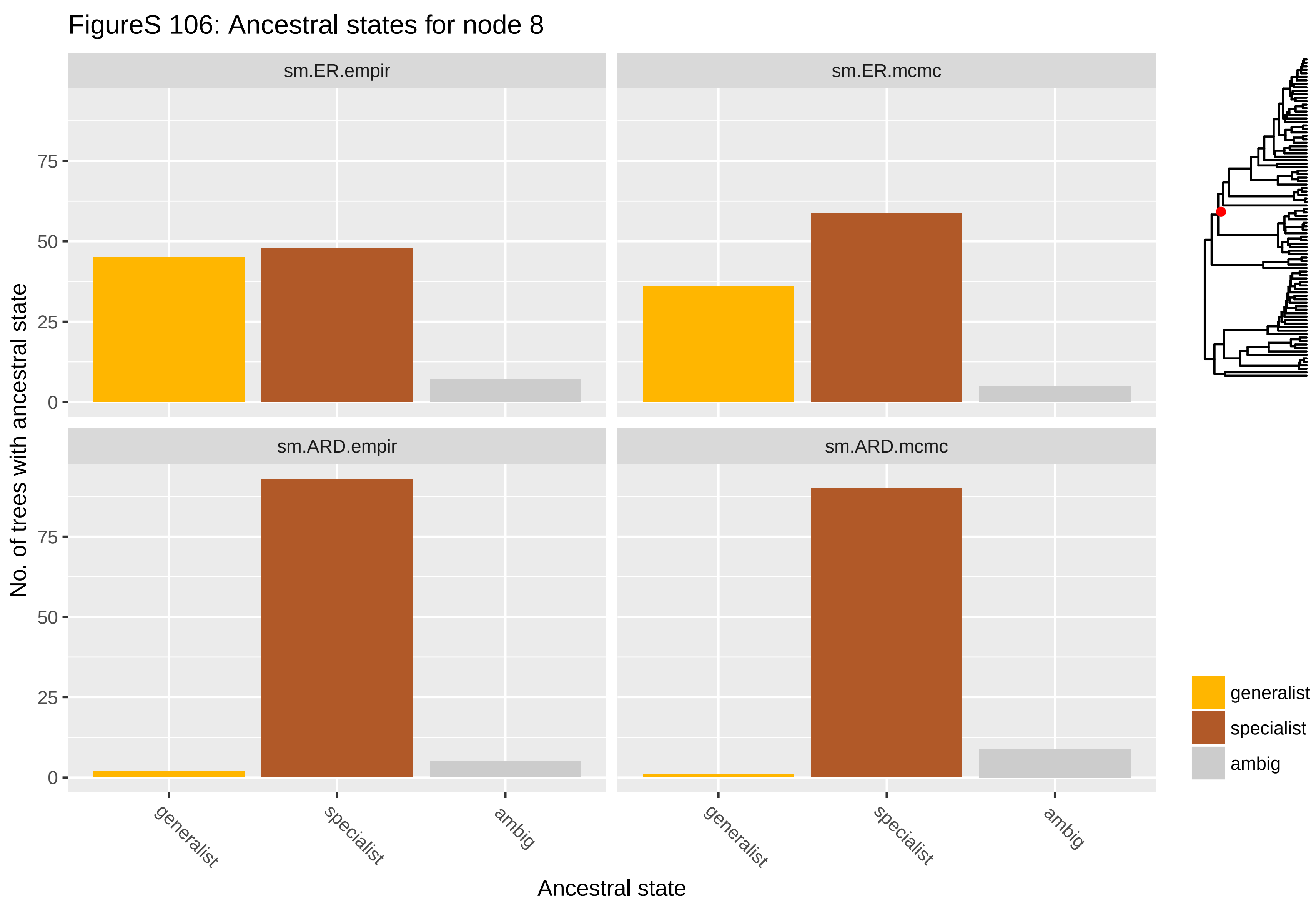

FigureS 106: Ancestral states for node 8
sm.ER.empir
sm.ER.mcmc
sm.ARD.empir
sm.ARD.mcmc
No. of trees with ancestral state
0
25
50
75
generalist
specialist
ambig
Ancestral state
generalist
specialist
ambig

FigureS 107: Ancestral states for node 9

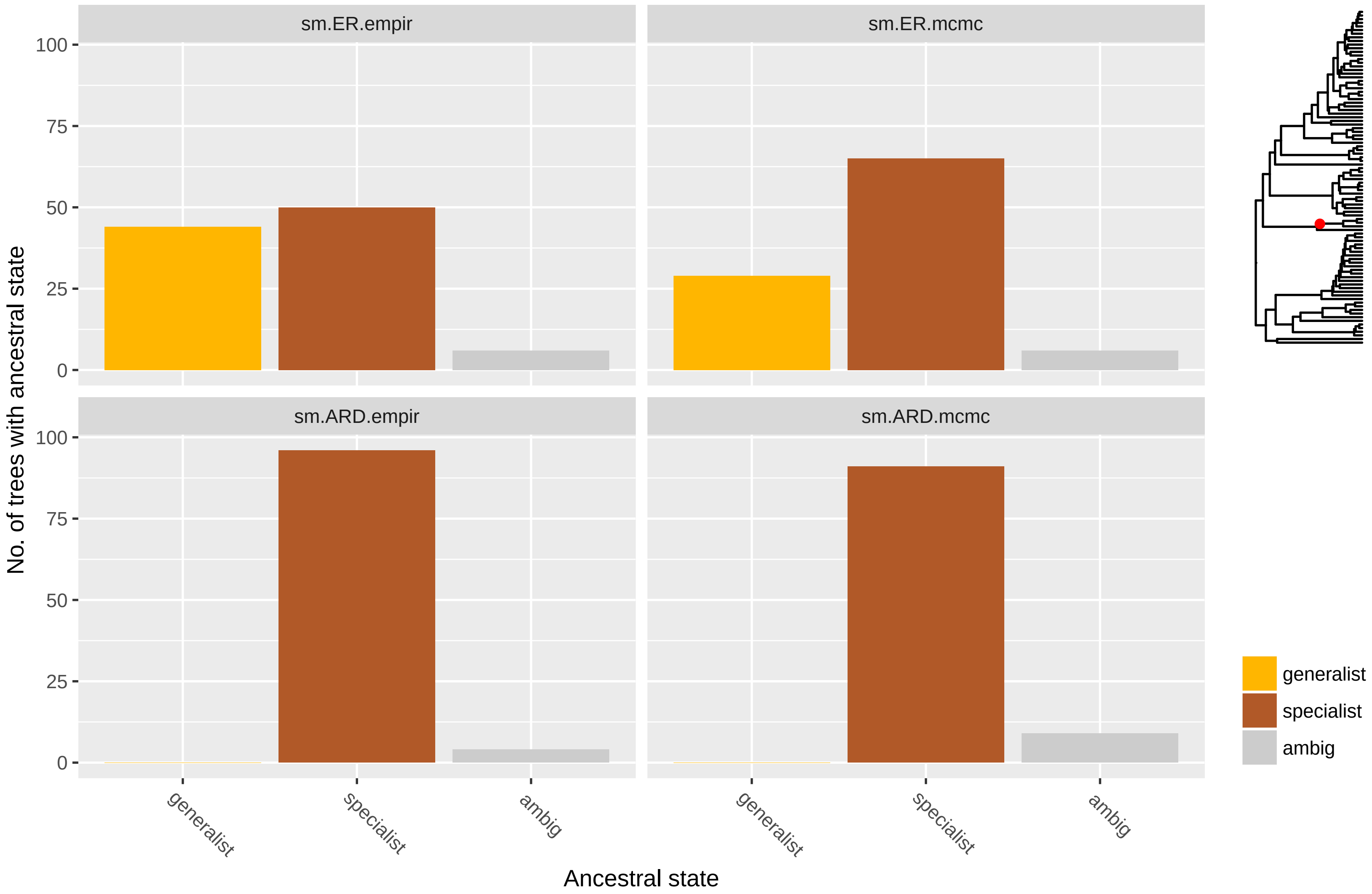

FigureS 108: Ancestral states for node 10

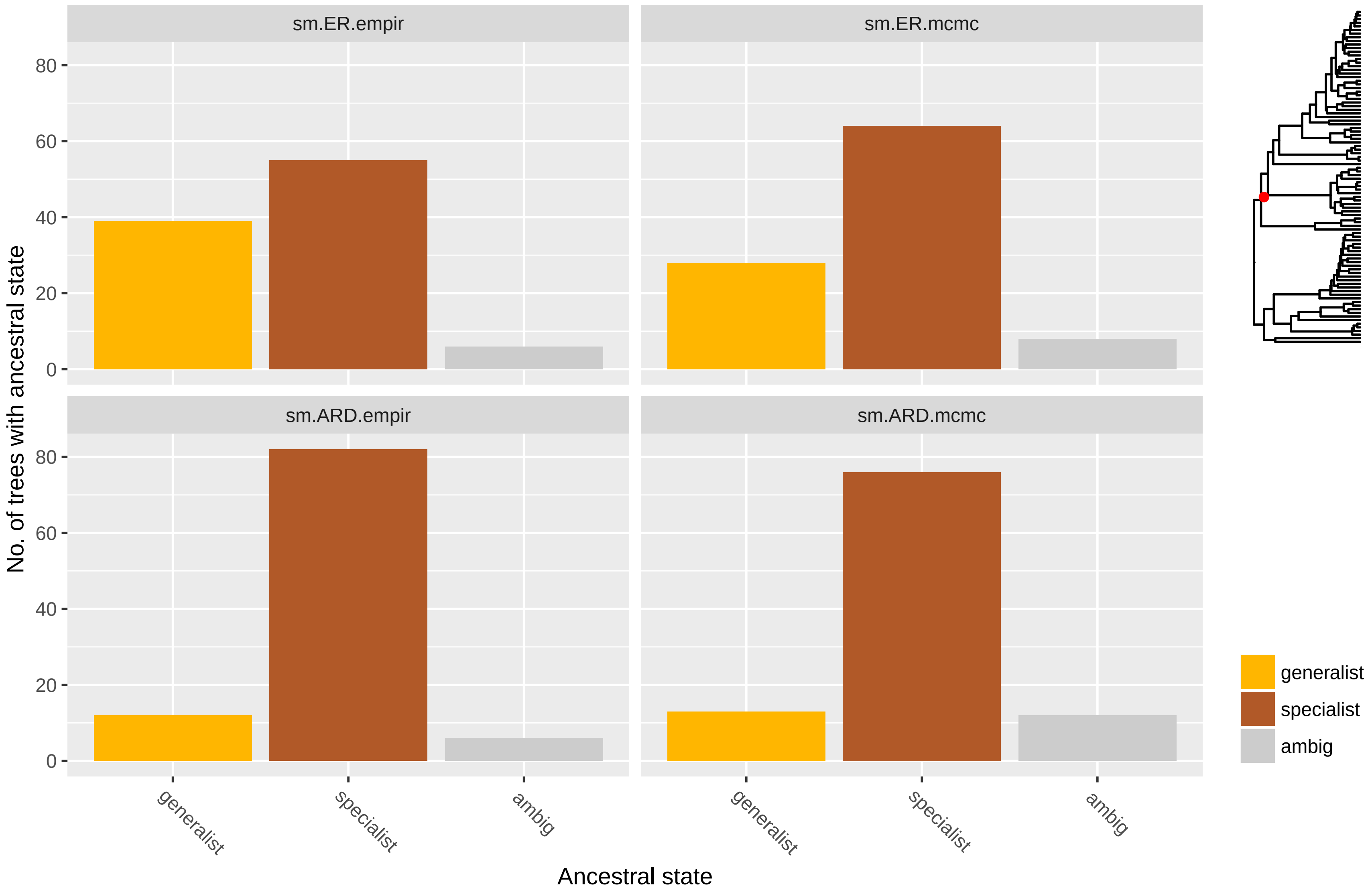

FigureS 109: Ancestral states for node 11

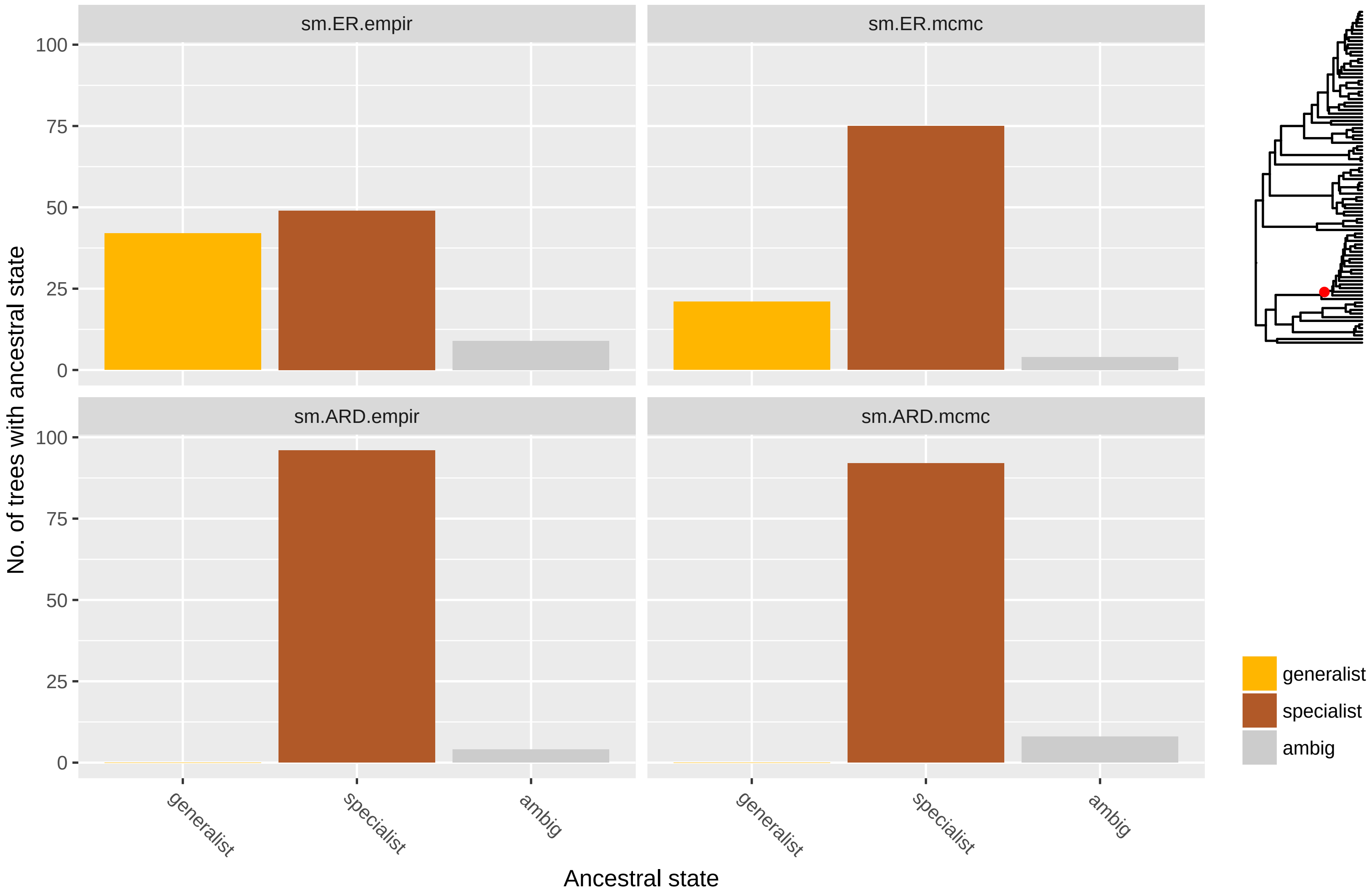

FigureS 110: Ancestral states for node 12

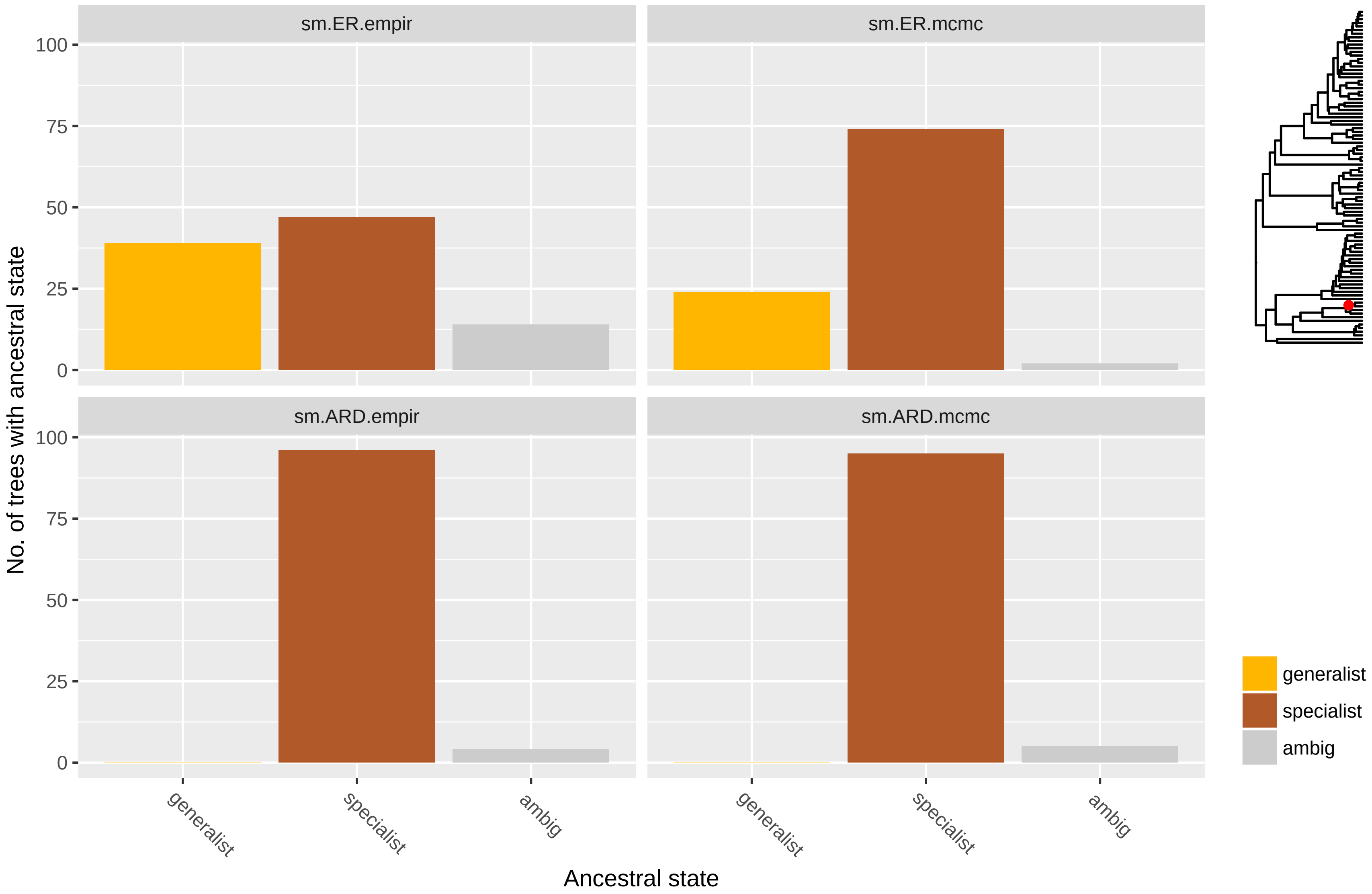

FigureS 111: Ancestral states for node 13

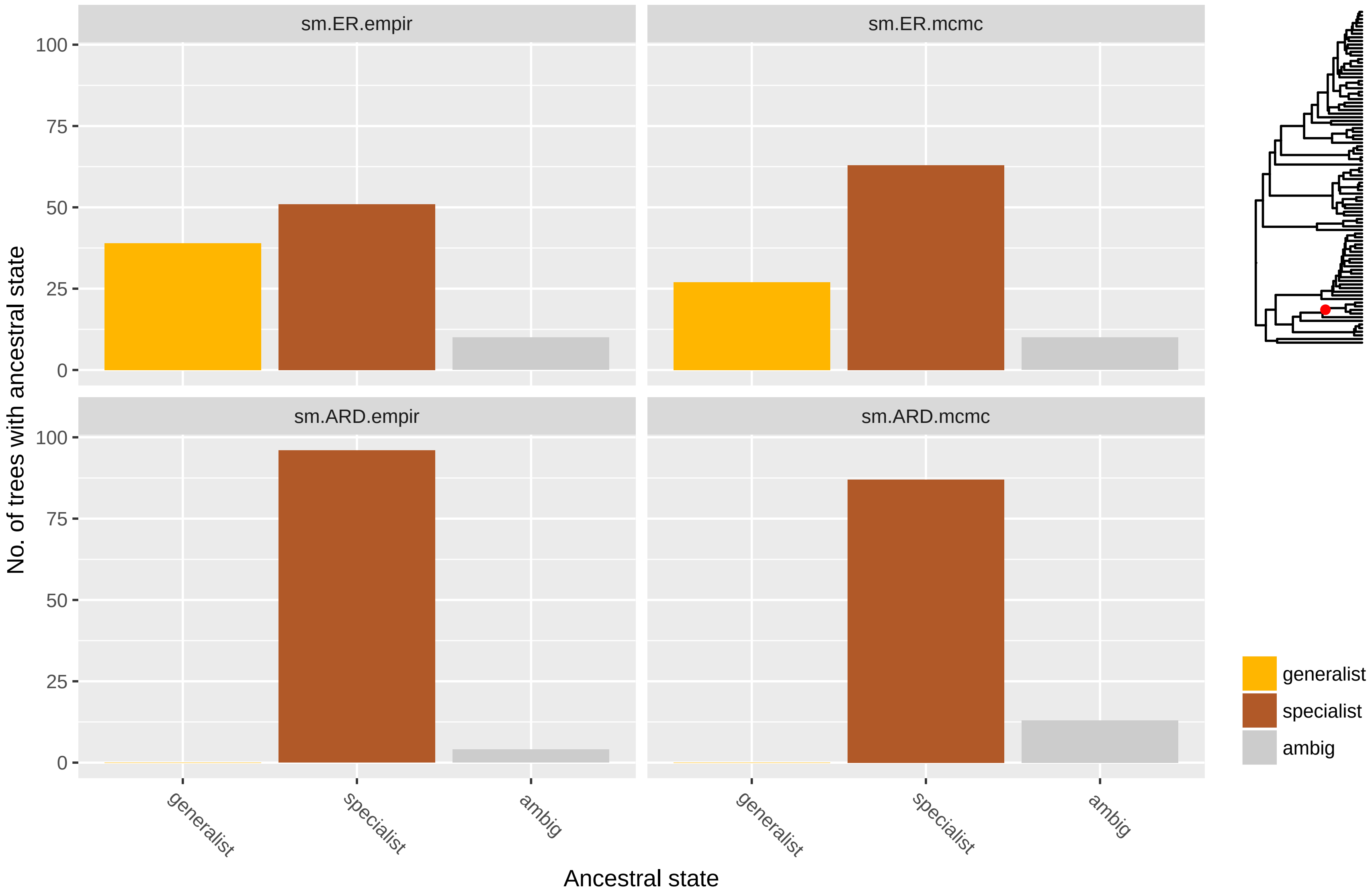

FigureS 112: Ancestral states for node 14

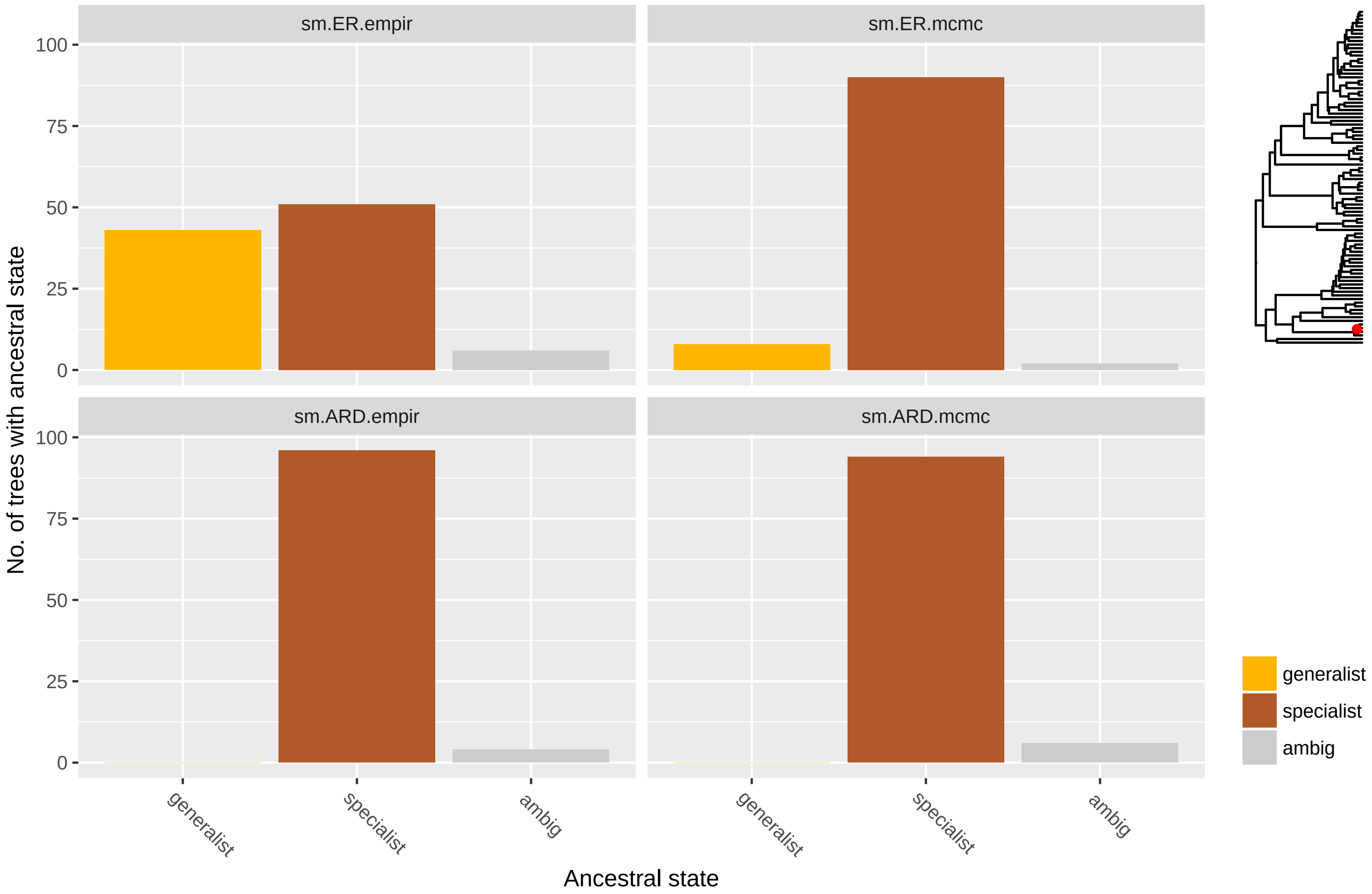

FigureS 113: Ancestral states for node 15

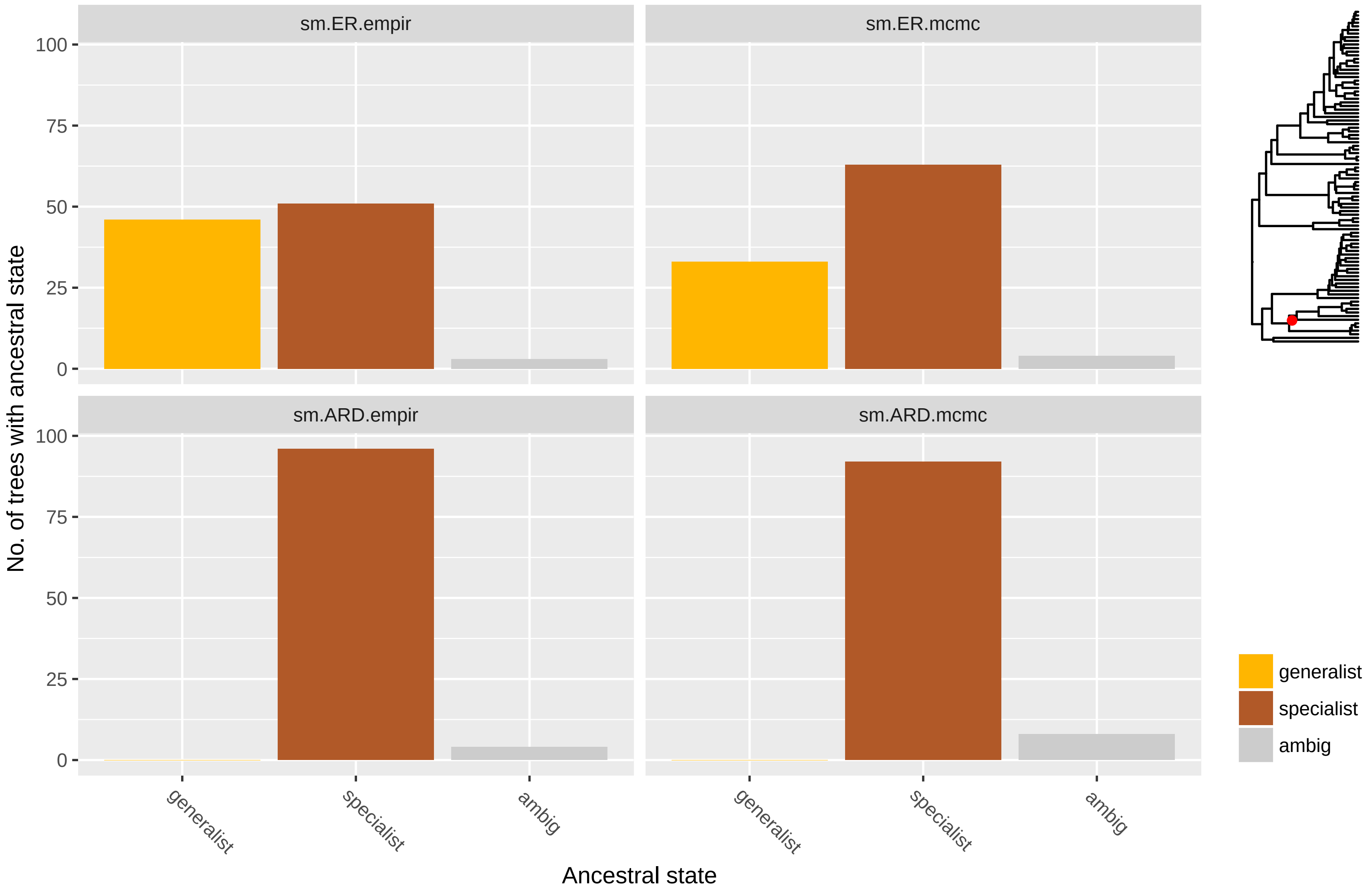

FigureS 114: Ancestral states for node 16

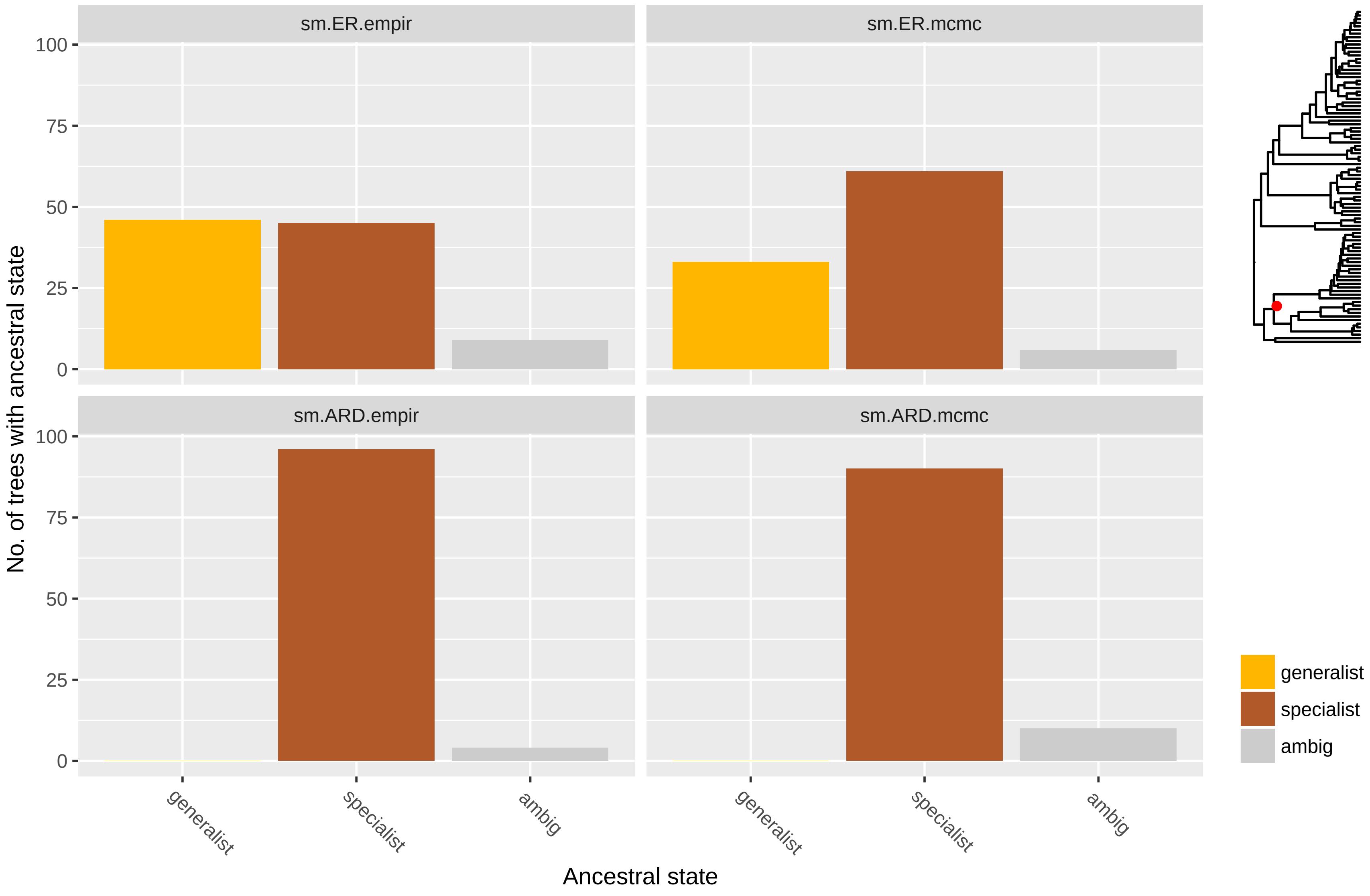

FigureS 115: Ancestral states for node 17

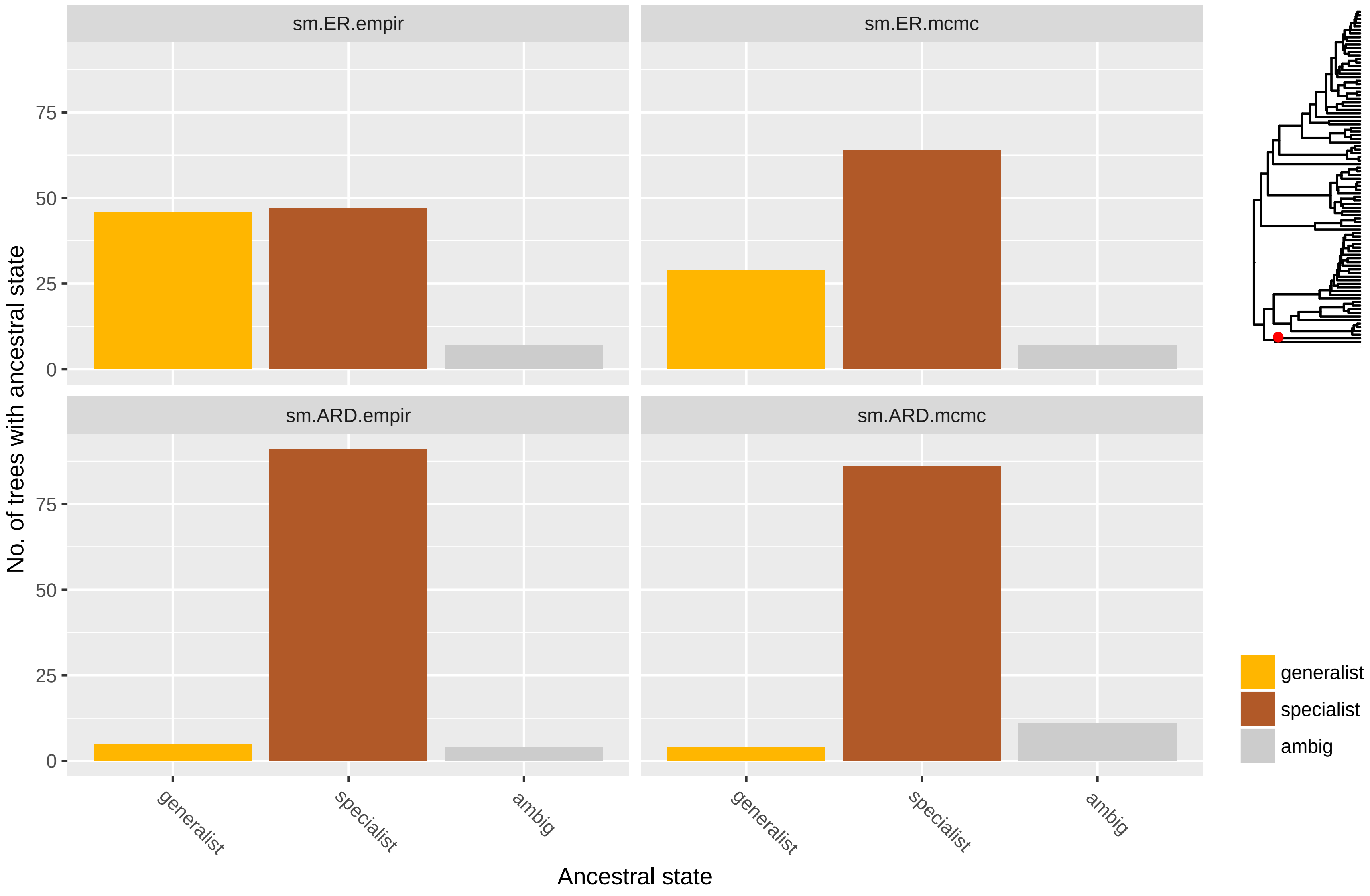

FigureS 116: Ancestral states for node 18

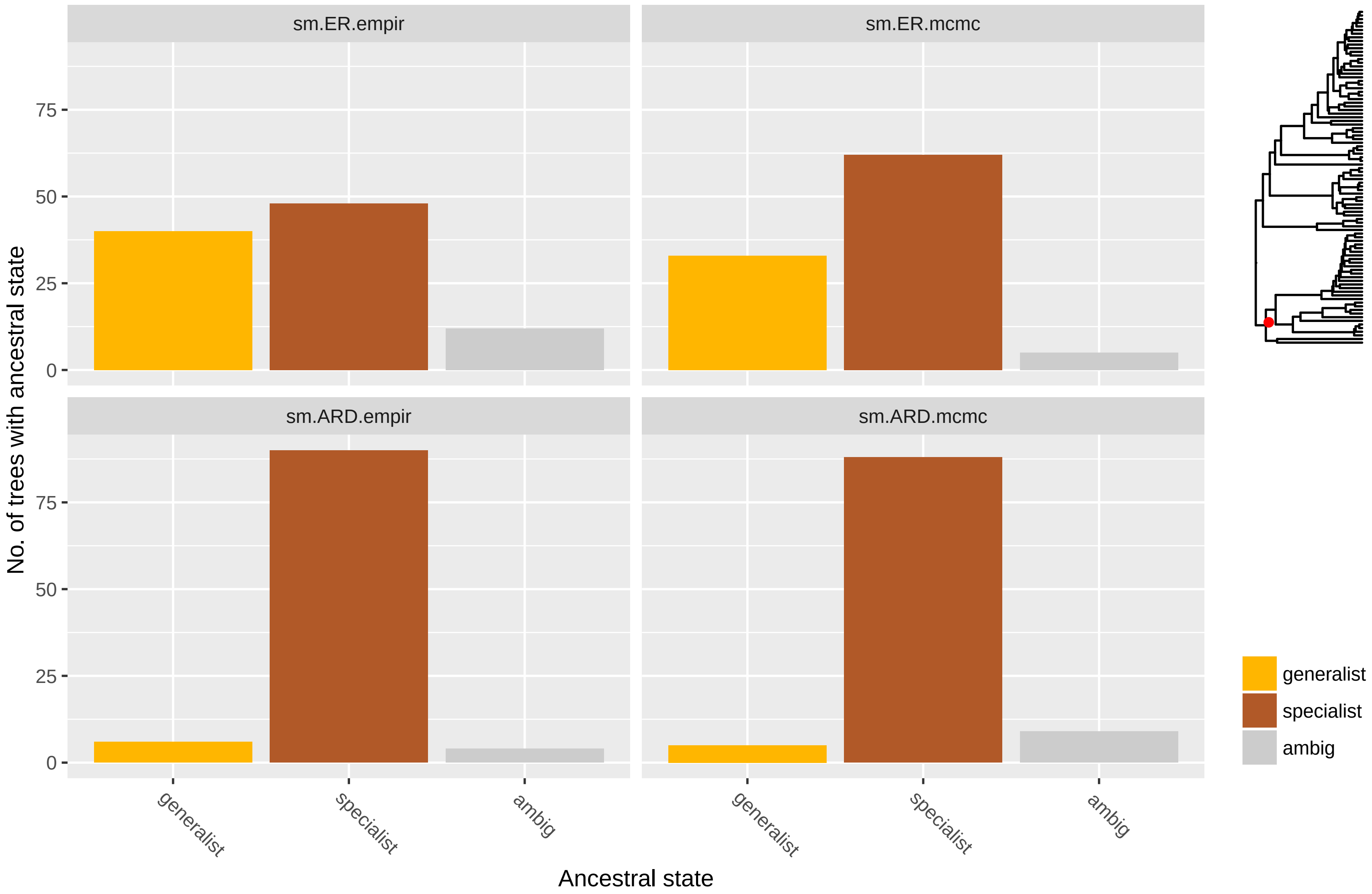

FigureS 117: Ancestral states for node 19

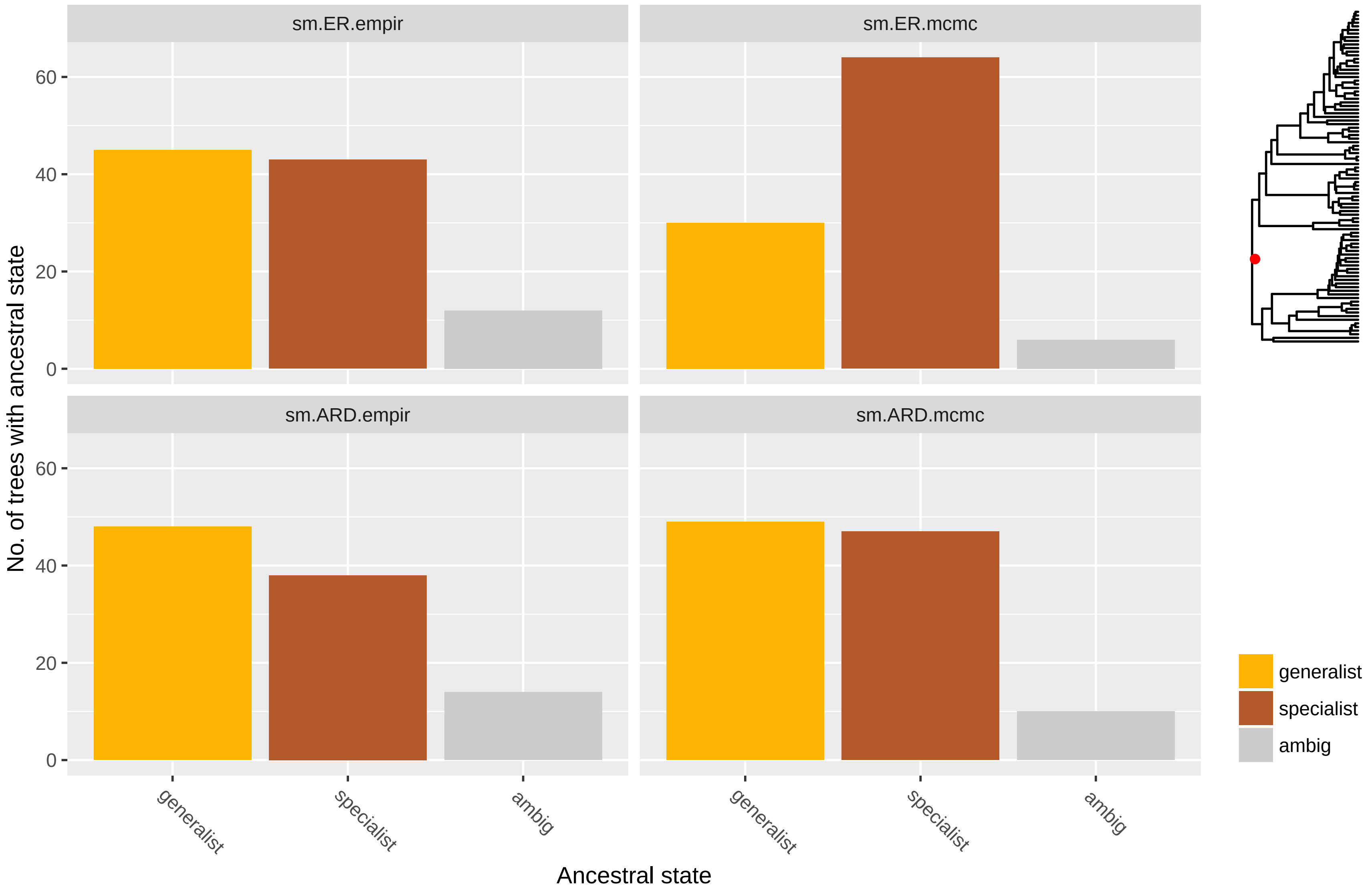

Figure S118 - Individual plots for speciation and extinction rate from MuSSE analyses

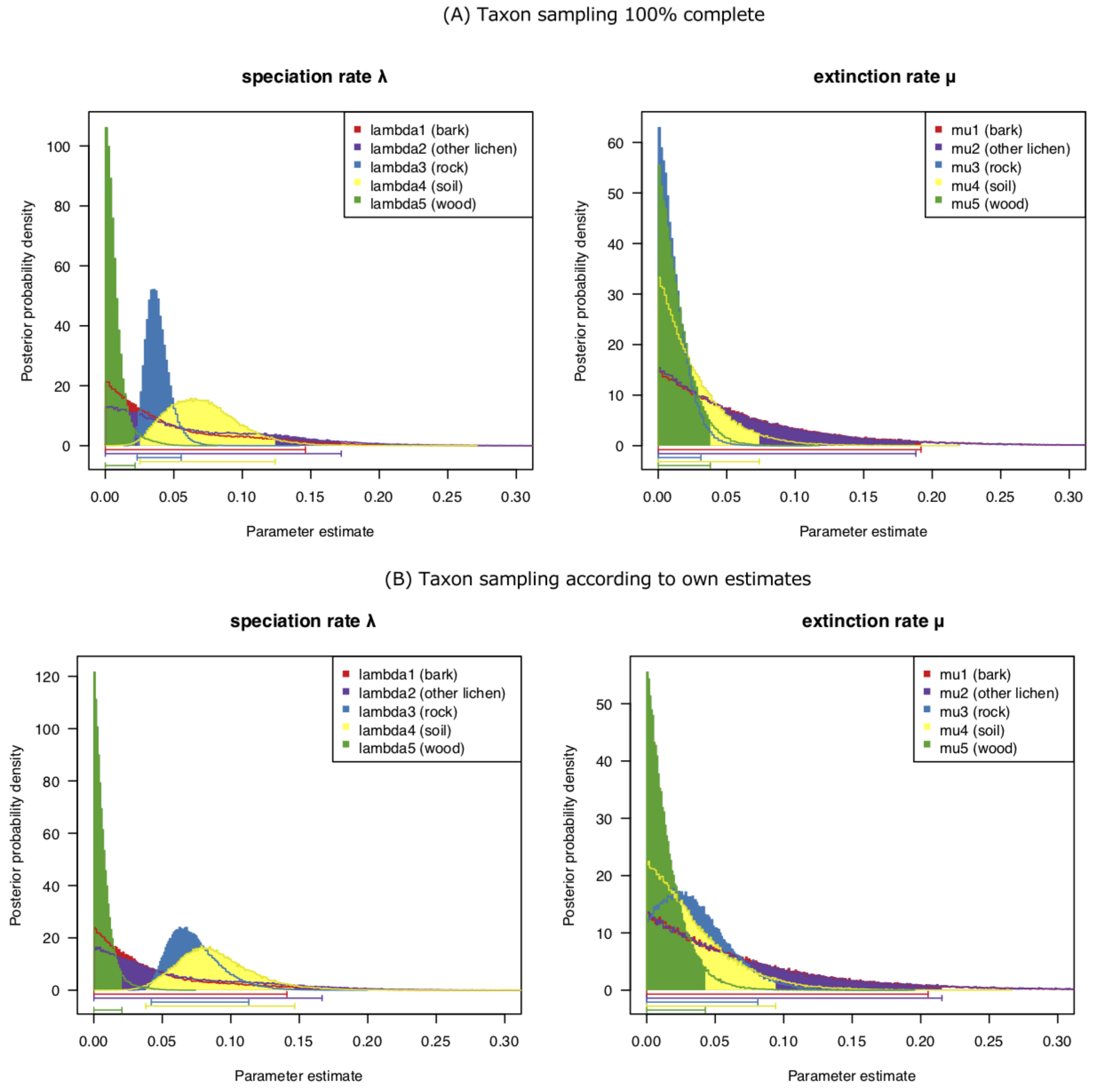

Figure S119 - MuSSE validation simulation

Simulation results to test for elevated type1 error rates in SSE analyses. For details refer to main text.

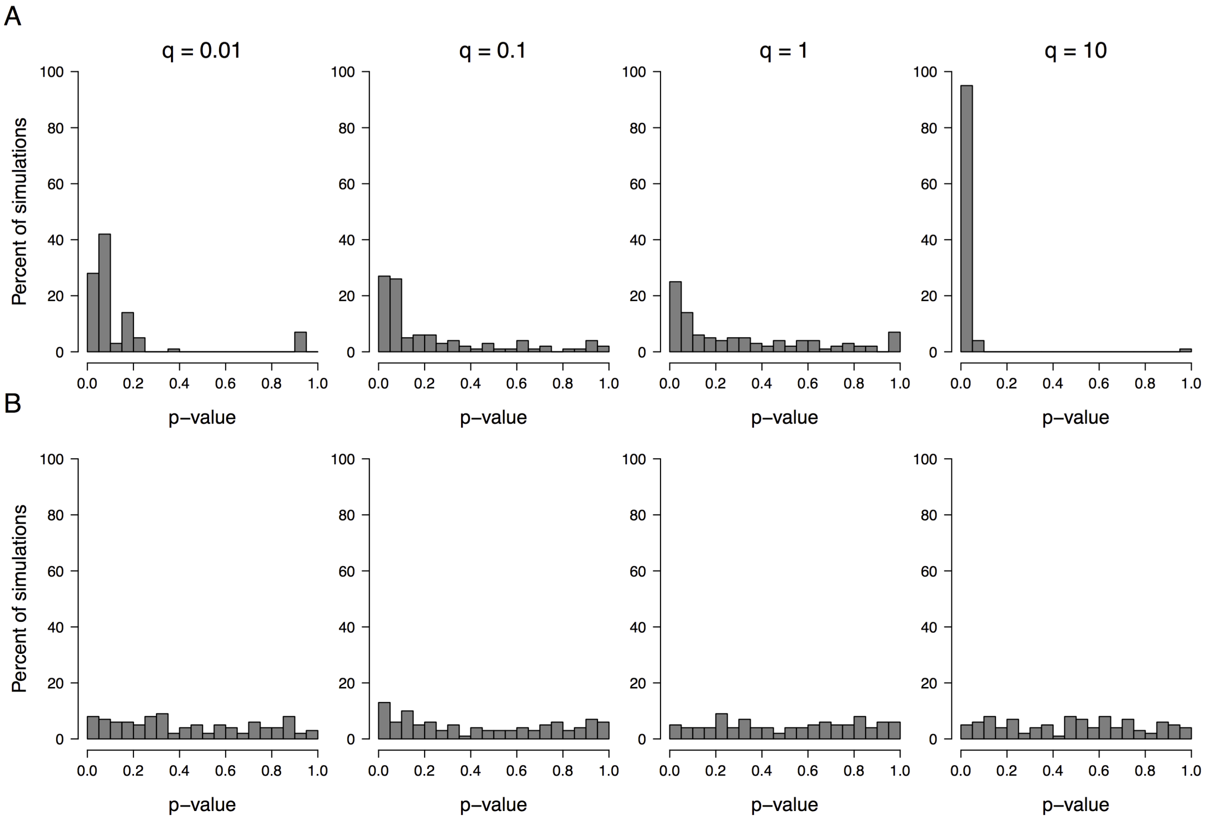
A
q = 0.01
q = 0.1
q = 1
q = 10
Percent of simulations
p−value
B
Percent of simulations
p−value
0
20
40
60
80
100
0.0
0.2
0.4
0.6
0.8
1.0

Figure S120 - BAMM rate through time plot for different trapelioid clades assuming 100% sampling completeness

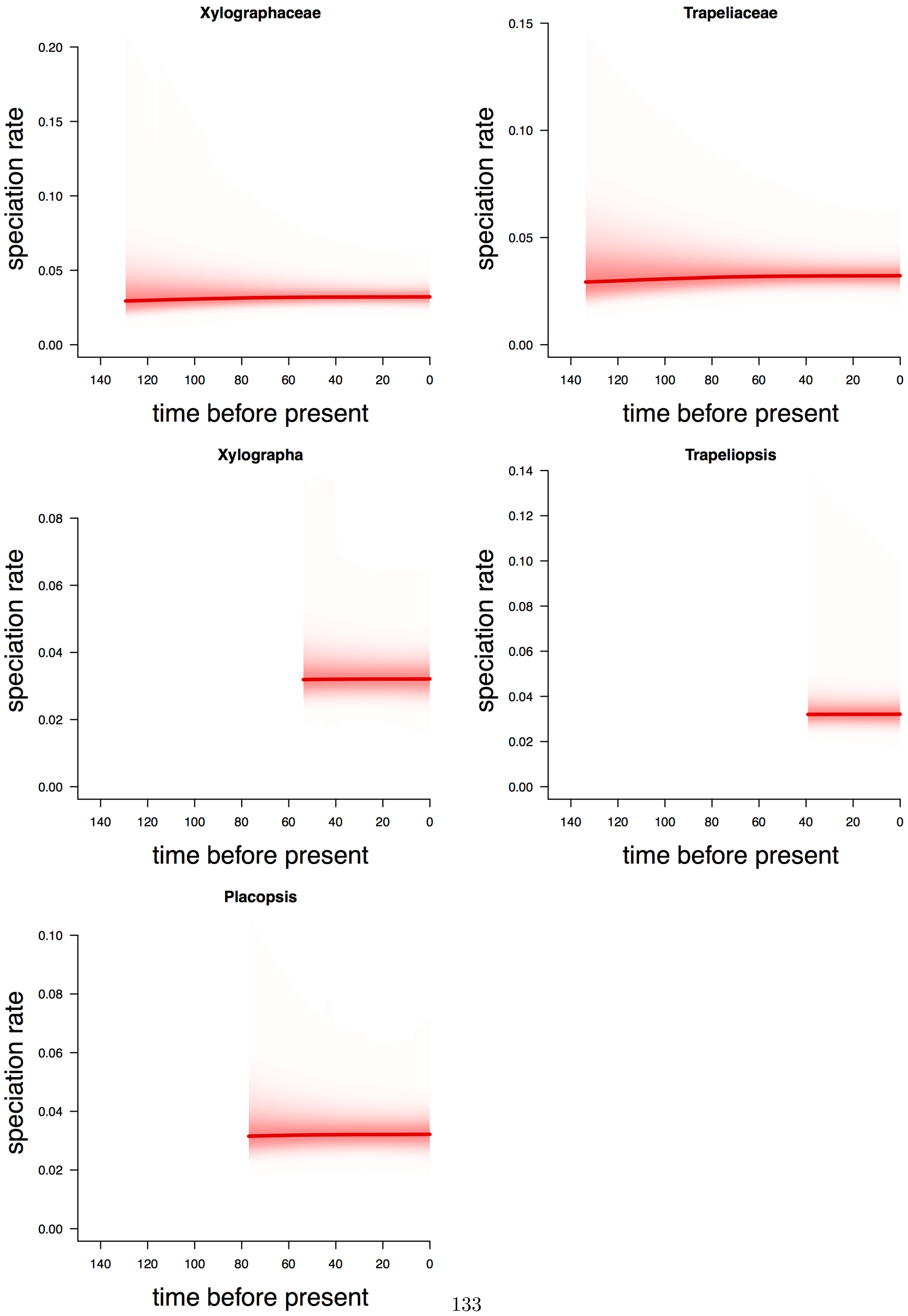

Figure S121 - BAMM rate through time plot for different trapelioid clades assuming sampling completeness according to indexfungorum.org

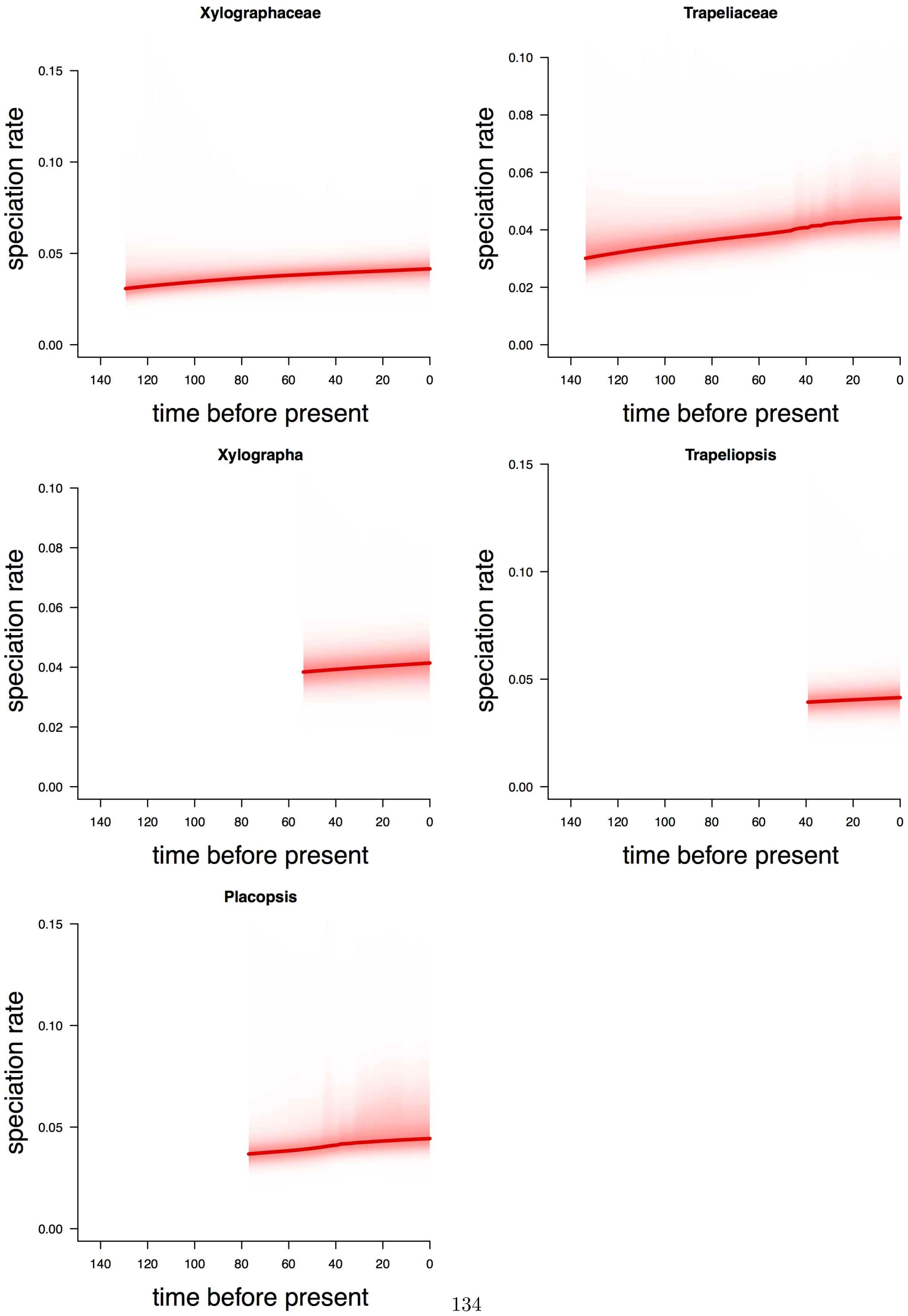

Figure S122 - BAMM rate through time plot for different trapelioid clades assuming sampling completeness according to own diversity estimates

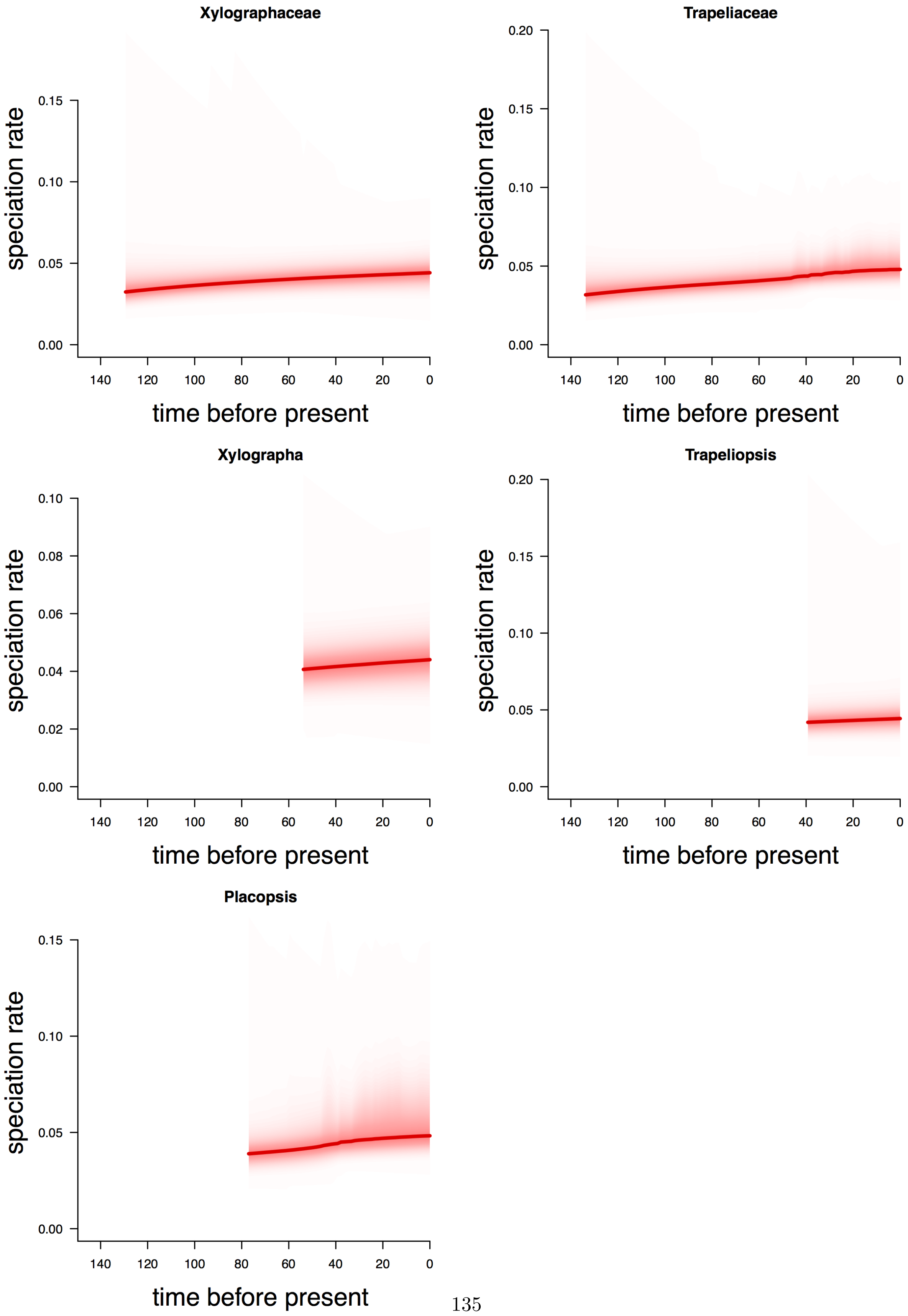

Figure S123 - Results of blocked transition model comparison with ace based on AIC score comparison of thirty models fitted to 100 trapelioid trees

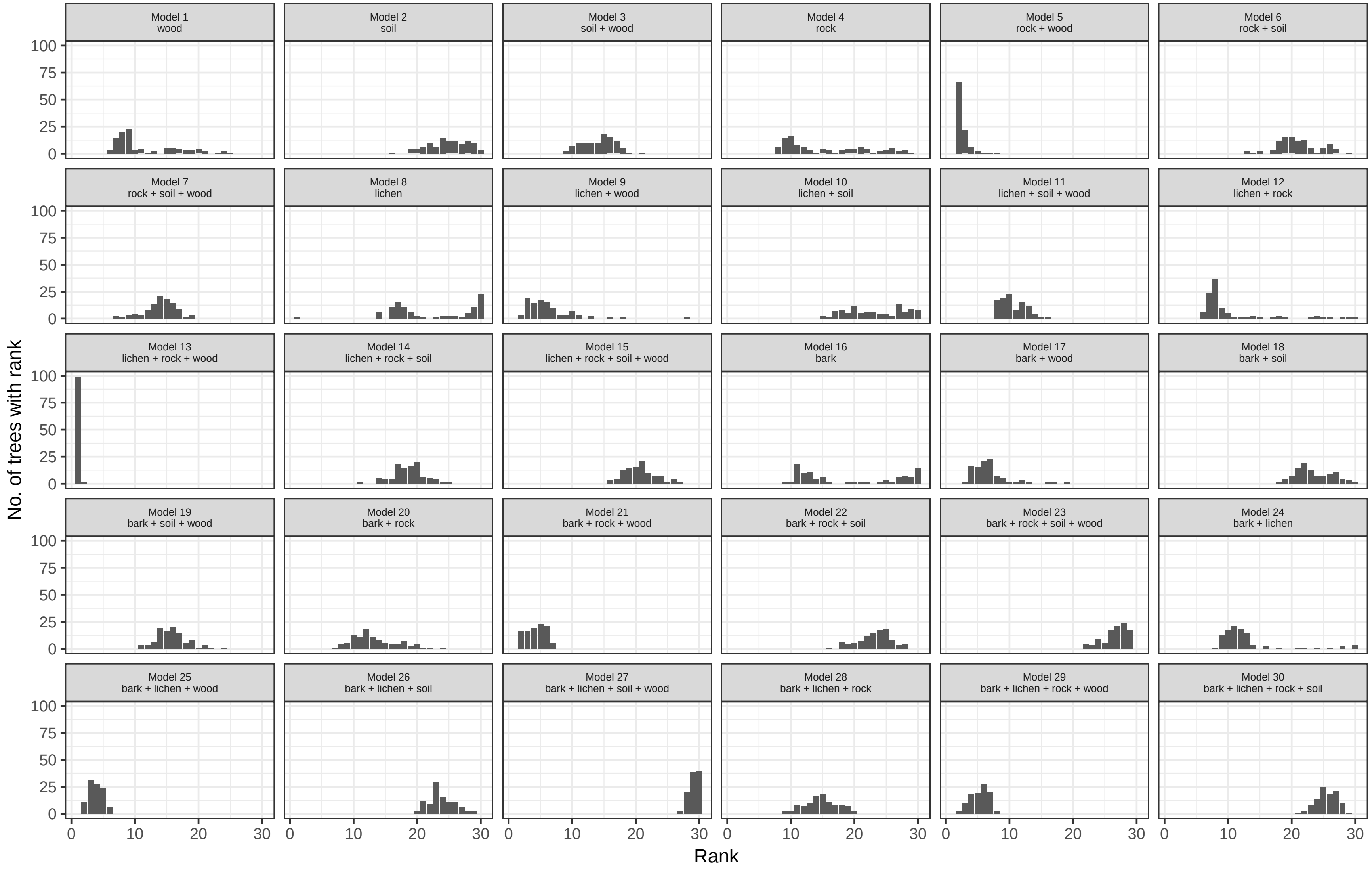

Table S1 - Specimen and sequence vouchers used in this study

For this information please refer to the separately provided spreadsheet: TableS1.xlsx

Table S2 - Mean number of character state transitions for the preferred substrate character set

Data from this table was used to create Figure 3a.

states	to	from	total
bark	3.624654	2.576534	6.201188
lichen	2.221585	1.848812	4.070397
rock	2.208812	6.941881	9.150693
soil	8.150099	9.141188	17.291287
wood	8.905446	4.602080	13.507525

Table S3 - Results from evolutionary dead end scenario analyses in tabular form.

Rank of model (based on AIC score comparison) among all models for one tree / Number of trees for which the model ranked nth place, of 100 trees, based on AIC scores. Data from this table was used to create Figure 2 and Figure S123.

	Model.No.	Description	1	2	3	4	5	6	7	8	9	10	11	12	13	14	15	16	17	18	19	20	21	22	23	24	25	26	27	28	29	30	mean AIC*
1	13	lichen + rock + wood	99	1																													104,5443
2	5	rock + wood		66	22	6	2	1	1	1																							107,8556
3	25	bark + lichen + wood		11	31	27	24	6																									109,2197
4	21	bark + rock + wood		16	16	19	23	21	5																								109,3961
5	29	bark + lichen + rock + wood			3	10	18	19	27	20	3																						110,1557
6	9	lichen + wood		3	19	14	17	15	10	3	3	7	3		2			1		1										1			111,6885
7	17	bark + wood			2	16	15	21	23	7	5	2	1	3	2			1	1		1												111,9313
8	12	lichen + rock						6	24	37	10	5	1	1	1	2	1		1	2	1				1	2	1	1		1	1	1	115,9281
9	11	lichen + soil + wood								17	19	23	8	15	12	4	1	1															117,2357
10	1	wood						3	14	20	23	3	4	1	2		5	5	4	3	3	4	2	1	2	1							117,3383
11	20	bark + rock							1	4	5	13	11	18	11	8	5	4	4	7	2	4	1	1		1							120,0705
12	24	bark + lichen								1	13	17	21	18	15	3		2		1			1	1		1		1		2		3	120,676
13	7	rock + soil + wood							2	1	3	4	3	8	13	21	18	14	9	1	3												120,8881
14	3	soil + wood									2	7	10	10	10	10	18	15	11	5	1		1										121,0753
15	28	bark + lichen + rock									2	2	8	7	10	16	18	11	8	8	7	2											121,6284
16	19	bark + soil + wood											3	3	6	19	16	20	14	5	8	1	3	1		1							122,5742
17	4	rock								6	14	16	8	6	3	1	4	3	1	3	4	4	6	4	1	2	3	5	2	3	1		122,5929
18	14	lichen + rock + soil											1			5	4	4	18	14	16	20	6	5	4	1	2						126,3328
19	15	lichen + rock + soil + wood																3	4	12	14	15	21	10	7	7	2	4	1				128,2759
20	6	rock + soil													2	1	2		3	12	15	15	12	13	5	1	5	9	4		1		129,0554
21	22	bark + rock + soil																1		6	4	5	7	12	15	17	18	8	3	4			131,5949
22	26	bark + lichen + soil																				3	12	9	29	15	11	11	6	2	2		132,1792
23	18	bark + soil																		1	4	7	14	19	13	7	7	9	11	4	3	1	133,2124
24	30	bark + lichen + rock + soil																					1	3	8	13	25	18	21	10	1		134,4323
25	23	bark + rock + soil + wood																						4	3	9	5	17	21	24	17		136,923
26	2	soil																1			4	4	6	10	6	14	11	11	9	11	10	3	137,2116
27	16	bark									1	1	18	10	11	4	6	2			2	2	1	2		1	3	2	6	7	6	14	138,0049
28	10	lichen + soil															2	1	7	8	5	12	5	6	6	4	4	2	13	6	9	8	140,5222
29	8	lichen	1													6		11	15	11	6	2	1		1	2	2	2	1	5	11	23	142,6968
30	27	bark + lichen + soil + wood																											2	20	38	40	152,0688

* per model for all trees

Table S4 - Individual models of dead end analyses for which ace failed to return results

model.no.	Description	tree.no.
28	bark + lichen + rock	34
9	lichen + wood	68
10	lichen + soil	71
5	rock + wood	72
25	bark + lichen + wood	84
16	bark	91
10	lichen + soil	95

Table S5a - Summary MuSSE diversification rate 100%

	bark	lichen	rock	soil	wood	mean	stddev
bark		p=1	p=1	p=1	p=1	-0.0158288	0.0791713
lichen	p<2.2e-16		p=1	p=1	p<2.2e-16	0.0024461	0.0852084
rock	p<2.2e-16	p<2.2e-16		p=1	p<2.2e-16	0.0265715	0.0076929
soil	p<2.2e-16	p<2.2e-16	p<2.2e-16		p<2.2e-16	0.0458740	0.0258588
wood	p<2.2e-16	p=1	p=1	p=1		-0.0067468	0.0132246

Interpretation: Can we reject the H0 that (row) is not significantly larger then (col). When $p < 0.05$ value in row is significantly larger then value in col.

Table S5b - Summary MuSSE diversification own diversity estimates

	bark	lichen	rock	soil	wood	mean	stddev
bark		p=1	p=1	p=1	p=1	-0.0253580	0.0809662
lichen	p<2.2e-16		p=1	p=1	p<2.2e-16	-0.0139080	0.0900584
rock	p<2.2e-16	p<2.2e-16		p=1	p<2.2e-16	0.0378680	0.0117604
soil	p<2.2e-16	p<2.2e-16	p<2.2e-16		p<2.2e-16	0.0544373	0.0236842
wood	p<2.2e-16	p=1	p=1	p=1		-0.0081949	0.0148699

Interpretation: Can we reject the H0 that (row) is not significantly larger then (col). When $p < 0.05$ value in row is significantly larger then value in col.

Table S6 - Overview about the number of methods which recovered the most frequent ancestral state for 10 nodes along the trapelioid tree

	Node	consensus state substrate character	no. of methods recovering substrates (total = 30)	consensus state ecol. strategy	no. of methods recovering ecological amplitude (total = 20
1	1	rock	29	specialist	17
2	2	rock	29	specialist	17
3	3	rock	29	specialist	17
4	4	soil	28	generalist	15
5	5	rock	27	specialist	17
6	6	rock	27	specialist	17
7	7	soil	22	generalist	15
8	8	rock	16	specialist	16
9	9	rock	29	specialist	17
10	10	rock	18	specialist	17
11	11	wood	28	specialist	17
12	12	bark	22	specialist	17
13	13	rock	18	specialist	17
14	14	rock	29	specialist	17
15	15	rock	18	specialist	17
16	16	rock	17	specialist	16
17	17	rock	17	specialist	17
18	18	rock	17	specialist	17
19	19	rock	16	specialist	13
